# miR-27a is a master regulator of metabolic reprogramming and chemoresistance in colorectal cancer

**DOI:** 10.1038/s41416-020-0773-2

**Published:** 2020-03-05

**Authors:** Giovannina Barisciano, Tommaso Colangelo, Valeria Rosato, Livio Muccillo, Maria Letizia Taddei, Luigi Ippolito, Paola Chiarugi, Mario Galgani, Sara Bruzzaniti, Giuseppe Matarese, Matteo Fassan, Marco Agostini, Francesca Bergamo, Salvatore Pucciarelli, Annalucia Carbone, Gianluigi Mazzoccoli, Vittorio Colantuoni, Fabrizio Bianchi, Lina Sabatino

**Affiliations:** 1 Department of Sciences and Technologies, University of Sannio, Via Francesco de Sanctis, 82100 Benevento, Italy; 2 Fondazione IRCCS Casa Sollievo della Sofferenza, Cancer Biomarkers Unit, Viale Padre Pio, 7, 71013 San Giovanni Rotondo, FG Italy; 3 Department of Experimental Biomedical and Clinical Medicine, University of Florence, Viale Morgagni 50, 50134 Florence, Italy; 4 Department of Molecular Medicine and Medical Biotechnologies “Federico II”, University, Naples Via S. Pansini, 5, 80131 Naples, Italy; 5 Department of Biology, “Federico II” University, 80126 Naples, Italy; 6 Laboratory of Immunology, Institute of Experimental Endocrinology and Oncology (IEOS-CNR), Via S. Pansini, 5, 80131 Naples, Italy; 7 Department of Medicine (DIMED), Surgical Pathology Unit, University of Padua, Via Giustiniani, 2, 35128 Padua, Italy; 8 Department of Surgery, Oncology and Gastroenterology, First Surgical Clinic Section, University of Padua, Via Giustiniani, 2, 35128 Padua, Italy; 9 Department of Clinical and Experimental Oncology, Unit of Medical Oncology 1, Veneto Institute of Oncology IOV, IRCCS, Via Gattamelata, 64, 35128 Padua, Italy; 10 Fondazione IRCCS Casa Sollievo della Sofferenza, Division of Internal Medicine and Chronobiology Unit, Viale Padre Pio, 7, 71013 San Giovanni Rotondo, FG Italy

**Keywords:** Cancer metabolism, miRNAs

## Abstract

**Background:**

Metabolic reprogramming towards aerobic glycolysis in cancer supports unrestricted cell proliferation, survival and chemoresistance. The molecular bases of these processes are still undefined. Recent reports suggest crucial roles for microRNAs. Here, we provide new evidence of the implication of miR-27a in modulating colorectal cancer (CRC) metabolism and chemoresistance.

**Methods:**

A survey of miR-27a expression profile in TCGA-COAD dataset revealed that miR-27a-overexpressing CRCs are enriched in gene signatures of mitochondrial dysfunction, deregulated oxidative phosphorylation, mTOR activation and reduced chemosensitivity. The same pathways were analysed in cell lines in which we modified miR-27a levels. The response to chemotherapy was investigated in an independent cohort and cell lines.

**Results:**

miR-27a upregulation in vitro associated with impaired oxidative phosphorylation, overall mitochondrial activities and slight influence on glycolysis. miR-27a hampered AMPK, enhanced mTOR signalling and acted in concert with oncogenes and tumour cell metabolic regulators to force an aerobic glycolytic metabolism supporting biomass production, unrestricted growth and chemoresistance. This latter association was confirmed in our cohort of patients and cell lines.

**Conclusions:**

We disclose an unprecedented role for miR-27a as a master regulator of cancer metabolism reprogramming that impinges on CRC response to chemotherapy, underscoring its theragnostic properties.

## Background

Metabolic rewiring is a hallmark of cancer.^1,2^ Normal cells under aerobic conditions process glucose to pyruvate via glycolysis in the cytosol and, subsequently, to carbon dioxide in the mitochondria. Under anaerobic conditions, although glycolysis is favoured, only a relatively little amount of pyruvate is conveyed to the oxygen-consuming mitochondria. Cancer cells redirect their energy metabolism by restricting it largely to glycolysis, even in the presence of oxygen, leading to a process called “aerobic glycolysis” (Warburg effect).^3^ To support unrestrained cell growth and compensate for the lower efficiency of ATP production supplied by glycolysis, cancer cells must activate alternative routes to exploit glycolytic intermediates for anabolic pathways (i.e. the pentose phosphate pathway). In addition, the accumulation of pyruvate is rapidly converted to lactate which is then secreted out of the cell where it can inhibit the immune response, activate endothelial cells and fibroblasts, thus favouring cancer progression.^4,5^ Metabolic rewiring of cancer cells has been coupled with mechanisms implicated in chemoresistance.^6,7^ The response rates of current therapies in patients with metastatic cancers are, in fact, quite poor also because responsive patients eventually acquire secondary resistance to anti-cancer drugs.^8,9^

Although these events (glycolytic switch, metabolic rewiring and chemotherapy resistance) have been tightly linked, a clear representation of the mechanisms involved is still missing. In general, metabolic reprogramming can be orchestrated by cancer genes such as *K-RAS, c-MYC, PI3K, TP53, AMPK* and their signalling, which enhance the glycolytic and glutamine pathways to support biosynthesis, redox homoeostasis, cell growth, survival and enhanced drug resistance.^10–13^ Furthermore, other global modulators of cell biofunctions such as mTOR and HIF-1α, may accentuate these effects by acting pleiotropically to modify the overall cell metabolism, biosynthetic pathways and drug response.^14,15^

MicroRNAs (miRNA) are emerging as a class of master regulators of a multitude of cell processes.^16^ In the last years, several miRNAs were shown to play key roles in modulating normal and cancer cell metabolism through targeting several transporters, metabolic enzymes and oncogenes, either directly or indirectly.^17,18^ Consistently, changes in the expression of many miRNAs were functionally correlated with various types of human cancers, including CRC,^19,20^ enhancing resistance to chemotherapy.^21–24^ Among these, the miR-23a~27a~24-2 cluster was shown to have oncogenic function;^25^ we previously showed that miR-27a is upregulated in CRC tissues and targets genes involved in cell proliferation, tumour growth and immune evasion.^26,27^

Here we provide the first evidence that miR-27a acts as a hub to orchestrate pathways connecting metabolic rewiring (forced aerobic glycolysis, impaired AMPK and activated mTOR and oncogenes crosstalk) with chemoresistance in CRC.

## Methods

### Cell culture and proliferation assay

The human CRC cell lines HCT116, SW480 and HT29 were acquired from the American Type Culture Collection (ATCC, Rockville, MD, USA) and cultured as described in refs. ^26,27^ To evaluate the proliferation ability, HCT116 and HT29 clones were seeded into 96-well plates in 90 μL complete RPMI medium, at a density of 2 × 10^4^ cells/well, and incubated overnight at 37 °C in 5% CO_2_ atmosphere to enable cell adhesion. Cell number and density of viable cells were determined at 0, 24, 48, 72 h using PrestoBlue™ Cell Viability (Thermo Fisher Scientific, Waltham, MA, USA) assay according to the manufacturer’s instructions. Three biological replicates were prepared, and each condition assayed in triplicate; the results are expressed as mean ± standard deviation (SD).

### Migration and Invasion assay

Cell motility was evaluated by the wound-healing assay; the edges of the initial scratch are indicated with a black line in the figure and the wound closure values refer to this initial position. The cell free area (percentage of control at 0 h) was then measured by ImageJ software (v. 1.8.0, National Institutes of Health Image). All experiments were performed at least three times in triplicates. Invasion was evaluated by the Transwell assay. In all, 1 × 10^5^ HCT116- and HT29-clones were resuspended in serum free cell culture media and seeded onto Matrigel-coated Transwell filters (8-µm pore size) (Costar, Corning Inc., Corning, NY, USA) coated with 200 μl of Corning® Matrigel® matrix (final concentration of 250 μg/mL) according to manufacturer’s protocol. The outer chamber was filled with 600 μl of medium containing 20% FBS and incubated at 37 °C for 48 h. Non-invading cells on the upper surface of the insert were removed with cotton swab, while those on the lower surface (invasive cells) were fixed and stained with 300 nM DAPI solution. The number of invading cells from twenty fields of each of three separate experiments was counted under the fluorescent microscope using a 10X objective and fields pictures analysed by using ImageJ software.

### RNA extraction and qRT-PCR analysis

Total RNA was extracted using TRIZOL® Reagent (Invitrogen, Carlsbad, CA, USA) following manufacturer’s instruction. The mature miR-27a-3p was detected and quantified by NCode miRNA qRT-PCR method (Invitrogen) following the manufacturer’s instructions, as reported.^26,27^

### Lentiviral infection

Lentiviral constructs overexpressing (Cat# PMIRH27a-onlyPA-1, System Biosciences, Mountain View, CA, USA), or functionally knocking-down miR-27a (Cat# MZIP27a-PA-1, System Biosciences), along with the corresponding controls (Cat# PMIRH000-PA-1 and MZIP000-PA-1, System Biosciences) were transduced and packaged in 293T cells. Stable cell lines (HCT116, SW480 or HT29) overexpressing or silencing miR-27a or the corresponding controls were generated *via* lentiviral transduction, in the presence of polybrene (8μg/ml) (Sigma-Aldrich, S.Louis, MO, USA), and selected with puromycin or by flow cytometry sorting for GFP positive populations (the MoFlo Astrios EQ, Cell Sorter, Beckman Coulter, Indianapolis, IN, USA).

### Oxygen consumption rate and extracellular acidification rate measurements

Real time oxygen consumption rate (OCR) and extracellular acidification rate (ECAR) measurements were made using an XF-96 Extracellular Flux Analyzer (Seahorse Bioscience, Agilent, Santa Clara, CA, USA) according to the manufacturer’s instructions.

### Western blot analysis

Protein extracts from cell lines or tumour extracts were analysed as reported.^26^ The antibodies used are listed in Supplementary Table 1. The bands obtained were densitometrically estimated by Imagelab software (BIO-RAD, Hercules, CA, USA) and, subsequently, analysed by GraphPad Prism 6 (GraphPad Software Inc., San Diego, CA, USA).

### PKM2 oxidation by carboxymethylation assay and intracellular reactive oxygen species assessment

PKM2 oxidation and reactive oxygen species (ROS) quantification were assayed as previously described in ref. ^28^

### Citrate synthase activity assay

Citrate Synthase Activity was measured in cell lysates of HCT116 miR-27a_KD and CTRL_KD cells by MitoCheck Citrate Synthase Activity Assay Kit (Cayman Chemical, Ann Arbor, MI, USA) following the manufacturer’s instructions. Citrate synthase was expressed as µmols/min/ml.

### ATP assay and pentose phosphate pathway activity

Intracellular ATP levels were measured in lysates of HCT116 miR-27a_KD and CTRL_KD cells as reported in refs. ^26,27^ PPP activity was evaluated as described in ref. ^29^

### In vivo experiments

Western blots were performed on protein extracts of tumours from immunocompromised mice injected with HCT116 cells and treated with a miR-27a anti-sense or a scrambled control, as previously described in ref. ^26^ Briefly, 20 × 10^6^ HCT116 cells were subcutaneously transplanted into the flank of 20 female athymic nude mice (6–8-weeks-old; Charles River, Lecco, Italy). Mice were maintained according to United Kingdom Coordinating Committee on Cancer Research (UKCCCR) guidelines and tumour volumes monitored twice a week by calliper measurement. Two weeks later, when tumours reached the volume of 200 mm^3^, mice were grouped (*N* = 5/group) and intratumorally injected every 7 days for four times with anti–miR-27a (4 ng/mm^3^) or with a scramble RNA as control (indicated as miR-27a_AS and Sc_CTRL, respectively). At day 36, the animals were subjected to gaseous anaesthesia (2–3% isoflurane and 1 lt/min O_2_) and sacrificed by cervical dislocation; the tumour masses were evaluated and excised for further analysis; xenograft RNAs were subject to qRT-PCR to establish the efficiency of miR-27a inhibition. This experiment was carried out in duplicate. No adverse or toxic effects were observed. Animal experiments were reviewed and approved by the Ethics Commission at Menarini Ricerche, according to the guidelines of the European Directive (2010/63/UE).

### 5-FluoroUracil and oxaliplatin treatment and apoptosis evaluation

HCT116 miR-27a_KD, SW480 miR-27a_KD cells and HT29 miR-27a_OE cells and relative controls were treated with 5-Fluorouracil [50 μg/ml] or Oxaliplatin [5 μg/ml] and cytotoxicity assessed after 72 h by the Prestoblue assay (Thermo Fisher) following the manufacturer’s instructions. Efficacy of drug treatments was calculated by the Growth Rate 50 (GR_50_), as described in ref. ^30^ Treated cells were evaluated for apoptosis by flow cytometry using the PE-Annexin V Apoptosis Detection I-kit, following the manufacturer’s instructions (559763, BD Biosciences, Franklin Lakes, NJ, USA). The results were analysed with the FACSuite Software v.1.0.5.3841 (BD Biosciences).

### TCGA-COAD in silico analysis

#### Database

We downloaded clinical info, mRNA and miRNA sequencing data of 459 patients affected by colon adenocarcinoma available in the TCGA-Data portal (https://portal.gdc.cancer.gov/). To correlate miRNA and gene expression, we selected 283 out of 459 patients for whom both mRNA and miRNA sequencing data were available. The analysis of correlation of treatment success with miR-27a expression was performed in a subset of patients where follow-up of treatment success (*n* = 126) and information on the chemotherapy regimen (*n* = 57, 5-Fluorouracil (5FU), Oxaliplatin (OXA) or a combination) was available.

Protein expression analysis of TCGA-COAD cohort was performed by downloading level 4 data from the TCPA portal that contains a total of 358 colon adenocarcinoma (COAD) samples and 223 proteins (44 metabolic proteins) analysed by reverse-protein-phase-array (RPPA).

#### miRNA expression analysis

miRNA isoform and mature raw counts for hsa-miR-27a from 457 tumour samples and 8 normal samples were downloaded from TCGA-Data portal (https://portal.gdc.cancer.gov/). RPM (reads per million) for the isoform raw counts were calculated with a custom perl script. From this analysis, we identified miR-27a-3p as the predominant mature form in CRC, henceforth named miR-27a (Supplementary Fig. 1A),

#### miR-27a-3p target prediction analysis

To predict putative targets of miR-27a-3p we used the mirWalk 2,0 tool that takes into account all possible binding sites (in the 5′UTR, coding sequences and 3′UTR of cognate mRNAs), estimates the binding affinity and compares the results with other predictive tools.^31^

#### GSEA and IPA analysis

Gene Set Enrichment Analysis (GSEA) was performed by using the entire collection of CGP gene-sets (MSIGDB2 - C2) for a total of 3272 gene-sets and Signal2Noise metric, 1000 random permutation of gene-sets to compute FDR, and median to centre gene expression values. Leading edge analysis was performed in GSEA software environment using default setting. IPA (Ingenuity Systems) was performed for analysis of enriched pathways (Benjamini-Hochberg correction was applied to score significant pathways).

#### Metabolic pathways activity prediction

We calculated activity/inhibition scores of selected cancer metabolic pathways based on the expression profile of a set of genes representing key metabolic proteins (identified by searching the MSigDB, KEGG and ccmGDB databases; see Supplementary Table 2A). The scores (s) represent the sum of expression values of genes coding for proteins that activate the pathway (A, activator; B, inducer) minus the sum of expression values of genes coding for proteins that inhibit the pathway (I):

$$s = {\sum} {(A,B)} - {\sum} {(I)}$$

### Clinical samples

Tumour biopsies were collected from patients before chemoradiotherapy, according to a standard protocol approved by the local ethics committee (University of Padua) and upon signing an informed consent. Five μm frozen sections from all biopsies were haematoxylin-eosin stained and only specimens with ≥ 60% malignant cells selected. A series of 62 patients with locally advanced CRCs (all Caucasian; M/F = 42/20; age 64.3 ± 9.2 years; cTNM Stage II/III = 5/57) subjected to preoperative chemoradiotherapy followed by surgery was considered. The treatment conditions were as described in refs. ^32,33^ All patients received a total dose of at least 45 Gy to the whole pelvis at 1.8 Gy daily, five times per week (median 50.5 Gy; range 45–58). Patients were defined responders (*n* = 30, Stage II = 3, Stage III = 27) or non-responders (*n* = 32, Stage II = 2, Stage III = 30) according to RECIST criteria histological tumour response to preoperative chemoradiotherapy, as assessed on the surgical specimen. Tumour response was histologically assessed according to Mandard et al.^34^ and patients were subdivided into responders (R, TRG 1–2) and non-responders (NR, TRG 3–5). Disease free survival was defined as the interval time between the date of surgery and the date of clinically evidence (histological and/or radiological) local recurrence during the follow-up.

### Statistics

All statistical analyses were made using Statistical Package from Social Science (SPSS; version 16.0) for Windows (SPSS Inc., Chicago, IL, USA), GraphPad Prism 6 (GraphPad Software Inc), and JMP 13 (SAS). Data are reported as mean ± SEM of experiments performed in triplicate; the type of statistical test used in the various analyses is indicated in the relative figure legend. Statistical significance was considered when *p* ≤ 0.05.

## Results

### In silico analysis reveals alterations of mitochondrial activity, oxidative phosphorylation and mTOR signalling in miR-27a overexpressing CRCs

**Fig. 1:**
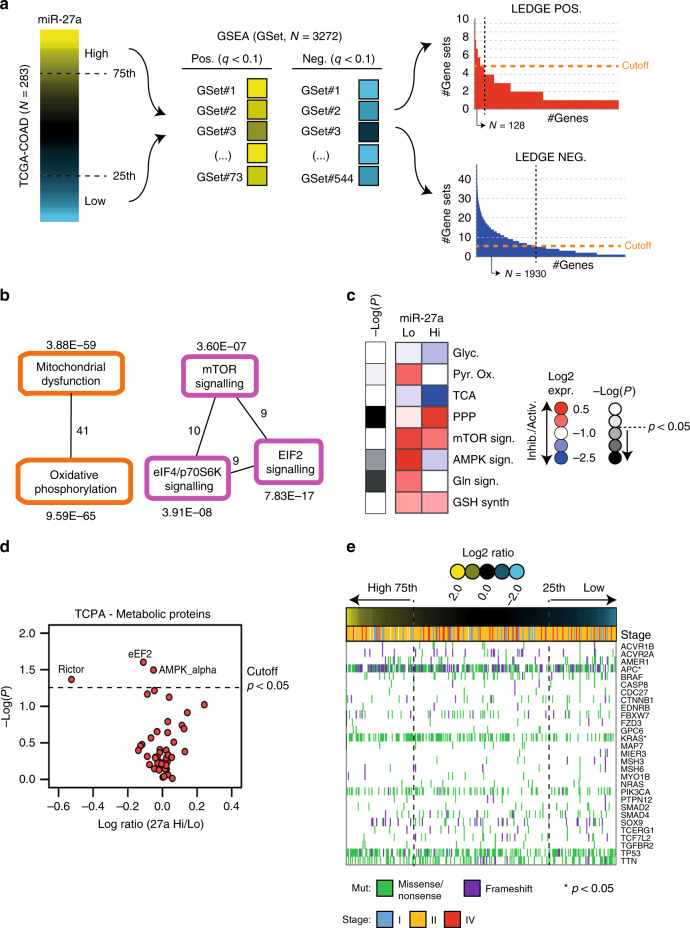
In silico analysis of miR-27a expression in the TCGA-COAD dataset. **a** Overview of the strategy used to identify dysregulated molecular mechanisms in high- or low-miR-27a expressing tumours. On the left, ranked CRC samples based on miR-27a expression; the 75th and 25th percentile rank of expression values were used to categorise high- or low-miR-27a expressing CRCs, respectively. In the middle, GSEA analysis of a total of 3,272 gene-sets (see methods) to select those significantly enriched in high-miR-27a (*q* value < 0.1; Benjiamini-Hochberg multiple test correction) or low-miR-27a CRCs (*q* value < 0.1). On the right, leading-edge analysis to identify overlapping core-genes in the various gene-sets found enriched in high/low miR-27a CRCs; genes overlapping in at least five gene-sets were considered. **b** Ingenuity pathway analysis (IPA) of canonical pathways overrepresented in high-miR-27a CRCs. Overlapping pathways are shown together with significance of overrepresentation (*q* value; Benjiamini-Hochberg multiple test correction) and number of molecules overlapping in different pathways (numbers close to lines). **c** Heatmap of median of scores (see methods) in high-miR-27a (*N* = 71) and low-miR-27a (*N* = 71) CRCs of metabolic pathways as *per* the legend, i.e.: Glyc glycolysis; Pyr. Ox. pyruvate oxidation; TCA tricarboxylic acid cycle; PPP pentose phosphate pathway; mTOR sign. mTOR signalling; AMPK sign. AMPK signalling; Gln lysis glutaminolysis; GSH synth. glutathione synthesis. Colour codes in the heatmap indicate: predicted pathway activation (Activ., red), predicted pathway inhibition (Inhib., blue), significance of scores (−Log *p* value; calculated by the Mann–Whitney *U* test) difference in high- vs. low-miR-27a CRCs (grey scale). **d** Scatterplot shows the distribution of log_2_ transformed TCPA metabolic proteins abundance ratio from high/low miR-27a. The *p* value is calculated by the Mann–Whitney *U* test. **e** Mutational analysis of 30 recurrently mutated genes with available information in TCGA-COAD cohort (224 patients). Columns represent patients and are ordered according to miR-27a expression level (upper coloured bar). Rows represent the mutational profile of each of the 29 genes across patients. The type of mutation for each gene is colour-coded as per the legend (see below the diagram). Tumour stages are indicated in the upper part of the diagram by coloured bars. Asterisks indicate significantly mutated genes upon comparing high- vs low-miR-27a CRCs (LR test).

In line with our previous findings that miR-27a is overexpressed in a cohort of CRC patients and in cell lines,^26,27^ we confirmed miR-27a upregulation in tumour vs. normal samples of the Cancer Genome Atlas (TCGA)-COAD dataset (*p* < 0.0001; Supplementary Fig. 1B). We also correlated miR-27a levels with patients’ clinical-pathological characteristics (Table 1). To predict pathways influenced by miR-27a, we ordered CRC samples according to miR-27a expression and selected high-expressing miR-27a (i.e. with miR-27a expression > 75th percentile distribution) or low-expressing miR-27a CRCs (i.e. with miR-27a expression < 25th percentile distribution) (Fig. 1a).

**Table 1:** Clinical-pathological features and radiochemotherapy response of TCGA and our patients’ series.

	TCGA-COAD samples (*n* = 283)				Subset of TCGA-COAD samples with follow-up treatment success (*n* = 126)				Our whole series (*n* = 62)			
	Total	High miR-27a	Low miR-27a	*p*-value	Total	High miR-27a	Low miR-27a	*p*-value	Total	High miR-27a	Low miR-27a	*p*-value
*N*	283	71	71	*–*	126	25	37	*–*	62	31	31	*–*
Age (years)												
Mean	66	69	65		64	68	63		64	68	64	
Min–max	31–90	31–90	35–90	0.03^a^		41–85	40–88	0.1^a^	43–79	50–79	43–78	0.18^a^
Gender												
F	156	22 (14%)	37 (24%)		51	5 (10%)	17 (33%)		20	7 (11%)	13 (21%)	
M	127	49 (39%)	34 (27%)	0.01^b^	75	20 (27%)	20 (27%)	0.03^b^	42	24 (39%)	18 (29%)	0.10^b^
Stage												
I	47	12 (26%)	11 (23%)		24	4 (17%)	4 (17%)		–	–	–	
II	113	28 (25%)	31 (27%)		50	7 (14%)	20 (40%)		5	3 (10%)	2 (4%)	
III	94	25 (27%)	19 (20%)		42	9 (21%)	11 (26%)		57	28 (90%)	29 (94%)	
IV	29	6 (21%)	10 (34%)	0.34^b^	10	5 (50%)	2 (20%)	0.19^b^	–	–	–	0.99^b^
Histological type												
Adeno	243	61 (25%)	62 (26%)		110	25 (23%)	33 (30%)		57	29 (47%)	28 (45%)	
Mucinous Adeno	37	7 (19%)	9 (24%)		16	0	4 (25%)		5	2 (3%)	3 (5%)	
NA	3	3 (100%)	0 (0%)	0.66^b^	–	–	–	0.04^b^	–	–	–	0.64^b^
Radiotherapy												
Yes	4	1 (25%)	1 (25%)		2	1 (50%)	0 (0%)		62	31 (50%)	31 (50%)	
No	228	48 (21%)	64 (28%)		123	24 (20%)	37 (30%)		–	–	–	
NA	33	19 (58%)	3 (9%)	0.84^b^	1	0 (0%)	0 (0%)	0.17^b^	–	–	–	1.00^b^
Follow-up												
Deaths within 5 years	57	18 (32%)	11 (19%)	–	24	7 (24%)	5 (20%)	–	na	–	–	–

^a^Comparison of distributions between high-miR-27a and low-miR-27a sets. Wilcoxon test.

^b^Comparison of distributions between high-miR-27a and low-miR-27a sets. Likelihood Ratio Chi-square test.

Next, we performed Gene Set Enrichment Analysis (GSEA) using ~3300 curated gene-sets (C2-CGP: chemical and genetic perturbation) virtually representing all known molecular mechanisms (Fig. 1a; see methods). GSEA detects over-representation of gene-sets in a ranked list of genes obtained by comparing, in our case, the transcriptional profile of high- vs low-miR-27a expressing tumours. We therefore identified 73 gene-sets significantly enriched (False Discovery Rate, FDR < 10%) in high- miR-27a and 544 (FDR < 10%) in low-miR-27a tumours (Fig. 1a and Supplementary Table 2B–C). Gene-sets represent a combination of interacting biological processes, not all members of which contribute to the final enrichment score (ES). In addition, a fraction of enriched gene-sets might participate in the same biological pathways, while others in separate, distinct processes. Therefore, to refine our analysis, we performed Leading-Edge analysis (LEDGE), available in GSEA software package, which selects only core members in enriched gene-sets that largely contribute to ES, and searches for overlapping genes (Fig. 1a). By doing so, we derived a list of 128 overlapping genes (≥5 gene-sets) in high-miR-27a (i.e. the “high-miR27a set”) and 1930 overlapping genes (≥5 gene-sets) in low-miR-27a CRCs (i.e. the “low-miR27a set”) (Fig. 1a; Supplementary Table 2D–E). Next, we investigated these two LEDGE derived gene sets using ingenuity pathway analysis (IPA) to dissect the pathways influenced by miR-27a expression modulation. Strikingly, in ‘the high-miR-27a set’ we found that 63 of the 128 (49%) genes matched pathways involved in mitochondrial dysfunction, oxidative phosphorylation and mTOR signalling network (Fig. 1b, Supplementary Fig. 1C, Supplementary Table 2F). Next, we predicted the regulation of cancer metabolic pathways by looking at the expression profile of manually curated gene sets coding for mostly relevant metabolic proteins (Supplementary Table 2A; see methods). In high-miR-27a vs. low-miR-27a CRCs, we confirmed a significant (*p* < 0.05) differential modulation of pathways including: (i) AMPK; (ii) enzymes involved in glutaminolysis; and iii) pentose phosphate pathway (PPP) (Fig. 1c, Supplementary Table 2A). These results suggest a role for miR-27a as a regulator of cancer metabolism. Conversely, the 1930 genes in the low-miR-27a LEDGE-gene signature matched pathways spanning many biological functions (Fig. 1b, Supplementary Fig. 1D, Supplementary Table 2G). Finally, we analysed the protein expression profile (TCPA) in a subset of tumours belonging to the TCGA-COAD cohort (see methods) from which protein data were available and confirmed that metabolic enzymes were indeed significantly regulated in miR-27a high-expressing CRC patients (Fig. 1d). We also queried the mutational profile of TCGA-COAD and found that in high-miR-27a CRC samples there was a significant higher frequency of mutations of canonical cancer drivers in CRC, i.e. *APC* and *K-RAS* (*p* < 0.05; likelihood ratio test) (Fig. 1e, Supplementary Fig. 1E).

### miR-27a influences CRC mitochondrial activity

Having shown that miR-27a modulates pathways involved in mitochondrial dysfunction, oxidative phosphorylation and mTOR signalling network as from our in silico analysis (Fig. 1b), we investigated the impact of miR-27a expression variations on metabolism in a panel of CRC cell lines previously assessed for miR-27a levels.^26^ To this goal, the high-miR-27a expressing HCT116 and SW480 cells (Supplementary Fig. 2A) were transduced with viral vectors carrying a short hairpin anti-sense RNA to generate pools of clones, henceforward named miR-27a_KD cells. Contrariwise, the low-miR-27a expressing HT29 cells (Supplementary Fig. 2A) were transduced with an expression vector for a mimic RNA to generate pools of clones, henceforward named miR-27a_OE cells. Vectors carrying scrambled sequences were transduced to produce stable control clones, named HCT116 and SW480 CTRL_KD and HT29 CTRL_OE, respectively. The miR-27a expression profile was verified by qRT-PCR analysis (Supplementary Fig. 2A) and the efficacy of silencing by assessing *FBXW7* and *PPARG*, two known miR-27a targets (Supplementary Fig. 2B). HCT116 miR-27a_OE KD and HT29 miR-27a_OE clones and their relative controls were evaluated for proliferation, migration and invasion ability. The data indicate that miR-27a stimulates growth and spreading consistent with our previous results^26,27^ (Supplementary Fig. 2C–E).

**Fig. 2:**
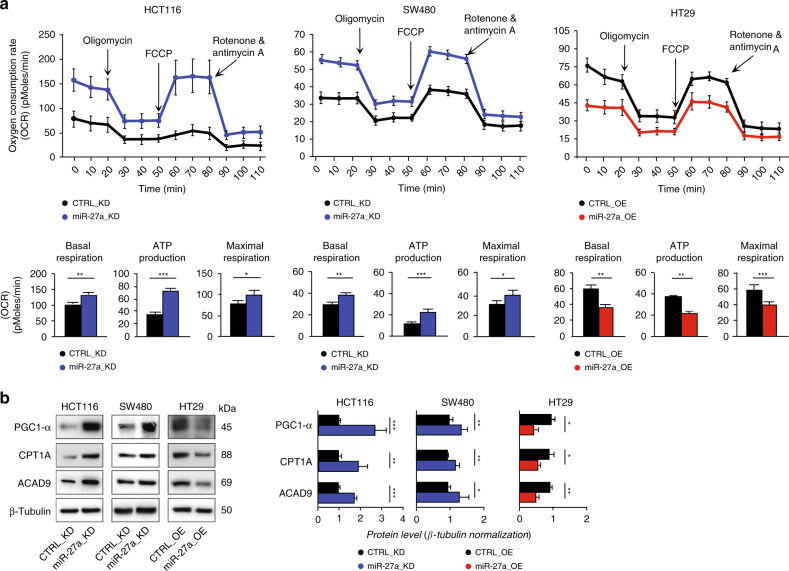
miR-27a affects overall mitochondrial activities. **a** Kinetic profile of the Oxygen Consuming Rate (OCR) assay in HCT116 and SW480 miR-27a_KD and HT29 miR-27a_OE cells and their relative CTRL_KD and CTRL_OE cells, respectively. OCR, expressed as picomoles of oxygen/minute, was measured in real time under basal conditions and in response to the indicated compounds: Oligomycin, FCCP, Rotenone and Antimycin A. Data are reported as mean ± SEM of experiments performed in triplicate; the means values were compared by one-way analysis of variance test (ANOVA). Statistical significance was considered when *p ≤ 0.05, **p ≤ 0.01, ***p < 0.001. The histograms show the values of basal respiration, ATP production and maximal respiration as by the calculations of the experimental OCR data obtained from the various pairs of cell lines analysed. **b** Immunoblot analysis of PGC-1α, CPT1A and ACAD9 in the same cells as in **a**. β-Tubulin was used for protein loading normalisation. Data are reported as mean ± SEM of experiments performed in triplicate; the means values were compared by one-way analysis of variance test (ANOVA). Statistical significance was considered when *p ≤ 0.05, **p ≤ 0.01, ***p < 0.001.

We first evaluated the influence of miR-27a expression variations on mitochondrial functions by assessing the oxidative phosphorylation with the OCR assay. In particular, HCT116 miR-27a_KD cells displayed a higher value of both basal and maximal respiration, with a good production of ATP and a moderate respiratory capacity with respect to the corresponding CTRL_KD (Fig. 2a). In the SW480 miR-27a_KD cells we observed a very similar OCR profile, with lower basal levels but overlapping differences (Fig. 2a). On the contrary, in HT29 miR-27a_OE cells we found a basal and maximal respiration/ATP production lower than the corresponding CTRL_OE (Fig. 2a). These results were supported by those obtained surveying bioinformatics tools to search for predicted targets of miR-27a (Supplementary Table 3). From this analysis, we identified several components of Complex 1 and Complex 5 of the electron transport chain and PPAR gamma co-activator-1α (PGC-1α), a central regulator of mitochondrial activity and biogenesis.^35^ We thus checked PGC-1α expression and found that it increased in HCT116 and SW480 miR-27a_KD cells (Fig. 2b), while it diminished in HT29 miR-27a_OE cells with respect to their controls. PGC-1α directly regulates PPARγ which, in turn, modulates fatty acids mitochondrial import and degradation.^35^ In line, we analysed carnitine palmitoyl-transferase 1A (CPT1A), the acyl-CoA transporter into mitochondria, and Acyl-CoA dehydrogenase family member 9 (ACAD9), the first enzyme of fatty acid β-oxidation, which are also predicted targets of miR-27a (Supplementary Table 3). The levels of both proteins increased in HCT116- and SW480- miR-27a_KD cells (Fig. 2b) while decreased in the HT29 miR-27a_OE cells with respect to their relative controls.

Consistent with these results, we observed in miR-27a_KD cells: (i) an increase of the citrate synthase activity i.e. the tricarboxylic acids (TCA) cycle gatekeeper enzyme (*p* < 0.01); (ii) an increase of the intracellular ATP levels (*p* < 0.01), in line with our previous report;^27^ (iii) an increase of the production of reactive-oxygen species (ROS) that paralleled the augmented mitochondrial respiration (Supplementary Fig. 3A–C).

All together these results support that miR-27a modulates the overall mitochondrial metabolism either directly, via PGC1α, and, indirectly, via PPARγ we previously validated as a direct target.^26^

### miR-27a modulates CRC metabolic potential

**Fig. 3:**
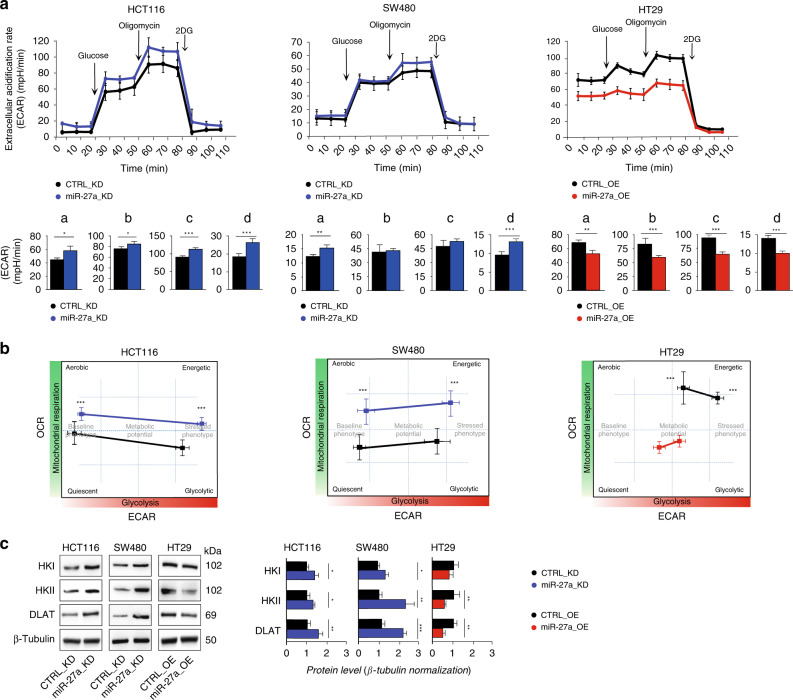
miR-27a controls the destiny of glucose in CRC cells. **a** Kinetic profile of extracellular acidification rate (ECAR) in HCT116 and SW480 miR-27a_KD and HT29 miR-27a_OE cells and their relative CTRL_KD and CTRL_OE cells, respectively. ECAR, expressed as mpH/min, was measured in real time, under basal conditions and in response to glucose, oligomycin and 2DG. Data are reported as mean ± SEM of experiments performed in triplicate; the means values were compared by one-way analysis of variance test (ANOVA). Statistical significance was considered when *p ≤ 0.05, **p ≤ 0.01, ***p < 0.001. The histograms show the quantification of ECAR kinetic profile in: (a) basal glycolysis, (b) basal glycolysis post-glucose injection, (c) glycolytic capacity, (d) glycolytic reserve (d = c − b). **b** “Cell energy phenotype profile”. The drawings illustrate the metabolic potential of the same cells as in **a** as a measure of cells’ ability to respond to an energy request after a stress via mitochondrial respiration and glycolysis. The baseline phenotype (levels of mitochondrial respiration and glycolysis in basal conditions) is reported on the left of each drawing; the stressed phenotype (relative utilisation of aerobic respiration and glycolysis upon stressed conditions) is on the right. Data are reported as mean ± SEM of experiments performed in triplicate; the means values were compared by one-way analysis of variance test (ANOVA). Statistical significance was considered when ***p < 0.001. **c** Immunoblot analysis of glycolytic enzymes levels in the same cell lines as in **a**. β-Tubulin was used for protein loading normalisation. Data are reported as mean ± SEM of experiments performed in triplicate; the means values were compared by one-way analysis of variance test (ANOVA). Statistical significance was considered when *p ≤ 0.05, **p ≤ 0.01, ***p < 0.001.

Dysregulation of oxidative metabolism in cancer is frequently associated with aerobic glycolysis (Warburg effect). We verified the impact of variations of miR-27a expression on glycolysis in our cell model system by performing the ECAR assay, which measures the extracellular acidification rate. miR-27a silencing augmented the proficiency of glycolysis in HCT116 miR-27a_KD cells while in SW480 miR-27a_KD the effect was borderline (Fig. 3a). On the contrary, HT29 miR-27a_OE cells displayed a lower basal glycolysis and glycolytic reserve (Fig. 3a, right panel). We also correlated the OCR to the ECAR values obtained (Fig. 3b; see methods) and, interestingly, the low miR-27a expressing cells were more aerobic and energetic than the high-miR-27a expressing ones that were also less responsive to energetic request than the relative controls (Fig. 3b).

We corroborated these results by analysing the expression of a set of glycolytic enzymes that catalyse unidirectional reactions. In particular, we analysed Hexokinase 1 and 2 (HK1 and HK2), the first enzymes of the pathway, and the DihydroLipoamide S-AcetylTransferase (DLAT), the E2 component of the pyruvate dehydrogenase complex (PDH) which converts pyruvate into acetyl-CoA, as they were predicted targets of miR-27a in our in silico survey (Supplementary Table 3). HK1 and HK2 increased their expression in both HCT116- and SW480 miR-27a_KD cells compared to their CTRL_KD; HK1-2 were, instead, lower in HT29 miR-27a_OE than their CTRL_OE (Fig. 3c). Similarly, DLAT increased in both HCT116 and SW480 miR-27a_KD cells while decreased in HT29 miR-27a_OE cells with respect to controls. Glucokinase (GCK), Aldolase (ALDO) and Enolase (ENO) showed no variations in HCT116 derived cells (Supplementary Fig. 3D). In addition, in the same HCT116 miR-27a_KD cells, we observed an increase of the tetrameric and reduced form of Pyruvate Kinase Muscle Isoform 2 (PKM2) and a lower flux of pentose phosphate pathway (PPP) with respect to the CTRL_KD (Supplementary Fig. 3E, F).

Finally, we evaluated some of the metabolic enzymes in a xenograft mouse model. HCT116 cells were subcutaneously implanted and, when formed, the tumour mass injected with a miR-27a inhibitor or a scrambled control as we previously reported.^26^ HK1, HK2, DLAT, CPT1A and ACAD9, representative of the overall mitochondrial activity, displayed higher expression in tumours injected with a miR-27a anti-sense than those injected with a scrambled control (Supplementary Fig. 3G). All together these results indicate that miR-27a plays a key role in modulating the overall cell metabolism.

### miR-27a cross talks with other genes and pathways involved in tumorigenesis

**Fig. 4:**
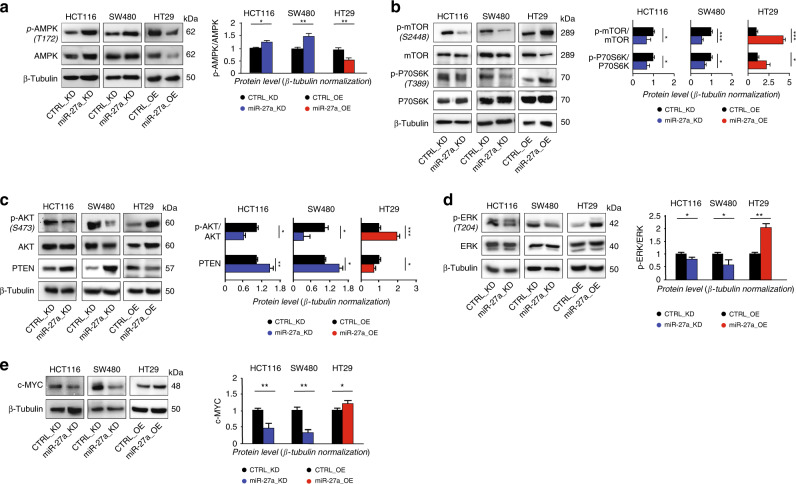
miR-27a finely orchestrates different cell signalling. Total protein extracts from HCT116 and SW480 miR-27a_KD and HT29 miR-27a_OE cells and their relative CTRL_KD and CTRL_OE were analysed by Western blot for: **a** AMPK and its phosphorylation level**; b** mTOR pathway activation; **c** pAKT/AKT and PTEN; **d** pERK/ERK; **e** cMYC. The density value of each band was referred to β-Tubulin, used for protein loading normalisation, and expressed as fold-change relative to control. Data are reported as mean ± SEM of experiments performed in triplicate; the means values were compared by one-way analysis of variance test (ANOVA). Statistical significance was considered when *p ≤ 0.05, **p ≤ 0.01, ***p < 0.001.

AMP-activated protein kinase (AMPK), the main sensor of the cellular energetic status and regulator of the entire metabolism in eukaryotic cells,^36^ was predicted to be reduced in high-miR-27a expressing CRCs from our in silico analysis (Fig. 1c, d). AMPK was also identified as a target by analysing miR-27a predictive tools (Supplementary Table 3) and in a different cell context.^37^ We found that its expression increased in HCT116 and SW480 miR-27a_KD cells, as was its phosphorylation (p-AMPK Thr172), while both decreased in HT29 miR-27a_OE cells relative to controls (Fig. 4a). Notably, AMPK modulates TSC1/2 and the downstream factor RHEB, a repressor of mTOR complex 1.^38,39^ Therefore, we hypothesised that miR-27a overexpression by downmodulating AMPK would activate the mTOR pathway and the associated protein synthesis, ultimately sustaining tumour biomass production in the context of aerobic glycolysis. In keeping with this hypothesis, we found that mTOR complex 1 phosphorylation increased in HT29 miR-27a_OE while decreased in HCT116 and SW480 miR-27a_KD cells (Fig. 4b). Likewise, we found that phosphorylation of Thr389 of the p70-S6K kinase diminished in miR-27a_KD cells while augmented in miR-27a_OE cells compared to their relative controls (Fig. 4b). Consistent with these results, the p-AMPK/AMPK ratio increased while the p-mTOR/mTOR ratio slightly diminished in extracts from xenografts of HCT116 cells injected with a miR-27a anti-sense with respect to their basal levels (Supplementary Fig. 3H).

In cancer cells, mTOR can be regulated by the mitogen-responsive signalling, such as PI3K/AKT which contributes to tumorigenesis and chemoresistance.^14,39,40^ Interestingly, we found that AKT phosphorylation was reduced in HCT116 and SW480 miR-27a_KD cells while increased in HT29 miR-27_OE (Fig. 4c). Conversely, the expression of PTEN, an inhibitor of PIK3/AKT signalling, increased in miR-27a_KD cells while diminished in miR-27a_OE cells with respect to their controls, consistent with PTEN being a direct target of miR-27a^40^ (Fig. 4c). mTOR can be regulated also in an AKT-independent manner via the RAS/MEK/ERK pathway.^41^ Notably, and in line, we found that ERK phosphorylation was reduced in HCT116 and SW480 miR-27a_KD cells while increased in HT29 miR-27a_OE cells, relative to controls likely through inhibition of Sprouty2, a miR-27a direct target^42^ (Fig. 4d). Furthermore, miR-27a correlates also with *cMYC*, an oncogene known to influence metabolic reprogramming and chemoresistance.^43^ In accordance, its expression was high in miR-27a_OE cells while was low in miR-27a_KD cells with respect to their controls (Fig. 4e). This result can be explained by the fact that miR-27a directly targets FBXW7, an E3 ubiquitin protein ligase, which, in turn, targets c-MYC,^44,45^ supporting a positive regulatory loop (Supplementary Fig. 2B). Of note, FBXW7 targets also mTOR accounting for the inverse relationship already reported^46^ and for the existence of the miR-27a-FBXW7-mTOR axis documented here.

Finally, miR-27a positively influences the Wnt/β−catenin pathway that is pivotal in colorectal tumorigenesis, via PPARγ, RXRα and SRFP1.^47–49^ We have shown and confirmed here that PPARγ is a miR-27a direct target along with RXRα and SFRP1, two previously validated ones, all implicated in the control of this pathway (Supplementary Fig. 2B). Taken together, these results indicate that miR-27a orchestrates the metabolic reprogramming of tumour cells through the interplay with several oncogene-dependent and tumour-associated pathways to promote cancer progression.

### miR-27a affects the response to chemotherapy

**Fig. 5:**
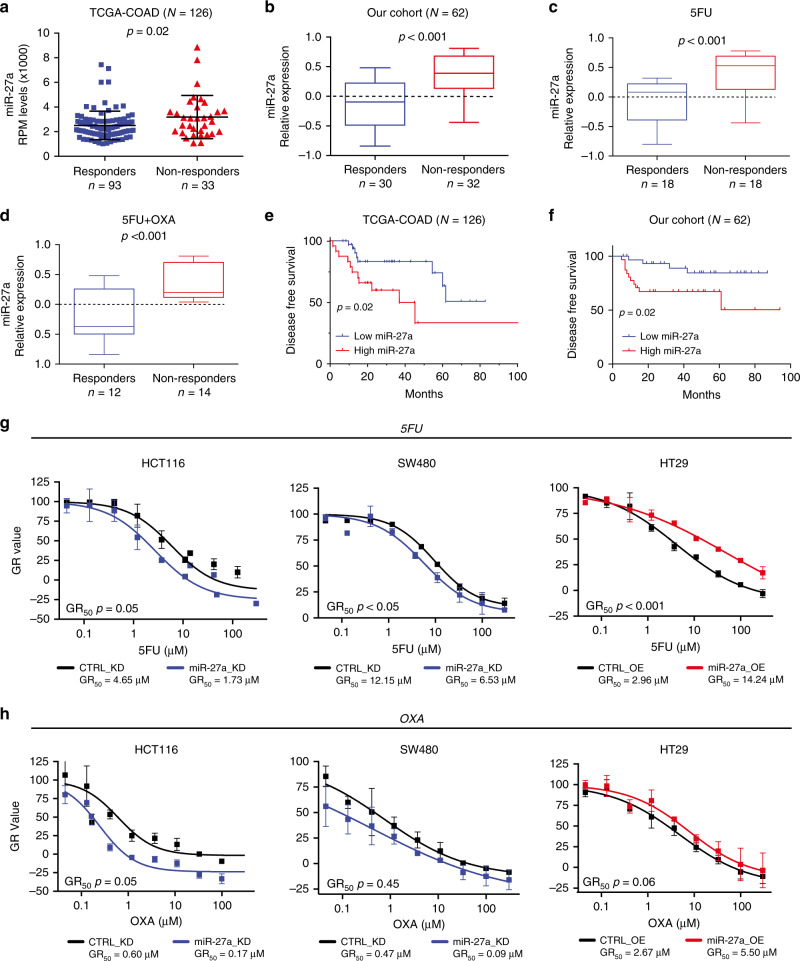
miR-27a modulates sensitivity to chemotherapeutics in CRC patients and cells in culture. **a** The scatter dot plots report miR-27a expression levels referred to the response to therapy in a subset of the TCGA-COAD cohort (*n* = 126) for which follow-up information was available. The *p* value was calculated by the Mann–Whitney *U* test. **b** The box plots show miR-27a expression levels referred to the response to chemotherapy in our series of CRC bearing-patients (*n* = 62) stratified in responders (*n* = 30) vs non-responders (*n* = 32), while the box plots in **c**, **d** show miR-27a expression levels referred to responders and non-responders to 5FU alone or to 5FU + OXA. The *p* value was calculated by the Mann–Whitney *U* test. **e** Kaplan–Meier curve of disease free survival (DFS) in the TCGA COAD cohort: patients with low miR-27a expression (indicated in blue) show a better DFS (*p* = 0.02) than those with high miR-27a expression (indicated in red). DFS was calculated with the Log-rank test. **f** Kaplan–Meier curve of disease free survival (DFS) in our cohort: patients with low miR-27a expression (indicated in blue) show a better DFS (*p* = 0.02) than those with high miR-27a expression (indicated in red). DFS was calculated with the Log-rank test. **g**, **h** The graphs illustrate the growth curves of HCT116 and SW480 miR-27a_KD as well as of HT29 miR-27a_OE and relative controls, respectively, exposed to increasing concentrations of 5FU and OXA for 72 h. The doses that inhibit cell growth by 50% are reported as GR_50_ and represent the mean ± SEM of three independent experiments carried out in triplicates; the *p* values are calculated by the two-way analysis of variance test (ANOVA).

Metabolic rewiring of cancer cells and cancer pathways activate mechanisms implicated in chemoresistance.^6,7^ We, therefore, asked whether miR-27a was able to influence the CRC treatment response. Our in silico analysis of the TCGA-COAD dataset revealed that miR-27a expression was higher in the CRCs resistant to therapy (i.e. with a stable or progressive disease (*p* = 0.02, Wilcoxon test; Fig. 5a; Table 1 and see methods).

We confirmed this correlation in our independent cohort of CRC patients (*n* = 62) subjected to surgical resection and treated with adjuvant chemoradiotherapy represented by 5FU or 5FU + OXA and equivalent amount of radiations (median 50 GY) (Table 1). miR-27a overexpression was significantly associated with resistance to 5FU and/or 5FU + OXA (*p* < 0.001; Mann–Whitney *U* test) (Fig. 5b). We then divided our patients in two subgroups according to the treatments, and, consistently, we found that miR-27a overexpression was significantly associated with drug-resistance (*p* < 0.001; Mann–Whitney *U* test) (Fig. 5c, d). Notably, patients bearing high miR-27a expressing tumours showed a significant higher risk of recurrence, reported as disease free survival (DSF), in both the TCGA-COAD and our cohort of patients (*p* = 0.02; Log-rank test) (Fig. 5e, f). We corroborated these results by treating HCT116 and SW480 miR-27a_KD and HT29 miR-27a_OE cells and their relative controls with increasing concentrations of 5FU and OXA and evaluated the half maximal growth rate (GR_50_) which is a more reliable means to evaluate a chemo-sensitive profile.^30^ HCT116 and SW480 miR-27a_KD cells displayed increased sensitivity to 5FU, while HT29 miR-27a_OE cells were more resistant with respect to their relative controls in a statistically significant manner (Fig. 5g). When we analysed the sensitivity to OXA, we observed similar trends, despite that the significance was reached only in the HCT116 miR-27a_KD cells (Fig. 5h). Lastly, miR-27a overexpression decreased the number of pre-/apoptotic cells after 5FU or OXA treatment, while in contrast miR-27a downregulation increased the percentage of pre-/apoptotic cells (Supplementary Fig. 3I). These results indicate that miR-27a influences the different response to 5FU and OXA in CRC.

## Discussion

Reprogramming cell metabolism is a hallmark of cancer, whose underlying molecular mechanisms are still undefined.^1,2^ Here we describe the first in-depth analysis of miR-27a biological functions in CRC, further to cell proliferation and immune evasion as reported.^26,27^ We employed an in silico approach, a TCGA transcriptome survey and several experimentations using CRC cells in which we modulated miR-27a expression and found that it is a key determinant with a profound impact on CRC metabolic reprogramming. Through this analysis, we identified that gene-sets whose expression alteration is linked to mitochondrial dysfunction, oxidative phosphorylation and mTOR signalling pathways are significantly enriched in high-miR-27a CRCs.

Molecular dissection of these metabolic pathways revealed that mitochondrial dysfunction involves impaired oxidative phosphorylation, low ATP levels, reduced TCA cycle and fatty acid β-oxidation through a direct or indirect modulation of several key enzymes of these processes. Since miR-27a overexpressing CRC cells proliferate more and have increased invasive and migratory potential (Supplementary Fig. 2C–E), as in diverse cancer types,^26,40,45,50^ they ought to upregulate several biosynthetic pathways (nucleotides, proteins, lipids) to favour biomass production and availability of ATP to sustain tumour growth. We show here that high-miR-27a expressing tumours (COAD) and cells manage these requirements by restricting catabolic pathways and diverting glycolytic intermediates to enhance the pentose phosphate pathway (PPP) (Fig. 1c and Supplementary Fig. 3F) towards biosynthetic pathways as well as anaplerotic cycle reactions to provide a sufficient supply of energy as ATP. Consistently, miR-27a negatively modulates AMPK and positively mTOR, two crucial sensors of the energetic status of the cell, likely pushing protein synthesis and reducing autophagy and apoptosis so to rewire metabolism to that of a more proliferating cell.

Importantly, the data presented suggest that miR-27a establishes cross-talks with activated oncogenes such as *AKT* and *K-RAS/B-RAF*^37,40,41^ via PTEN and Sprouty2, two direct targets of miR-27a,^40,42^ contributing to modulate these pathways, notwithstanding the presence of mutations of the driving oncogenes. Likewise, they indicate that miR-27a establishes a regulatory axis with *cMYC* through FBXW7^44^ and with the Wnt/β-catenin pathway through PPARγ, RXRα and SFRP1,^47–49^ all direct targets. Thus, miR-27a coordinates and modulates oncogenes-driven and tumour-associated pathways to reprogramme cell homoeostasis towards a more proliferating and aggressive phenotype.

High-miR-27a expression is associated with increased resistance to chemotherapy as shown here in two independent cohorts of CRC patients (TCGA-COAD and our series) and confirmed in our CRC cell model system. Chemoresistance is achieved by diverse means, such as through membrane proteins that modulate absorption and excretion of many compounds to maintain their concentration at non-toxic levels. Some of these proteins are, indeed, targets of cMYC^51^ and miR-27a.^52^ Resistance can also be elicited by interfering with various steps in nucleic acid metabolism through inhibition/activation of key target enzymes.^53^ Dihydropyrimidine dehydrogenase (DPYD), the rate-limiting enzyme of 5FU pharmacokinetics, is a direct target of miR-27a thus influencing the efficacy of treatment.^54^ Likewise, ERCC1 and ERCC4, enzymes that repair Oxaliplatin-induced DNA damages, are predicted targets of miR-27a leading to accumulation of damages, contributing to chemoresistance (Supplementary Table 3).^24^ Generating new epitopes from mutated and unrepaired genes is a means for an efficient adaptive immune response. However, this cannot be achieved as the exposure of neoantigens and stimulation of an efficient immune response is impaired in miR-27a overexpressing CRCs, as previously reported.^26,27^

In conclusion, using independent cohorts of CRC patients and ad hoc experimental models, we provide evidence that miR-27a acts as a key regulator of CRC metabolism which favours an aggressive phenotype and acquisition of chemoresistance. miR-27a exerts pleiotropic effects by modulating diverse and interconnected pathways, that closely resemble those obtained upon activation of known cancer drivers involved in metabolism reprogramming and the associated drug sensitivity. For this reason, it would be deemed to investigate the molecular determinants that impinge on these mechanisms and whether miR-27a or downstream-modulated proteins can be potential therapeutic targets in aggressive chemoresistant CRCs.

## Supplementary information

Supplementary Information

## Data Availability

Clinical information, mRNA and miRNA sequencing data of 459 patients affected by colon adenocarcinoma are available in the TCGA-Data portal (https://portal.gdc.cancer.gov/). Protein expression analysis of TCGA-COAD cohort was performed by downloading level 4 data from the TCPA portal. Data on our cohort’s patients are available from the corresponding author on reasonable request.
