# Correction: miR-27a is a master regulator of metabolic reprogramming and chemoresistance in colorectal cancer

**DOI:** 10.1038/s41416-020-0855-1

**Published:** 2020-04-17

**Authors:** Giovannina Barisciano, Tommaso Colangelo, Valeria Rosato, Livio Muccillo, Maria Letizia Taddei, Luigi Ippolito, Paola Chiarugi, Mario Galgani, Sara Bruzzaniti, Giuseppe Matarese, Matteo Fassan, Marco Agostini, Francesca Bergamo, Salvatore Pucciarelli, Annalucia Carbone, Gianluigi Mazzoccoli, Vittorio Colantuoni, Fabrizio Bianchi, Lina Sabatino

**Affiliations:** 1 Department of Sciences and Technologies, University of Sannio, Via Francesco de Sanctis, 82100 Benevento, Italy; 2 Fondazione IRCCS Casa Sollievo della Sofferenza, Cancer Biomarkers Unit, Viale Padre Pio, 7, 71013 San Giovanni Rotondo, FG Italy; 3 Department of Experimental Biomedical and Clinical Medicine, University of Florence, Viale Morgagni 50, 50134 Florence, Italy; 4 Department of Molecular Medicine and Medical Biotechnologies “Federico II”, University, Naples Via S. Pansini, 5, 80131 Naples, Italy; 5 Department of Biology, “Federico II” University, 80126 Naples, Italy; 6 Laboratory of Immunology, Institute of Experimental Endocrinology and Oncology (IEOS-CNR), Via S. Pansini, 5, 80131 Naples, Italy; 7 Department of Medicine (DIMED), Surgical Pathology Unit, University of Padua, Via Giustiniani, 2, 35128 Padua, Italy; 8 Department of Surgery, Oncology and Gastroenterology, First Surgical Clinic Section, University of Padua, Via Giustiniani, 2, 35128 Padua, Italy; 9 Department of Clinical and Experimental Oncology, Unit of Medical Oncology 1, Veneto Institute of Oncology IOV, IRCCS, Via Gattamelata, 64, 35128 Padua, Italy; 10 Fondazione IRCCS Casa Sollievo della Sofferenza, Division of Internal Medicine and Chronobiology Unit, Viale Padre Pio, 7, 71013 San Giovanni Rotondo, FG Italy

**Keywords:** Cancer metabolism, miRNAs

Correction to: *British Journal of Cancer* (2020); 10.1038/s41416-020-0773-2, published online 5 March 2020

Since the publication of this paper the authors noticed an error in the name of Maria Letizia Taddei, where Professor Taddei’s surname was incorrectly listed as Professor Letizia Taddei. This has been corrected above.

