# Supplementary material for: miR-27a is a master regulator of metabolic reprogramming and chemoresistance in colorectal cancer
Source: Br J Cancer. 2020 Mar 5;122(9):1354–66. doi: 10.1038/s41416-020-0773-2 (PMC7188668; doi:10.1038/s41416-020-0773-2)
Supplement: Supplementary file 1 — Supplementary Information [file 41416_2020_773_MOESM1_ESM.pdf]

miR-27a is a master regulator of metabolic reprogramming and chemoresistance in colorectal cancer

Giovannina Barisciano[1§], Tommaso Colangelo[2§], Valeria Rosato[1§], Livio Muccillo[1], Maria Letizia Taddei[3], Luigi Ippolito[3], Paola Chiarugi[3], Mario Galgani[4], Sara Bruzzaniti[5], Giuseppe Matarese[4,6], Matteo Fassan[7], Marco Agostini[8], Francesca Bergamo[9], Salvatore Pucciarelli[8], Annalucia Carbone[10], Gianluigi Mazzoccoli[10], Vittorio Colantuoni[1], Fabrizio Bianchi[2*] and Lina Sabatino[1*]

Content of Supplementary Information:

Supplementary Figure 1

Supplementary Figure 2

Supplementary Figure 3

Supplementary Table 1

Supplementary Table 2

Supplementary Table 3

Supplementary Figure 1

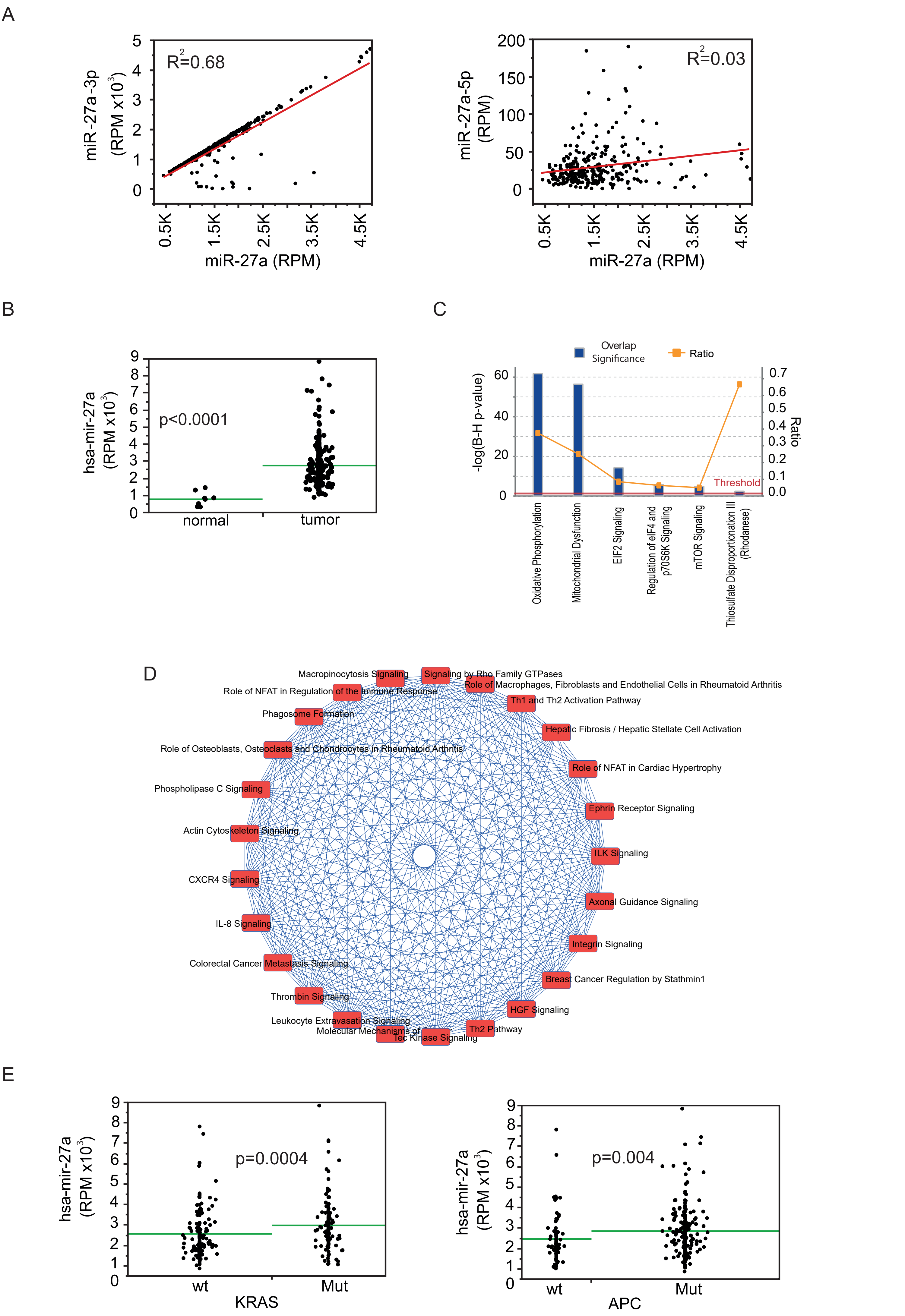

Data supporting miR-27a in silico analysis of TCGA-COAD dataset

A) Evaluation of the contribution of the two mature miR-27a-3p or miR-27a-5p forms to the total miR-27a expression levels measured in the TCGA-COAD dataset. The Reads Per Million (RPM) are reported in Y- and X-axes. R2 of linear fit is reported.

B) Expression analysis of miR-27a in CRC samples (n=283) vs. normal tissues (n=8) available in the TCGA-COAD dataset. The Reads Per Million (RPM) are reported in Y-axes. Wilcoxon test was performed to test for significance of the differential expression.

C) Overrepresentation of canonical pathways in high-miR-27a CRCs using IPA. The blue bars indicate the overlap significance (Benjiamini-Hochberg multiple test correction; primary Y-axes) in high-miR-27a CRCs related to the selected pathways (X-axes); red line indicates significance threshold (q <0.05); the yellow line indicates the ratio (secondary Y-axes) of present genes over the total constituting each pathway.

D) IPA network of overlapping canonical pathways significantly overrepresented in low-miR-27a CRCs (q<0.05; Benjiamini-Hochberg multiple test correction). The blue lines (i.e. edges) represent the connection among pathways due to overlapping genes within.

E) Expression analysis of miR-27a in CRC samples mutated in KRAS and APC vs. wt in the TCGA-COAD dataset. The Reads Per Million (RPM) are reported in Y-axes. Wilcoxon test was performed to test for significance of the differential expression.

Supplementary Figure 2

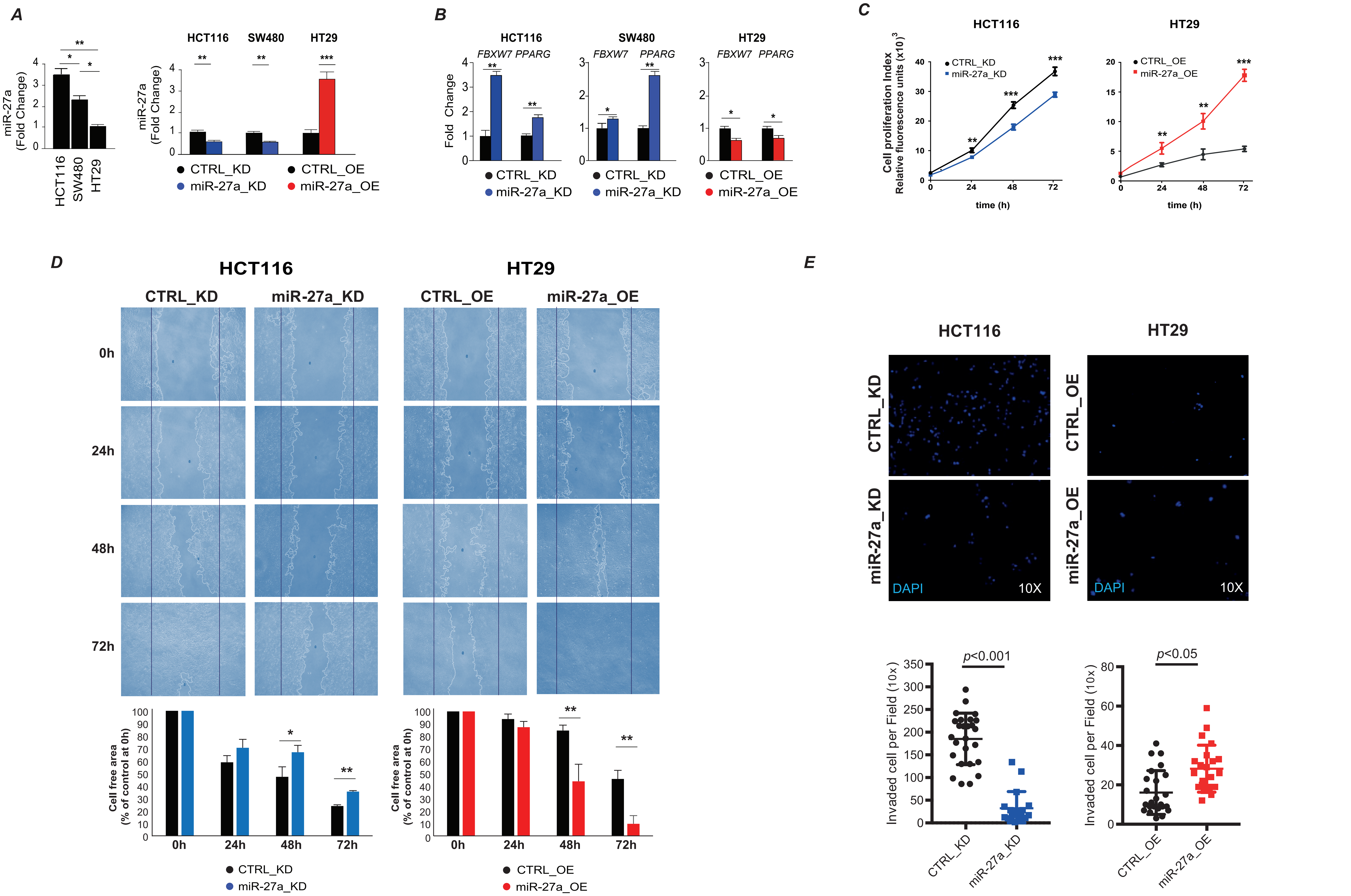

Experimental data supporting the results illustrated in the main figures.

A) miR-27a expression was quantified by qRT-PCR in HCT116, SW480 and HT29 cells (left panel), or in HCT116 and SW480 miR-27a_KD and HT29 miR-27a_OE cells and their relative controls (right panel). Normalization was carried out by assessing U6 RNA levels. Data are reported as mean ± SEM of experiments performed in triplicate; the mean values were compared by one-way analysis of variance test (ANOVA). *p ≤0.05, **p ≤0.01 ***p ≤0.001.

B) The expression profile of FBXW7 and PPARG, two known miR-27a targets, was assessed by qRT-PCR analysis in HCT116 and SW480 miR-27a_KD and HT29 miR-27a_OE cells and their relative controls. Data are reported as mean ± SEM of experiments performed in triplicate; the mean values were compared by one-way analysis of variance test (ANOVA). *p ≤0.05, **p ≤0.01.

C) Growth assessment of HCT116 miR-27a_KD, and HT29 miR-27a_OE cells and relative controls was carried out at 72hs using the PrestoBlue TM Cell Viability test. Data are reported as mean ± SEM of experiments performed in triplicate; the mean values were compared by one-way analysis of variance test (ANOVA). *p ≤0.05,**p ≤0.01. ***p ≤0.001.

D) Wound healing assay of the same cells as in C) was performed at 24, 48 and 72 hs after scratches. Cells migration was inversely evaluated as percentage of cell-free area at the indicated time-points compared with the area at the start of the experiments. Blue lines indicate the positions of the initial scratches. Data are reported as mean ± SEM of experiments performed in triplicate; the mean values were compared by one-way analysis of variance test (ANOVA). *p ≤0.05, **p ≤0.01.

E) Invasion ability of same cells as in C was evaluated by the Transwell assay. The scatter dot plots show the numbers of invading cells (twenty fields analyzed; magnitude 10X) in triplicate experiments, counted by DAPI nuclear fluorescence. Data are reported as mean ± SEM of experiments performed in triplicate; the mean values are compared by one-way analysis of variance test (ANOVA). *p ≤0.05, ***p ≤0.001.

Supplementary Figure 3

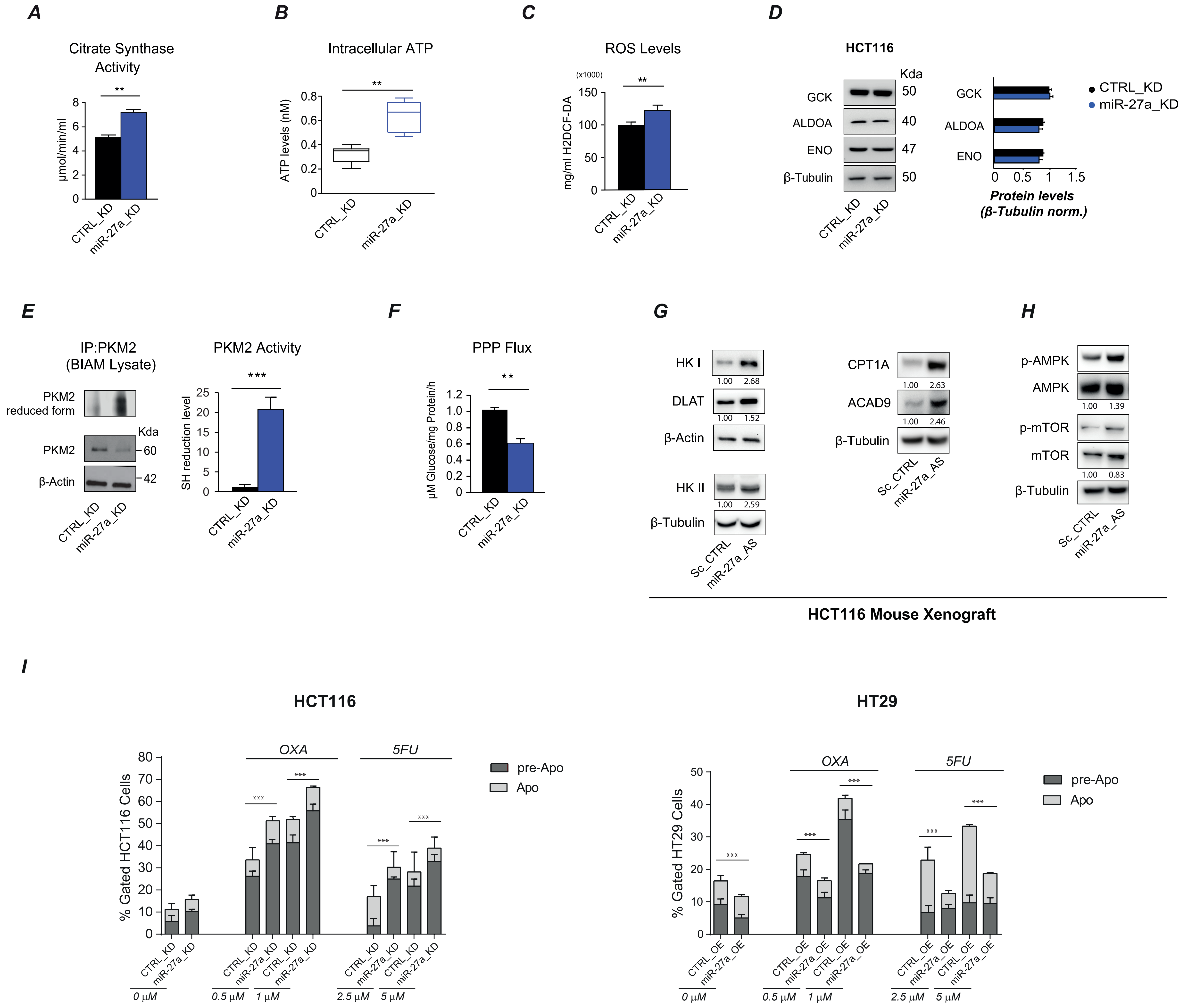

Experimental data supporting the results illustrated in the main figures

A) Mitochondrial citrate synthase activity was fluorimetrically determined in HCT116 miR-27a_KD and CTRL_KD cells as described in Materials and Methods. Data are reported as mean ± SEM of experiments performed in triplicate; the mean values were compared by one-way analysis of variance test (ANOVA). **p ≤0.01

B) Intracellular ATP levels were assayed in the same cells as in C), as previously published26. Data are reported as mean ± SEM of experiments performed in triplicate; the mean values were compared by one-way analysis of variance test (ANOVA). **p ≤0.01.

C) Intracellular ROS levels were determined in the same cells as in C) with the H2DCF-DA fluorimetric assay and normalized to protein content. Data are reported as mean ± SEM of experiments performed in triplicate; the mean values were compared by one-way analysis of variance test (ANOVA) **p ≤0.01.

D) Glucokinase (GCK), Aldolase (ALDO) and Enolase (ENO) immunoblot showed no variations in HCT116 derived cells. Data are reported as mean ± SEM of experiments performed in triplicate; the mean values were compared by one-way analysis of variance test (ANOVA)

E) PKM2 BIAM-labeled immunocomplexes and immunoblot of PKM2 in HCT116 derived cells. Data are reported as mean ± SEM of experiments performed in triplicate; the mean values were compared by one-way analysis of variance test (ANOVA) ***p ≤0.001

F) Measurement of the PPP flux in HCT116 miR-27a_KD compared to CTRL cells. Data are reported as mean ± SEM of experiments performed in triplicate; the mean values were compared by one-way analysis of variance test (ANOVA). **p ≤0.01.

G, H) Immunoblot analysis of HKI, HKII, DLAT, CPT1A, ACAD9, p-AMPK/AMPK and p-mTOR/mTOR on tumor extracts obtained from HCT116 cells xenografted in immunocompromised mice.β-Tubulin and β-Actin were used for protein loading normalization. The fold change is reported below the corresponding bands and referred to Sc_CTRL= Scrambled Control and miR-27a_AS= miR-27a Antisense.

I) Flow cytometry analysis of pre-apoptotic (annexin V-PE+ and 7-AAD-) and apoptotic (annexin V-PE+ and 7-AAD+) in HCT116 miR-27a_KD and HT29 miR-27a_OE cells, with respect to their relative controls, treated with Oxaliplatin (OXA, 0.5 or 1 µM) or with 5-fluorouracil (5-FU, 2.5 or 5 µM) for 72h. One representative experiment out of three is shown. The mean values are tested by one-way analysis of variance (ANOVA). ***p ≤0.001.

Supplementary Table 1.

List of the antibodies used in the experiments reported in this manuscript.

Cell Signaling Technology (Beverly MA USA)	Santa Cruz Biotechnology (Dallas TX USA)	Sigma Aldrich (St Louis MO USA)
Hexokinase I (#2024)	PGC-1α (sc-13067)	anti-β-Actin (F-3022)
Hexokinase II (#2867)	GCK (sc-17819)	
Aldolase (#3188s)	PTEN (sc-7974)	
Enolase (#3810)	ERK1 (sc-93)	
DLAT (#12362)	p-ERK (sc-7383)	
CPT1A (#12252)	c-MYC (sc-40)	
ACAD9 (#9796)	anti-mouse (sc-2031)	
AMPK (#2603)	anti-rabbit (sc-2004)	
p-AMPK (Thr172) (#2335)		
mTOR (#2983)		
p-mTOR (Ser 2448) (#2971)		
P70S6K (#2708)		
p-P70S6K (Thr389) (#9205)		
AKT (#9272)		
p-AKT (Ser473) (#9271)		
p-AKT (Thr308) (#4056)		
PKM2 (#4053S)		
β-Tubulin (#2146)		

Supplementary Table 2.

GSEA, leading edge and IPA pathway analyses performed on tumor samples from a selected TCGA-COAD cohort stratified according to miR-27a levels.

2A) List of metabolic pathways represented in Figure 1C (upper table). Median of metabolic scores in high- vs. low-miR-27a are shown together with significance p values; box plots of metabolic scores for each CRC sample is also shown (on the top right); List of genes coding for metabolic proteins used to calculate metabolic scores (lower table; see also methods

List of metabolic pathways represented in Figure 1C

ID	Pathway	Median Metabolic Score Low-miR-27a (N=71)	gh-miR-27a (N=7	P value	-Log (P)
Glyc	Glycolysis	-1,12	-1,45	0,94	0,03
Pyr. Ox.	Pyruvate oxidation	-0,01	-1,05	0,21	0,68
TCA	Tricarboxylic acid cycle	-1,25	-2,69	0,81	0,09
PPP	Pentose phosphate pathway	-0,70	0,24	0,001	3,00
mTOR sign.	mTOR signaling	0,24	-0,11	0,37	0,43
AMPK sign.	AMPK signaling	0,50	-1,34	0,023	1,64
Gln sign.	Glutaminolysis	0,08	-1,01	0,003	2,52
GSH synth	Glutathione synthesis	-0,38	-0,43	0,63	0,20

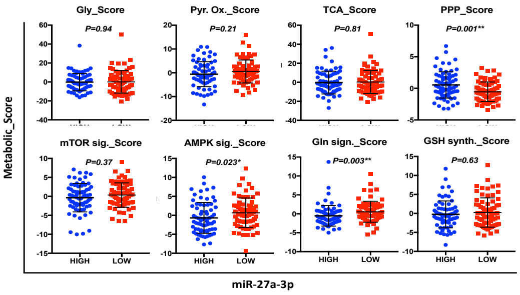

List of genes coding for metabolic proteins used to calculate metabolic scores

ID	Gene name	Activator (A) Inducer (B) Inhibitor (I)
Glyc	GCK	A
	HK1	A
	HK2	A
	HK3	A
	PFKFB1	A
	PFKFB2	A
	PFKFB3	A
	PFKFB4	A
	PFKL	A
	PFKM	A
	PFKP	A
	PKLR	A
	PKM	A
	SLC2A1	A
	SLC2A2	A
	SLC2A3	A
	SLC2A4	A
	SLC2A5	A
	ALDOA	B
	ALDOB	B
	ALDOC	B
	ENO1	B
	ENO2	B
	ENO3	B
	GAPDH	B
	GAPDHS	B
	GPI	B
	PGAM1	B
	PGK1	B
	PGK2	B
	TPI1	B
	PGAM2	B
	GCKR	I
Pyr. Ox.	DLAT	A
	PDHA1	A
	PDHA2	A
	PDHB	A
	PDHX	A
	PDP1	A
	DLD	A
	PDP2	B
	PDPR	B
	MPC1	B
	MPC2	B
	PDK1	I
	PDK2	I
	PDK3	I
	PDK4	I
TCA	CS	A
	DLD	A
	DLST	A
	OGDH	A
	PC	A
	FH	B
	IDH1	B
	IDH2	B
	IDH3A	B
	IDH3B	B
	IDH3G	B
	MDH1	B
	MDH1B	B
	MDH2	B
	ME1	B
	ME2	B
	SUCLA2	B
	SUCLG1	B
	SUCLG2	B
	SDHA	B
	SDHB	B
	SDHC	B
	SDHD	B
	SDHAF1	B
	SDHAF2	B
	ACO1	B
	ACO2	B
	PCK1	I
	PCK2	I
PPP	G6PD	A
	PGD	A
	PGLS	A
mTOR sig.	MTOR	A
	RPTOR	A
	RHEB	B
	RPS6KB1	B
	EIF4E	B
	EIF4B	B
	RPS6	B
	MLST8	B
	TELO2	B
	TTI1	B
	TSC2	I
	TSC1	I
	AKT1S1	I
	DEPTOR	I
	TBC1D7	I
	EIF4EBP1	I
AMPK sig.	STK11	A
	CAB39	A
	STRADB	A
	CAMKK1	A
	CAMKK2	A
	STRADA	A
	PRKAB2	A
	PRKAG1	A
	PRKAG3	A
	PRKAB1	A
	PRKAA2	A
	PRKAG2	A
	PRKAA1	A
	CAB39L	A
	MAP3K7	A
	PPM1E	I
	PPM1F	I
Gln sign.	GLS	A
	GLS2	A
	GLUD1	A
	GLUD2	A
GSH synth.	GCLC	B
	GCLM	B
	GPX1	B
	GPX2	B
	GPX3	B
	GPX4	B
	GPX5	B
	GPX6	B
	GPX7	B
	GPX8	B
	GSR	B
	GSS	B

Supplementary Table 2.

GSEA, leading edge and IPA pathway analyses performed on tumor samples from a selected TCGA-COAD cohort stratified according to miR-27a levels.

2B) Gene-sets enriched in high-miR-27a CRCs;

NAME	GS<br>follow link to MSigDB	GS DETAILS	SIZE	ES	NES	NOM p-val	FDR q-val	FWER p-val	RANK AT MA	LEADING EDGE
GINESTIER_BREAST_CANCER_ZNF217_AMPLIFIED_DN	GINESTIER_BREAST_CANCER_ZNF217_AMPLIFIED_DN	Details ...	306	0,61977124	2,8014293	0	0	0	3763	tags=55%, list=19%, signal=66%
LI_DCP2_BOUND_MRNA	LI_DCP2_BOUND_MRNA	Details ...	87	0,63672185	2,4697342	0	0	0	4134	tags=62%, list=21%, signal=78%
WAMUNYOKOLI_OVARIAN_CANCER_LMP_UP	WAMUNYOKOLI_OVARIAN_CANCER_LMP_UP	Details ...	244	0,5614954	2,468834	0	0	0	3231	tags=46%, list=16%, signal=55%
NIKOLSKY_BREAST_CANCER_16P13_AMPLICON	NIKOLSKY_BREAST_CANCER_16P13_AMPLICON	Details ...	111	0,6043511	2,3895779	0	0	0	4472	tags=51%, list=22%, signal=66%
MOOTHA_VOXPHOS	MOOTHA_VOXPHOS	Details ...	85	0,6166583	2,3765981	0	0	0	4522	tags=68%, list=23%, signal=88%
DAIRKEE_TERT_TARGETS_UP	DAIRKEE_TERT_TARGETS_UP	Details ...	343	0,5164307	2,3388069	0	0	0	4144	tags=48%, list=21%, signal=60%
NIKOLSKY_BREAST_CANCER_16Q24_AMPLICON	NIKOLSKY_BREAST_CANCER_16Q24_AMPLICON	Details ...	46	0,6841178	2,324745	0	0	0	3452	tags=50%, list=17%, signal=60%
BILANGES_SERUM_AND_RAPAMYCIN_SENSITIVE_GENES	BILANGES_SERUM_AND_RAPAMYCIN_SENSITIVE_GENES	Details ...	68	0,62630635	2,2839308	0	9,18E-05	0,001	4794	tags=62%, list=24%, signal=81%
CHNG_MULTIPLE_MYELOMA_HYPERPLOID_UP	CHNG_MULTIPLE_MYELOMA_HYPERPLOID_UP	Details ...	52	0,6452931	2,265758	0	8,16E-05	0,001	4309	tags=69%, list=22%, signal=88%
PECE_MAMMARY_STEM_CELL_UP	PECE_MAMMARY_STEM_CELL_UP	Details ...	135	0,5571837	2,2543752	0	7,35E-05	0,001	5383	tags=67%, list=27%, signal=91%
NIKOLSKY_BREAST_CANCER_1Q21_AMPLICON	NIKOLSKY_BREAST_CANCER_1Q21_AMPLICON	Details ...	37	0,69354004	2,2526822	0	6,68E-05	0,001	4597	tags=68%, list=23%, signal=88%
LU_EZH2_TARGETS_UP	LU_EZH2_TARGETS_UP	Details ...	264	0,49526238	2,1703641	0	3,71E-04	0,006	4180	tags=47%, list=21%, signal=59%
NIKOLSKY_BREAST_CANCER_12Q24_AMPLICON	NIKOLSKY_BREAST_CANCER_12Q24_AMPLICON	Details ...	15	0,8187875	2,1501544	0	5,11E-04	0,009	2701	tags=60%, list=14%, signal=69%
CHARAFE_BREAST_CANCER_LUMINAL_VS_MESENCHYMAL_UP	CHARAFE_BREAST_CANCER_LUMINAL_VS_MESENCHYMAL_UP	Details ...	418	0,46311936	2,1349561	0	5,27E-04	0,01	3722	tags=43%, list=19%, signal=51%
KIM_ALL_DISORDERS_DURATION_CORR_DN	KIM_ALL_DISORDERS_DURATION_CORR_DN	Details ...	140	0,5251871	2,1031315	0	0,00112455	0,022	4144	tags=56%, list=21%, signal=71%
SPIELMAN_LYMPHOBLAST_EUROPEAN_VS_ASIAN_UP	SPIELMAN_LYMPHOBLAST_EUROPEAN_VS_ASIAN_UP	Details ...	467	0,4528463	2,0859635	0	0,00151184	0,031	3791	tags=46%, list=19%, signal=55%
SCHLOSSER_MYC_AND_SERUM_RESPONSE_SYNERGY	SCHLOSSER_MYC_AND_SERUM_RESPONSE_SYNERGY	Details ...	31	0,6687953	2,0815234	0	0,00163839	0,036	2348	tags=61%, list=12%, signal=69%
WONG_MITOCHONDRIA_GENE_MODULE	WONG_MITOCHONDRIA_GENE_MODULE	Details ...	215	0,47671196	2,047557	0	0,00328799	0,078	5034	tags=58%, list=25%, signal=77%
CREIGHTON_AKT1_SIGNALING_VIA_MTOR_UP	CREIGHTON_AKT1_SIGNALING_VIA_MTOR_UP	Details ...	34	0,6498718	2,0460303	0,0025974	0,00326896	0,082	3925	tags=59%, list=20%, signal=73%
YAO_TEMPORAL_RESPONSE_TO_PROGESTERONE_CLUSTER_13	YAO_TEMPORAL_RESPONSE_TO_PROGESTERONE_CLUSTER_13	Details ...	159	0,4992591	2,0386188	0	0,00339596	0,09	3982	tags=50%, list=20%, signal=62%
SHEDDEN_LUNG_CANCER_GOOD_SURVIVAL_A5	SHEDDEN_LUNG_CANCER_GOOD_SURVIVAL_A5		63	0,5509695	2,0292678	0	0,00372008	0,103	2497	tags=41%, list=12%, signal=47%
RUNNE_GENDER_EFFECT_UP	RUNNE_GENDER_EFFECT_UP		8	0,93734765	2,0270898	0	0,00361753	0,105	918	tags=88%, list=5%, signal=92%
LIN_APC_TARGETS	LIN_APC_TARGETS		76	0,53565	1,9934074	0	0,00546252	0,157	4311	tags=49%, list=22%, signal=62%
WELCSH_BRCA1_TARGETS_DN	WELCSH_BRCA1_TARGETS_DN		139	0,49500626	1,9928306	0	0,00523492	0,157	3211	tags=41%, list=16%, signal=49%
CROMER_METASTASIS_DN	CROMER_METASTASIS_DN		79	0,5261268	1,9887983	0	0,00525999	0,162	3039	tags=41%, list=15%, signal=48%
WEBER_METHYLATED_HCP_IN_SPERM_UP	WEBER_METHYLATED_HCP_IN_SPERM_UP		20	0,7094615	1,9790269	0,00257069	0,00576544	0,186	3657	tags=35%, list=18%, signal=43%
JIANG_TIP30_TARGETS_DN	JIANG_TIP30_TARGETS_DN		23	0,66534203	1,9746488	0,00544959	0,00601622	0,2	1679	tags=39%, list=8%, signal=43%
HOLLEMAN_ASPARAGINASE_RESISTANCE_ALL_UP	HOLLEMAN_ASPARAGINASE_RESISTANCE_ALL_UP		22	0,6866668	1,9739554	0	0,00585189	0,201	1920	tags=45%, list=10%, signal=50%
CHARAFE_BREAST_CANCER_BASAL_VS_MESENCHYMAL_UP	CHARAFE_BREAST_CANCER_BASAL_VS_MESENCHYMAL_UP		112	0,49292246	1,9714457	0	0,00580213	0,207	3264	tags=41%, list=16%, signal=49%
CREIGHTON_AKT1_SIGNALING_VIA_MTOR_DN	CREIGHTON_AKT1_SIGNALING_VIA_MTOR_DN		23	0,6894869	1,96952	0	0,00568052	0,208	5034	tags=78%, list=25%, signal=104%
MOOTHA_HUMAN_MITODB_6_2002	MOOTHA_HUMAN_MITODB_6_2002		418	0,42412034	1,9623524	0	0,00608409	0,228	4002	tags=41%, list=20%, signal=51%
LI_AMPLIFIED_IN_LUNG_CANCER	LI_AMPLIFIED_IN_LUNG_CANCER		174	0,47027424	1,9538959	0	0,00676457	0,257	3573	tags=39%, list=18%, signal=47%
SCHLOSSER_SERUM_RESPONSE_AUGMENTED_BY_MYC	SCHLOSSER_SERUM_RESPONSE_AUGMENTED_BY_MYC		101	0,49921402	1,9449719	0	0,00752847	0,284	3028	tags=47%, list=15%, signal=55%
HOLLEMAN_VINCRISTINE_RESISTANCE_ALL_DN	HOLLEMAN_VINCRISTINE_RESISTANCE_ALL_DN		19	0,6985665	1,9374452	0,00247525	0,00799416	0,306	4445	tags=84%, list=22%, signal=108%
MOOTHA_MITOCHONDRIA	MOOTHA_MITOCHONDRIA		431	0,41950506	1,9163755	0	0,01070632	0,403	4051	tags=41%, list=20%, signal=50%
OKAWA_NEUROBLASTOMA_1P36_31_DELETION	OKAWA_NEUROBLASTOMA_1P36_31_DELETION		22	0,66468775	1,9153379	0	0,0105723	0,407	1521	tags=41%, list=8%, signal=44%
SANSOM_APC_TARGETS_REQUIRE_MYC	SANSOM_APC_TARGETS_REQUIRE_MYC		191	0,4463298	1,9127492	0	0,0106844	0,417	4183	tags=48%, list=21%, signal=60%
DAZARD_UV_RESPONSE_CLUSTER_G5	DAZARD_UV_RESPONSE_CLUSTER_G5		12	0,7863257	1,9100306	0	0,01067362	0,425	2520	tags=58%, list=13%, signal=67%
WHITE_NEUROBLASTOMA_WITH_1P36.3_DELETION	WHITE_NEUROBLASTOMA_WITH_1P36.3_DELETION		20	0,66898453	1,8998445	0	0,0121045	0,486	1521	tags=45%, list=8%, signal=49%
LIU_CDX2_TARGETS_DN	LIU_CDX2_TARGETS_DN		8	0,86239207	1,8763633	0	0,01619142	0,602	1689	tags=38%, list=8%, signal=41%
SABATES_COLORECTAL_ADENOMA_UP	SABATES_COLORECTAL_ADENOMA_UP		128	0,45642254	1,8691233	0	0,0171702	0,628	5801	tags=51%, list=29%, signal=71%
HOLLEMAN_VINCRISTINE_RESISTANCE_B_ALL_DN	HOLLEMAN_VINCRISTINE_RESISTANCE_B_ALL_DN		15	0,7033762	1,8689619	0,00471698	0,01679729	0,629	4155	tags=87%, list=21%, signal=109%
TAKAO_RESPONSE_TO_UVB_RADIATION_UP	TAKAO_RESPONSE_TO_UVB_RADIATION_UP		86	0,488814	1,8606431	0	0,01830847	0,669	4828	tags=60%, list=24%, signal=79%
NIKOLSKY_BREAST_CANCER_17Q21_Q25_AMPLICON	NIKOLSKY_BREAST_CANCER_17Q21_Q25_AMPLICON		313	0,4168596	1,8549353	0	0,01905478	0,691	3100	tags=32%, list=15%, signal=37%
NIKOLSKY_BREAST_CANCER_7P22_AMPLICON	NIKOLSKY_BREAST_CANCER_7P22_AMPLICON		34	0,59195644	1,8495029	0	0,0197533	0,711	3301	tags=59%, list=17%, signal=70%
ODONNELL_METASTASIS_DN	ODONNELL_METASTASIS_DN		23	0,62623036	1,8356116	0	0,02246768	0,766	3385	tags=43%, list=17%, signal=52%
TIMOFEEVA_GROWTH_STRESS_VIA_STAT1_DN	TIMOFEEVA_GROWTH_STRESS_VIA_STAT1_DN		16	0,70214176	1,8280098	0,00247525	0,02382766	0,788	4630	tags=75%, list=23%, signal=98%
SENGUPTA_EBNA1_ANTICORRELATED	SENGUPTA_EBNA1_ANTICORRELATED		142	0,44266927	1,8183287	0	0,02631569	0,838	3176	tags=35%, list=16%, signal=42%
RICKMAN_METASTASIS_DN	RICKMAN_METASTASIS_DN		243	0,41624513	1,8157729	0	0,02655714	0,843	3760	tags=39%, list=19%, signal=48%
NIKOLSKY_BREAST_CANCER_20Q12_Q13_AMPLICON	NIKOLSKY_BREAST_CANCER_20Q12_Q13_AMPLICON		129	0,43961748	1,8137465	0	0,02649285	0,849	3954	tags=35%, list=20%, signal=43%
ZUCCHI_METASTASIS_DN	ZUCCHI_METASTASIS_DN		41	0,5523463	1,8115171	0	0,02668107	0,86	4012	tags=49%, list=20%, signal=61%
BARRIER_CANCER_RELAPSE_NORMAL_SAMPLE_DN	BARRIER_CANCER_RELAPSE_NORMAL_SAMPLE_DN		28	0,5964707	1,8013793	0,00288184	0,02909372	0,895	3646	tags=64%, list=18%, signal=79%
LAIHO_COLORECTAL_CANCER_SERRATED_DN	LAIHO_COLORECTAL_CANCER_SERRATED_DN		77	0,47419295	1,7941638	0	0,03116865	0,917	4034	tags=48%, list=20%, signal=60%
HSIAO_HOUSEKEEPING_GENES	HSIAO_HOUSEKEEPING_GENES		378	0,3936959	1,782124	0	0,0350607	0,945	3954	tags=41%, list=20%, signal=50%
FAELT_B_CLL_WITH_VH3_21_UP	FAELT_B_CLL_WITH_VH3_21_UP		42	0,54012704	1,7798955	0,00303951	0,0352087	0,946	3028	tags=48%, list=15%, signal=56%
NIKOLSKY_BREAST_CANCER_20Q11_AMPLICON	NIKOLSKY_BREAST_CANCER_20Q11_AMPLICON		28	0,58151084	1,7757787	0,0027248	0,03611989	0,954	3291	tags=50%, list=16%, signal=60%
YANG_BREAST_CANCER_ESR1_LASER_UP	YANG_BREAST_CANCER_ESR1_LASER_UP		32	0,5750255	1,7740098	0,00555556	0,03612828	0,955	2336	tags=41%, list=12%, signal=46%
LUI_THYROID_CANCER_CLUSTER_3	LUI_THYROID_CANCER_CLUSTER_3		28	0,58037764	1,773409	0,00527705	0,03577017	0,955	3929	tags=50%, list=20%, signal=62%
LIM_MAMMARY_STEM_CELL_DN	LIM_MAMMARY_STEM_CELL_DN		405	0,38666487	1,7715669	0	0,03598473	0,958	4339	tags=41%, list=22%, signal=52%
BONOME_OVARIAN_CANCER_POOR_SURVIVAL_DN	BONOME_OVARIAN_CANCER_POOR_SURVIVAL_DN		20	0,6172401	1,7547132	0,01015228	0,04219415	0,979	3721	tags=60%, list=19%, signal=74%
NIKOLSKY_BREAST_CANCER_5P15_AMPLICON	NIKOLSKY_BREAST_CANCER_5P15_AMPLICON		25	0,59309	1,7476573	0	0,04470218	0,982	4638	tags=40%, list=23%, signal=52%
HONMA_DOCETAXEL_RESISTANCE	HONMA_DOCETAXEL_RESISTANCE		32	0,5554958	1,7464287	0,00251889	0,04453478	0,982	4014	tags=56%, list=20%, signal=70%
DAZARD_UV_RESPONSE_CLUSTER_G4	DAZARD_UV_RESPONSE_CLUSTER_G4		20	0,60824084	1,7255377	0,01084011	0,05466839	0,994	5206	tags=55%, list=26%, signal=74%
MUELLER_COMMON_TARGETS_OF_AML_FUSIONS_UP	MUELLER_COMMON_TARGETS_OF_AML_FUSIONS_UP		14	0,6759497	1,7184759	0,01522843	0,05784596	0,996	1846	tags=43%, list=9%, signal=47%
BILANGES_RAPAMYCIN_SENSITIVE_VIA_TSC1_AND_TSC2	BILANGES_RAPAMYCIN_SENSITIVE_VIA_TSC1_AND_TSC2		69	0,45817512	1,6943016	0	0,07191318	1	3214	tags=41%, list=16%, signal=48%
GENTILE_UV_LOW_DOSE_UP	GENTILE_UV_LOW_DOSE_UP		27	0,5579804	1,6835866	0,00789474	0,07780741	1	4610	tags=59%, list=23%, signal=77%
STARK_PREFRONTAL_CORTEX_22Q11_DELETION_DN	STARK_PREFRONTAL_CORTEX_22Q11_DELETION_DN		465	0,36245567	1,6760212	0	0,08236612	1	3374	tags=34%, list=17%, signal=40%
GRADE_METASTASIS_DN	GRADE_METASTASIS_DN		43	0,4990298	1,6709152	0,0026738	0,08495686	1	4954	tags=63%, list=25%, signal=83%
YANAGISAWA_LUNG_CANCER_RECURRENCE	YANAGISAWA_LUNG_CANCER_RECURRENCE		7	0,8083519	1,6690421	0,01631702	0,08496416	1	3414	tags=86%, list=17%, signal=103%
FOSTER_KDM1A_TARGETS_DN	FOSTER_KDM1A_TARGETS_DN		196	0,3892077	1,6684625	0	0,08410447	1	3118	tags=33%, list=16%, signal=39%
YAO_TEMPORAL_RESPONSE_TO_PROGESTERONE_CLUSTER_17	YAO_TEMPORAL_RESPONSE_TO_PROGESTERONE_CLUSTER_17		172	0,39954036	1,668025	0	0,08319925	1	3984	tags=41%, list=20%, signal=50%
KIM_GASTRIC_CANCER_CHEMOSENSITIVITY	KIM_GASTRIC_CANCER_CHEMOSENSITIVITY		95	0,42754507	1,6580029	0	0,0900038	1	3720	tags=39%, list=19%, signal=48%
BILANGES_SERUM_RESPONSE_TRANSLATION	BILANGES_SERUM_RESPONSE_TRANSLATION		36	0,51448333	1,6548424	0,01139601	0,09136397	1	3786	tags=33%, list=19%, signal=41%

Supplementary Table 2.

GSEA, leading edge and IPA pathway analyses performed on tumor samples from a selected TCGA-COAD cohort stratified according to miR-27a levels.

2C) Gene-sets enriched in low-miR-27a CRCs;

NAME	GS<br>follow link to MSigDB	GS DETAILS	SIZE	ES	NES	NOM p-val	FDR q-val	FWER p-val	RANK AT MAX	LEADING EDGE
NIKOLSKY_BREAST_CANCER_8Q12_Q22_AMPLICON	NIKOLSKY_BREAST_CANCER_8Q12_Q22_AMPLICON	Details ...	123	-0,7006094	-2,5147116	0	0	0	3309	tags=56%, list=17%, signal=67%
SCHUETZ_BREAST_CANCER_DUCTAL_INVASIVE_UP	SCHUETZ_BREAST_CANCER_DUCTAL_INVASIVE_UP	Details ...	325	-0,6129879	-2,4288673	0	0	0	5959	tags=74%, list=30%, signal=104%
GABRIELY_MIR21_TARGETS	GABRIELY_MIR21_TARGETS	Details ...	279	-0,6163254	-2,4268758	0	0	0	3912	tags=55%, list=20%, signal=67%
ZHENG_FOXP3_TARGETS_IN_THYMUS_UP	ZHENG_FOXP3_TARGETS_IN_THYMUS_UP	Details ...	182	-0,6206951	-2,3675563	0	0	0	4918	tags=63%, list=25%, signal=82%
DACOSTA_UV_RESPONSE_VIA_ERCC3_COMMON_DN	DACOSTA_UV_RESPONSE_VIA_ERCC3_COMMON_DN	Details ...	466	-0,5654507	-2,313893	0	0	0	5517	tags=61%, list=28%, signal=82%
SHEN_SMARCA2_TARGETS_UP	SHEN_SMARCA2_TARGETS_UP	Details ...	410	-0,56636244	-2,2862017	0	0	0	5516	tags=58%, list=28%, signal=79%
GINESTIER_BREAST_CANCER_ZNF217_AMPLIFIED_UP	GINESTIER_BREAST_CANCER_ZNF217_AMPLIFIED_UP	Details ...	70	-0,69384956	-2,2847683	0	0	0	3105	tags=64%, list=16%, signal=76%
WAMUNYOKOLI_OVARIAN_CANCER_LMP_DN	WAMUNYOKOLI_OVARIAN_CANCER_LMP_DN	Details ...	185	-0,596337	-2,2509537	0	0	0	5283	tags=66%, list=26%, signal=89%
VECCHI_GASTRIC_CANCER_ADVANCED_VS_EARLY_UP	VECCHI_GASTRIC_CANCER_ADVANCED_VS_EARLY_UP	Details ...	165	-0,5963601	-2,2191494	0	1,25E-04	0,001	6328	tags=76%, list=32%, signal=110%
LINDGREN_BLADDER_CANCER_CLUSTER_2B	LINDGREN_BLADDER_CANCER_CLUSTER_2B	Details ...	369	-0,5550859	-2,217552	0	1,13E-04	0,001	6100	tags=68%, list=30%, signal=96%
MILI_PSEUDOPODIA_HAPTOTAXIS_UP	MILI_PSEUDOPODIA_HAPTOTAXIS_UP	Details ...	484	-0,5357085	-2,171551	0	1,02E-04	0,001	4403	tags=47%, list=22%, signal=59%
OSMAN_BLADDER_CANCER_UP	OSMAN_BLADDER_CANCER_UP	Details ...	386	-0,5374869	-2,1590774	0	9,39E-05	0,001	3512	tags=46%, list=18%, signal=55%
WANG_SMARCE1_TARGETS_UP	WANG_SMARCE1_TARGETS_UP	Details ...	271	-0,53919315	-2,1292868	0	8,67E-05	0,001	6348	tags=63%, list=32%, signal=92%
SMID_BREAST_CANCER_NORMAL_LIKE_UP	SMID_BREAST_CANCER_NORMAL_LIKE_UP	Details ...	442	-0,52696127	-2,1292453	0	8,05E-05	0,001	6091	tags=62%, list=30%, signal=87%
JOHNSTONE_PARVB_TARGETS_2_DN	JOHNSTONE_PARVB_TARGETS_2_DN	Details ...	312	-0,5359187	-2,123969	0	7,51E-05	0,001	4813	tags=54%, list=24%, signal=71%
PICCALUGA_ANGIOIMMUNOBLASTIC_LYMPHOMA_UP	PICCALUGA_ANGIOIMMUNOBLASTIC_LYMPHOMA_UP	Details ...	196	-0,5647805	-2,122693	0	7,04E-05	0,001	6617	tags=77%, list=33%, signal=113%
WALLACE_PROSTATE_CANCER_RACE_UP	WALLACE_PROSTATE_CANCER_RACE_UP	Details ...	267	-0,5428804	-2,1208642	0	6,63E-05	0,001	5287	tags=57%, list=26%, signal=76%
CROONQUIST_NRAS_SIGNALING_UP	CROONQUIST_NRAS_SIGNALING_UP	Details ...	38	-0,6987006	-2,111943	0	6,26E-05	0,001	4151	tags=66%, list=21%, signal=83%
LIAN_LIPA_TARGETS_3M	LIAN_LIPA_TARGETS_3M	Details ...	57	-0,65302056	-2,109934	0	5,93E-05	0,001	4335	tags=60%, list=22%, signal=76%
ZHENG_BOUND_BY_FOXP3	ZHENG_BOUND_BY_FOXP3	Details ...	463	-0,51749974	-2,1077492	0	5,63E-05	0,001	4833	tags=50%, list=24%, signal=65%
POOLA_INVASIVE_BREAST_CANCER_UP	POOLA_INVASIVE_BREAST_CANCER_UP		265	-0,53872585	-2,1015718	0	5,36E-05	0,001	5109	tags=53%, list=26%, signal=70%
LIAN_LIPA_TARGETS_6M	LIAN_LIPA_TARGETS_6M		72	-0,6297944	-2,0964875	0	5,12E-05	0,001	4340	tags=53%, list=22%, signal=67%
MCLACHLAN_DENTAL_CARIES_UP	MCLACHLAN_DENTAL_CARIES_UP		224	-0,543708	-2,0943847	0	4,90E-05	0,001	5180	tags=58%, list=26%, signal=77%
DE_YY1_TARGETS_DN	DE_YY1_TARGETS_DN		91	-0,5983978	-2,0846815	0	4,69E-05	0,001	3948	tags=49%, list=20%, signal=61%
SABATES_COLORECTAL_ADENOMA_DN	SABATES_COLORECTAL_ADENOMA_DN		266	-0,5347776	-2,0793543	0	4,51E-05	0,001	8310	tags=75%, list=42%, signal=126%
PLASARI_TGFB1_TARGETS_10HR_DN	PLASARI_TGFB1_TARGETS_10HR_DN		238	-0,53687245	-2,0756593	0	4,33E-05	0,001	5988	tags=63%, list=30%, signal=88%
SENESE_HDAC1_TARGETS_UP	SENESE_HDAC1_TARGETS_UP		424	-0,51125014	-2,0714128	0	4,17E-05	0,001	4806	tags=47%, list=24%, signal=61%
SENGUPTA_NASOPHARYNGEAL_CARCINOMA_UP	SENGUPTA_NASOPHARYNGEAL_CARCINOMA_UP		278	-0,5263592	-2,06641	0	4,02E-05	0,001	6418	tags=65%, list=32%, signal=95%
SENESE_HDAC2_TARGETS_UP	SENESE_HDAC2_TARGETS_UP		107	-0,5825622	-2,0612206	0	1,16E-04	0,003	4156	tags=50%, list=21%, signal=63%
IKEDA_MIR30_TARGETS_UP	IKEDA_MIR30_TARGETS_UP		114	-0,5747332	-2,05768	0	1,12E-04	0,003	4991	tags=61%, list=25%, signal=81%
DAZARD_UV_RESPONSE_CLUSTER_G6	DAZARD_UV_RESPONSE_CLUSTER_G6		142	-0,56146395	-2,0542617	0	1,09E-04	0,003	5485	tags=57%, list=27%, signal=78%
THUM_SYSTOLIC_HEART_FAILURE_UP	THUM_SYSTOLIC_HEART_FAILURE_UP		389	-0,51130956	-2,0415652	0	1,41E-04	0,004	5890	tags=60%, list=29%, signal=83%
CHARAFE_BREAST_CANCER_LUMINAL_VS_MESENCHYMAL_DN	CHARAFE_BREAST_CANCER_LUMINAL_VS_MESENCHYMAL_DN		443	-0,501861	-2,0383823	0	1,37E-04	0,004	5554	tags=56%, list=28%, signal=75%
JAATINEN_HEMATOPOIETIC_STEM_CELL_DN	JAATINEN_HEMATOPOIETIC_STEM_CELL_DN		217	-0,52946466	-2,0277543	0	2,00E-04	0,006	5571	tags=56%, list=28%, signal=77%
VECCHI_GASTRIC_CANCER_EARLY_DN	VECCHI_GASTRIC_CANCER_EARLY_DN		332	-0,51500654	-2,027517	0	1,94E-04	0,006	7461	tags=61%, list=37%, signal=95%
KIM_GERMINAL_CENTER_T_HELPER_UP	KIM_GERMINAL_CENTER_T_HELPER_UP		62	-0,6104188	-2,0186632	0	2,84E-04	0,009	3336	tags=53%, list=17%, signal=64%
BERENJENO_ROCK_SIGNALING_NOT_VIA_RHOA_DN	BERENJENO_ROCK_SIGNALING_NOT_VIA_RHOA_DN		44	-0,64384764	-2,0155196	0	3,37E-04	0,011	3428	tags=57%, list=17%, signal=68%
LIM_MAMMARY_STEM_CELL_UP	LIM_MAMMARY_STEM_CELL_UP		456	-0,4955462	-2,0149078	0	3,28E-04	0,011	6586	tags=62%, list=33%, signal=90%
NAKAYAMA_SOFT_TISSUE_TUMORS_PCA1_UP	NAKAYAMA_SOFT_TISSUE_TUMORS_PCA1_UP		69	-0,5987683	-2,0019944	0	6,09E-04	0,021	4527	tags=59%, list=23%, signal=77%
TURASHVILI_BREAST_LOBULAR_CARCINOMA_VS_DUCTAL_NORMAL_UP	TURASHVILI_BREAST_LOBULAR_CARCINOMA_VS_DUCTAL_NORMAL_UP		67	-0,6032891	-1,9982352	0	6,22E-04	0,022	6140	tags=73%, list=31%, signal=105%
ANASTASSIOU_MULTICANCER_INVASIVENESS_SIGNATURE	ANASTASSIOU_MULTICANCER_INVASIVENESS_SIGNATURE		63	-0,6091951	-1,9969862	0	6,62E-04	0,024	7021	tags=87%, list=35%, signal=134%
MARTORIATI_MDM4_TARGETS_NEUROEPITHELIUM_DN	MARTORIATI_MDM4_TARGETS_NEUROEPITHELIUM_DN		153	-0,5401867	-1,9940957	0	6,73E-04	0,025	5539	tags=51%, list=28%, signal=70%
HADDAD_T_LYMPHOCYTE_AND_NK_PROGENITOR_DN	HADDAD_T_LYMPHOCYTE_AND_NK_PROGENITOR_DN		61	-0,60978395	-1,9916737	0	7,10E-04	0,027	5567	tags=56%, list=28%, signal=77%
DAZARD_RESPONSE_TO_UV_NHEK_DN	DAZARD_RESPONSE_TO_UV_NHEK_DN		301	-0,5009451	-1,9873191	0	7,44E-04	0,029	5320	tags=51%, list=27%, signal=69%
BEGUM_TARGETS_OF_PAX3_FOXO1_FUSION_UP	BEGUM_TARGETS_OF_PAX3_FOXO1_FUSION_UP		60	-0,61255735	-1,9835445	0	7,78E-04	0,031	5616	tags=62%, list=28%, signal=85%
TAKEDA_TARGETS_OF_NUP98_HOXA9_FUSION_10D_DN	TAKEDA_TARGETS_OF_NUP98_HOXA9_FUSION_10D_DN		132	-0,53661364	-1,971649	0	0,001007686	0,041	6373	tags=64%, list=32%, signal=94%
LINDGREN_BLADDER_CANCER_CLUSTER_2A_DN	LINDGREN_BLADDER_CANCER_CLUSTER_2A_DN		134	-0,53968716	-1,9672546	0	0,00110746	0,045	6042	tags=66%, list=30%, signal=95%
CHIANG_LIVER_CANCER_SUBCLASS_INTERFERON_UP	CHIANG_LIVER_CANCER_SUBCLASS_INTERFERON_UP		26	-0,7040112	-1,9635183	0	0,001202218	0,05	2616	tags=46%, list=13%, signal=53%
BYSTROEM_CORRELATED_WITH_IL5_DN	BYSTROEM_CORRELATED_WITH_IL5_DN		64	-0,5959227	-1,9612213	0	0,001247076	0,052	4677	tags=56%, list=23%, signal=73%
PLASARI_TGFB1_SIGNALING_VIA_NFIC_1HR_UP	PLASARI_TGFB1_SIGNALING_VIA_NFIC_1HR_UP		33	-0,68144774	-1,9587841	0	0,001222135	0,052	3703	tags=61%, list=19%, signal=74%
CHICAS_RB1_TARGETS_GROWING	CHICAS_RB1_TARGETS_GROWING		232	-0,5140095	-1,9578447	0	0,001198171	0,052	6008	tags=57%, list=30%, signal=81%
DEURIG_T_CELL_PROLYMPHOCYTIC_LEUKEMIA_DN	DEURIG_T_CELL_PROLYMPHOCYTIC_LEUKEMIA_DN		294	-0,49500477	-1,954054	0	0,001349836	0,06	5331	tags=55%, list=27%, signal=74%
TAKEDA_TARGETS_OF_NUP98_HOXA9_FUSION_16D_UP	TAKEDA_TARGETS_OF_NUP98_HOXA9_FUSION_16D_UP		162	-0,5262164	-1,9526672	0	0,001345728	0,061	4133	tags=47%, list=21%, signal=59%
ONDER_CDH1_TARGETS_2_UP	ONDER_CDH1_TARGETS_2_UP		242	-0,50487345	-1,9519813	0	0,001320807	0,061	7231	tags=69%, list=36%, signal=106%
SENESE_HDAC3_TARGETS_UP	SENESE_HDAC3_TARGETS_UP		468	-0,47778296	-1,947896	0	0,001357787	0,064	4814	tags=45%, list=24%, signal=57%
HUTTMANN_B_CLL_POOR_SURVIVAL_DN	HUTTMANN_B_CLL_POOR_SURVIVAL_DN		56	-0,61092997	-1,9470125	0	0,00133354	0,064	4723	tags=61%, list=24%, signal=79%
ZHANG_TLX_TARGETS_36HR_DN	ZHANG_TLX_TARGETS_36HR_DN		183	-0,5148443	-1,946974	0	0,001310145	0,064	4036	tags=45%, list=20%, signal=56%
FARMER_BREAST_CANCER_CLUSTER_1	FARMER_BREAST_CANCER_CLUSTER_1		39	-0,655212	-1,9460291	0	0,001306839	0,065	5106	tags=69%, list=26%, signal=93%
MARKEY_RB1_CHRONIC_LOF_DN	MARKEY_RB1_CHRONIC_LOF_DN		113	-0,54478645	-1,9456868	0	0,001303704	0,066	5053	tags=54%, list=25%, signal=72%
BOSCO_TH1_CYTOTOXIC_MODULE	BOSCO_TH1_CYTOTOXIC_MODULE		110	-0,54809713	-1,9450778	0	0,001338304	0,069	5015	tags=55%, list=25%, signal=74%
BOQUEST_STEM_CELL_UP	BOQUEST_STEM_CELL_UP		257	-0,49839628	-1,9444846	0	0,001334881	0,07	7320	tags=68%, list=37%, signal=106%
FULCHER_INFLAMMATORY_RESPONSE_LECTIN_VS_LPS_DN	FULCHER_INFLAMMATORY_RESPONSE_LECTIN_VS_LPS_DN		429	-0,47451508	-1,9339613	0	0,001568553	0,082	5346	tags=52%, list=27%, signal=70%
SEIDEN_ONCOGENESIS_BY_MET	SEIDEN_ONCOGENESIS_BY_MET		84	-0,56898004	-1,9287039	0	0,001723504	0,092	3859	tags=46%, list=19%, signal=57%
HORIUCHI_WTAP_TARGETS_UP	HORIUCHI_WTAP_TARGETS_UP		285	-0,48911294	-1,9252473	0	0,001855401	0,099	4893	tags=49%, list=24%, signal=65%
WINTER_HYPOXIA_DN	WINTER_HYPOXIA_DN		48	-0,6167749	-1,924763	0	0,001861772	0,1	4388	tags=63%, list=22%, signal=80%
GARGALOVIC_RESPONSE_TO_OXIDIZED_PHOSPHOLIPIDS_YELLOW_UP	GARGALOVIC_RESPONSE_TO_OXIDIZED_PHOSPHOLIPIDS_YELLOW_UP		26	-0,70031154	-1,9239124	0	0,00185077	0,1	1831	tags=50%, list=9%, signal=55%
DEBIASI_APOPTOSIS_BY_REOVIRUS_INFECTION_UP	DEBIASI_APOPTOSIS_BY_REOVIRUS_INFECTION_UP		304	-0,48547694	-1,9215014	0	0,00187339	0,102	4612	tags=45%, list=23%, signal=58%
CHEN_HOXA5_TARGETS_9HR_UP	CHEN_HOXA5_TARGETS_9HR_UP		206	-0,5055191	-1,9212695	0	0,001862703	0,103	4293	tags=44%, list=21%, signal=55%
LEE_DIFFERENTIATING_T_LYMPHOCYTE	LEE_DIFFERENTIATING_T_LYMPHOCYTE		181	-0,5063532	-1,9109691	0	0,002276745	0,127	5232	tags=59%, list=26%, signal=79%
LU_AGING_BRAIN_UP	LU_AGING_BRAIN_UP		254	-0,4890837	-1,90889	0	0,002373667	0,133	4856	tags=48%, list=24%, signal=63%
BAELDE_DIABETIC_NEPHROPATHY_DN	BAELDE_DIABETIC_NEPHROPATHY_DN		423	-0,4706551	-1,9081876	0	0,002356209	0,134	6084	tags=55%, list=30%, signal=77%
SWEET_LUNG_CANCER_KRAS_DN	SWEET_LUNG_CANCER_KRAS_DN		419	-0,47318757	-1,9081788	0	0,002323484	0,134	5424	tags=45%, list=27%, signal=61%
KONDO_PROSTATE_CANCER_WITH_H3K27ME3	KONDO_PROSTATE_CANCER_WITH_H3K27ME3		181	-0,50300425	-1,907325	0	0,002322406	0,135	5779	tags=32%, list=29%, signal=45%
SAKAI_CHRONIC_HEPATITIS_VS_LIVER_CANCER_UP	SAKAI_CHRONIC_HEPATITIS_VS_LIVER_CANCER_UP		78	-0,565366	-1,9044738	0	0,002458278	0,146	5413	tags=58%, list=27%, signal=79%
ACEVEDO_LIVER_CANCER_WITH_H3K27ME3_UP	ACEVEDO_LIVER_CANCER_WITH_H3K27ME3_UP		257	-0,4872621	-1,9006779	0	0,002560739	0,154	5961	tags=45%, list=30%, signal=63%
SHARMA_PILOCYTIC_ASTROCYTOMA_LOCATION_UP	SHARMA_PILOCYTIC_ASTROCYTOMA_LOCATION_UP		22	-0,71019715	-1,9006475	0	0,002527044	0,154	4637	tags=55%, list=23%, signal=71%
LINDSTEDT_DENDRITIC_CELL_MATURATION_D	LINDSTEDT_DENDRITIC_CELL_MATURATION_D		64	-0,5830873	-1,8972208	0	0,002699933	0,163	5375	tags=61%, list=27%, signal=83%
BONOME_OVARIAN_CANCER_POOR_SURVIVAL_UP	BONOME_OVARIAN_CANCER_POOR_SURVIVAL_UP		31	-0,6636852	-1,8971632	0	0,002665318	0,163	2995	tags=55%, list=15%, signal=64%
SCHAEFFER_PROSTATE_DEVELOPMENT_6HR_UP	SCHAEFFER_PROSTATE_DEVELOPMENT_6HR_UP		160	-0,5082074	-1,8957082	0	0,002760622	0,17	5694	tags=58%, list=28%, signal=80%
PETROVA_PROX1_TARGETS_DN	PETROVA_PROX1_TARGETS_DN		62	-0,5817184	-1,8950384	0	0,002740541	0,17	5960	tags=61%, list=30%, signal=87%
WELCSH_BRCA1_TARGETS_UP	WELCSH_BRCA1_TARGETS_UP		193	-0,5022806	-1,8949561	0	0,002706707	0,17	5746	tags=54%, list=29%, signal=75%
MORI_MATURE_B_LYMPHOCYTE_UP	MORI_MATURE_B_LYMPHOCYTE_UP		80	-0,55510676	-1,8939835	0	0,002728812	0,173	5084	tags=58%, list=25%, signal=77%
MIYAGAWA_TARGETS_OF_EWSR1_ETS_FUSIONS_DN	MIYAGAWA_TARGETS_OF_EWSR1_ETS_FUSIONS_DN		199	-0,49976283	-1,8937505	0	0,002709587	0,174	6217	tags=57%, list=31%, signal=82%
CHUNG_BLISTER_CYTOTOXICITY_DN	CHUNG_BLISTER_CYTOTOXICITY_DN		39	-0,6310876	-1,8928493	0	0,002731045	0,178	3373	tags=62%, list=17%, signal=74%
JI_RESPONSE_TO_FSH_DN	JI_RESPONSE_TO_FSH_DN		57	-0,5993485	-1,8917786	0	0,002738652	0,18	4761	tags=56%, list=24%, signal=73%
GRABARCZYK_BCL11B_TARGETS_UP	GRABARCZYK_BCL11B_TARGETS_UP		72	-0,5609937	-1,8881564	0	0,002917202	0,192	1839	tags=38%, list=9%, signal=41%
RUIZ_TNC_TARGETS_UP	RUIZ_TNC_TARGETS_UP		145	-0,5109991	-1,8849689	0	0,003064956	0,2	5893	tags=58%, list=29%, signal=82%
TONKS_TARGETS_OF_RUNX1_RUNX1T1_FUSION_MONOCYTE_DN	TONKS_TARGETS_OF_RUNX1_RUNX1T1_FUSION_MONOCYTE_DN		51	-0,59507614	-1,8827041	0	0,003107027	0,206	5424	tags=69%, list=27%, signal=94%
WILSON_PROTEASES_AT_TUMOR_BONE_INTERFACE_UP	WILSON_PROTEASES_AT_TUMOR_BONE_INTERFACE_UP		21	-0,71382695	-1,8819965	0	0,00311041	0,206	3497	tags=62%, list=17%, signal=75%
STEIN_ESRRA_TARGETS_RESPONSIVE_TO_ESTROGEN_DN	STEIN_ESRRA_TARGETS_RESPONSIVE_TO_ESTROGEN_DN		39	-0,6229873	-1,8791674	0	0,003288741	0,218	3547	tags=54%, list=18%, signal=65%
DING_LUNG_CANCER_EXPRESSION_BY_COPY_NUMBER	DING_LUNG_CANCER_EXPRESSION_BY_COPY_NUMBER		97	-0,54003227	-1,8663024	0	0,004122701	0,266	3897	tags=46%, list=19%, signal=57%
HOFMANN_CELL_LYMPHOMA_UP	HOFMANN_CELL_LYMPHOMA_UP		49	-0,5896451	-1,8648105	0	0,004114884	0,268	4648	tags=53%, list=23%, signal=69%
SENGUPTA_NASOPHARYNGEAL_CARCINOMA_WITH_LMP1_UP	SENGUPTA_NASOPHARYNGEAL_CARCINOMA_WITH_LMP1_UP		377	-0,46419442	-1,8629758	0	0,004265014	0,279	5432	tags=46%, list=27%, signal=63%
WORSCHECH_TUMOR_EVASION_AND_TOLEROGENICITY_UP	WORSCHECH_TUMOR_EVASION_AND_TOLEROGENICITY_UP		30	-0,6536683	-1,8618824	0,001574803	0,004255646	0,28	5857	tags=60%, list=29%, signal=85%
MARTORIATI_MDM4_TARGETS_FETAL_LIVER_DN	MARTORIATI_MDM4_TARGETS_FETAL_LIVER_DN		498	-0,45567223	-1,8593552	0	0,004306478	0,286	4727	tags=44%, list=24%, signal=56%
TAKEDA_TARGETS_OF_NUP98_HOXA9_FUSION_8D_DN	TAKEDA_TARGETS_OF_NUP98_HOXA9_FUSION_8D_DN		193	-0,4926255	-1,8577619	0	0,004344431	0,292	6137	tags=60%, list=31%, signal=86%
VILIMAS_NOTCH1_TARGETS_DN	VILIMAS_NOTCH1_TARGETS_DN		20	-0,71959203	-1,8517708	0,00331675	0,004811246	0,322	4052	tags=60%, list=20%, signal=75%
PETROVA_ENDOTHELIUM_LYMPHATIC_VS_BLOOD_UP	PETROVA_ENDOTHELIUM_LYMPHATIC_VS_BLOOD_UP		127	-0,5118776	-1,8501577	0	0,004934683	0,331	5827	tags=54%, list=29%, signal=76%
VILIMAS_NOTCH1_TARGETS_UP	VILIMAS_NOTCH1_TARGETS_UP		52	-0,5830118	-1,8443271	0	0,00545588	0,365	6428	tags=65%, list=32%, signal=96%
TIEN_INTESTINE_PROBIOTICS_2HR_DN	TIEN_INTESTINE_PROBIOTICS_2HR_DN		84	-0,5356827	-1,8372502	0	0,006238363	0,409	5216	tags=57%, list=26%, signal=77%
LU_EZH2_TARGETS_DN	LU_EZH2_TARGETS_DN		357	-0,45903698	-1,8328103	0	0,006556818	0,431	5780	tags=53%, list=29%, signal=73%
ONDER_CDH1_TARGETS_1_DN	ONDER_CDH1_TARGETS_1_DN		160	-0,49127808	-1,8309424	0	0,00677038	0,445	3888	tags=37%, list=19%, signal=45%
LAIHO_COLORECTAL_CANCER_SERRATED_UP	LAIHO_COLORECTAL_CANCER_SERRATED_UP		106	-0,5170719	-1,8307644	0	0,006715771	0,446	3383	tags=43%, list=17%, signal=52%
WEI_MIR34A_TARGETS	WEI_MIR34A_TARGETS		142	-0,49696973	-1,830034	0	0,006672922	0,446	4684	tags=48%, list=23%, signal=62%
DACOSTA_UV_RESPONSE_VIA_ERCC3_XPCS_DN	DACOSTA_UV_RESPONSE_VIA_ERCC3_XPCS_DN		85	-0,53551984	-1,8277535	0	0,006856405	0,457	5952	tags=64%, list=30%, signal=90%
WILENSKY_RESPONSE_TO_DARAPLADIB	WILENSKY_RESPONSE_TO_DARAPLADIB		28	-0,65355027	-1,8276947	0	0,006813094	0,459	3357	tags=64%, list=17%, signal=77%
DURCHDEWALD_SKIN_CARCINOGENESIS_DN	DURCHDEWALD_SKIN_CARCINOGENESIS_DN		257	-0,47273445	-1,8266413	0	0,006854679	0,465	3697	tags=37%, list=18%, signal=45%
RIGGINS_TAMOXIFEN_RESISTANCE_DN	RIGGINS_TAMOXIFEN_RESISTANCE_DN		214	-0,47717485	-1,8257179	0	0,006937698	0,475	5780	tags=51%, list=29%, signal=72%
FRASOR_RESPONSE_TO_SERM_OR_FULVESTRANT_UP	FRASOR_RESPONSE_TO_SERM_OR_FULVESTRANT_UP		21	-0,70557106	-1,8228424	0,004901961	0,007226303	0,486	3894	tags=57%, list=19%, signal=71%
IZADPANAH_STEM_CELL_ADIPOSE_VS_BONE_DN	IZADPANAH_STEM_CELL_ADIPOSE_VS_BONE_DN		103	-0,5239548	-1,8227726	0	0,00716061	0,486	5594	tags=54%, list=28%, signal=75%
WONG_ENDMETRIUM_CANCER_DN	WONG_ENDMETRIUM_CANCER_DN		78	-0,5492793	-1,8224583	0	0,007157369	0,489	6065	tags=64%, list=30%, signal=92%
SHAFFER_IRF4_MULTIPLE_MYELOMA_PROGRAM	SHAFFER_IRF4_MULTIPLE_MYELOMA_PROGRAM		34	-0,6409831	-1,8220642	0	0,007113776	0,491	4321	tags=59%, list=22%, signal=75%
KUROZUMI_RESPONSE_TO_ONCOCYTIC_VIRUS_AND_CYCLIC_RGD	KUROZUMI_RESPONSE_TO_ONCOCYTIC_VIRUS_AND_CYCLIC_RGD		21	-0,71034366	-1,821982	0	0,00707095	0,491	3948	tags=71%, list=20%, signal=89%
PICCALUGA_ANGIOIMMUNOBLASTIC_LYMPHOMA_DN	PICCALUGA_ANGIOIMMUNOBLASTIC_LYMPHOMA_DN		127	-0,50636613	-1,8200202	0	0,007257346	0,503	4103	tags=47%, list=21%, signal=59%
MAYBURD_RESPONSE_TO_L663536_DN	MAYBURD_RESPONSE_TO_L663536_DN		51	-0,5731172	-1,8194355	0	0,007332226	0,508	3538	tags=45%, list=18%, signal=55%
EPPERT_PROGENITOR	EPPERT_PROGENITOR		126	-0,50172365	-1,8175459	0	0,007454474	0,519	4231	tags=47%, list=21%, signal=59%
DAIRKEE_TERT_TARGETS_DN	DAIRKEE_TERT_TARGETS_DN		113	-0,51067764	-1,8123386	0	0,008017802	0,545	6167	tags=61%, list=31%, signal=88%
RAGHAVACHARI_PLATELET_SPECIFIC_GENES	RAGHAVACHARI_PLATELET_SPECIFIC_GENES		69	-0,5451582	-1,8098233	0	0,008294425	0,565	4921	tags=52%, list=25%, signal=69%
SCHAEFFER_PROSTATE_DEVELOPMENT_48HR_DN	SCHAEFFER_PROSTATE_DEVELOPMENT_48HR_DN		405	-0,449466	-1,8055437	0	0,008813851	0,59	5859	tags=48%, list=29%, signal=66%
KANG_GIST_WITH_PDGFRA_UP	KANG_GIST_WITH_PDGFRA_UP		48	-0,570444	-1,8052269	0	0,00874981	0,59	5970	tags=63%, list=30%, signal=89%
DUTERTRE_ESTRADIOL_RESPONSE_6HR_DN	DUTERTRE_ESTRADIOL_RESPONSE_6HR_DN		90	-0,5202614	-1,8046538	0	0,008780147	0,597	4707	tags=43%, list=24%, signal=56%
ALTEMEIER_RESPONSE_TO_LPS_WITH_MECHANICAL_VENTILATION	ALTEMEIER_RESPONSE_TO_LPS_WITH_MECHANICAL_VENTILATION		127	-0,4977521	-1,8003709	0	0,009327523	0,62	6219	tags=58%, list=31%, signal=84%
WIELAND_UP_BY_HBV_INFECTION	WIELAND_UP_BY_HBV_INFECTION		86	-0,5225837	-1,7998497	0	0,009333883	0,625	4584	tags=50%, list=23%, signal=65%
TAKEDA_TARGETS_OF_NUP98_HOXA9_FUSION_10D_UP	TAKEDA_TARGETS_OF_NUP98_HOXA9_FUSION_10D_UP		179	-0,4776461	-1,7960236	0	0,009740904	0,643	6335	tags=56%, list=32%, signal=81%
SU_THYMUS	SU_THYMUS		20	-0,70781434	-1,7959828	0	0,00967194	0,644	4888	tags=85%, list=24%, signal=112%
GARGALOVIC_RESPONSE_TO_OXIDIZED_PHOSPHOLIPIDS_TURQUOISE_UP	GARGALOVIC_RESPONSE_TO_OXIDIZED_PHOSPHOLIPIDS_TURQUOISE_UP		74	-0,53081083	-1,7928629	0	0,010000886	0,655	4038	tags=49%, list=20%, signal=61%
CHANG_IMMORTALIZED_BY_HPV31_UP	CHANG_IMMORTALIZED_BY_HPV31_UP		75	-0,5284316	-1,792277	0	0,010037547	0,659	4091	tags=44%, list=20%, signal=55%
HERNANDEZ_ABERRANT_MITOSIS_BY_DOCETACEL_2NM_UP	HERNANDEZ_ABERRANT_MITOSIS_BY_DOCETACEL_2NM_UP		78	-0,5292877	-1,7919198	0	0,010012449	0,662	4221	tags=49%, list=21%, signal=62%
MARTINEZ_RESPONSE_TO_TRABECTEDIN_DN	MARTINEZ_RESPONSE_TO_TRABECTEDIN_DN		262	-0,4589004	-1,7911814	0	0,01002238	0,666	4331	tags=41%, list=22%, signal=51%
BROWNE_HCMV_INFECTION_4HR_UP	BROWNE_HCMV_INFECTION_4HR_UP		52	-0,55753595	-1,7900623	0	0,010093076	0,673	3623	tags=38%, list=18%, signal=47%
SENESE_HDAC1_AND_HDAC2_TARGETS_UP	SENESE_HDAC1_AND_HDAC2_TARGETS_UP		222	-0,46187475	-1,7900105	0	0,010024719	0,673	4825	tags=42%, list=24%, signal=55%
IKEDA_MIR1_TARGETS_UP	IKEDA_MIR1_TARGETS_UP		53	-0,5631765	-1,7864715	0	0,010445708	0,689	3278	tags=45%, list=16%, signal=54%
RUTELLA_RESPONSE_TO_HGF_UP	RUTELLA_RESPONSE_TO_HGF_UP		400	-0,4431697	-1,786037	0	0,010486722	0,692	5428	tags=49%, list=27%, signal=66%

BIDUS_METASTASIS_UP	BIDUS_METASTASIS_UP	207	-0,46950707	-1,7849591	0	0,010627254	0,702	4846	tags=47%, list=24%, signal=62%
LEE_RECENT_THYMIC_EMIGRANT	LEE_RECENT_THYMIC_EMIGRANT	206	-0,46862516	-1,7849433	0	0,010548533	0,702	5311	tags=49%, list=27%, signal=66%
PECE_MAMMARY_STEM_CELL_DN	PECE_MAMMARY_STEM_CELL_DN	131	-0,49114558	-1,7836385	0	0,01061254	0,706	6328	tags=62%, list=32%, signal=90%
MIKKELSEN_MCV6_ICP_WITH_H3K27ME3	MIKKELSEN_MCV6_ICP_WITH_H3K27ME3	72	-0,5257402	-1,7835758	0	0,010543438	0,706	6055	tags=38%, list=30%, signal=54%
DUTERTRE_ESTRADIOL_RESPONSE_24HR_DN	DUTERTRE_ESTRADIOL_RESPONSE_24HR_DN	488	-0,43497646	-1,78089	0	0,010918035	0,721	6085	tags=51%, list=30%, signal=72%
LIU_PROSTATE_CANCER_DN	LIU_PROSTATE_CANCER_DN	449	-0,4356195	-1,7804332	0	0,010953059	0,728	6588	tags=57%, list=33%, signal=83%
XU_GH1_EXOGENOUS_TARGETS_DN	XU_GH1_EXOGENOUS_TARGETS_DN	115	-0,48900837	-1,7801248	0	0,010915203	0,729	4097	tags=37%, list=20%, signal=47%
FINETTI_BREAST_CANCERS_KINOME_GRAY	FINETTI_BREAST_CANCERS_KINOME_GRAY	15	-0,73547053	-1,7800376	0,00317965	0,01083779	0,729	2857	tags=67%, list=14%, signal=78%
ZHANG_BREAST_CANCER_PROGENITORS_UP	ZHANG_BREAST_CANCER_PROGENITORS_UP	397	-0,4406051	-1,7773467	0	0,011046256	0,739	4282	tags=38%, list=21%, signal=47%
KUROZUMI_RESPONSE_TO_ONCOCYTIC_VIRUS	KUROZUMI_RESPONSE_TO_ONCOCYTIC_VIRUS	44	-0,5766129	-1,7772379	0	0,010984981	0,739	5275	tags=59%, list=26%, signal=80%
KONDO_EZH2_TARGETS	KONDO_EZH2_TARGETS	220	-0,46315387	-1,7770106	0	0,010924601	0,739	6856	tags=56%, list=34%, signal=85%
IGARASHI_ATF4_TARGETS_DN	IGARASHI_ATF4_TARGETS_DN	88	-0,5131117	-1,775381	0	0,011122059	0,746	6164	tags=59%, list=31%, signal=85%
VERNELL_RETINOBLASTOMA_PATHWAY_UP	VERNELL_RETINOBLASTOMA_PATHWAY_UP	68	-0,531123	-1,7743222	0	0,011209032	0,751	5228	tags=54%, list=26%, signal=73%
FLECHNER_BIOPSY_KIDNEY_TRANSPLANT_REJECTED_VS_OK_UP	FLECHNER_BIOPSY_KIDNEY_TRANSPLANT_REJECTED_VS_OK_UP	84	-0,52313805	-1,7743014	0	0,01114037	0,751	5161	tags=58%, list=26%, signal=78%
VART_KSHV_INFECTION_ANGIOGENIC_MARKERS_UP	VART_KSHV_INFECTION_ANGIOGENIC_MARKERS_UP	164	-0,47444373	-1,7735674	0	0,011210439	0,753	4733	tags=45%, list=24%, signal=59%
ZHENG_FOXP3_TARGETS_UP	ZHENG_FOXP3_TARGETS_UP	26	-0,64617735	-1,7703328	0	0,011581755	0,765	5880	tags=85%, list=29%, signal=120%
PLASARI_NFIC_TARGETS_BASAL_DN	PLASARI_NFIC_TARGETS_BASAL_DN	18	-0,69647175	-1,7656329	0,003361345	0,012203818	0,793	3703	tags=61%, list=19%, signal=75%
GAURNIER_PSMD4_TARGETS	GAURNIER_PSMD4_TARGETS	52	-0,55328304	-1,758739	0	0,01341517	0,819	5969	tags=62%, list=30%, signal=87%
PAPASPYRIDONOS_UNSTABLE_ATEROSCLEROTIC_PLAQUE_DN	PAPASPYRIDONOS_UNSTABLE_ATEROSCLEROTIC_PLAQUE_DN	42	-0,5712707	-1,7572724	0	0,01360123	0,823	7023	tags=79%, list=35%, signal=121%
ENK_UV_RESPONSE_KERATINOCYTE_DN	ENK_UV_RESPONSE_KERATINOCYTE_DN	481	-0,42764503	-1,7570916	0	0,013542124	0,824	5171	tags=44%, list=26%, signal=58%
IGLESIAS_E2F_TARGETS_UP	IGLESIAS_E2F_TARGETS_UP	147	-0,47829702	-1,7565928	0	0,013564522	0,826	6020	tags=59%, list=30%, signal=84%
GUTIERREZ_CHRONIC_LYMPHOCYTIC_LEUKEMIA_UP	GUTIERREZ_CHRONIC_LYMPHOCYTIC_LEUKEMIA_UP	11	-0,7930824	-1,756405	0	0,013506275	0,826	2859	tags=64%, list=14%, signal=74%
RIGGI_EWING_SARCOMA_PROGENITOR_UP	RIGGI_EWING_SARCOMA_PROGENITOR_UP	401	-0,43657672	-1,7553446	0	0,013686745	0,832	7070	tags=55%, list=35%, signal=84%
DAVIES_MULTIPLE_MYELOMA_VS_MGUS_DN	DAVIES_MULTIPLE_MYELOMA_VS_MGUS_DN	26	-0,64294505	-1,7546679	0,001607717	0,013649978	0,833	2843	tags=46%, list=14%, signal=54%
LI_WILMS_TUMOR_VS_FETAL_KIDNEY_1_UP	LI_WILMS_TUMOR_VS_FETAL_KIDNEY_1_UP	180	-0,46651086	-1,7529068	0	0,014006727	0,844	6008	tags=61%, list=30%, signal=87%
PLASARI_TGFB1_SIGNALING_VIA_NFIC_1HR_DN	PLASARI_TGFB1_SIGNALING_VIA_NFIC_1HR_DN	104	-0,49736992	-1,7527719	0	0,013954049	0,844	4744	tags=42%, list=24%, signal=55%
SEITZ_NEOPLASTIC_TRANSFORMATION_BY_8P_DELETION_UP	SEITZ_NEOPLASTIC_TRANSFORMATION_BY_8P_DELETION_UP	73	-0,5248939	-1,7513032	0,001447178	0,014127788	0,851	6311	tags=56%, list=32%, signal=82%
EPPERT_HSC_R	EPPERT_HSC_R	113	-0,4899527	-1,7470738	0	0,014958994	0,87	4648	tags=48%, list=23%, signal=62%
GENTLES_LEUKEMIC_STEM_CELL_UP	GENTLES_LEUKEMIC_STEM_CELL_UP	26	-0,6467346	-1,7461777	0,004769475	0,015083469	0,877	5763	tags=69%, list=29%, signal=97%
GOLDRATH_IMMUNE_MEMORY	GOLDRATH_IMMUNE_MEMORY	63	-0,53219885	-1,7461035	0	0,014997784	0,877	5032	tags=54%, list=25%, signal=72%
TURASHVILI_BREAST_DUCTAL_CARCINOMA_VS_LOBULAR_NORMAL_UP	TURASHVILI_BREAST_DUCTAL_CARCINOMA_VS_LOBULAR_NORMAL_UP	71	-0,52343255	-1,7432761	0	0,015616301	0,886	6029	tags=63%, list=30%, signal=90%
GAVIN_FOXP3_TARGETS_CLUSTER_T4	GAVIN_FOXP3_TARGETS_CLUSTER_T4	90	-0,50294584	-1,7428937	0	0,015562565	0,889	3876	tags=46%, list=19%, signal=56%
DELYS_THYROID_CANCER_DN	DELYS_THYROID_CANCER_DN	227	-0,44799587	-1,7394412	0	0,01625111	0,898	5837	tags=50%, list=29%, signal=70%
WANG_RESPONSE_TO_FORSKOLIN_DN	WANG_RESPONSE_TO_FORSKOLIN_DN	8	-0,8548288	-1,735573	0,001697793	0,017074054	0,91	2210	tags=75%, list=11%, signal=84%
LIEN_BREAST_CARCINOMA_METAPLASTIC	LIEN_BREAST_CARCINOMA_METAPLASTIC	35	-0,5883765	-1,7349995	0,001569859	0,017066192	0,912	5487	tags=63%, list=27%, signal=86%
GEORGANTAS_HSC_MARKERS	GEORGANTAS_HSC_MARKERS	64	-0,5312414	-1,7339352	0	0,017260464	0,915	5232	tags=47%, list=26%, signal=63%
BROWNE_HCMV_INFECTION_16HR_UP	BROWNE_HCMV_INFECTION_16HR_UP	217	-0,45448032	-1,7329233	0	0,017444381	0,92	4038	tags=41%, list=20%, signal=51%
DAZARD_RESPONSE_TO_UV_SCC_UP	DAZARD_RESPONSE_TO_UV_SCC_UP	115	-0,48124516	-1,732592	0	0,017428473	0,921	3857	tags=40%, list=19%, signal=49%
CHEN_ETV5_TARGETS_SERTOLI	CHEN_ETV5_TARGETS_SERTOLI	19	-0,67800605	-1,730078	0,004823151	0,018056044	0,932	5838	tags=84%, list=29%, signal=119%
FOSTER_TOLERANT_MACROPHAGE_DN	FOSTER_TOLERANT_MACROPHAGE_DN	393	-0,42791912	-1,7299471	0	0,017991181	0,933	5348	tags=46%, list=27%, signal=62%
BROWN_MYELOID_CELL_DEVELOPMENT_UP	BROWN_MYELOID_CELL_DEVELOPMENT_UP	155	-0,4633024	-1,7269853	0	0,018672895	0,939	4360	tags=43%, list=22%, signal=55%
YAMAZAKI_TCEB3_TARGETS_UP	YAMAZAKI_TCEB3_TARGETS_UP	171	-0,46130416	-1,7256227	0	0,018876731	0,944	4918	tags=47%, list=25%, signal=62%
BOQUEST_STEM_CELL_DN	BOQUEST_STEM_CELL_DN	200	-0,45616663	-1,7229097	0	0,019464262	0,95	4928	tags=49%, list=25%, signal=64%
KANG_DOXORUBICIN_RESISTANCE_DN	KANG_DOXORUBICIN_RESISTANCE_DN	18	-0,67866534	-1,7214762	0,003164557	0,01978814	0,954	5305	tags=78%, list=27%, signal=106%
PYEON_CANCER_HEAD_AND_NECK_VS_CERVICAL_UP	PYEON_CANCER_HEAD_AND_NECK_VS_CERVICAL_UP	174	-0,46109587	-1,7193582	0	0,020298526	0,958	4587	tags=41%, list=23%, signal=53%
IKEDA_MIR133_TARGETS_UP	IKEDA_MIR133_TARGETS_UP	43	-0,5591197	-1,7185987	0	0,020380633	0,959	4377	tags=58%, list=22%, signal=74%
GEORGES_CELL_CYCLE_MIR192_TARGETS	GEORGES_CELL_CYCLE_MIR192_TARGETS	61	-0,52650934	-1,7184994	0,001416431	0,020299051	0,959	3687	tags=36%, list=18%, signal=44%
BOQUEST_STEM_CELL_CULTURED_VS_FRESH_UP	BOQUEST_STEM_CELL_CULTURED_VS_FRESH_UP	409	-0,42965615	-1,7180265	0	0,020336764	0,959	7364	tags=62%, list=37%, signal=96%
WIERENGA_STAT5A_TARGETS_GROUP2	WIERENGA_STAT5A_TARGETS_GROUP2	56	-0,5413043	-1,715583	0,001531394	0,020901706	0,963	6368	tags=68%, list=32%, signal=99%
HAHTOLA_SEZARY_SYNDROM_DN	HAHTOLA_SEZARY_SYNDROM_DN	38	-0,5682201	-1,7152895	0,001526718	0,020830655	0,964	7209	tags=68%, list=36%, signal=107%
LIU_TARGETS_OF_VMYB_VS_CMYB_DN	LIU_TARGETS_OF_VMYB_VS_CMYB_DN	42	-0,56277376	-1,7151648	0,002971768	0,02074196	0,964	6417	tags=64%, list=32%, signal=94%
CHYLA_CBFA2T3_TARGETS_UP	CHYLA_CBFA2T3_TARGETS_UP	359	-0,42753622	-1,7137457	0	0,020965539	0,964	4914	tags=43%, list=25%, signal=56%
CHEN_LVAD_SUPPORT_OF_FAILING_HEART_DN	CHEN_LVAD_SUPPORT_OF_FAILING_HEART_DN	41	-0,56197083	-1,7131295	0,001474926	0,021035679	0,964	5132	tags=54%, list=26%, signal=72%
YAGI_AML_FAB_MARKERS	YAGI_AML_FAB_MARKERS	187	-0,4505258	-1,7118771	0	0,021363681	0,966	6423	tags=57%, list=32%, signal=83%
CHEBOTAEV_GR_TARGETS_UP	CHEBOTAEV_GR_TARGETS_UP	73	-0,5051097	-1,7111795	0	0,02143049	0,967	4621	tags=44%, list=23%, signal=57%
ABDELMOHSEN_ELAVL4_TARGETS	ABDELMOHSEN_ELAVL4_TARGETS	15	-0,69309217	-1,7109576	0,003344482	0,021389011	0,967	4373	tags=73%, list=22%, signal=94%
GRADE_COLON_CANCER_DN	GRADE_COLON_CANCER_DN	31	-0,60360146	-1,7064798	0,001560062	0,02277911	0,971	5811	tags=61%, list=29%, signal=86%
WATANABE_ULCERATIVE_COLITIS_WITH_CANCER_DN	WATANABE_ULCERATIVE_COLITIS_WITH_CANCER_DN	14	-0,7199552	-1,705783	0,003194888	0,022890287	0,972	4280	tags=79%, list=21%, signal=100%
SESTO_RESPONSE_TO_UV_C2	SESTO_RESPONSE_TO_UV_C2	53	-0,5360482	-1,7057235	0	0,02280081	0,973	4612	tags=55%, list=23%, signal=71%
FLECHNER_PBL_KIDNEY_TRANSPLANT_OK_VS_DONOR_DN	FLECHNER_PBL_KIDNEY_TRANSPLANT_OK_VS_DONOR_DN	41	-0,5702061	-1,7026136	0	0,023724841	0,981	3686	tags=51%, list=18%, signal=63%
HOWLIN_PUBERTAL_MAMMARY_GLAND	HOWLIN_PUBERTAL_MAMMARY_GLAND	66	-0,51805484	-1,7017628	0	0,023888394	0,981	5368	tags=48%, list=27%, signal=66%
GRANDVAUX_IRF3_TARGETS_DN	GRANDVAUX_IRF3_TARGETS_DN	19	-0,6599694	-1,7012962	0,003333333	0,023899224	0,981	3939	tags=63%, list=20%, signal=79%
HESS_TARGETS_OF_HOXA9_AND_MEIS1_DN	HESS_TARGETS_OF_HOXA9_AND_MEIS1_DN	76	-0,5054978	-1,7006681	0,001455604	0,024013363	0,981	5122	tags=51%, list=26%, signal=69%
BERTUCCI_MEDULLARY_VS_DUCTAL_BREAST_CANCER_DN	BERTUCCI_MEDULLARY_VS_DUCTAL_BREAST_CANCER_DN	159	-0,45742893	-1,6994288	0	0,024304774	0,981	6665	tags=60%, list=33%, signal=90%
TURASHVILI_BREAST_DUCTAL_CARCINOMA_VS_DUCTAL_NORMAL_UP	TURASHVILI_BREAST_DUCTAL_CARCINOMA_VS_DUCTAL_NORMAL_UP	43	-0,5525976	-1,6990556	0,004424779	0,024255998	0,982	6779	tags=72%, list=34%, signal=109%
PETRETTO_CARDIAC_HYPERTROPHY	PETRETTO_CARDIAC_HYPERTROPHY	33	-0,58707446	-1,6987321	0,001517451	0,024236552	0,983	5549	tags=67%, list=28%, signal=92%
ROETH_TERT_TARGETS_DN	ROETH_TERT_TARGETS_DN	8	-0,8305323	-1,6977853	0,005235602	0,024414333	0,983	2819	tags=88%, list=14%, signal=102%
GAVIN_FOXP3_TARGETS_CLUSTER_P4	GAVIN_FOXP3_TARGETS_CLUSTER_P4	96	-0,48304674	-1,6971382	0	0,02453498	0,984	4265	tags=44%, list=21%, signal=55%
BOYLAN_MULTIPLE_MYELOMA_PCA1_UP	BOYLAN_MULTIPLE_MYELOMA_PCA1_UP	97	-0,47974196	-1,6969603	0	0,024458192	0,984	5767	tags=53%, list=29%, signal=74%
LINDSTEDT_DENDRITIC_CELL_MATURATION_A	LINDSTEDT_DENDRITIC_CELL_MATURATION_A	66	-0,5191287	-1,6968404	0,001490313	0,024376733	0,984	5767	tags=59%, list=29%, signal=83%
CARD_MIR302A_TARGETS	CARD_MIR302A_TARGETS	77	-0,50372964	-1,6956905	0	0,024677811	0,984	5030	tags=48%, list=25%, signal=64%
RUTELLA_RESPONSE_TO_HGF_VS_CSF2RB_AND_IL4_UP	RUTELLA_RESPONSE_TO_HGF_VS_CSF2RB_AND_IL4_UP	389	-0,422025	-1,6953375	0	0,02463473	0,984	5196	tags=48%, list=26%, signal=63%
CROMER_METASTASIS_UP	CROMER_METASTASIS_UP	75	-0,501774	-1,6947619	0	0,024668185	0,984	4422	tags=45%, list=22%, signal=58%
IVANOVA_HEMATOPOIESIS_LATE_PROGENITOR	IVANOVA_HEMATOPOIESIS_LATE_PROGENITOR	497	-0,41448998	-1,6944854	0	0,024658157	0,985	5676	tags=46%, list=28%, signal=63%
AZARE_NEOPLASTIC_TRANSFORMATION_BY_STAT3_UP	AZARE_NEOPLASTIC_TRANSFORMATION_BY_STAT3_UP	117	-0,4757749	-1,6932822	0	0,024946803	0,988	5572	tags=48%, list=28%, signal=66%
BORCZUK_MALIGNANT_MESOTHELIOMA_UP	BORCZUK_MALIGNANT_MESOTHELIOMA_UP	295	-0,42876983	-1,6930945	0	0,024892308	0,988	4496	tags=39%, list=22%, signal=50%
BURTON_ADIPOGENESIS_12	BURTON_ADIPOGENESIS_12	30	-0,59983355	-1,6928964	0,004643963	0,024848782	0,988	4032	tags=57%, list=20%, signal=71%
ROY_WOUND_BLOOD_VESSEL_UP	ROY_WOUND_BLOOD_VESSEL_UP	49	-0,5432893	-1,691074	0,003095975	0,025266584	0,991	4603	tags=57%, list=23%, signal=74%
CHEMELLO_SOLEUS_VS_EDL_MYOFIBERS_DN	CHEMELLO_SOLEUS_VS_EDL_MYOFIBERS_DN	19	-0,664339	-1,6898122	0,003231018	0,02552552	0,992	5377	tags=58%, list=27%, signal=79%
IIZUKA_LIVER_CANCER_PROGRESSION_L1_G1_UP	IIZUKA_LIVER_CANCER_PROGRESSION_L1_G1_UP	24	-0,62686557	-1,6896929	0,004830918	0,02545919	0,992	5528	tags=58%, list=28%, signal=81%
FINETTI_BREAST_CANCER_KINOME_GREEN	FINETTI_BREAST_CANCER_KINOME_GREEN	16	-0,6858288	-1,6878796	0,006779661	0,025967984	0,992	3343	tags=63%, list=17%, signal=75%
REN_ALVEOLAR_RHABDOMYOSARCOMA_DN	REN_ALVEOLAR_RHABDOMYOSARCOMA_DN	400	-0,42083836	-1,6870881	0	0,02608293	0,992	5294	tags=48%, list=26%, signal=64%
TONKS_TARGETS_OF_RUNX1_RUNX1T1_FUSION_HSC_UP	TONKS_TARGETS_OF_RUNX1_RUNX1T1_FUSION_HSC_UP	180	-0,45110267	-1,6866533	0	0,026066598	0,992	3350	tags=33%, list=17%, signal=39%
BROWNE_HCMV_INFECTION_12HR_UP	BROWNE_HCMV_INFECTION_12HR_UP	101	-0,48052227	-1,6865999	0	0,025982983	0,992	3832	tags=38%, list=19%, signal=46%
WANG_RESPONSE_TO_ANDROGEN_UP	WANG_RESPONSE_TO_ANDROGEN_UP	28	-0,58968437	-1,6864719	0,006420546	0,0259158	0,992	3703	tags=50%, list=19%, signal=61%
BROWNE_HCMV_INFECTION_18HR_UP	BROWNE_HCMV_INFECTION_18HR_UP	172	-0,4519612	-1,6856762	0	0,026055949	0,992	5466	tags=48%, list=27%, signal=66%
GAUSSMANN_MLL_AF4_FUSION_TARGETS_A_UP	GAUSSMANN_MLL_AF4_FUSION_TARGETS_A_UP	176	-0,44867185	-1,684857	0	0,02625604	0,993	4852	tags=41%, list=24%, signal=54%
LENAOUR_DENDRITIC_CELL_MATURATION_DN	LENAOUR_DENDRITIC_CELL_MATURATION_DN	127	-0,46890327	-1,6844559	0	0,026208825	0,993	5593	tags=46%, list=28%, signal=64%
TAKEDA_TARGETS_OF_NUP98_HOXA9_FUSION_8D_UP	TAKEDA_TARGETS_OF_NUP98_HOXA9_FUSION_8D_UP	147	-0,457619	-1,6838629	0	0,02623269	0,993	6361	tags=54%, list=32%, signal=78%
AKL_HTLV1_INFECTION_DN	AKL_HTLV1_INFECTION_DN	65	-0,5139526	-1,6825985	0	0,026566178	0,994	5026	tags=52%, list=25%, signal=70%
ALONSO_METASTASIS_EMT_UP	ALONSO_METASTASIS_EMT_UP	36	-0,56641984	-1,6824201	0,001540832	0,026502905	0,994	4049	tags=44%, list=20%, signal=56%
BALDWIN_PRKCI_TARGETS_UP	BALDWIN_PRKCI_TARGETS_UP	33	-0,57333654	-1,6823553	0,009756098	0,02640013	0,994	4001	tags=52%, list=20%, signal=64%
GARGALOVIC_RESPONSE_TO_OXIDIZED_PHOSPHOLIPIDS_GREEN_UP	GARGALOVIC_RESPONSE_TO_OXIDIZED_PHOSPHOLIPIDS_GREEN_UP	23	-0,62541467	-1,6820345	0,003081664	0,026372725	0,994	4034	tags=61%, list=20%, signal=76%
GENTILE_UV_HIGH_DOSE_DN	GENTILE_UV_HIGH_DOSE_DN	302	-0,4287142	-1,6807138	0	0,026709942	0,995	6130	tags=54%, list=31%, signal=76%
ROZANOV_MMP14_TARGETS_SUBSET	ROZANOV_MMP14_TARGETS_SUBSET	31	-0,5928084	-1,6791153	0,001594896	0,02718731	0,996	4603	tags=61%, list=23%, signal=79%
DEMAGALHAES_AGING_UP	DEMAGALHAES_AGING_UP	52	-0,53284323	-1,6781654	0,002980626	0,027384015	0,996	6499	tags=67%, list=32%, signal=99%
GENTILE_UV_RESPONSE_CLUSTER_D4	GENTILE_UV_RESPONSE_CLUSTER_D4	54	-0,52297586	-1,6778302	0,006051437	0,027377801	0,996	3695	tags=43%, list=18%, signal=52%
PASINI_SUZ12_TARGETS_DN	PASINI_SUZ12_TARGETS_DN	310	-0,42314777	-1,677251	0	0,027488625	0,996	6336	tags=55%, list=32%, signal=79%
VERHAAK_GLIOBLASTOMA_MESENCHYMAL	VERHAAK_GLIOBLASTOMA_MESENCHYMAL	212	-0,43736535	-1,677138	0	0,027414098	0,996	5541	tags=51%, list=28%, signal=70%
NAGASHIMA_NRG1_SIGNALING_DN	NAGASHIMA_NRG1_SIGNALING_DN	54	-0,5317995	-1,6766112	0,006024096	0,027447233	0,996	5184	tags=48%, list=26%, signal=65%
WAMUNYOKOLI_OVARIAN_CANCER_GRADES_1_2_DN	WAMUNYOKOLI_OVARIAN_CANCER_GRADES_1_2_DN	63	-0,5133409	-1,6760594	0,003012048	0,027537316	0,997	5987	tags=56%, list=30%, signal=79%
NEMETH_INFLAMMATORY_RESPONSE_LPS_DN	NEMETH_INFLAMMATORY_RESPONSE_LPS_DN	31	-0,5834622	-1,6755036	0,001579779	0,027612554	0,997	3398	tags=42%, list=17%, signal=50%
SMID_BREAST_CANCER_LUMINAL_A_UP	SMID_BREAST_CANCER_LUMINAL_A_UP	78	-0,49687564	-1,6701341	0	0,029521106	0,997	8079	tags=69%, list=40%, signal=116%
BOYLAN_MULTIPLE_MYELOMA_C_D_DN	BOYLAN_MULTIPLE_MYELOMA_C_D_DN	239	-0,43374783	-1,6698046	0	0,029496651	0,997	5914	tags=53%, list=30%, signal=75%
ZHAN_MULTIPLE_MYELOMA_CD1_VS_CD2_DN	ZHAN_MULTIPLE_MYELOMA_CD1_VS_CD2_DN	46	-0,5442875	-1,669312	0,001485884	0,029543811	0,997	6551	tags=76%, list=33%, signal=113%
MARSON_FOXP3_TARGETS_STIMULATED_UP	MARSON_FOXP3_TARGETS_STIMULATED_UP	28	-0,5883984	-1,6668173	0,006259781	0,030436084	0,997	4970	tags=61%, list=25%, signal=81%
RIGGI_EWING_SARCOMA_PROGENITOR_DN	RIGGI_EWING_SARCOMA_PROGENITOR_DN	171	-0,44407985	-1,6653179	0	0,030856265	0,997	6089	tags=56%, list=30%, signal=79%
DASU_IL6_SIGNALING_SCAR_DN	DASU_IL6_SIGNALING_SCAR_DN	16	-0,68253136	-1,6638722	0,003338898	0,031313572	0,998	5759	tags=75%, list=29%, signal=105%
ZHAN_MULTIPLE_MYELOMA_CD1_AND_CD2_UP	ZHAN_MULTIPLE_MYELOMA_CD1_AND_CD2_UP	81	-0,4907027	-1,6633177	0,00141844	0,031351753	0,998	4117	tags=46%, list=21%, signal=57%
SUNG_METASTASIS_STROMA_UP	SUNG_METASTASIS_STROMA_UP	106	-0,46642393	-1,6629896	0	0,0313805	0,998	4721	tags=45%, list=24%, signal=59%
DACOSTA_UV_RESPONSE_VIA_ERCC3_TTD_DN	DACOSTA_UV_RESPONSE_VIA_ERCC3_TTD_DN	81	-0,4911839	-1,6613578	0,00286533	0,03188151	0,998	4628	tags=49%, list=23%, signal=64%
GAVIN_FOXP3_TARGETS_CLUSTER_P7	GAVIN_FOXP3_TARGETS_CLUSTER_P7	84	-0,48650253	-1,6597433	0,002853067	0,03231479	0,998	6393	tags=61%, list=32%, signal=89%
LIM_MAMMARY_LUMINAL_MATURE_DN	LIM_MAMMARY_LUMINAL_MATURE_DN	96	-0,47621942	-1,6593953	0	0,03236737	0,998	5122	tags=50%, list=26%, signal=67%
DURAND_STROMA_S_UP	DURAND_STROMA_S_UP	279	-0,4229565	-1,659369	0	0,032245535	0,998	7247	tags=63%, list=36%, signal=97%
GAVIN_FOXP3_TARGETS_CLUSTER_P3	GAVIN_FOXP3_TARGETS_CLUSTER_P3	152	-0,445675	-1,6589671	0	0,032243513	0,998	5965	tags=53%, list=30%, signal=74%
VALK_AML_CLUSTER_3	VALK_AML_CLUSTER_3	31	-0,5765181	-1,6588436	0,004761905	0,032141283	0,998	5261	tags=61%, list=26%, signal=83%
TORCHIA_TARGETS_OF_EWSR1_FLI1_FUSION_DN	TORCHIA_TARGETS_OF_EWSR1_FLI1_FUSION_DN	296	-0,4197781	-1,6559854	0	0,033291392	0,998	4118	tags=39%, list=21%, signal=48%
LEE_LIVER_CANCER_MYC_E2F1_DN	LEE_LIVER_CANCER_MYC_E2F1_DN	62	-0,5126126	-1,6553152	0,003053435	0,0334105	0,998	3410	tags=26%, list=17%, signal=31%
NIKOLSKY_BREAST_CANCER_8P12_P11_AMPLICON	NIKOLSKY_BREAST_CANCER_8P12_P11_AMPLICON	48	-0,53073853	-1,6552953	0,001545595	0,033282373	0,998	4561	tags=42%, list=23%, signal=54%
ZWANG_CLASS_1_TRANSIENTLY_INDUCED_BY_EGF	ZWANG_CLASS_1_TRANSIENTLY_INDUCED_BY_EGF	476	-0,40577424	-1,6546401	0	0,033422768	0,998	5219	tags=37%, list=26%, signal=49%
NOUZOVA_METHYLATED_IN_APL	NOUZOVA_METHYLATED_IN_APL	56	-0,51227134	-1,652175	0	0,03428352	0,998	4277	tags=43%, list=21%, signal=54%
HENDRICKS_SMARCA4_TARGETS_DN	HENDRICKS_SMARCA4_TARGETS_DN	48	-0,52866465	-1,6514767	0,00877193	0,034419347	0,999	4216	tags=46%, list=21%, signal=58%
STEIN_ESR1_TARGETS	STEIN_ESR1_TARGETS	82	-0,48690793	-1,6498017	0,001404494	0,03492908	0,999	5147	tags=45%, list=26%, signal=61%
GINESTIER_BREAST_CANCER_20Q13_AMPLIFICATION_UP	GINESTIER_BREAST_CANCER_20Q13_AMPLIFICATION_UP	108	-0,4621279	-1,6488185	0	0,03518831	0,999	3218	tags=31%, list=16%, signal=36%
CROONQUIST_NRAS_VS_STROMAL_STIMULATION_UP	CROONQUIST_NRAS_VS_STROMAL_STIMULATION_UP	37	-0,56136394	-1,6453195	0,001557632	0,03662414	0,999	4857	tags=57%, list=24%, signal=75%
FUJII_YBX1_TARGETS_DN	FUJII_YBX1_TARGETS_DN	196	-0,4312583	-1,6444931	0	0,03687968	0,999	5651	tags=46%, list=28%, signal=64%
GOBERT_CORE_OLIGODENDROCYTE_DIFFERENTIATION	GOBERT_CORE_OLIGODENDROCYTE_DIFFERENTIATION	40	-0,5480223	-1,6439883	0,004615385	0,03698134	0,999	3020	tags=43%, list=15%, signal=50%
WENDT_COHESIN_TARGETS_UP	WENDT_COHESIN_TARGETS_UP	31	-0,5878991	-1,643573	0,003115265	0,037008423	0,999	5578	tags=71%, list=28%, signal=98%
CHARAFE_BREAST_CANCER_LUMINAL_VS_BASAL_DN	CHARAFE_BREAST_CANCER_LUMINAL_VS_BASAL_DN	429	-0,40622786	-1,6427978	0	0,037225287	0,999	5188	tags=44%, list=26%, signal=58%
HOSHIDA_LIVER_CANCER_SUBCLASS_S1	HOSHIDA_LIVER_CANCER_SUBCLASS_S1	228	-0,42450374	-1,6423588	0	0,037290208	0,999	6100	tags=57%, list=30%, signal=82%
HERNANDEZ_ABERRANT_MITOSIS_BY_DOCETACEL_4NM_UP	HERNANDEZ_ABERRANT_MITOSIS_BY_DOCETACEL_4NM_UP	22	-0,62338066	-1,6416811	0,004672897	0,03743932	0,999	4767	tags=50%, list=24%, signal=66%
AZARE_STAT3_TARGETS	AZARE_STAT3_TARGETS	23	-0,6043246	-1,6414931	0,009677419	0,037387338	0,999	645	tags=26%, list=3%, signal=27%
TAKEDA_TARGETS_OF_NUP98_HOXA9_FUSION_6HR_UP	TAKEDA_TARGETS_OF_NUP98_HOXA9_FUSION_6HR_UP	80	-0,48141477	-1,6404538	0	0,037713446	0,999	6084	tags=50%, list=30%, signal=72%
HECKER_IFNB1_TARGETS	HECKER_IFNB1_TARGETS	92	-0,4731391	-1,6398772	0,001390821	0,03785925	0,999	6036	tags=59%, list=30%, signal=84%
WILCOX_RESPONSE_TO_PROGESTERONE_UP	WILCOX_RESPONSE_TO_PROGESTERONE_UP	140	-0,4438996	-1,6386087	0	0,03822897	0,999	6383	tags=50%, list=32%, signal=73%
FINAK_BREAST_CANCER_SDPP_SIGNATURE	FINAK_BREAST_CANCER_SDPP_SIGNATURE	23	-0,6071383	-1,6384201	0,008	0,03813749	0,999	6484	tags=70%, list=32%, signal=103%
KHETCHOUMIAN_TRIM24_TARGETS_UP	KHETCHOUMIAN_TRIM24_TARGETS_UP	47	-0,5293805	-1,6382884	0,005891016	0,038050536	0,999	3062	tags=40%, list=15%, signal=48%
GENTILE_UV_RESPONSE_CLUSTER_D8	GENTILE_UV_RESPONSE_CLUSTER_D8	39	-0,55063266	-1,6380761	0,003021148	0,038010158	0,999	3778	tags=51%, list=19%, signal=63%
IKEDA_MIR30_TARGETS_DN	IKEDA_MIR30_TARGETS_DN	26	-0,5921099	-1,6380578	0,003159558	0,037887044	0,999	3546	tags=46%, list=18%, signal=56%

CREIGHTON_ENDOCRINE_THERAPY_RESISTANCE_5	CREIGHTON_ENDOCRINE_THERAPY_RESISTANCE_5	432	-0,40312603	-1,6376002	0	0,037959237	0,999	5549	tags=44%, list=28%, signal=59%
CHEBOTAEV_GR_TARGETS_DN	CHEBOTAEV_GR_TARGETS_DN	115	-0,45833036	-1,6375865	0,00131579	0,037820697	0,999	7253	tags=60%, list=36%, signal=94%
TARTE_PLASMA_CELL_VS_B_LYMPHOCYTE_DN	TARTE_PLASMA_CELL_VS_B_LYMPHOCYTE_DN	37	-0,5555021	-1,6374886	0,00621118	0,037723996	0,999	4132	tags=49%, list=21%, signal=61%
MOSERLE_IFNA_RESPONSE	MOSERLE_IFNA_RESPONSE	31	-0,5794249	-1,6363754	0,001620746	0,038053244	1	6311	tags=68%, list=32%, signal=99%
MARTINEZ_RESPONSE_TO_TRABECTEDIN	MARTINEZ_RESPONSE_TO_TRABECTEDIN	49	-0,5172413	-1,6351678	0,004643963	0,038392633	1	5131	tags=51%, list=26%, signal=68%
GARY_CD5_TARGETS_DN	GARY_CD5_TARGETS_DN	418	-0,4065923	-1,63428	0	0,038628343	1	4892	tags=41%, list=24%, signal=53%
ROSS_AML_WITH_CBFB_MYH11_FUSION	ROSS_AML_WITH_CBFB_MYH11_FUSION	51	-0,51430357	-1,6336936	0,001529052	0,038736757	1	3024	tags=43%, list=15%, signal=51%
TING_SILENCED_BY_DICER	TING_SILENCED_BY_DICER	28	-0,58334935	-1,6333718	0,003194888	0,038768057	1	4933	tags=61%, list=25%, signal=80%
LIU_VMYB_TARGETS_UP	LIU_VMYB_TARGETS_UP	116	-0,454476	-1,6327273	0,00135318	0,038926918	1	5677	tags=53%, list=28%, signal=73%
CLIMENT_BREAST_CANCER_COPY_NUMBER_UP	CLIMENT_BREAST_CANCER_COPY_NUMBER_UP	23	-0,60182047	-1,6325213	0,004807693	0,03888119	1	4700	tags=57%, list=23%, signal=74%
QI_PLASMACYTOMA_UP	QI_PLASMACYTOMA_UP	255	-0,42505226	-1,6324521	0	0,038751703	1	5202	tags=43%, list=26%, signal=57%
LINDSTEDT_DENDRITIC_CELL_MATURATION_B	LINDSTEDT_DENDRITIC_CELL_MATURATION_B	52	-0,5113294	-1,6313336	0,004451039	0,03910962	1	3840	tags=42%, list=19%, signal=52%
SNIJDERS_AMPLIFIED_IN_HEAD_AND_NECK_TUMORS	SNIJDERS_AMPLIFIED_IN_HEAD_AND_NECK_TUMORS	37	-0,55043364	-1,6307648	0,01141925	0,03925398	1	5062	tags=51%, list=25%, signal=69%
HOELZEL_NF1_TARGETS_UP	HOELZEL_NF1_TARGETS_UP	131	-0,4468287	-1,6307039	0	0,039136477	1	7163	tags=59%, list=36%, signal=91%
ZHAN_MULTIPLE_MYELOMA_DN	ZHAN_MULTIPLE_MYELOMA_DN	41	-0,53340507	-1,6282485	0,009160305	0,04014532	1	6303	tags=59%, list=32%, signal=85%
NIKOLSKY_BREAST_CANCER_8Q23_Q24_AMPLICON	NIKOLSKY_BREAST_CANCER_8Q23_Q24_AMPLICON	142	-0,44306806	-1,6280775	0	0,040100288	1	5618	tags=39%, list=28%, signal=53%
VERHAAK_AML_WITH_NPM1_MUTATED_DN	VERHAAK_AML_WITH_NPM1_MUTATED_DN	234	-0,42134878	-1,6269872	0	0,04046516	1	5448	tags=44%, list=27%, signal=60%
PLASARI_TGFB1_SIGNALING_VIA_NFIC_10HR_UP	PLASARI_TGFB1_SIGNALING_VIA_NFIC_10HR_UP	51	-0,51261675	-1,6268983	0,006116208	0,040360533	1	5638	tags=49%, list=28%, signal=68%
LEE_DOUBLE_POLAR_THYMOCYTE	LEE_DOUBLE_POLAR_THYMOCYTE	24	-0,604155	-1,6268479	0,008064516	0,040252965	1	4410	tags=58%, list=22%, signal=75%
TURASHVILI_BREAST_NORMAL_DUCTAL_VS_LOBULAR_UP	TURASHVILI_BREAST_NORMAL_DUCTAL_VS_LOBULAR_UP	62	-0,49660406	-1,6263275	0,004424779	0,040331695	1	4137	tags=37%, list=21%, signal=47%
RHEIN_ALL_GLUCOCORTICOID_THERAPY_UP	RHEIN_ALL_GLUCOCORTICOID_THERAPY_UP	74	-0,48487362	-1,6257198	0,00140647	0,04044483	1	5118	tags=45%, list=26%, signal=60%
ZHENG_FOXP3_TARGETS_IN_T_LYMPHOCYTE_DN	ZHENG_FOXP3_TARGETS_IN_T_LYMPHOCYTE_DN	35	-0,5570388	-1,6243215	0,00927357	0,041029666	1	4856	tags=54%, list=24%, signal=72%
BURTON_ADIPOGENESIS_3	BURTON_ADIPOGENESIS_3	101	-0,46191457	-1,6240523	0	0,041039594	1	5809	tags=50%, list=29%, signal=71%
PARK_TRETINOIN_RESPONSE	PARK_TRETINOIN_RESPONSE	12	-0,7155759	-1,6232405	0,010238908	0,041271035	1	3737	tags=83%, list=19%, signal=102%
TRAYNOR_RETT_SYNDROM_UP	TRAYNOR_RETT_SYNDROM_UP	40	-0,5394074	-1,6213688	0,006163328	0,042025965	1	6921	tags=70%, list=35%, signal=107%
WU_HBX_TARGETS_2_DN	WU_HBX_TARGETS_2_DN	14	-0,67309004	-1,620759	0,018062398	0,042188644	1	4041	tags=57%, list=20%, signal=72%
BROWNE_HCMV_INFECTION_8HR_UP	BROWNE_HCMV_INFECTION_8HR_UP	102	-0,4569618	-1,6202446	0,002785515	0,042266518	1	4846	tags=45%, list=24%, signal=59%
VANASSE_BCL2_TARGETS_DN	VANASSE_BCL2_TARGETS_DN	67	-0,49713406	-1,6198478	0,002873563	0,042302717	1	6456	tags=60%, list=32%, signal=88%
BONCI_TARGETS_OF_MIR15A_AND_MIR16_1	BONCI_TARGETS_OF_MIR15A_AND_MIR16_1	90	-0,46730113	-1,6196685	0	0,042278547	1	4703	tags=41%, list=24%, signal=54%
BILBAN_B_CLL_LPL_UP	BILBAN_B_CLL_LPL_UP	59	-0,50223875	-1,619097	0,00456621	0,042441625	1	6280	tags=58%, list=31%, signal=84%
VANTVEER_BREAST_CANCER_METASTASIS_DN	VANTVEER_BREAST_CANCER_METASTASIS_DN	109	-0,4579058	-1,6188264	0,002773925	0,04243564	1	4957	tags=43%, list=25%, signal=57%
THUM_MIR21_TARGETS_HEART_DISEASE_UP	THUM_MIR21_TARGETS_HEART_DISEASE_UP	16	-0,65515244	-1,6186743	0,011705685	0,042396482	1	6671	tags=94%, list=33%, signal=141%
EPPERT_CE_HSC_LSC	EPPERT_CE_HSC_LSC	39	-0,54156184	-1,6167096	0,00308642	0,04319909	1	6347	tags=67%, list=32%, signal=97%
BROCKE_APOPTOSIS_REVERSED_BY_IL6	BROCKE_APOPTOSIS_REVERSED_BY_IL6	143	-0,44640598	-1,6156398	0	0,04355994	1	4073	tags=40%, list=20%, signal=50%
STREICHER_LSM1_TARGETS_UP	STREICHER_LSM1_TARGETS_UP	43	-0,53137815	-1,6153643	0,001567398	0,043554377	1	5980	tags=60%, list=30%, signal=86%
RUTELLA_RESPONSE_TO_CSF2RB_AND_IL4_DN	RUTELLA_RESPONSE_TO_CSF2RB_AND_IL4_DN	301	-0,4092196	-1,6142294	0	0,04399614	1	4835	tags=45%, list=24%, signal=59%
APPEL_IMATINIB_RESPONSE	APPEL_IMATINIB_RESPONSE	32	-0,551619	-1,6141459	0,008183306	0,043886688	1	5056	tags=53%, list=25%, signal=71%
WANG_RESPONSE_TO_GSK3_INHIBITOR_SB216763_UP	WANG_RESPONSE_TO_GSK3_INHIBITOR_SB216763_UP	339	-0,40422174	-1,6137267	0	0,043919753	1	5054	tags=45%, list=25%, signal=59%
DUNNE_TARGETS_OF_AML1_MTG8_FUSION_UP	DUNNE_TARGETS_OF_AML1_MTG8_FUSION_UP	50	-0,51749057	-1,6133503	0,002849003	0,04397076	1	6137	tags=60%, list=31%, signal=86%
YAMASHITA_METHYLATED_IN_PROSTATE_CANCER	YAMASHITA_METHYLATED_IN_PROSTATE_CANCER	54	-0,50716263	-1,6132332	0,006079027	0,043876834	1	7101	tags=39%, list=35%, signal=60%
MORI_LARGE_PRE_BII_LYMPHOCYTE_DN	MORI_LARGE_PRE_BII_LYMPHOCYTE_DN	54	-0,5007742	-1,6127492	0,005882353	0,043963812	1	2329	tags=35%, list=12%, signal=40%
HERNANDEZ_MITOTIC_ARREST_BY_DOCETAXEL_1_DN	HERNANDEZ_MITOTIC_ARREST_BY_DOCETAXEL_1_DN	37	-0,5527488	-1,6118727	0,003169572	0,044288233	1	4789	tags=54%, list=24%, signal=71%
WEST_ADRENOCORTICAL_CARCINOMA_VS_ADENOMA_DN	WEST_ADRENOCORTICAL_CARCINOMA_VS_ADENOMA_DN	20	-0,6279403	-1,6117138	0,014634146	0,044222802	1	5129	tags=60%, list=26%, signal=81%
IVANOVA_HEMATOPOIESIS_STEM_CELL_SHORT_TERM	IVANOVA_HEMATOPOIESIS_STEM_CELL_SHORT_TERM	30	-0,5689727	-1,6096841	0,006144393	0,045083594	1	4579	tags=50%, list=23%, signal=65%
GARGALOVIC_RESPONSE_TO_OXIDIZED_PHOSPHOLIPIDS_BLUE_UP	GARGALOVIC_RESPONSE_TO_OXIDIZED_PHOSPHOLIPIDS_BLUE_UP	116	-0,44674042	-1,6096125	0	0,044966225	1	3688	tags=34%, list=18%, signal=41%
IVANOVA_HEMATOPOIESIS_MATURE_CELL	IVANOVA_HEMATOPOIESIS_MATURE_CELL	264	-0,41227227	-1,6071415	0	0,04605026	1	4463	tags=39%, list=22%, signal=49%
LIU_VAV3_PROSTATE_CARCINOGENESIS_UP	LIU_VAV3_PROSTATE_CARCINOGENESIS_UP	86	-0,46596608	-1,6069711	0,002857143	0,046029642	1	5811	tags=47%, list=29%, signal=65%
TAKEDA_TARGETS_OF_NUP98_HOXA9_FUSION_3D_DN	TAKEDA_TARGETS_OF_NUP98_HOXA9_FUSION_3D_DN	28	-0,5752226	-1,6069598	0,012269938	0,0458858	1	4518	tags=54%, list=23%, signal=69%
BROWNE_HCMV_INFECTION_6HR_DN	BROWNE_HCMV_INFECTION_6HR_DN	154	-0,43215528	-1,6055431	0	0,04654224	1	5426	tags=49%, list=27%, signal=66%
GAL_LEUKEMIC_STEM_CELL_DN	GAL_LEUKEMIC_STEM_CELL_DN	239	-0,41316777	-1,6053807	0	0,04648576	1	6762	tags=56%, list=34%, signal=83%
KUNINGER_IGF1_VS_PDGFB_TARGETS_DN	KUNINGER_IGF1_VS_PDGFB_TARGETS_DN	42	-0,5274225	-1,6049008	0,01174743	0,046597134	1	6508	tags=57%, list=33%, signal=85%
WESTON_VEGFA_TARGETS_3HR	WESTON_VEGFA_TARGETS_3HR	71	-0,48183924	-1,6019928	0	0,04810248	1	3853	tags=37%, list=19%, signal=45%
RUTELLA_RESPONSE_TO_CSF2RB_AND_IL4_UP	RUTELLA_RESPONSE_TO_CSF2RB_AND_IL4_UP	324	-0,40223435	-1,6015366	0	0,048222177	1	5428	tags=45%, list=27%, signal=61%
WANG_RESPONSE_TO_BEXAROTENE_DN	WANG_RESPONSE_TO_BEXAROTENE_DN	29	-0,5713178	-1,6013511	0,006289308	0,04817153	1	5809	tags=69%, list=29%, signal=97%
GU_PDEF_TARGETS_UP	GU_PDEF_TARGETS_UP	71	-0,48218977	-1,6002891	0,004347826	0,048635893	1	5806	tags=56%, list=29%, signal=79%
PAPASPYRIDONOS_UNSTABLE_ATEROSCLEROTIC_PLAQUE_UP	PAPASPYRIDONOS_UNSTABLE_ATEROSCLEROTIC_PLAQUE_UP	50	-0,5113098	-1,597996	0,004636785	0,04975186	1	5122	tags=52%, list=26%, signal=70%
HORIUCHI_WTAP_TARGETS_DN	HORIUCHI_WTAP_TARGETS_DN	292	-0,40589133	-1,5979159	0	0,049651965	1	4425	tags=41%, list=22%, signal=52%
BASSO_HAIRY_CELL_LEUKEMIA_UP	BASSO_HAIRY_CELL_LEUKEMIA_UP	79	-0,47309104	-1,5977964	0,00284495	0,04959755	1	6405	tags=61%, list=32%, signal=89%
GENTILE_RESPONSE_CLUSTER_D3	GENTILE_RESPONSE_CLUSTER_D3	61	-0,49430308	-1,5977226	0,004322767	0,04948195	1	5816	tags=57%, list=29%, signal=81%
DAUER_STAT3_TARGETS_DN	DAUER_STAT3_TARGETS_DN	49	-0,5145583	-1,5960133	0,002949853	0,050340123	1	6335	tags=63%, list=32%, signal=92%
CHIANG_LIVER_CANCER_SUBCLASS_CTNNB1_DN	CHIANG_LIVER_CANCER_SUBCLASS_CTNNB1_DN	161	-0,4283934	-1,5959779	0	0,050209396	1	6335	tags=53%, list=32%, signal=78%
PILON_KLF1_TARGETS_UP	PILON_KLF1_TARGETS_UP	481	-0,39049518	-1,595814	0	0,050153572	1	6008	tags=45%, list=30%, signal=62%
HOFMANN_MYELODYSPLASTIC_SYNDROM_RISK_DN	HOFMANN_MYELODYSPLASTIC_SYNDROM_RISK_DN	22	-0,5980151	-1,5954206	0,016	0,050250277	1	1369	tags=32%, list=7%, signal=34%
ASGHARZADEH_NEUROBLASTOMA_POOR_SURVIVAL_DN	ASGHARZADEH_NEUROBLASTOMA_POOR_SURVIVAL_DN	40	-0,52042735	-1,5952439	0,010339734	0,05018456	1	4561	tags=40%, list=23%, signal=52%
MCCLUNG_CREB1_TARGETS_DN	MCCLUNG_CREB1_TARGETS_DN	55	-0,49633253	-1,5950611	0	0,050115928	1	4177	tags=40%, list=21%, signal=50%
GOTZMANN_EPITHELIAL_TO_MESENCHYMAL_TRANSITION_UP	GOTZMANN_EPITHELIAL_TO_MESENCHYMAL_TRANSITION_UP	68	-0,4823033	-1,5948554	0,00284495	0,050057855	1	6011	tags=57%, list=30%, signal=82%
VICENT_METASTASIS_UP	VICENT_METASTASIS_UP	14	-0,6725909	-1,5940366	0,025728988	0,05036334	1	4935	tags=64%, list=25%, signal=85%
CHIBA_RESPONSE_TO_TSA_DN	CHIBA_RESPONSE_TO_TSA_DN	22	-0,60135096	-1,5937487	0,004830918	0,05041426	1	3574	tags=50%, list=18%, signal=61%
MASRI_RESISTANCE_TO_TAMOXIFEN_AND_AROMATASE_INHIBITORS_UP	MASRI_RESISTANCE_TO_TAMOXIFEN_AND_AROMATASE_INHIBITORS_UP	20	-0,6081011	-1,5928136	0,013490725	0,05080597	1	6884	tags=65%, list=34%, signal=99%
NICK_RESPONSE_TO_PROC_TREATMENT_DN	NICK_RESPONSE_TO_PROC_TREATMENT_DN	25	-0,5864849	-1,5917172	0,006420546	0,05125186	1	5034	tags=64%, list=25%, signal=85%
ZHAN_MULTIPLE_MYELOMA_CD1_VS_CD2_UP	ZHAN_MULTIPLE_MYELOMA_CD1_VS_CD2_UP	62	-0,4883606	-1,5914389	0,00877193	0,051283035	1	7050	tags=61%, list=35%, signal=94%
KORKOLA_YOLK_SAC_TUMOR	KORKOLA_YOLK_SAC_TUMOR	56	-0,49616933	-1,5902861	0,001497006	0,05179103	1	6387	tags=54%, list=32%, signal=78%
GARGALOVIC_RESPONSE_TO_OXIDIZED_PHOSPHOLIPIDS_MAGENTA_UP	GARGALOVIC_RESPONSE_TO_OXIDIZED_PHOSPHOLIPIDS_MAGENTA_UP	25	-0,58201605	-1,5896301	0,0144	0,052007925	1	4163	tags=52%, list=21%, signal=66%
HAHTOLA_MYCOSIS_FUNGOIDES_UP	HAHTOLA_MYCOSIS_FUNGOIDES_UP	18	-0,6242128	-1,5895406	0,021666666	0,051896807	1	3374	tags=50%, list=17%, signal=60%
LU_IL4_SIGNALING	LU_IL4_SIGNALING	90	-0,46215573	-1,5892012	0	0,05197545	1	6628	tags=56%, list=33%, signal=83%
VANHARANTA_UTERINE_FIBROID_UP	VANHARANTA_UTERINE_FIBROID_UP	44	-0,5205825	-1,5882905	0,00591716	0,05236759	1	7111	tags=70%, list=36%, signal=109%
PEDERSEN_METASTASIS_BY_ERBB2_ISOFORM_6	PEDERSEN_METASTASIS_BY_ERBB2_ISOFORM_6	28	-0,5556192	-1,5876898	0,020537125	0,052605785	1	6371	tags=57%, list=32%, signal=84%
NEWMAN_ERCC6_TARGETS_DN	NEWMAN_ERCC6_TARGETS_DN	37	-0,5330815	-1,5871941	0,004724409	0,052688003	1	7532	tags=70%, list=38%, signal=112%
DANG_REGULATED_BY_MYC_DN	DANG_REGULATED_BY_MYC_DN	247	-0,4108562	-1,586909	0	0,052734207	1	5912	tags=47%, list=30%, signal=66%
STARK_PREFRONTAL_CORTEX_22Q11_DELETION_UP	STARK_PREFRONTAL_CORTEX_22Q11_DELETION_UP	187	-0,41910842	-1,586791	0	0,05264228	1	5698	tags=47%, list=28%, signal=64%
GENTILE_UV_LOW_DOSE_DN	GENTILE_UV_LOW_DOSE_DN	63	-0,48550132	-1,5866439	0,007153076	0,052576073	1	6204	tags=57%, list=31%, signal=83%
KYNG_DNA_DAMAGE_BY_GAMMA_AND_UV_RADIATION	KYNG_DNA_DAMAGE_BY_GAMMA_AND_UV_RADIATION	88	-0,4578371	-1,5866014	0,002886003	0,052478593	1	5747	tags=48%, list=29%, signal=67%
WIERENGA_STAT5A_TARGETS_UP	WIERENGA_STAT5A_TARGETS_UP	202	-0,42133087	-1,5863415	0	0,05250844	1	5685	tags=45%, list=28%, signal=62%
JOHNSTONE_PARVB_TARGETS_1_DN	JOHNSTONE_PARVB_TARGETS_1_DN	56	-0,49334115	-1,586193	0,005970149	0,052440677	1	6951	tags=75%, list=35%, signal=115%
MITSIADES_RESPONSE_TO_APLIDIN_DN	MITSIADES_RESPONSE_TO_APLIDIN_DN	243	-0,40854973	-1,5857052	0	0,05256863	1	4468	tags=38%, list=22%, signal=48%
TURASHVILI_BREAST_LOBULAR_CARCINOMA_VS_LOBULAR_NORMAL_DN	TURASHVILI_BREAST_LOBULAR_CARCINOMA_VS_LOBULAR_NORMAL_DN	74	-0,4764581	-1,5854822	0,005571031	0,052551523	1	6985	tags=77%, list=35%, signal=118%
GRAHAM_CML_QUIESCENT_VS_NORMAL_QUIESCENT_DN	GRAHAM_CML_QUIESCENT_VS_NORMAL_QUIESCENT_DN	39	-0,5259013	-1,5852503	0,007598784	0,052578565	1	7235	tags=72%, list=36%, signal=112%
NIELSEN_GIST_AND_SYNOVIAL_SARCOMA_DN	NIELSEN_GIST_AND_SYNOVIAL_SARCOMA_DN	20	-0,6057999	-1,5844389	0,016891891	0,05291298	1	4970	tags=70%, list=25%, signal=93%
KYNG_DNA_DAMAGE_BY_GAMMA_RADIATION	KYNG_DNA_DAMAGE_BY_GAMMA_RADIATION	78	-0,47295687	-1,5838737	0,00140647	0,053164177	1	5383	tags=50%, list=27%, signal=68%
IIZUKA_LIVER_CANCER_EARLY_RECURRENCE	IIZUKA_LIVER_CANCER_EARLY_RECURRENCE	10	-0,73517257	-1,5827821	0,01655629	0,053669013	1	4132	tags=80%, list=21%, signal=101%
SESTO_RESPONSE_TO_UV_C0	SESTO_RESPONSE_TO_UV_C0	107	-0,4460542	-1,5817199	0,001381216	0,05413395	1	2560	tags=30%, list=13%, signal=34%
MIKKELSEN_ES_LCP_WITH_H3K4ME3_AND_H3K27ME3	MIKKELSEN_ES_LCP_WITH_H3K4ME3_AND_H3K27ME3	6	-0,84895855	-1,5811199	0,017391304	0,05430868	1	1559	tags=33%, list=8%, signal=36%
WANG_CLIM2_TARGETS_DN	WANG_CLIM2_TARGETS_DN	177	-0,41850471	-1,5808564	0	0,05429278	1	3608	tags=36%, list=18%, signal=43%
STEGER_ADIPOGENESIS_DN	STEGER_ADIPOGENESIS_DN	25	-0,5799255	-1,5808342	0,007633588	0,054163165	1	5076	tags=56%, list=25%, signal=75%
THEILGAARD_NEUTROPHIL_AT_SKIN_WOUND_DN	THEILGAARD_NEUTROPHIL_AT_SKIN_WOUND_DN	224	-0,41039133	-1,5789564	0	0,055132896	1	4983	tags=45%, list=25%, signal=59%
BILD_CTNNB1_ONCOGENIC_SIGNATURE	BILD_CTNNB1_ONCOGENIC_SIGNATURE	76	-0,46826276	-1,578296	0,004285714	0,055422544	1	5563	tags=50%, list=28%, signal=69%
CHOI_ATL_CHRONIC_VS_ACUTE_DN	CHOI_ATL_CHRONIC_VS_ACUTE_DN	18	-0,62247396	-1,5777276	0,00955414	0,05557555	1	5209	tags=67%, list=26%, signal=90%
DANG_MYC_TARGETS_DN	DANG_MYC_TARGETS_DN	31	-0,55377	-1,577217	0,02	0,055736627	1	3684	tags=45%, list=18%, signal=55%
ROVERSI_GLIOMA_LOH_REGIONS	ROVERSI_GLIOMA_LOH_REGIONS	42	-0,5177785	-1,5770694	0,00933126	0,0556777	1	5374	tags=52%, list=27%, signal=71%
RAMALHO_STEMNESS_UP	RAMALHO_STEMNESS_UP	196	-0,41706628	-1,576821	0	0,055679955	1	3684	tags=35%, list=18%, signal=42%
CHEN_ETV5_TARGETS_TESTIS	CHEN_ETV5_TARGETS_TESTIS	23	-0,5918016	-1,5759745	0,014150944	0,056024007	1	3226	tags=39%, list=16%, signal=47%
VERHAAK_AML_WITH_NPM1_MUTATED_UP	VERHAAK_AML_WITH_NPM1_MUTATED_UP	180	-0,41790497	-1,5750356	0	0,056475222	1	6285	tags=58%, list=31%, signal=83%
CROMER_TUMORIGENESIS_UP	CROMER_TUMORIGENESIS_UP	61	-0,48241246	-1,5735795	0,001449275	0,05724964	1	6947	tags=64%, list=35%, signal=98%
WESTON_VEGFA_TARGETS	WESTON_VEGFA_TARGETS	105	-0,44434458	-1,5735644	0	0,057109397	1	6144	tags=51%, list=31%, signal=74%
MARIADASON_RESPONSE_TO_BUTYRATE_CURCUMIN_SULINDAC_TSA_8	MARIADASON_RESPONSE_TO_BUTYRATE_CURCUMIN_SULINDAC_TSA_8	15	-0,64460874	-1,5728321	0,016103059	0,057404283	1	2822	tags=47%, list=14%, signal=54%
SHAFFER_IRF4_TARGETS_IN_MYELOMA_VS_MATURE_B_LYMPHOCYTE	SHAFFER_IRF4_TARGETS_IN_MYELOMA_VS_MATURE_B_LYMPHOCYTE	98	-0,45300213	-1,5720931	0,001388889	0,057724956	1	4321	tags=42%, list=22%, signal=53%
LINDVALL_IMMORTALIZED_BY_TERT_DN	LINDVALL_IMMORTALIZED_BY_TERT_DN	78	-0,46221453	-1,5718708	0,00591716	0,057721525	1	6809	tags=64%, list=34%, signal=97%
VALK_AML_CLUSTER_5	VALK_AML_CLUSTER_5	33	-0,5446167	-1,5717281	0,00953895	0,05768896	1	4587	tags=70%, list=23%, signal=90%
RODRIGUES_THYROID_CARCINOMA_ANAPLASTIC_DN	RODRIGUES_THYROID_CARCINOMA_ANAPLASTIC_DN	468	-0,38485467	-1,5715482	0	0,05761737	1	4940	tags=39%, list=25%, signal=50%
WANG_CISPLATIN_RESPONSE_AND_XPC_DN	WANG_CISPLATIN_RESPONSE_AND_XPC_DN	219	-0,41300294	-1,5714483	0,001262626	0,057531584	1	3915	tags=37%, list=20%, signal=45%
GRANDVAUX_IRF3_TARGETS_UP	GRANDVAUX_IRF3_TARGETS_UP	15	-0,6467185	-1,5712878	0,020967742	0,05745477	1	6245	tags=73%, list=31%, signal=107%
LIU_SOX4_TARGETS_UP	LIU_SOX4_TARGETS_UP	129	-0,4329297	-1,5711603	0,004016064	0,057369623	1	6381	tags=60%, list=32%, signal=87%
YIH_RESPONSE_TO_ARSENITE_C2	YIH_RESPONSE_TO_ARSENITE_C2	18	-0,6190817	-1,5710406	0,014705882	0,057296943	1	4336	tags=50%, list=22%, signal=64%
LU_TUMOR_ENDOTHELIAL_MARKERS_UP	LU_TUMOR_ENDOTHELIAL_MARKERS_UP	22	-0,5890007	-1,5709212	0,024271844	0,05720703	1	4419	tags=55%, list=22%, signal=70%
ASTON_MAJOR_DEPRESSIVE_DISORDER_DN	ASTON_MAJOR_DEPRESSIVE_DISORDER_DN	153	-0,42675912	-1,5709013	0	0,057073817	1	6310	tags=52%, list=32%, signal=76%
FREDERICK_PRKCI_TARGETS	FREDERICK_PRKCI_TARGETS	9	-0,73005044	-1,5706449	0,030716723	0,057064287	1	4949	tags=67%, list=25%, signal=89%
VANHARANTA_UTERINE_FIBROID_DN	VANHARANTA_UTERINE_FIBROID_DN	63	-0,48290527	-1,5705231	0,002932551	0,056961082	1	5316	tags=54%, list=27%, signal=73%
KAMMINGA_EZH2_TARGETS	KAMMINGA_EZH2_TARGETS	41	-0,51673245	-1,5694934	0,009009009	0,05742159	1	5145	tags=54%, list=26%, signal=72%
RODRIGUES_DCC_TARGETS_DN	RODRIGUES_DCC_TARGETS_DN	118	-0,43802986	-1,569217	0,001358696	0,05741337	1	4001	tags=38%, list=20%, signal=47%
LIAN_NEUTROPHIL_GRANULE_CONSTITUENTS	LIAN_NEUTROPHIL_GRANULE_CONSTITUENTS	24	-0,58072877	-1,5691444	0,018333333	0,05732157	1	3365	tags=38%, list=17%, signal=45%
PEPPER_CHRONIC_LYMPHOCYTIC_LEUKEMIA_DN	PEPPER_CHRONIC_LYMPHOCYTIC_LEUKEMIA_DN	20	-0,59203434	-1,5676191	0,01650165	0,058193047	1	1048	tags=25%, list=5%, signal=26%
ZHOU_INFLAMMATORY_RESPONSE_FIMA_UP	ZHOU_INFLAMMATORY_RESPONSE_FIMA_UP	465	-0,3846313	-1,5675913	0	0,0580771	1	5673	tags=42%, list=28%, signal=57%
MA_PITUITARY_FETAL_VS_ADULT_DN	MA_PITUITARY_FETAL_VS_ADULT_DN	19	-0,6136498	-1,5672587	0,017770598	0,058121562	1	4616	tags=63%, list=23%, signal=82%
INGRAM_SHH_TARGETS_UP	INGRAM_SHH_TARGETS_UP	122	-0,43258634	-1,5652579	0,001371742	0,05926606	1	6071	tags=47%, list=30%, signal=67%
CHARAFE_BREAST_CANCER_BASAL_VS_MESENCHYMAL_DN	CHARAFE_BREAST_CANCER_BASAL_VS_MESENCHYMAL_DN	49	-0,5015729	-1,5646222	0,004552352	0,059560556	1	7464	tags=80%, list=37%, signal=127%
FARMER_BREAST_CANCER_CLUSTER_4	FARMER_BREAST_CANCER_CLUSTER_4	19	-0,6071485	-1,5643998	0,013745705	0,059527196	1	5935	tags=79%, list=30%, signal=112%
WANG_MLL_TARGETS	WANG_MLL_TARGETS	282	-0,40073776	-1,5642496	0	0,059482735	1	7034	tags=56%, list=35%, signal=86%
ICHIBA_GRAFT_VERSUS_HOST_DISEASE_D7_UP	ICHIBA_GRAFT_VERSUS_HOST_DISEASE_D7_UP	96	-0,45006654	-1,5633839	0,001414427	0,05993525	1	3774	tags=41%, list=19%, signal=50%
HUANG_DASATINIB_RESISTANCE_UP	HUANG_DASATINIB_RESISTANCE_UP	78	-0,4629936	-1,56298	0,002873563	0,060061876	1	5294	tags=53%, list=26%, signal=71%
DOUGLAS_BMI1_TARGETS_DN	DOUGLAS_BMI1_TARGETS_DN	299	-0,39561555	-1,5621945	0	0,060323365	1	4985	tags=42%, list=25%, signal=56%
ICHIBA_GRAFT_VERSUS_HOST_DISEASE_35D_UP	ICHIBA_GRAFT_VERSUS_HOST_DISEASE_35D_UP	120	-0,43720892	-1,561859	0,001381216	0,060383875	1	3434	tags=36%, list=17%, signal=43%
NIKOLSKY_BREAST_CANCER_14Q22_AMPLICON	NIKOLSKY_BREAST_CANCER_14Q22_AMPLICON	12	-0,6849914	-1,5612289	0,026533997	0,060703356	1	3240	tags=58%, list=16%, signal=70%
HAN_JNK_SINGALING_DN	HAN_JNK_SINGALING_DN	37	-0,52743125	-1,5610081	0,006069803	0,060712483	1	7021	tags=59%, list=35%, signal=91%
CERVERA_SDHB_TARGETS_2	CERVERA_SDHB_TARGETS_2	108	-0,4378899	-1,5597343	0	0,061402075	1	5596	tags=44%, list=28%, signal=60%
VERHAAK_GLIOBLASTOMA_NEURAL	VERHAAK_GLIOBLASTOMA_NEURAL	125	-0,43259332	-1,5596918	0	0,06126796	1	4556	tags=37%, list=23%, signal=47%
DAVICIONI_TARGETS_OF_PAX_FOXO1_FUSIONS_UP	DAVICIONI_TARGETS_OF_PAX_FOXO1_FUSIONS_UP	243	-0,40442142	-1,5589638	0,001221001	0,06159939	1	6130	tags=50%, list=31%, signal=72%
NIKOLSKY_BREAST_CANCER_12Q13_Q21_AMPLICON	NIKOLSKY_BREAST_CANCER_12Q13_Q21_AMPLICON	42	-0,51765203	-1,5580854	0,007824726	0,06198497	1	5112	tags=45%, list=26%, signal=61%
MIYAGAWA_TARGETS_OF_EWSR1_ETS_FUSIONS_UP	MIYAGAWA_TARGETS_OF_EWSR1_ETS_FUSIONS_UP	246	-0,40174496	-1,5574616	0	0,062172372	1	5061	tags=41%, list=25%, signal=54%
SHARMA_PILOCYTIC_ASTROCYTOMA_LOCATION_DN	SHARMA_PILOCYTIC_ASTROCYTOMA_LOCATION_DN	7	-0,81844026	-1,5571873	0,008635579	0,062196825	1	1963	tags=29%, list=10%, signal=32%

ZHAN_LATE_DIFFERENTIATION_GENES_UP	ZHAN_LATE_DIFFERENTIATION_GENES_UP	33	-0,53136003	-1,5567151	0,014150944	0,062355883	1	5749	tags=64%, list=29%, signal=89%
KIM_GERMINAL_CENTER_T_HELPER_DN	KIM_GERMINAL_CENTER_T_HELPER_DN	22	-0,5897528	-1,5562459	0,025559105	0,062489275	1	2236	tags=41%, list=11%, signal=46%
NADERI_BREAST_CANCER_PROGNOSIS_DN	NADERI_BREAST_CANCER_PROGNOSIS_DN	18	-0,6112462	-1,5562242	0,01650165	0,062343784	1	4090	tags=56%, list=20%, signal=70%
CHOI_ATL_STAGE_PREDICTOR	CHOI_ATL_STAGE_PREDICTOR	38	-0,51508063	-1,5560774	0,009188362	0,062269777	1	4022	tags=37%, list=20%, signal=46%
MARZEC_IL2_SIGNALING_DN	MARZEC_IL2_SIGNALING_DN	36	-0,53157264	-1,554553	0,012269938	0,063095205	1	3855	tags=31%, list=19%, signal=38%
NEWMAN_ERCC6_TARGETS_UP	NEWMAN_ERCC6_TARGETS_UP	23	-0,58092445	-1,5544368	0,019543974	0,06300085	1	5106	tags=61%, list=26%, signal=82%
RAO_BOUND_BY_SALL4_ISOFORM_B	RAO_BOUND_BY_SALL4_ISOFORM_B	485	-0,38436833	-1,5544142	0	0,06286348	1	6269	tags=44%, list=31%, signal=63%
MYLLYKANGAS_AMPLIFICATION_HOT_SPOT_23	MYLLYKANGAS_AMPLIFICATION_HOT_SPOT_23	18	-0,6079122	-1,552594	0,028960818	0,06386226	1	2375	tags=56%, list=12%, signal=63%
NAKAJIMA_MAST_CELL	NAKAJIMA_MAST_CELL	46	-0,5037592	-1,5520992	0,007824726	0,06405135	1	6636	tags=63%, list=33%, signal=94%
LU_TUMOR_VASCULATURE_UP	LU_TUMOR_VASCULATURE_UP	29	-0,5479299	-1,552072	0,019817073	0,06390996	1	4581	tags=48%, list=23%, signal=63%
SERVITJA_ISLET_HNF1A_TARGETS_UP	SERVITJA_ISLET_HNF1A_TARGETS_UP	161	-0,41628984	-1,5512363	0	0,06427223	1	6140	tags=56%, list=31%, signal=80%
STEARMAN_LUNG_CANCER_EARLY_VS_LATE_UP	STEARMAN_LUNG_CANCER_EARLY_VS_LATE_UP	119	-0,43662053	-1,5511196	0	0,064187154	1	4917	tags=42%, list=25%, signal=55%
YANG_BREAST_CANCER_ESR1_LASER_DN	YANG_BREAST_CANCER_ESR1_LASER_DN	48	-0,50567585	-1,5510844	0,00777605	0,0640651	1	5224	tags=54%, list=26%, signal=73%
BAKKER_FOXO3_TARGETS_UP	BAKKER_FOXO3_TARGETS_UP	58	-0,4788783	-1,5508115	0,008810572	0,064092666	1	3462	tags=36%, list=17%, signal=44%
ROYLANCE_BREAST_CANCER_16Q_COPY_NUMBER_DN	ROYLANCE_BREAST_CANCER_16Q_COPY_NUMBER_DN	25	-0,56737614	-1,548794	0,023847377	0,0653349	1	7530	tags=60%, list=38%, signal=96%
NIELSEN_LEIOMYOSARCOMA_CNN1_DN	NIELSEN_LEIOMYOSARCOMA_CNN1_DN	19	-0,6066364	-1,5473546	0,021943573	0,06613399	1	5311	tags=68%, list=27%, signal=93%
COLIN_PILOCYTIC_ASTROCYTOMA_VS_GLIOBLASTOMA_DN	COLIN_PILOCYTIC_ASTROCYTOMA_VS_GLIOBLASTOMA_DN	28	-0,549347	-1,5471017	0,01710731	0,06614077	1	4581	tags=61%, list=23%, signal=79%
LINDGREN_BLADDER_CANCER_HIGH_RECURRENCE	LINDGREN_BLADDER_CANCER_HIGH_RECURRENCE	48	-0,49315277	-1,5449888	0,006069803	0,067334294	1	6336	tags=60%, list=32%, signal=88%
GAUSSMANN_MLL_AF4_FUSION_TARGETS_B_UP	GAUSSMANN_MLL_AF4_FUSION_TARGETS_B_UP	26	-0,569945	-1,5448033	0,021103896	0,06728242	1	7047	tags=62%, list=35%, signal=95%
HOOI_ST7_TARGETS_DN	HOOI_ST7_TARGETS_DN	109	-0,43399328	-1,5430813	0,00135318	0,06841793	1	6671	tags=49%, list=33%, signal=73%
GARGALOVIC_RESPONSE_TO_OXIDIZED_PHOSPHOLIPIDS_RED_DN	GARGALOVIC_RESPONSE_TO_OXIDIZED_PHOSPHOLIPIDS_RED_DN	24	-0,5656095	-1,5421327	0,01724138	0,06891094	1	4315	tags=50%, list=22%, signal=64%
SANA_RESPONSE_TO_IFNG_UP	SANA_RESPONSE_TO_IFNG_UP	64	-0,47627088	-1,5407721	0,004477612	0,06964341	1	4794	tags=47%, list=24%, signal=61%
SUNG_METASTASIS_STROMA_DN	SUNG_METASTASIS_STROMA_DN	51	-0,48907867	-1,5378028	0,004285714	0,071580745	1	6755	tags=63%, list=34%, signal=94%
WINZEN_DEGRADED_VIA_KHSRP	WINZEN_DEGRADED_VIA_KHSRP	97	-0,43566906	-1,5371072	0,002762431	0,07189917	1	6389	tags=53%, list=32%, signal=77%
DUNNE_TARGETS_OF_AML1_MTG8_FUSION_DN	DUNNE_TARGETS_OF_AML1_MTG8_FUSION_DN	19	-0,5881513	-1,5365651	0,0237691	0,072117485	1	4001	tags=53%, list=20%, signal=66%
WEBER_METHYLATED_ICP_IN_SPERM_UP	WEBER_METHYLATED_ICP_IN_SPERM_UP	8	-0,76420957	-1,5362738	0,032258064	0,07215426	1	4311	tags=13%, list=22%, signal=16%
BROWNE_HCMV_INFECTION_20HR_DN	BROWNE_HCMV_INFECTION_20HR_DN	98	-0,43525591	-1,5361112	0,005464481	0,07214697	1	4970	tags=47%, list=25%, signal=62%
ZHANG_RESPONSE_TO_CANTHARIDIN_UP	ZHANG_RESPONSE_TO_CANTHARIDIN_UP	19	-0,6068217	-1,5356325	0,02306425	0,072373874	1	6228	tags=68%, list=31%, signal=99%
YAGUE_PRETUMOR_DRUG_RESISTANCE_DN	YAGUE_PRETUMOR_DRUG_RESISTANCE_DN	12	-0,66723216	-1,5356162	0,025974026	0,07222474	1	5514	tags=83%, list=28%, signal=115%
SPIELMAN_LYMPHOBLAST_EUROPEAN_VS_ASIAN_2FC_DN	SPIELMAN_LYMPHOBLAST_EUROPEAN_VS_ASIAN_2FC_DN	20	-0,59443307	-1,534879	0,040123455	0,07259059	1	6217	tags=70%, list=31%, signal=101%
MULLIGHAN_NPM1_MUTATED_SIGNATURE_1_UP	MULLIGHAN_NPM1_MUTATED_SIGNATURE_1_UP	260	-0,38978407	-1,534573	0	0,07263094	1	4875	tags=38%, list=24%, signal=50%
BURTON_ADIPOGENESIS_9	BURTON_ADIPOGENESIS_9	89	-0,4472461	-1,534307	0,00554785	0,07263028	1	6059	tags=56%, list=30%, signal=80%
BOSCO_ALLERGEN_INDUCED_TH2_ASSOCIATED_MODULE	BOSCO_ALLERGEN_INDUCED_TH2_ASSOCIATED_MODULE	143	-0,42078272	-1,5341269	0	0,07256806	1	4498	tags=39%, list=22%, signal=50%
SANA_TNF_SIGNALING_DN	SANA_TNF_SIGNALING_DN	88	-0,44483447	-1,5339202	0,00140647	0,07255245	1	5105	tags=44%, list=26%, signal=59%
ZHU_SKIL_TARGETS_UP	ZHU_SKIL_TARGETS_UP	20	-0,5823353	-1,533559	0,015479876	0,072640024	1	3722	tags=45%, list=19%, signal=55%
BOYAULT_LIVER_CANCER_SUBCLASS_G3_UP	BOYAULT_LIVER_CANCER_SUBCLASS_G3_UP	183	-0,40476087	-1,5325557	0	0,07308581	1	5872	tags=49%, list=29%, signal=68%
WANG_NEOPLASTIC_TRANSFORMATION_BY_CCND1_MYC	WANG_NEOPLASTIC_TRANSFORMATION_BY_CCND1_MYC	21	-0,5870218	-1,5324212	0,025316456	0,07302093	1	7181	tags=76%, list=36%, signal=119%
MMS_MOUSE_LYMPH_HIGH_4HRS_UP	MMS_MOUSE_LYMPH_HIGH_4HRS_UP	34	-0,5243264	-1,5310652	0,0171875	0,073852524	1	4159	tags=50%, list=21%, signal=63%
SANA_TNF_SIGNALING_UP	SANA_TNF_SIGNALING_UP	78	-0,450803	-1,5304253	0,002853067	0,07417993	1	5244	tags=50%, list=26%, signal=67%
GYORFFY_MITOXANTRONE_RESISTANCE	GYORFFY_MITOXANTRONE_RESISTANCE	50	-0,4896841	-1,5303156	0,014018691	0,0740753	1	4569	tags=48%, list=23%, signal=62%
NELSON_RESPONSE_TO_ANDROGEN_UP	NELSON_RESPONSE_TO_ANDROGEN_UP	82	-0,44539654	-1,5288403	0,001408451	0,074973695	1	4043	tags=39%, list=20%, signal=49%
RIZ_ERYTHROID_DIFFERENTIATION_HBZ	RIZ_ERYTHROID_DIFFERENTIATION_HBZ	40	-0,50867283	-1,5264289	0,010852713	0,07655428	1	4215	tags=35%, list=21%, signal=44%
KOBAYASHI_EGFR_SIGNALING_24HR_UP	KOBAYASHI_EGFR_SIGNALING_24HR_UP	97	-0,44566962	-1,526202	0,002840909	0,076544784	1	5187	tags=38%, list=26%, signal=51%
BERTUCCI_INVASIVE_CARCINOMA_DUCTAL_VS_LOBULAR_DN	BERTUCCI_INVASIVE_CARCINOMA_DUCTAL_VS_LOBULAR_DN	45	-0,49038917	-1,5259465	0,00927357	0,076577745	1	5749	tags=58%, list=29%, signal=81%
OUILLETTE_CLL_13Q14_DELETION_UP	OUILLETTE_CLL_13Q14_DELETION_UP	70	-0,45917	-1,5255916	0,010479042	0,07666969	1	2743	tags=33%, list=14%, signal=38%
TAKEDA_TARGETS_OF_NUP98_HOXA9_FUSION_3D_UP	TAKEDA_TARGETS_OF_NUP98_HOXA9_FUSION_3D_UP	168	-0,40967008	-1,5248948	0	0,07704355	1	6384	tags=51%, list=32%, signal=75%
CHAN_INTERFERON_PRODUCING_DENDRITIC_CELL	CHAN_INTERFERON_PRODUCING_DENDRITIC_CELL	12	-0,6612962	-1,5248543	0,039933443	0,07690503	1	6777	tags=92%, list=34%, signal=139%
KIM_MYC_AMPLIFICATION_TARGETS_DN	KIM_MYC_AMPLIFICATION_TARGETS_DN	89	-0,44105902	-1,5238558	0,002710027	0,077480964	1	6966	tags=64%, list=35%, signal=98%
PEDRIOLI_MIR31_TARGETS_DN	PEDRIOLI_MIR31_TARGETS_DN	384	-0,37950376	-1,5222926	0	0,07854505	1	6559	tags=50%, list=33%, signal=73%
SCHAEFFER_PROSTATE_DEVELOPMENT_12HR_UP	SCHAEFFER_PROSTATE_DEVELOPMENT_12HR_UP	113	-0,42486307	-1,5221944	0,002781641	0,0784653	1	5594	tags=48%, list=28%, signal=66%
ROSS_LEUKEMIA_WITH_MLL_FUSIONS	ROSS_LEUKEMIA_WITH_MLL_FUSIONS	74	-0,4565033	-1,5213537	0,007173601	0,078900255	1	5287	tags=46%, list=26%, signal=62%
WU_SILENCED_BY_METHYLATION_IN_BLADDER_CANCER	WU_SILENCED_BY_METHYLATION_IN_BLADDER_CANCER	53	-0,47820103	-1,5213019	0,008746356	0,0787736	1	6268	tags=57%, list=31%, signal=82%
EHRLICH_ICF_SYNDROM_DN	EHRLICH_ICF_SYNDROM_DN	14	-0,63611853	-1,5207089	0,033280507	0,079053685	1	2636	tags=50%, list=13%, signal=58%
GENTILE_UV_RESPONSE_CLUSTER_D6	GENTILE_UV_RESPONSE_CLUSTER_D6	34	-0,52282965	-1,5197642	0,015243903	0,07960276	1	5978	tags=62%, list=30%, signal=88%
GAVIN_IL2_RESPONSIVE_FOXP3_TARGETS_UP	GAVIN_IL2_RESPONSIVE_FOXP3_TARGETS_UP	18	-0,6006114	-1,5193257	0,037096772	0,07981046	1	4482	tags=50%, list=22%, signal=64%
BURTON_ADIPOGENESIS_11	BURTON_ADIPOGENESIS_11	53	-0,47654948	-1,518748	0,01542777	0,08008262	1	3608	tags=42%, list=18%, signal=51%
IWANAGA_E2F1_TARGETS_INDUCED_BY_SERUM	IWANAGA_E2F1_TARGETS_INDUCED_BY_SERUM	27	-0,5477822	-1,5185543	0,028125	0,08004886	1	5908	tags=67%, list=30%, signal=94%
SASSON_RESPONSE_TO_FORSKOLIN_DN	SASSON_RESPONSE_TO_FORSKOLIN_DN	88	-0,43677434	-1,5175345	0,001392758	0,08058382	1	5666	tags=55%, list=28%, signal=76%
FAELT_B_CLL_WITH_VH_REARRANGEMENTS_UP	FAELT_B_CLL_WITH_VH_REARRANGEMENTS_UP	46	-0,48705825	-1,5166354	0,007575758	0,08112401	1	2952	tags=37%, list=15%, signal=43%
BOYLAN_MULTIPLE_MYELOMA_C_DN	BOYLAN_MULTIPLE_MYELOMA_C_DN	53	-0,4806147	-1,5158116	0,017804155	0,08157594	1	4722	tags=51%, list=24%, signal=67%
HUANG_FOXA2_TARGETS_UP	HUANG_FOXA2_TARGETS_UP	43	-0,4930765	-1,5154878	0,01724138	0,08165672	1	6455	tags=56%, list=32%, signal=82%
WESTON_VEGFA_TARGETS_6HR	WESTON_VEGFA_TARGETS_6HR	58	-0,46960947	-1,5150551	0,010130246	0,08185867	1	6140	tags=57%, list=31%, signal=82%
GARGALOVIC_RESPONSE_TO_OXIDIZED_PHOSPHOLIPIDS_LIGHTYELLOW_UP	GARGALOVIC_RESPONSE_TO_OXIDIZED_PHOSPHOLIPIDS_LIGHTYELLOW_UP	11	-0,68344766	-1,514851	0,034188036	0,0818362	1	2021	tags=55%, list=10%, signal=61%
HIRSCH_CELLULAR_TRANSFORMATION_SIGNATURE_UP	HIRSCH_CELLULAR_TRANSFORMATION_SIGNATURE_UP	232	-0,3918826	-1,5146224	0	0,08183712	1	3915	tags=36%, list=20%, signal=44%
PANGAS_TUMOR_SUPPRESSION_BY_SMAD1_AND_SMAD5_UP	PANGAS_TUMOR_SUPPRESSION_BY_SMAD1_AND_SMAD5_UP	123	-0,42054588	-1,5136861	0	0,08238087	1	4777	tags=45%, list=24%, signal=58%
OSWALD_HEMATOPOIETIC_STEM_CELL_IN_COLLAGEN_GEL_DN	OSWALD_HEMATOPOIETIC_STEM_CELL_IN_COLLAGEN_GEL_DN	255	-0,38488278	-1,5136	0,001199041	0,08229806	1	3962	tags=35%, list=20%, signal=42%
HANSON_HRAS_SIGNALING_VIA_NFKB	HANSON_HRAS_SIGNALING_VIA_NFKB	21	-0,58281076	-1,5133189	0,02852615	0,08230804	1	4417	tags=48%, list=22%, signal=61%
SASAI_RESISTANCE_TO_NEOPLASTIC_TRANSFROMATION	SASAI_RESISTANCE_TO_NEOPLASTIC_TRANSFROMATION	49	-0,48604286	-1,5126936	0,021084337	0,08269197	1	7023	tags=65%, list=35%, signal=100%
MAHADEVAN_IMATINIB_RESISTANCE_UP	MAHADEVAN_IMATINIB_RESISTANCE_UP	21	-0,5675584	-1,5126038	0,042789225	0,08259496	1	5542	tags=57%, list=28%, signal=79%
NIELSEN_SYNOVIAL_SARCOMA_DN	NIELSEN_SYNOVIAL_SARCOMA_DN	19	-0,58809215	-1,5119905	0,03773585	0,08292591	1	2634	tags=42%, list=13%, signal=48%
SCHAEFFER_PROSTATE_DEVELOPMENT_AND_CANCER_BOX6_UP	SCHAEFFER_PROSTATE_DEVELOPMENT_AND_CANCER_BOX6_UP	8	-0,74432445	-1,5109946	0,030852994	0,083546266	1	5122	tags=100%, list=26%, signal=134%
SHIN_B_CELL_LYMPHOMA_CLUSTER_8	SHIN_B_CELL_LYMPHOMA_CLUSTER_8	36	-0,51593435	-1,5102259	0,026825633	0,083979666	1	4793	tags=53%, list=24%, signal=69%
NAM_FXYD5_TARGETS_DN	NAM_FXYD5_TARGETS_DN	18	-0,6005935	-1,5099063	0,028571429	0,08403293	1	969	tags=33%, list=5%, signal=35%
SHAFFER_IRF4_TARGETS_IN_ACTIVATED_DENDRITIC_CELL	SHAFFER_IRF4_TARGETS_IN_ACTIVATED_DENDRITIC_CELL	61	-0,4659909	-1,509278	0,01179941	0,08432284	1	5243	tags=51%, list=26%, signal=69%
CAFFAREL_RESPONSE_TO_THC_UP	CAFFAREL_RESPONSE_TO_THC_UP	33	-0,5122352	-1,5084424	0,031866465	0,08482385	1	3743	tags=42%, list=19%, signal=52%
IWANAGA_CARCINOGENESIS_BY_KRAS_PTEN_DN	IWANAGA_CARCINOGENESIS_BY_KRAS_PTEN_DN	323	-0,37933537	-1,5083119	0	0,08474743	1	5670	tags=44%, list=28%, signal=60%
RAY_TARGETS_OF_P210_BCR_ABL_FUSION_UP	RAY_TARGETS_OF_P210_BCR_ABL_FUSION_UP	14	-0,6233929	-1,507802	0,034201954	0,08492081	1	4042	tags=57%, list=20%, signal=72%
JI_CARCINOGENESIS_BY_KRAS_AND_STK11_DN	JI_CARCINOGENESIS_BY_KRAS_AND_STK11_DN	17	-0,59900767	-1,5073234	0,037096772	0,08512576	1	6130	tags=76%, list=31%, signal=110%
BROWNE_INTERFERON_RESPONSIVE_GENES	BROWNE_INTERFERON_RESPONSIVE_GENES	66	-0,45394105	-1,5055093	0,010130246	0,08639342	1	5989	tags=50%, list=30%, signal=71%
HAHTOLA_SEZARY_SYNDROM_UP	HAHTOLA_SEZARY_SYNDROM_UP	93	-0,43573296	-1,5051231	0,008583691	0,08653713	1	4298	tags=41%, list=21%, signal=52%
HOWLIN_CITED1_TARGETS_1_DN	HOWLIN_CITED1_TARGETS_1_DN	35	-0,5186031	-1,5047951	0,010590015	0,08661294	1	5809	tags=57%, list=29%, signal=80%
GUO_HEX_TARGETS_UP	GUO_HEX_TARGETS_UP	81	-0,44181257	-1,5045844	0,005617978	0,086606964	1	5516	tags=51%, list=28%, signal=70%
BAE_BRCA1_TARGETS_DN	BAE_BRCA1_TARGETS_DN	32	-0,52765924	-1,5045244	0,023183925	0,08649346	1	4074	tags=47%, list=20%, signal=59%
YAUCH_HEDGEHOG_SIGNALING_PARACRINE_UP	YAUCH_HEDGEHOG_SIGNALING_PARACRINE_UP	139	-0,41501975	-1,5040797	0,004	0,08669969	1	6369	tags=49%, list=32%, signal=71%
FIGUEROA_AML_METHYLATION_CLUSTER_5_DN	FIGUEROA_AML_METHYLATION_CLUSTER_5_DN	44	-0,48566493	-1,5038272	0,019519519	0,08675039	1	2982	tags=30%, list=15%, signal=35%
PEDERSEN_METASTASIS_BY_ERBB2_ISOFORM_3	PEDERSEN_METASTASIS_BY_ERBB2_ISOFORM_3	18	-0,58941567	-1,5026155	0,032357473	0,08751455	1	4789	tags=56%, list=24%, signal=73%
EPPERT_LSC_R	EPPERT_LSC_R	38	-0,50208044	-1,5025446	0,012232416	0,08741817	1	5763	tags=66%, list=29%, signal=92%
RUIZ_TNC_TARGETS_DN	RUIZ_TNC_TARGETS_DN	136	-0,41324532	-1,501604	0,00538358	0,08802739	1	4159	tags=34%, list=21%, signal=42%
TOMLINS_PROSTATE_CANCER_DN	TOMLINS_PROSTATE_CANCER_DN	40	-0,5013023	-1,5015172	0,022522522	0,08792813	1	7680	tags=75%, list=38%, signal=121%
BOSCO_INTERFERON_INDUCED_ANTIVIRAL_MODULE	BOSCO_INTERFERON_INDUCED_ANTIVIRAL_MODULE	74	-0,45020682	-1,5010755	0,01396648	0,08815387	1	5810	tags=50%, list=29%, signal=70%
HOSHIDA_LIVER_CANCER_LATE_RECURRENCE_UP	HOSHIDA_LIVER_CANCER_LATE_RECURRENCE_UP	58	-0,45942605	-1,5005213	0,015558699	0,08841473	1	5690	tags=52%, list=28%, signal=72%
LIEN_BREAST_CARCINOMA_METAPLASTIC_VS_DUCTAL_UP	LIEN_BREAST_CARCINOMA_METAPLASTIC_VS_DUCTAL_UP	77	-0,44348398	-1,5003661	0,004267425	0,088371314	1	6547	tags=55%, list=33%, signal=81%
GUILLAUMOND_KLF10_TARGETS_UP	GUILLAUMOND_KLF10_TARGETS_UP	48	-0,48661447	-1,5000925	0,01826484	0,08839766	1	3228	tags=31%, list=16%, signal=37%
JAATINEN_HEMATOPOIETIC_STEM_CELL_UP	JAATINEN_HEMATOPOIETIC_STEM_CELL_UP	292	-0,38093257	-1,4992046	0	0,0889765	1	6361	tags=51%, list=32%, signal=73%
MCCABE_HOXC6_TARGETS_DN	MCCABE_HOXC6_TARGETS_DN	20	-0,5787407	-1,4971383	0,03	0,09050715	1	5760	tags=75%, list=29%, signal=105%
ZHANG_RESPONSE_TO_IKK_INHIBITOR_AND_TNF_UP	ZHANG_RESPONSE_TO_IKK_INHIBITOR_AND_TNF_UP	211	-0,39257607	-1,4970837	0,001305483	0,09036197	1	5359	tags=42%, list=27%, signal=56%
DORMOY_ELAVL1_TARGETS	DORMOY_ELAVL1_TARGETS	16	-0,60331595	-1,4962497	0,046104927	0,090871714	1	4059	tags=50%, list=20%, signal=63%
BRUINS_UVC_RESPONSE_VIA_TP53_GROUP_D	BRUINS_UVC_RESPONSE_VIA_TP53_GROUP_D	254	-0,38539445	-1,4960096	0	0,0909571	1	5399	tags=39%, list=27%, signal=53%
MARKEY_RB1_ACUTE_LOF_DN	MARKEY_RB1_ACUTE_LOF_DN	215	-0,3935748	-1,4959059	0,001248439	0,090880625	1	5683	tags=47%, list=28%, signal=64%
SHEDDEN_LUNG_CANCER_GOOD_SURVIVAL_A4	SHEDDEN_LUNG_CANCER_GOOD_SURVIVAL_A4	189	-0,3955025	-1,4958394	0,002534854	0,090751365	1	6896	tags=46%, list=34%, signal=69%
AMUNDSON_POOR_SURVIVAL_AFTER_GAMMA_RADIATION_8G	AMUNDSON_POOR_SURVIVAL_AFTER_GAMMA_RADIATION_8G	93	-0,4295567	-1,495257	0,009628611	0,09106776	1	4598	tags=44%, list=23%, signal=57%
RODWELL_AGING_KIDNEY_DN	RODWELL_AGING_KIDNEY_DN	132	-0,4089515	-1,4950961	0,002638523	0,09104211	1	5815	tags=42%, list=29%, signal=58%
APRELIKOVA_BRCA1_TARGETS	APRELIKOVA_BRCA1_TARGETS	48	-0,47631672	-1,4943738	0,023564065	0,09148523	1	4583	tags=46%, list=23%, signal=59%
CASORELLI_ACUTE_PROMYELOCYTIC_LEUKEMIA_UP	CASORELLI_ACUTE_PROMYELOCYTIC_LEUKEMIA_UP	163	-0,39808407	-1,4934925	0,003911343	0,09189145	1	5410	tags=42%, list=27%, signal=58%
ZHOU_INFLAMMATORY_RESPONSE_LPS_UP	ZHOU_INFLAMMATORY_RESPONSE_LPS_UP	367	-0,3739276	-1,4934536	0	0,09185892	1	5669	tags=40%, list=28%, signal=54%
MIKKELSEN_IPS_ICP_WITH_H3K27ME3	MIKKELSEN_IPS_ICP_WITH_H3K27ME3	51	-0,47470722	-1,4931102	0,013493253	0,09201809	1	4950	tags=27%, list=25%, signal=36%
PETROVA_ENDOTHELIUM_LYMPHATIC_VS_BLOOD_DN	PETROVA_ENDOTHELIUM_LYMPHATIC_VS_BLOOD_DN	157	-0,4024571	-1,4925983	0	0,09232659	1	6140	tags=50%, list=31%, signal=72%
HOWLIN_CITED1_TARGETS_1_UP	HOWLIN_CITED1_TARGETS_1_UP	33	-0,52212036	-1,4925032	0,020155039	0,09242315	1	4951	tags=48%, list=25%, signal=64%
WESTON_VEGFA_TARGETS_12HR	WESTON_VEGFA_TARGETS_12HR	35	-0,5120894	-1,4913644	0,029185869	0,09302618	1	7227	tags=69%, list=36%, signal=107%
DACOSTA_ERCC3_ALLELE_XPCS_VS_TTD_DN	DACOSTA_ERCC3_ALLELE_XPCS_VS_TTD_DN	34	-0,5157633	-1,490698	0,020833334	0,09334669	1	5893	tags=68%, list=29%, signal=96%
JIANG_AGING_CEREBRAL_CORTEX_DN	JIANG_AGING_CEREBRAL_CORTEX_DN	53	-0,46662998	-1,4902447	0,023809524	0,09355492	1	4390	tags=42%, list=22%, signal=53%
ONDER_CDH1_SIGNALING_VIA_CTNNB1	ONDER_CDH1_SIGNALING_VIA_CTNNB1	78	-0,4364166	-1,4886473	0,014409222	0,094765745	1	6140	tags=55%, list=31%, signal=79%
XU_HGF_SIGNALING_NOT_VIA_AKT1_48HR_DN	XU_HGF_SIGNALING_NOT_VIA_AKT1_48HR_DN	20	-0,56988305	-1,4868313	0,03210273	0,096187815	1	4714	tags=50%, list=24%, signal=65%
WOTTON_RUNX_TARGETS_DN	WOTTON_RUNX_TARGETS_DN	27	-0,53690016	-1,4866695	0,033762056	0,09614651	1	6443	tags=59%, list=32%, signal=87%
XU_RESPONSE_TO_TRETINOIN_UP	XU_RESPONSE_TO_TRETINOIN_UP	14	-0,6267907	-1,4865484	0,04418985	0,09605373	1	2021	tags=43%, list=10%, signal=48%
HELLER_SILENCED_BY_METHYLATION_UP	HELLER_SILENCED_BY_METHYLATION_UP	264	-0,37938824	-1,4864186	0	0,095995754	1	5714	tags=43%, list=29%, signal=59%
FARMER_BREAST_CANCER_CLUSTER_2	FARMER_BREAST_CANCER_CLUSTER_2	32	-0,51694983	-1,4864179	0,038639877	0,09581826	1	4957	tags=44%, list=25%, signal=58%
GOLDRATH_ANTIGEN_RESPONSE	GOLDRATH_ANTIGEN_RESPONSE	330	-0,3722865	-1,4853339	0	0,09657903	1	5934	tags=47%, list=30%, signal=65%
KYNG_DNA_DAMAGE_UP	KYNG_DNA_DAMAGE_UP	221	-0,385891	-1,4848161	0,002475248	0,09685952	1	5413	tags=43%, list=27%, signal=58%
ZHENG_IL22_SIGNALING_DN	ZHENG_IL22_SIGNALING_DN	40	-0,49069878	-1,4846692	0,014469453	0,0968132	1	6504	tags=55%, list=33%, signal=81%
CAIRO_HEPATOBLASTOMA_CLASSES_DN	CAIRO_HEPATOBLASTOMA_CLASSES_DN	204	-0,3919622	-1,4843605	0,001270648	0,096928716	1	5764	tags=46%, list=29%, signal=63%
GARGALOVIC_RESPONSE_TO_OXIDIZED_PHOSPHOLIPIDS_GREY_UP	GARGALOVIC_RESPONSE_TO_OXIDIZED_PHOSPHOLIPIDS_GREY_UP	16	-0,6116549	-1,4843397	0,044045676	0,09676176	1	2146	tags=44%, list=11%, signal=49%
NAKAMURA_ADIPOGENESIS_EARLY_UP	NAKAMURA_ADIPOGENESIS_EARLY_UP	60	-0,4642465	-1,4843204	0,010294118	0,096599646	1	6098	tags=55%, list=30%, signal=79%
NAKAYAMA_FRA2_TARGETS	NAKAYAMA_FRA2_TARGETS	41	-0,48787373	-1,4842622	0,026356589	0,09647401	1	4789	tags=54%, list=24%, signal=70%
SCHAEFFER_SOX9_TARGETS_IN_PROSTATE_DEVELOPMENT_DN	SCHAEFFER_SOX9_TARGETS_IN_PROSTATE_DEVELOPMENT_DN	44	-0,48314053	-1,4839789	0,016691957	0,09654248	1	5749	tags=59%, list=29%, signal=83%
ZHENG_RESPONSE_TO_ARSENITE_UP	ZHENG_RESPONSE_TO_ARSENITE_UP	18	-0,5911289	-1,4817388	0,034201954	0,09843831	1	7442	tags=78%, list=37%, signal=124%
HASINA_NOL7_TARGETS_UP	HASINA_NOL7_TARGETS_UP	13	-0,64230686	-1,4814422	0,04116638	0,09855564	1	3915	tags=54%, list=20%, signal=67%
PEPPER_CHRONIC_LYMPHOCYTIC_LEUKEMIA_UP	PEPPER_CHRONIC_LYMPHOCYTIC_LEUKEMIA_UP	32	-0,51293534	-1,4812747	0,030497592	0,098525725	1	4293	tags=47%, list=21%, signal=60%
XU_AKT1_TARGETS_48HR	XU_AKT1_TARGETS_48HR	9	-0,70607084	-1,4811916	0,05930807	0,09842469	1	5616	tags=78%, list=28%, signal=108%
MOREIRA_RESPONSE_TO_TSA_DN	MOREIRA_RESPONSE_TO_TSA_DN	18	-0,5866832	-1,4810556	0,035947714	0,098344855	1	4652	tags=56%, list=23%, signal=72%
LI_CISPLATIN_RESISTANCE_UP	LI_CISPLATIN_RESISTANCE_UP	24	-0,54897934	-1,4808382	0,03949447	0,098378174	1	5585	tags=50%, list=28%, signal=69%
WANG_IMMORTALIZED_BY_HOXA9_AND_MEIS1_DN	WANG_IMMORTALIZED_BY_HOXA9_AND_MEIS1_DN	23	-0,55259526	-1,4805888	0,031045752	0,098409094	1	6294	tags=70%, list=31%, signal=101%
PUIFFE_INVASION_INHIBITED_BY_ASCITES_DN	PUIFFE_INVASION_INHIBITED_BY_ASCITES_DN	140	-0,40736902	-1,4802569	0,009408603	0,09853315	1	5156	tags=43%, list=26%, signal=57%

Supplementary Table 2.

GSEA, leading edge and IPA pathway analyses performed on tumor samples from a selected TCGA-COAD cohort stratified according to miR-27a levels.

2D) number of gene-sets (core-gene sets) enriched in high-miR-27a CRCs where a gene was found overlapping (in at least 5 gene-sets);

SYMBOL	#shared genesets	SYMBOL	#shared genesets	SYMBOL	#shared genesets	SYMBOL	#shared genesets	SYMBOL	#shared genesets	SYMBOL	#shared genesets	SYMBOL	#shared genesets	SYMBOL	#shared genesets	SYMBOL	#shared genesets	SYMBOL	#shared genesets
COX6A1	10	PWWP2B	3	ZNF593	2	PYGO2	1	XRCC1	1	ITGB4	4	P4HB	2	WNK4	1	NMU	1	ZNF629	1
COX4I1	10	ICA1	3	AP2S1	2	DCST1	1	TECR	1	SLC16A5	4	NCOR2	2	DHX32	1	EPHX4	1	ARTN	1
COX5B	9	ARRDC1	3	CHAF1A	2	DCST2	1	KIF22	1	NAT6	4	ABLIM1	2	ANO9	1	TGFBI	1	HYAL3	1
ATP5E	9	MLPH	3	CUEDC2	2	EFNA1	1	CTTN	1	FAM195A	4	STK11	2	PACSIN3	1	CADPS	1	CRYBB2	1
TMSB10	8	TMEM125	3	PPP1R7	2	EFNA3	1	SERTAD3	1	ATP5J2	4	NUP62	2	CPT2	1	POFUT1	1	PHF19	1
MRPS12	8	TMC6	3	DCXR	2	THBS3	1	DPAGT1	1	COX7A2L	4	TCF25	2	ABHD14B	1	SMYD5	1	ZP3	1
GPX4	8	LLGL2	3	SSNA1	2	THEM4	1	MZF1	1	MTA1	4	UBTF	2	NUP37	1	CCDC94	1	PIH1D1	1
COX6C	8	ABCC3	3	SHFM1	2	CLK2	1	RTN3	1	MRPL41	4	PPP5C	2	RNF135	1	NBEAL2	1	GSDMA	1
COX7C	8	NONO	3	MRPL52	2	FAM189B	1	PEX6	1	ABHD11	4	HGS	2	TMEM158	1	RRP9	1	TYSND1	1
NDUFA7	8	PSMB3	3	PSMC4	2	HCN3	1	SOX12	1	CSTB	4	BSG	2	SLC28A3	1	GTPBP3	1	TRIP6	1
NDUFS8	8	CCND2	3	ELOF1	2	OBFC1	1	BRD8	1	RPL39	4	PMPCA	2	ILDR1	1	WDR62	1	CYB561D2	1
NDUFS6	8	ARF1	3	TXNDC17	2	FRAT1	1	WWP2	1	RPL37A	4	PFKP	2	COASY	1	GAR1	1	ZNF589	1
UQCR11	8	PTMA	3	CSK	2	SIRT3	1	TUSC2	1	AES	4	FLNB	2	UNC93B1	1	LHPP	1	FAM71E1	1
ATP5D	8	UBE2C	3	PSMD4	2	DNAJC17	1	NAGLU	1	NDUFB11	4	AKAP17A	2	VIPR1	1	SETD4	1	WASH2P	1
PRDX2	8	ERCC1	3	SHARPIN	2	ARHGAP8	1	GTF2F2	1	DBI	4	NLRC5	2	CMAS	1	WDR34	1	PCNXL3	1
RPLP2	7	PPAN	3	THOC3	2	P4HTM	1	ZNF544	1	PQBP1	4	TFEB	2	CCT6B	1	WDR4	1	HABP4	1
EIF3K	7	PMF1	3	ISOC2	2	ZNF74	1	WRAP73	1	EBP	4	SLC19A1	2	MIF4GD	1	METTL1	1	CLIC3	1
RPL18	7	POLE	3	EMD	2	FUK	1	IMPDH1	1	DPM3	4	AP3D1	2	KRT18	1	DTYMK	1	MYL6B	1
RPS2	7	UBL5	3	PTTG1	2	ARFGAP2	1	MAP4	1	GRK6	4	THOP1	2	CHCHD5	1	MYEF2	1	TMEM141	1
TOMM40	7	SAFB	3	FOXK2	2	UBQLN4	1	CCHCR1	1	STXBP2	4	TSPAN14	2	RWDD3	1	MAPK7	1	ARHGAP21	1
ETFB	7	ACY1	3	CUTA	2	TTC39A	1	B4GALT2	1	S100A11	4	MCF2L	2	CDKN2D	1	AAAS	1	NUDT19	1
FARSA	7	GALE	3	SURF2	2	CCDC97	1	HEBP2	1	MAP2K2	4	AIM1L	2	RPS11	1	TSEN2	1	CNPY2	1
ATP5C1	7	TCIRG1	3	TSPAN17	2	DHRS13	1	B4GALT7	1	BCAP31	4	ARHGAP27	2	HMGB1	1	RNASEH2C	1	SLC27A4	1
ATP5H	7	GTF3C1	3	IFT43	2	CACNA1D	1	ARL4A	1	GPX1	4	NSUN5	2	UBE2E3	1	LRP8	1	LINC00116	1
MRPS18A	7	PIN1	3	PSMB8	2	DEGS2	1	ACTR1A	1	CLDN4	4	RRP1	2	NCBP2	1	ZFP2	1	OCIAD2	1
MRPL12	7	PSMB5	3	PPP1R1B	2	FBP1	1	GABBR1	1	SGTA	4	TRPM4	2	UBOX5	1	PCBP4	1	CWF19L1	1
COX8A	7	EIF6	3	MGMT	2	RSPH1	1	VPS28	1	TRAPPC2L	4	ULK1	2	LRP5L	1	EXOSC1	1	HIBADH	1
ATP5G2	7	TMEM147	3	LMTK3	2	PRKD2	1	NBL1	1	HCFC1R1	4	EFNA4	2	RRN3P1	1	FHOD1	1	PCM1	1
ATP5G1	7	CDK2AP2	3	RPS19BP1	2	KIFC2	1	GRHPR	1	TELO2	4	MUC1	2	FUS	1	ATG9B	1	MIS18A	1
NDUFAB1	7	RPL37	3	CENPV	2	TMEM229B	1	PFDN1	1	MRPL23	4	RORC	2	BECN1	1	DDX49	1	TMEM208	1
NDUFS3	7	SCNN1A	3	PSMB10	2	RAB3D	1	MAPK3	1	SNRPE	4	TP53TG1	2	HNRNPF	1	RANGRF	1	PHC2	1
NDUFV2	7	SNRPA	3	PLEKHJ1	2	PLXNA3	1	ECSIT	1	NDUFA13	4	RAB17	2	PSMB2	1	HOXA9	1	NFIX	1
ATP5J	7	RHOT2	3	IFI27	2	FANCF	1	RABAC1	1	GUK1	4	PATZ1	2	SQSTM1	1	DUS3L	1	MINOS1	1
ATP5I	7	NTHL1	3	IER3	2	DNAJC1	1	PEF1	1	MRPL28	4	SNRNP35	2	SLC25A6	1	SKA1	1	NR1H2	1
UQCRC1	7	MRPL38	3	ZNF133	2	CCDC64	1	DHX30	1	ROMO1	4	SLC29A2	2	AAMP	1	BIVM	1	THOC7	1
NDUFA2	7	CDK16	3	PSMB7	2	PNN	1	PES1	1	NENF	4	KIAA0556	2	CETN2	1	NHP2	1	HSPBP1	1
UQCRQ	7	UBXN6	3	PMVK	2	COG7	1	PLA2G16	1	SCAND1	4	FRAT2	2	CD63	1	DNMT3B	1	DDRGK1	1
APRT	7	SCO2	3	ALKBH2	2	CCDC64B	1	MAST3	1	MLF2	4	PEX11B	2	RPL34	1	TP53RK	1	COQ4	1
EIF3G	7	ADA	3	AVPI1	2	ROGDI	1	ACD	1	SIGIRR	4	RHBDF1	2	RPL9	1	BCL11B	1	FAM136A	1
SNRNP70	6	DGCR6	3	ODF2	2	DBP	1	CTDNEP1	1	CLDN7	4	PAQR4	2	STMN1	1	BCL11A	1	TMEM44	1
QARS	6	RUVBL2	3	LAMTOR2	2	TMEM79	1	SPAG7	1	ENSA	4	ZG16B	2	SSR2	1	HSPBAP1	1	TMEM42	1
RPS16	6	CDK10	3	EIF2B4	2	GGCT	1	DTX4	1	LSM7	4	JMJD8	2	GPS1	1	USP20	1	MRPL55	1
RPL13	6	TKT	3	COMT	2	SLC25A29	1	CDIPT	1	KRTCAP2	4	CYB561	2	NCL	1	RGS12	1	DGCR2	1
RPL18A	6	DYNLRB1	3	PSMC1	2	EPHB3	1	YIF1A	1	RPL36AL	4	WFS1	2	CD151	1	MMP24	1	LYSMD4	1
GLTSCR2	6	TESK1	3	SPSB3	2	ENTPD2	1	VEGFA	1	PSMB4	4	SPATA2L	2	MGST2	1	CEP250	1	ZDHHC8	1
SPG7	6	TAF15	3	HIGD2A	2	ULK3	1	SPINK1	1	GPI	4	GET4	2	ANP32B	1	MAP1LC3A	1	IMMP1L	1
ECHS1	6	WDR74	3	RAB4B	2	MMEL1	1	MAGED1	1	NDUFB10	4	AZGP1	2	CPT1B	1	TRPC4AP	1	ARVCF	1
DAP3	6	H2AFJ	3	CHMP4B	2	SFI1	1	DSP	1	NME1	4	PRSS8	2	TCF7L2	1	PIGU	1	BCCIP	1
FDXR	6	IFI35	3	SLIRP	2	NPDC1	1	DPP7	1	EIF4A1	4	SETD6	2	KIF21B	1	SPAG4	1	PPP1R9A	1
NDUFA4	6	LOC93622	3	PRELID1	2	PLEKHA6	1	ITPA	1	PDLIM1	4	SH2D3A	2	JAG1	1	CENPB	1	DDX56	1
COX6B1	6	TRAPPC5	3	FAM162A	2	CAPN8	1	PPP1R14C	1	TAF10	4	EPHA1	2	PKP1	1	SRSF9	1	PLCB4	1
ATP5O	6	TEAD4	3	MRPL51	2	GGT6	1	RNF39	1	CCDC85B	4	TMEM191A	2	EEF1D	1	VPS25	1	FIG4	1
NDUFB1	6	STUB1	3	MRPS17	2	EPS8L1	1	RHOD	1	NAA10	4	DUSP16	2	RALA	1	CCDC12	1	BSCL2	1
NDUFA1	6	PPP1R14B	3	TPD52L1	2	ZXDC	1	MYO7A	1	INTS1	4	TMEM134	2	TIMM8B	1	LIAS	1	TRMT2A	1
NDUFB8	6	BUD31	3	ZNHIT1	2	PRR15	1	PPP3CC	1	NUDT1	4	SH3YL1	2	EHD4	1	COPE	1	SS18L2	1
NDUFA3	6	SEC61B	3	CBR1	2	RNF103	1	CRTC1	1	CSNK1G2	4	HN1	2	CNP	1	MRPL21	1	CORO7	1
NDUFC1	6	EIF1	3	WHSC1	2	TNKS1BP1	1	TARS2	1	TOM1	3	PTRH2	2	CEBPD	1	SAMM50	1	CHCHD1	1
NDUFB2	6	PFDN5	3	FN3K	2	CACNB3	1	FKBP8	1	OS9	3	PNPO	2	SPTBN1	1	DDX54	1	DPM2	1
MBD3	6	PRDX1	3	MZT2B	2	ANKRD22	1	GOLGA8A	1	CLDN3	3	PPP1R9B	2	RPS29	1	PARL	1	POMP	1
NDUFB7	6	PFDN6	3	GDF15	2	SERINC2	1	DNAJC4	1	PVRL4	3	FASN	2	RPLP1	1	TMEM160	1	DTNBP1	1
BCR	6	NDUFA11	3	ZNF263	2	SMPDL3B	1	POLR1E	1	KRT19	3	GALK1	2	PERP	1	TOR2A	1	SYF2	1
SMARCA4	6	PSMB6	3	JUND	2	GOLT1A	1	VAC14	1	SULT2B1	3	STRA13	2	LAMB3	1	BAG1	1	CDKN1A	1
PFN1	6	SLC25A39	3	BAP1	2	ABCG1	1	TMEM8B	1	AP1M2	3	SAP30BP	2	RPL41	1	EIF4E2	1	PCGF3	1
GIPC1	6	CYHR1	3	CXCL3	2	SKAP1	1	ZNF219	1	DLG3	3	PSMC5	2	AK1	1	TWF2	1	SMOX	1
ACOT7	6	WDR45	3	SEMA3B	2	ARL16	1	EPHB1	1	CIB1	3	ACOX1	2	ABHD6	1	PDCD6	1	EFNB1	1
PDCD5	6	RMND5B	3	PABPC4	2	CSNK1D	1	AATK	1	PGD	3	SMARCD2	2	COX7A2	1	CEP72	1	CCNE1	1
S100A6	6	RAD23A	3	LMNB2	2	B3GNTL1	1	CHKA	1	SCNM1	3	RNF43	2	EIF3M	1	NKD2	1	TAP1	1
TCEB2	6	SAT1	3	KAT2A	2	NAT9	1	PMS2P1	1	RPS15A	3	DNAJC7	2	USE1	1	TERT	1	RSL1D1	1
F11R	6	HSPA1A	3	RARS	2	XYLT2	1	DBNDD1	1	RPL12	3	SPATA24	2	RPS23	1	SLC6A3	1	LIF	1
LMNA	6	CST3	3	GCSH	2	ARMC7	1	CPNE7	1	DHPS	3	TRIOBP	2	RPS8	1	BRD9	1	STRN4	1
TXN	6	CXCL1	3	PIEZO1	2	NUP85	1	DEF8	1	EEF2	3	WIPI2	2	SPRY2	1	TMCO6	1	PRKACA	1
PAFAH1B3	5	CXCL2	3	SMAD6	2	LRRC37A3	1	KLHDC4	1	RPL32	3	HAUS5	2	CBX4	1	TONSL	1	U2AF1	1
RPS12	5	SRM	3	HNRNPA2B1	2	MKS1	1	ZNF276	1	S100A10	3	TUBGCP2	2	DHX38	1	SCO1	1	AGPAT2	1
RPL29	5	ALG3	3	ERCC5	2	SLC38A10	1	ANKRD11	1	FKBP4	3	NPEPL1	2	F8A1	1	CRAT	1	SCRIB	1
RPS14	5	SCAP	3	CDC37	2	WIPI1	1	SPIRE2	1	ANXA11	3	CTBP2	2	MCCC1	1	PCCB	1	EDC4	1
RPL28	5	ARF5	3	MANF	2	CCDC57	1	ZFPM1	1	RPS20	3	UBE2I	2	ACSL5	1	ATP6V1G1	1	USP5	1
RPS19	5	S100A4	3	RPS28	2	SLC35B1	1	BANP	1	PLP2	3	CLDN9	2	BAD	1	NDUFAF1	1	SKIV2L	1
RPL7A	5	VDAC1	3	UBC	2	STRADA	1	ACSF3	1	RPL17	3	LUC7L	2	SHB	1	DIABLO	1	BYSL	1
GSTP1	5	PPM1G	3	SEPW1	2	RFNG	1	ALKBH7	1	HNRNPA1	3	MAPK8IP3	2	PPA1	1	NDUFA12	1	BMS1	1
ACTG1	5	IMPDH2	3	SOD1	2	NT5C	1	PLOD3	1	GAPDH	3	ZNF598	2	CROT	1	ATP1B3	1	RPIA	1
RPL35A	5	EIF3B	3	GPS2	2	TSEN54	1	PCYT2	1	HINT1	3	FLYWCH1	2	ATPAF2	1	COQ3	1	PWP2	1
TALDO1	5	HNRNPD	3	SNRPF	2	TMEM92	1	CDC25C	1	RPL23	3	MPG	2	PYGB	1	SLC40A1	1	NOL12	1
EIF3F	5	SGSM3	3	PTOV1	2	SNF8	1	RFXANK	1	RPS13	3	WDR90	2	ADCK2	1	MRPL32	1	ETV2	1
GNB2L1	5	ARPC5L	3	UBXN1	2	LRRC45	1	FAM207A	1	SIN3B	3	GNPTG	2	ADCK3	1	MRPL45	1	RNPEP	1
RPS25	5	HIP1R	3	PPIB	2	HEXDC	1	ADORA2B	1	UFC1	3	ASCL2	2	SLC25A38	1	FH	1	POLR2L	1
NDUFS7	5	EHMT2	3	CD99	2	GPRC5C	1	RILP	1	TIMM17A	3	HES6	2	G6PD	1	SLC9A5	1	NXF1	1
TST	5	ARMC6	3	ACTN4	2	RECQL5	1	RIN1	1	TRAP1	3	NQO1	2	DDX28	1	HAX1	1	RPLP0	1
ENDOG	5	IFRD2	3	PRKCSH	2	FTSJ3	1	MRPS24	1	ALAS1	3	RTEL1	2	METTL17	1	NDUFB4	1	IER2	1
TUFM	5	LRCH4	3	AGRN	2	FSCN2	1	AP4B1	1	NCAPH2	3	TMEM186	2	PAM16	1	CHDH	1	ISCU	1
ALDH4A1	5	SFSWAP	3	CCND1	2	SPATA20	1	MANBAL	1	SLC25A11	3	NRF1	2	LCAT	1	PRDX5	1	EIF5A	1
IDH2	5	ZNF444	3	ABCC1	2	RSAD1	1	GNB2	1	GNAS	3	RNASEH2A	2	DUSP1	1	FIBP	1	DEPDC5	1
NFS1	5	RASSF7	3	YKT6	2	CHAD	1	CARKD	1	MRPS2	3	DHX58	2	TAF6L	1	ATP8A1	1	CTDSPL	1
UQCRH	5	TCF3	3	ARHGEF1	2	CCDC137	1	POLD2	1	OGDH	3	MVK	2	TMED9	1	MCCC2	1	SORBS3	1
SURF1	5	TNFRSF25	2	SMG5	2	DDX42	1	CCS	1	CLN3	3	NOP56	2	NR2E3	1	MRPS18B	1	TPRA1	1
VDAC2	5	PLEKHG5	2	CNOT3	2	RAB40B	1	IPO13	1	OAZ3	3	RBM39	2	LSM12	1	HINT2	1	SEC61A1	1
NDUFB5	5	THAP3	2	SMARCD1	2	HOXB9	1	GNL3	1	TK2	3	DRAP1	2	ZBED1	1	TOMM7	1	SRC	1
NDUFS2	5	GPR153	2	OLA1	2	HOXB7	1	ADRBK1	1	MRPL16	3	N6AMT2	2	CTRL	1	NCLN	1	TOP3B	1
ATP5L	5	ESPN	2	GAPDHS	2	SRP68	1	DHRS1	1	MRPL2	3	FAM96B	2	AURKB	1	CCDC9	1	HMGA1	1
SDHA	5	DNAJC11	2	NOL3	2	FAM104A	1	DEF6	1	TOMM20	3	HMBS	2	RBM42	1	ECHDC2	1	TSPAN15	1
POLG	5	PFKL	2	BLVRB	2	METRNL	1	HMGN3	1	HMGCL	3	LSM4	2	MAU2	1	ETS2	1	EIF2S3	1
MPST	5	TSPAN1	2	FBXL18	2	NACA2	1	CCDC25	1	ABCB8	3	ERP29	2	MMP15	1	GTF2IRD1	1	HSPB11	1
PPP2R1A	5	ATP6AP1	2	FTSJ2	2	KCTD2	1	MAP2K7	1	DNASE2	3	UBE2S	2	HNRNPAB	1	ASXL1	1	ACBD6	1
ARHGDIA	5	ATP6V1F	2	MAD1L1	2	TBCD	1	PIR	1	FPGS	3	EXOC7	2	BRMS1	1	GABRE	1	COX19	1
JUP	5	PAOX	2	LFNG	2	ACSF2	1	ZNF777	1	IDH3B	3	NDUFA8	2	TOLLIP	1	APBB3	1	TNRC18	1
LAD1	5	ASPSCR1	2	TMEM184A	2	TMEM104	1	NOB1	1	SLC25A28	3	COX7B	2	MBD2	1	PIGL	1	BRAT1	1
SPINT1	5	ATP6V0E2	2	IQCE	2	TMEM105	1	NOA1	1	DHODH	3	COX5A	2	COL17A1	1	KATNB1	1	TTYH3	1
BAIAP2	5	NDUFV3	2	GTF2H4	2	LUC7L3	1	HDAC6	1	GCAT	3	GSTO1	2	NOP16	1	SH2B1	1	PSMG3	1
NME3	5	ARHGEF16	2	CSNK1E	2	CHMP6	1	DGCR6L	1	PPIF	3	SEC61G	2	TRIM16	1	ERAL1	1	MICALL2	1
HAGHL	5	KIAA1522	2	HHLA3	2	IFT172	1	CBR3	1	CLPP	3	NDUFV1	2	CELSR1	1	PEX5	1	FOXK1	1
NACA	5	CAPG	2	SLC12A9	2	PPP2R4	1	STOML2	1	POLG2	3	GSTZ1	2	CARD10	1	UPF3A	1	MAFK	1
TP53	5	ZDHHC12	2	DAZAP1	2	SDHAF1	1	PPP2R2D	1	SLC25A10	3	SCAMP3	2	NOP2	1	FMR1	1	FNBP4	1
SFN	5	MTUS1	2	TMEM63A	2	RAPGEF3	1	LIPE	1	MRPS16	3	NDUFS5	2	TNFRSF10B	1	REPIN1	1	LARP1	1
ATOX1	5	PPFIBP2	2	GRAMD4	2	NADK	1	IKBKG	1	COX17	3	FBXO46	2	PA2G4	1	INPP5D	1	SPHK2	1
RNF126	5	GSTO2	2	TNK2	2	UBE3B	1	TCOF1	1	PDHA1	3	PABPN1	2	DTX2	1	ELMO2	1	ATXN2L	1
CHMP2A	5	MYO5C	2	LPAR2	2	CELSR3	1	CAPZB	1	WARS2	3	CDK5RAP3	2	JUN	1	SPATA2	1	FBXO44	1
H2AFZ	5	DENND2D	2	SRRT	2	IRF2BP1	1	BIRC5	1	AIFM1	3	ZNF652	2	SPTAN1	1	UCKL1	1	PPP6R2	1
MTX1	5	RALGPS1	2	DCAF8	2	RARG	1	MMAB	1	DLD	3	HOXB5	2	TYK2	1	PTK6	1	LENG8	1
RPL36	5	CAMSAP3	2	CNTROB	2	LRFN3	1	NSMCE1	1	GOT2	3	EPHB2	2	CPSF1	1	RAB22A	1	KCTD13	1
NME2	5	LSR	2	ILF3	2	DND1	1	STX10	1	GCDH	3	POMZP3	2	STK25	1	GMEB2	1	SUGP2	1
OGFR	5	TRAPPC6A	2	ACIN1	2	CPSF3L	1	GPC1	1	MRPS18C	3	PCMTD2	2	PPP1R15A	1	ARFRP1	1	ARHGEF2	1
UQCR10	5	TMC4	2	NFKBIL1	2	POU2F1	1	GEMIN4	1	TSFM	3	STX16	2	ATG4B	1	YTHDF1	1	DENND4B	1
MKNK2	5	PPM1H	2	CC2D1B	2	MRPL10	1	DDX41	1	COX15	3	ARFGAP1	2	DDA1	1	OSBPL2	1	EPN1	1
SNRPD2	5	FAAH	2	SLC39A3	2	FASTK	1	GLRX2	1	CNOT7	3	FZR1	2	CENPT	1	PHACTR3	1	TMEM201	1
RPL36A	5	BOLA1	2	NSUN5P1	2	PPP1R14D	1	TAF6	1	TXN2	3	CD2BP2	2	TBC1D22A	1	DNAJC5	1	ZMIZ2	1
ALDOA	5	MYH14	2	PUS1	2	RASSF1	1	MECR	1	PIN4	3	FBRS	2	SDC1	1	CSTF1	1	DDX11	1
SSR4	5	GSTK1	2	TRABD	2	PIK3R2	1	TRMT1	1	ZFP36	3	EPS8L2	2	IL18	1	LIME1	1	SETD1A	1
AKR1A1	5	CRB3	2	ZMYND19	2	DDX27	1	TMEM154	1	TUBB4B	3	NET1	2	PITPNM3	1	PFDN4	1	MESP1	1
DYNLL1	5	SLC35D2	2	VARS	2	NISCH	1	LSM6	1	RER1	3	SART1	2	SPRYD7	1	SAMD10	1	SMG9	1

RPL22	4	BAIAP2L1	2	QTRT1	2	SLC6A9	1	BET1L	1	MAZ	3	TEX264	2	GTPBP1	1	ZNF217	1	ABCC10	1
ZBTB48	4	MVP	2	SNHG3	2	ZNF579	1	SRPK3	1	UBE2M	3	ZMYND8	2	DENND1A	1	SLC9A8	1	SCAF1	1
KRT8	4	STX8	2	ZNF358	2	DGKQ	1	PRPF31	1	PPP4C	3	ZGPAT	2	TOM1L2	1	PMEPA1	1	ANKRD13D	1
TJP3	4	ARPC1A	2	RANBP3	2	RPUSD3	1	SPNS2	1	SDC4	3	FAM83H	2	TRAPPC1	1	CTSZ	1	LEMD2	1
ATP1B1	4	STAP2	2	PHKG2	2	ANKZF1	1	EBPL	1	PSD4	3	FA2H	2	PML	1	UBE2V1	1	DMWD	1
LGALS3BP	4	SULF2	2	MOGS	2	TBL3	1	MRPS6	1	IFITM1	3	CANT1	2	RALGDS	1	ABHD16B	1	SLC4A11	1
CD44	4	SQRDL	2	NUP214	2	METRN	1	APEH	1	ZBTB7A	3	TSPAN8	2	ALDH16A1	1	TAF4	1	EVI5L	1
HNRNPU	4	S100A13	2	TAF1C	2	PKMYT1	1	TACO1	1	RNH1	3	DOHH	2	AGAP3	1	RNF114	1	MSTO1	1
RPL35	4	RPL23A	2	HDAC10	2	SLC9A3R2	1	HMOX2	1	AURKAIP1	3	FOXQ1	2	SH3BGRL3	1	TMEM189	1	HAUS8	1
CLTA	4	RPS24	2	SH3BP2	2	UNKL	1	DLEC1	1	FLAD1	3	ACYP1	2	SH3GLB2	1	SLCO4A1	1	FBXL19	1
RPS17	4	ATP13A2	2	ZNF524	2	CCDC78	1	PDXK	1	RABEP2	3	CFDP1	2	CCM2	1	ADRM1	1	KAT8	1
RPS9	4	UBA7	2	UNC13D	2	NOXO1	1	FLII	1	FAM110A	3	FKBP2	2	KEAP1	1	STK4	1	FXR2	1
EEF1G	4	RPS15	2	CDK3	2	LMF1	1	FAHD2A	1	ARFIP2	3	CDK5	2	MIER2	1	TCFL5	1	PPRC1	1
RPL14	4	RPL11	2	RTKN	2	NARFL	1	MRTO4	1	RAB40C	3	RPL19	2	PCSK7	1	PKP4	1	GLTSCR1	1
RPS6	4	ATIC	2	CLIC1	2	EME2	1	FAM50A	1	DDR1	3	SF3A2	2	ALS2CL	1	OTUD3	1	NOL8	1
RPL13A	4	CIRBP	2	PDCD11	2	AXIN1	1	PITRM1	1	S100A14	3	EIF4EBP1	2	NPM2	1	WDR33	1	SPPL2B	1
RPL24	4	ITPK1	2	EIF1AY	1	MSLNL	1	RAC1	1	CDH3	3	PARK7	2	CLK3	1	L3MBTL1	1	SZT2	1
UBA52	4	H3F3B	2	KDM5D	1	TMEM8A	1	MCL1	1	IGFBP2	3	FKBP3	2	TBC1D2	1	YIPF2	1	SNAPC4	1
FBL	4	KDELR1	2	USP9Y	1	RHBDL1	1	KPNB1	1	MYO5B	3	PSME2	2	ATG4D	1	KLHL26	1	CRYGS	1
RPL3	4	TRIM28	2	UTY	1	PIGQ	1	CUX1	1	CBLC	3	FDPS	2	DAPK3	1	XAB2	1	CYP2D6	1
ACTB	4	RPL27	2	ZFY	1	ZNF205	1	PRPSAP2	1	SSH3	3	PGLS	2	S100A16	1	CHERP	1	COPS7B	1
NEDD8	4	SERPINB6	2	PRKY	1	FAM173A	1	ECH1	1	BSPRY	3	ENO3	2	PAX8	1	DCAF15	1	ABCA7	1
RPS21	4	NFIB	2	DDX3Y	1	CLCN7	1	TMEM205	1	ESRP2	3	HES1	2	PHLDA3	1	LCMT1	1	PICK1	1
RPL31	4	S100A2	2	NUCB1	1	PRSS27	1	CD320	1	OVOL2	3	CEBPB	2	BTBD2	1	MED16	1	GGA1	1
RPL38	4	KRT6B	2	MIF	1	PRSS22	1	ACYP2	1	HOOK2	3	BLOC1S1	2	RNF187	1	EIF5B	1	MICALL1	1
PPIA	4	CLTB	2	CTSA	1	RPUSD1	1	RBM3	1	ITGA3	3	DVL1	2	RHOG	1	NOP58	1	GATAD2A	1
RPL6	4	SNHG6	2	MRPS7	1	PDIA2	1	PSMG4	1	SLC16A3	3	PKN1	2	SLC2A1	1	NANS	1	ZNF692	1
SERPINB5	4	GAS5	2	DHCR7	1	TSC2	1	PIGX	1	MRPL27	3	RGS14	2	ARHGAP4	1	EIF2B5	1	FBRSL1	1
RPL27A	4	RPS27A	2	ATP6V0C	1	CHTF18	1	HDDC2	1	EVPL	3	GDI1	2	SMPD3	1	ARRDC2	1	MYPOP	1
FAU	4	RPL15	2	FCHO1	1	PKD1	1	CETN3	1	NARF	3	SF3B2	2	TP53I13	1	USMG5	1	GIGYF1	1
MDH2	4	RPS7	2	ALKBH6	1	SLC7A5	1	DBNL	1	DUS1L	3	SAC3D1	2	CISH	1	KRTCAP3	1	PPP1R12C	1
MRPL17	4	RPL26	2	ORAI1	1	DGAT2	1	PEX7	1	NFE2L1	3	NDUFAF3	2	KCNK7	1	IL20RA	1	FURIN	1
TXNRD2	4	BLCAP	2	TFCP2L1	1	LCN2	1	NAA38	1	PRR15L	3	NUDC	2	CES4A	1	SIRT7	1	SH3BP1	1
TOMM34	4	AKAP1	2	SLC39A11	1	MSX2	1	SF3B5	1	TOB1	3	RNPS1	2	KCTD17	1	CDC42EP5	1	L3MBTL2	1
POR	4	DGCR14	2	ZSWIM4	1	GRM8	1	LAGE3	1	RPS6KA4	3	RBM10	2	SREBF1	1	SURF6	1	TMEM86B	1
LONP1	4	MTOR	2	SNX15	1	ETV4	1	B9D1	1	ADAP1	3	RAP1GAP2	2	UBE2O	1	FUT2	1	SUV39H1	1
ECI1	4	ACAA1	2	CORO2A	1	CHI3L1	1	TOMM5	1	KDM4B	3	STOML1	2	WDR81	1	RRBP1	1	GRIPAP1	1
MRPL4	4	RPAIN	2	DBNDD2	1	AP1S3	1	TSEN34	1	MSLN	3	SLC22A18	2	MAP2K3	1	BACE2	1	DOT1L	1
HK1	4	PRPF6	2	PPA2	1	FREM2	1	COMTD1	1	GFER	3	ATP1A1	2	PLEKHM1	1	INF2	1	HSF4	1
BAK1	4	IMP3	2	HTATIP2	1	PF4	1	HDGFRP2	1	HAGH	3	SLPI	2	ATP6V0E1	1	EFHD2	1	ATAD3B	1
MRPL9	4	HLCS	2	MRPS21	1	UGT1A6	1	VOPP1	1	AXIN2	3	PDZK1IP1	2	ZDHHC14	1	NADSYN1	1	RBM33	1
BDH1	4	UROS	2	VILL	1	SLC22A3	1	JOSD2	1	DRG2	3	KRT15	2	VAV2	1	CC2D1A	1	MIIP	1
IVD	4	MLYCD	2	PYROXD2	1	DUOX2	1	LINC00152	1	SHMT1	3	MAPKAPK3	2	GDPD5	1	ANKRD9	1	ANKRD54	1
MRPL34	4	HPS1	2	SDSL	1	FAIM2	1	UNC45A	1	DLGAP4	3	PLXNB1	2	FCHSD1	1	UNC5CL	1	ZNF580	1
MRPS34	4	ACP6	2	PRKCZ	1	UCA1	1	HNRNPA3	1	GSS	3	B3GAT3	2	ZFYVE28	1	TMEM129	1	PHPT1	1
MRPS11	4	TFB1M	2	GRTP1	1	GPSM2	1	SNHG11	1	PSMD13	3	MVD	2	HPX	1	DHTKD1	1	CUL9	1
MRPS15	4	MRPS27	2	CGN	1	CLDN2	1	SNHG10	1	MARCKSL1	3	DPEP1	2	TTLL3	1	GMDS	1	CUL7	1
TIMM13	4	TIMM44	2	ATG2A	1	CLDN1	1	PEX16	1	NR2F6	3	GAS8	2	STK10	1	RPL26L1	1	SAFB2	1
DUT	4	PPOX	2	DHRS4	1	LRRC36	1	PODXL2	1	ICT1	3	NUP93	2	ZBTB17	1	PHKA2	1	WASH3P	1
TSPO	4	TIMM8A	2	THNSL2	1	LRRC34	1	GALT	1	PDAP1	3	NPRL3	2	DNHD1	1	HEXIM1	1	PTBP1	1
COX10	4	ZNF33B	2	AP1G2	1	SRPX2	1	WDR83	1	MRPL36	3	D2HGDH	2	NSUN5P2	1	APEX2	1	REXO1	1
SLC25A1	4	HTATSF1	2	PLEKHG3	1	FABP6	1	MEX3D	1	CLPTM1L	3	POLR2E	2	WDTC1	1	TARBP1	1	TRAF2	1
NDUFA9	4	PYCR1	2	COBL	1	RPL22L1	1	OSBPL5	1	ATP5G3	3	HMG20B	2	ABHD15	1	PIAS4	1	RANGAP1	1
MRPS33	4	CASP8	2	HDAC11	1	SLCO1B3	1	PPIL6	1	UQCRFS1	3	SH2B2	2	PCNXL2	1	SNRPD1	1	DAGLB	1
NDUFA10	4	SUPV3L1	2	SIL1	1	TNFRSF10C	1	PPP2R2A	1	KARS	3	CAPN1	2	NHLH1	1	POLA2	1	TMCC1	1
NDUFB3	4	MRPL20	2	PPL	1	NFE2L3	1	HSD11B1L	1	POLR2I	3	ZNRD1	2	EFTUD2	1	TAX1BP3	1	MAPRE3	1
BCL2L1	4	MRPL24	2	ZFAND2B	1	DSG3	1	OCEL1	1	ENGASE	3	LMAN2	2	TMEM102	1	PEX14	1	RBM15B	1
ATPIF1	4	LYPLA2	2	ZDHHC24	1	CCNO	1	ZNF814	1	DNAJB2	3	CDC34	2	REC8	1	TACC3	1	ARHGAP17	1
POLRMT	4	MRPL46	2	RHBDD2	1	ART3	1	HES4	1	CNPPD1	3	ALDH7A1	2	PHF7	1	PNPLA6	1	CLASRP	1
BCAT2	4	ME3	2	PGAP2	1	DUOXA2	1	PUSL1	1	EDF1	3	H3F3A	2	CATSPER2	1	ZNF84	1	KLF16	1
BAX	4	SLC25A14	2	RFX1	1	SH3TC2	1	SYMPK	4	CHMP1A	3	CDC25B	2	HSPB9	1	TCF7	1	RNF207	1
HDGF	4	SLC25A37	2	NGEF	1	GALNT6	1	SYTL1	4	DGUOK	2	POLD1	2	NOC4L	1	CDK11A	1	ABCF3	1
JTB	4	ACADS	2	SLC5A1	1	RAB8A	1	ELMO3	4	ACADVL	2	LYPLAL1	1	ANKLE2	1	GNL1	1	POLM	1
SYNGR2	4	ACAD8	2	SIRT5	1	PHYH	1	PKP3	4	ALDH2	2	TMEM54	1	PRMT1	1	ZNF330	1	NEURL4	1
CSNK2B	4	TIMM22	2	DDX51	1	TERF2IP	1	S100P	4	MCAT	2	PCBD2	1	CCL20	1	AUP1	1	CDCA3	1
VSNL1	4	AMT	2	CHFR	1	HTT	1	SPINT2	4	MTX2	2	MBOAT1	1	PHLDA1	1	CCT7	1	PSME1	1
HRAS	4	FXR1	2	GOLGA3	1	ZNF212	1	CDH1	4	SLC25A22	2	PTPN6	1	RNF183	1	HIST2H2AC	1	OAZ1	1
TNPO2	4	ENO1	2	TRIM46	1	TROAP	1	ELF3	4	CAPNS1	2	SLC25A35	1	TNS4	1	STK19	1	SSBP1	1
RALY	3	HSD17B10	2	ADAM15	1	E4F1	1	RAB25	4	TUBA4A	2	TMEM41A	1	SLC6A14	1	PITX1	1		
PPDPF	3	DEAF1	2	SLC50A1	1	MBOAT7	1	CD24	4	TUBB3	2	PEX11G	1	ACSL6	1	SLC25A26	1		

Supplementary Table 2.
GSEA, leading edge and IPA pathway analyses performed on tumor samples from a selected TCGA-COAD cohort stratified according to miR-27a levels.
2E) the number of gene-sets (core-gene sets) enriched in low-miR-27a CRCs where a gene was found overlapping (in at least 5 gene-sets);

Symbol	#shared genesets	Symbol	#shared genesets	Symbol	#shared genesets	Symbol	#shared genesets	Symbol	#shared genesets	Symbol	#shared genesets	Symbol	#shared genesets	Symbol	#shared genesets	Symbol	#shared genesets	Symbol	#shared genesets
NRP1	35	PLK4	11	FEZ1	8	LAP3	6	VPS13A	4	SIGLEC5	3	PATL1	3	CACYBP	2	TEF	1	ADH1A	1
SPARC	33	RBL1	11	CHN1	8	TBC1D9	6	PAK1IP1	4	TET2	3	PAPSS2	3	LSAMP	2	ASB3	1	SH3BGR	1
TGFBR2	32	PPP1R16B	11	FBLN5	8	KIF5C	6	CSNK1A1	4	FFAR2	3	FGF2	3	CHAC2	2	GBP5	1	CCL14	1
CCL2	31	SGMS2	11	COL1A1	8	FAM134B	6	EEA1	4	SSTR2	3	ZNF180	3	PLA2G4C	2	ZC3H12D	1	SEMA3D	1
FN1	31	CADM1	11	SPON2	8	RPS6KA3	6	ATM	4	CLEC12A	3	EAPP	3	TEAD2	2	ZBTB2	1	HTRA3	1
CALD1	31	DDX60	11	LAMA2	8	XK	6	SUSD5	4	TMEM144	3	GTF2B	3	RBP5	2	EXOC2	1	MRGPRF	1
EMP1	30	DDX58	11	GCNT2	8	PRKD1	6	ARRB2	4	PRSS16	3	MRFAP1L1	3	ROR2	2	TAF8	1	CDYL2	1
DAB2	30	CHST2	11	ANK2	8	FRZB	6	IL4I1	4	TIFAB	3	TFAP2C	3	NUP62CL	2	ZFP91	1	RALGAPA1	1
IL7R	29	EIF5	11	TMEM47	8	TXNRD1	6	TMTC2	4	BRD2	3	PPT1	3	FXYD6	2	PARP11	1	PYGO1	1
CXCR4	29	CD302	11	SEC24D	8	NF1	6	KLHL5	4	DCLRE1C	3	MFN1	3	KIRREL3	2	POLI	1	ZDHHC15	1
TOP2A	29	UTRN	11	FAT4	8	COPS8	6	PPM1K	4	NT5DC2	3	CBLL1	3	RSPO2	2	TTC5	1	RNF150	1
MMP9	28	HIVEP2	11	RUNX2	8	HNRNPR	6	SPI1	4	STEAP4	3	ASH2L	3	GNAO1	2	ZNF347	1	EIF4E3	1
NR3C1	28	RAB8B	11	CAT	8	HSPA5	6	RNASE2	4	PELI2	3	BAG4	3	RBPMS2	2	RAB19	1	AKAP6	1
GPNMB	27	FAM69A	11	HSD17B11	8	ITGA1	6	EVL	4	USP51	3	BLMH	3	NCKAP5	2	NUS1	1	ANGEL1	1
COL5A2	27	GPR183	11	PXDN	8	MAP2K4	6	SPATA13	4	TMOD2	3	FBXO11	3	LRRC4C	2	PRRG4	1	DOCK3	1
COL3A1	27	TRAT1	11	PER3	8	EXTL2	6	SLFN5	4	GAPT	3	CCDC90B	3	ANGPTL4	2	PTDSS1	1	RAB3IL1	1
TIMP3	27	TNF	11	ESR1	8	CCDC50	6	HAVCR2	4	PTENP1	3	KIDINS220	3	SLC43A1	2	GLB1L	1	ELFN1	1
CXCL10	26	MMP12	11	SRSF1	8	MTM1	6	MAGEH1	4	NCALD	3	CHMP3	3	CA5B	2	ZNF454	1	ALPL	1
SGK1	26	C1QC	11	UBE2D1	8	RSRC2	6	PTPRR	4	RAMP2	3	KIAA0355	3	KBTBD7	2	MOSPD2	1	NYNRIN	1
CYBB	26	SLAMF8	11	UBE2V2	8	RNF2	6	COX7A1	4	PLEKHA1	3	PON2	3	PCDHB15	2	SLC35D1	1	TSPAN6	1
SULF1	26	SPHK1	11	ZYG11B	8	TIA1	6	EPHA5	4	FEZ2	3	FUBP3	3	ZNF618	2	KY	1	PAX9	1
COL1A2	26	ASAH1	11	CLIP4	8	CTDSP2	6	ATP8A2	4	FOXF2	3	DHX29	3	LRIG3	2	CC2D2A	1	RTN4RL1	1
VCAN	26	SGMS1	11	SLC44A1	8	PSAP	6	ASB2	4	GPX7	3	SENP6	3	CD164	2	FAM81A	1	PHOSPHO1	1
PDGFRA	26	MCTP1	11	MAGT1	8	XRCC5	6	OLFM3	4	EFS	3	IDE	3	TRPC6	2	CBX7	1	IL10RB	1
ALCAM	26	ADAMDEC1	11	FUBP1	8	PJA2	6	RAB27B	4	SLC24A3	3	DENND4C	3	SNTA1	2	CASS4	1	GP1BA	1
PALLD	26	TNS3	11	ZNF22	8	NFYC	6	XAF1	4	NAPSB	3	RAB23	3	GGH	2	CCL4L2	1	FAM20B	1
IFIT1	25	MAN2A1	11	ARNT	8	ROCK2	6	PYROXD1	4	CLECL1	3	MAP4K3	3	PKIA	2	SLC2A14	1	LDB1	1
TNFAIP3	25	PLXNC1	11	TRAK2	8	GNAQ	6	AVL9	4	SIGLEC6	3	COMMD10	3	NUP50	2	PRUNE	1	PTPN4	1
MAF	25	PTEN	11	SRPK2	8	NUMB	6	APOL3	4	ADCYAP1	3	LAPTM4A	3	PNMA2	2	ORC1	1	P2RY12	1
FGL2	25	NOV	11	MEF2A	8	STARD13	6	BTN2A1	4	REXO2	3	SLC35A5	3	OGN	2	PILRA	1	GPR21	1
EVI2A	25	TWSG1	11	PBX3	8	TRIM44	6	FBXO33	4	UST	3	GIN1	3	ADH1B	2	HCAR3	1	USP32	1
MARCKS	25	CALCRL	11	FOXN3	8	CAMSAP2	6	METAP2	4	ANO1	3	CLINT1	3	MYH11	2	CARD9	1	PDCD4	1
PLSCR1	25	CD37	11	MAPK6	8	GDA	6	NCBP1	4	GRHL2	3	DERA	3	SLC16A7	2	PALB2	1	CFD	1
IFIT3	24	HCK	11	LUZP1	8	HOMER1	6	FRS2	4	CNST	3	MKLN1	3	RPS6KC1	2	LILRA6	1	FOSB	1
C3AR1	24	CSF2RB	11	GK	8	USP12	6	AHCYL1	4	NRGN	3	ST7	3	NR3C2	2	SIRPA	1	MAP3K12	1
CREM	24	NKG7	11	REL	8	DLAT	6	RAPGEF4	4	ZNF331	3	HSD17B6	3	MAP7D2	2	BUB1B	1	DHRS2	1
SRGN	24	CD2	11	TRA2B	8	TIAL1	6	RAP1B	4	IKZF5	3	SPTSSA	3	ACE2	2	REST	1	HMGCS2	1
LUM	24	MOXD1	11	ABCG2	8	OGFRL1	6	IMPA1	4	MYNN	3	TRIM34	3	CGNL1	2	TLR2	1	GFRA1	1
ENPP2	24	FAM126A	11	RNF19A	8	TMEM65	6	GPRIN3	4	YRDC	3	SCFD1	3	SPG20	2	LRRC49	1	ATRNL1	1
COL4A1	24	EDNRA	11	SESN3	8	PM20D2	6	NR5A2	4	BRCA2	3	TM9SF2	3	SDPR	2	DIEXF	1	PTHLH	1
FERMT2	24	NUAK1	11	BASP1	8	SYTL2	6	HK3	4	EAF2	3	CTCF	3	RNF180	2	SH2D1B	1	CIDEC	1
GBP1	23	COL6A1	11	ZNF704	8	SORBS2	6	ANKRD44	4	NRIP3	3	KIAA1524	3	GPR155	2	FGFBP2	1	MMP3	1
IL6	23	COL6A3	11	SMC3	8	TLE1	6	RAPGEF6	4	PARVG	3	TOPORS	3	NDNF	2	PYHIN1	1	ANKRD29	1
CCR1	23	WISP1	11	OXR1	8	PTGIS	6	RFX3	4	PGM3	3	ZCCHC11	3	BMP3	2	HAPLN3	1	SPESP1	1
IL6ST	23	GPC6	11	KIAA1033	8	ZKSCAN1	6	STAT5B	4	ACSBG1	3	SPOP	3	PLA2G12B	2	IL12RB1	1	SLC36A4	1
TFPI	23	RBPMS	11	MS4A7	8	SOS2	6	CDK19	4	OLIG2	3	PUM1	3	LAMA1	2	CCR3	1	MT1H	1
F13A1	23	IGF1	11	CDC5L	8	PARP9	6	TNFSF11	4	ARF3	3	SP4	3	NDN	2	VSTM1	1	LOC400043	1
PRNP	23	PTN	11	SMCHD1	8	FAM171B	6	FAM91A1	4	ARL6	3	GPLD1	3	NRXN1	2	INADL	1	BNIP3	1
CD48	23	NR2F2	11	MBNL2	8	UACA	6	ANKRD55	4	TLR8	3	PTPRF	3	SCG2	2	SLFN13	1	WIPF3	1
POSTN	23	GADD45B	11	ZC3HAV1	8	ENPP4	6	ZDHHC20	4	CORIN	3	CTAGE5	3	VIP	2	TMEM45B	1	RASSF10	1
VCAM1	23	ITIH5	11	MED13L	8	PTAR1	6	PRTG	4	CD200R1	3	PCYOX1	3	LINC00341	2	ACE	1	RIMS3	1
APOE	22	PTGER3	11	PNO1	8	SSX2IP	6	MARK1	4	GIMAP1	3	POU2F2	2	PKIB	2	CHIT1	1	MAL	1
TNFAIP6	22	LHFP	11	PDE4D	8	MR1	6	GNPDA2	4	GTDC1	3	WNT8B	2	SYNPO2	2	OSM	1	ZNF385B	1
CTSC	22	PDGFC	11	RALGPS2	8	ZAK	6	EHD3	4	HERPUD2	3	FGF10	2	KIAA2022	2	SETD1B	1	BEX1	1
PTPRC	22	DSE	11	WARS	8	RSBN1	6	EGFL6	4	EXOC6B	3	CALM3	2	CNN1	2	ST6GALNAC5	1	SULT1B1	1
C1S	22	LBH	11	G3BP2	8	MORC3	6	GABARAPL1	4	NSMCE2	3	BHLHE22	2	ANGPTL1	2	CST1	1	SYT4	1
EFEMP1	22	ACTN1	11	DPY19L1	8	RAB14	6	ERMAP	4	HEATR5A	3	ARCN1	2	PLP1	2	P2RY1	1	RAB9B	1
IL1R1	22	FNDC3B	11	UHMK1	8	RAD1	6	SPATA6	4	BTBD10	3	SPAST	2	CDH19	2	ZNF225	1	NLRP7	1
RECK	22	RNASE1	11	TMOD3	8	STAG2	6	CD163	4	SLAMF6	3	GUF1	2	SFRP1	2	METTL14	1	BMPR1B	1
EGFR	22	APOD	11	ZMAT3	8	PLS1	6	CEBPE	4	SMAP2	3	GINS4	2	VSTM2A	2	MRPL19	1	KCNMB2	1
THBS1	22	PDLIM3	11	DNAJB14	8	PNPT1	6	NDRG2	4	CAMK4	3	ZRANB1	2	LYVE1	2	PRMT5	1	GPR27	1
TMPO	22	EDNRB	11	MAX	8	MTMR6	6	SNX18	4	GPR180	3	FLVCR1	2	EVC	2	CBFB	1	KIAA1324	1
ITM2A	21	AGPS	11	RFC5	8	EIF4E	6	FAM26F	4	DNAJA4	3	SPPL2A	2	CYGB	2	DCTN2	1	SLC1A2	1
C1QB	21	PTGS1	11	SCML1	8	ZNF292	6	CCL23	4	ERCC8	3	ALDH9A1	2	TMEM121	2	FES	1	FCRL5	1
C1QA	21	NR2F1	11	CASC5	8	RCHY1	6	SLC38A6	4	GPR34	3	ZMPSTE24	2	KDELC1	2	SIGLEC9	1	TPCN2	1
FYN	21	ZNF148	11	SGOL2	8	PAQR3	6	CLEC6A	4	FRMD5	3	ISOC1	2	LCA5	2	MGAT1	1	KLF9	1
PLA2G7	21	GBE1	11	MAP2K1	8	SLC6A8	6	MYD88	4	PEAR1	3	LRP6	2	NGFR	2	CASP5	1	PBLD	1
CRIM1	21	STK3	11	TDG	8	DENND1B	6	DPEP2	4	TRAF1	3	BTC	2	AGPAT4	2	AP3S2	1	PLCXD3	1
RAB31	21	DLC1	11	SERTAD2	8	GATA2	6	CTBS	4	CEP112	3	LUC7L2	2	HS3ST3A1	2	ARHGEF17	1	FXYD1	1
CCR2	21	MYBL1	11	SSH1	8	TSPYL5	6	ZNF706	4	TYMP	3	ANKRD1	2	FAS	2	YPEL1	1	TEX11	1
CDH11	21	CH25H	11	SMURF2	8	FLNA	6	FDX1	4	ZNF518A	3	DYSF	2	SCN4B	2	SIDT1	1	PRKAA2	1
DOCK4	21	DAAM1	11	TERF1	8	SERINC1	6	STS	4	DCAF12	3	S100A8	2	PTPRT	2	PALM	1	TM6SF2	1
PRKAR2B	21	PIK3R3	11	MED21	8	STX7	6	CCDC69	4	RBP7	3	PCDHB10	2	RAPGEF1	2	CHM	1	SST	1
IGFBP7	21	PAWR	11	SMARCC1	8	ATP6AP2	6	PDIA5	4	PPM1L	3	SHQ1	2	CHST7	2	BRPF3	1	LGALS2	1
ATP2B1	21	RIF1	11	ITSN2	8	SMARCA2	6	IL5RA	4	KLHDC1	3	RAB2B	2	MBLAC2	2	ZNF518B	1	CES2	1
IFIT2	20	KANK1	11	NCOA1	8	TMED10	6	HVCN1	4	CD163L1	3	FAT3	2	GNAL	2	AQP11	1	PCK1	1
MAFB	20	PPFIA1	11	PRKCI	8	MXRA8	6	OLIG1	4	HS3ST2	3	XRN2	2	NUP155	2	FBXO4	1	SPINK5	1
CD14	20	CCNC	11	PUM2	8	TCF21	6	SLCO2B1	4	HS3ST1	3	COLEC10	2	LRRK1	2	ZNF582	1	CNTN4	1
LCP1	20	RORA	11	ADCY9	8	FRY	6	GJA4	4	SNX6	3	MTBP	2	SMAD9	2	ZNF304	1	CA2	1
F2R	20	HMMR	11	FOXJ3	8	FGF1	6	PHF6	4	SLC37A2	3	KCNQ3	2	NECAP2	2	FLCN	1	SLC4A4	1
FYB	20	CENPA	11	FOXO1	8	BMP7	6	CKAP5	4	OAZ2	3	VAT1	2	SLC33A1	2	SPCS3	1	SLC17A4	1
FBN1	20	NUSAP1	11	AKAP9	8	RCAN1	6	GCLM	4	CALHM2	3	SGOL1	2	COPB2	2	LIG3	1	BCHE	1
ARL4C	20	PLS3	11	RNF13	8	RGS10	6	YIPF6	4	TRIT1	3	SKA2	2	SERP1	2	RNF219	1	USP2	1
QKI	20	SLC16A1	11	APPBP2	8	SACM1L	6	TNIP3	4	ELOVL7	3	TRPM7	2	TSPAN4	2	LRRC16A	1	GBA3	1
LCP2	20	SSFA2	11	ATF2	8	LBR	6	RAB9A	4	PADI4	3	CNTLN	2	MON2	2	IL9R	1	EDN3	1
NRIP1	20	GNG12	11	REV3L	8	DBF4	6	NETO2	4	MS4A2	3	PKIG	2	TBCCD1	2	TRPS1	1	GDPD2	1
RRM2	20	PTPN11	11	BAZ1A	8	CRY1	6	CRYZ	4	STAC	3	IL17D	2	AREG	2	TMEM25	1	THRB	1
SDC2	20	EZH2	11	RB1CC1	8	CD180	6	ADRBK2	4	ADORA3	3	RNGTT	2	SCOC	2	ELK1	1	MFSD4	1
MBNL1	20	ASAP1	11	VPS13B	8	GGA2	6	MPHOSPH6	4	RAB30	3	OSBPL10	2	TMEM43	2	GSTCD	1	C7	1
MAN1A1	19	IDH1	11	PIK3C2A	8	ANKRD46	6	NPC2	4	DYNC1LI1	3	GM2A	2	TNFAIP8L2	2	EIF4A3	1	FAM13C	1
ETS1	19	SLBP	11	MAPK14	7	BICD2	6	COL8A2	4	TMEM184C	3	TUBGCP5	2	LRMP	2	EXD2	1	CNNM2	1
TIMP2	19	POLE2	11	NBPF10	7	MAP3K2	6	EHD2	4	PDCD6IP	3	PSME3	2	RICTOR	2	TTC17	1	SLC25A34	1
TFRC	19	EPS15	11	USP15	7	SERINC3	6	VAMP5	4	ATP6V1H	3	S1PR5	2	DIRAS3	2	FGD2	1	EPHA7	1
G0S2	19	WEE1	11	RNF125	7	YTHDF3	6	PDE3B	4	EIF2B3	3	SLC7A3	2	CCT2	2	HIST2H2BF	1	SI	1
BIRC3	19	KAT6A	11	SMARCA1	7	EDEM1	6	RGS19	4	TFG	3	CIB2	2	SLC30A9	2	DYM	1	CDHR5	1
GEM	19	MPHOSPH9	11	DAPK1	7	FCHO2	6	HCST	4	FOXJ2	3	FGD5	2	CLCN5	2	ACRBP	1	CHGA	1
LAPTM5	19	VLDLR	11	ARHGEF6	7	MOSPD1	6	SNRPN	4	CD209	3	PTCRA	2	CHRM3	2	FAM175A	1	GLDN	1
LYN	19	CAP2	10	GSTM3	7	MCM7	6	ATP7A	4	CYB5A	3	NOTCH3	2	GPR22	2	ZNF573	1	HOXD10	1
SRPX	19	NUP153	10	ZNF207	7	FBXO5	6	FLVCR2	4	PSMD2	3	SLA2	2	SYT14	2	ZNF555	1	GUCA2A	1
THBS2	19	IDI1	10	KLRK1	7	ACER3	6	CTNND1	4	CAPZA1	3	P2RX7	2	ZDHHC2	2	CD101	1	KCNIP4	1
TPM1	19	CHEK1	10	LYL1	7	CDK8	6	LTA	4	STAT6	3	TCEA2	2	FSTL5	2	ATF7	1	NPY	1
CXCL12	19	FGFR1	10	GUCY1B3	7	C2CD2	6	CD40LG	4	SIGLEC7	3	TMEM119	2	LIMCH1	2	ZNF429	1	STAP1	1
COL4A2	19	CD93	10	RHOJ	7	BMPR1A	6	ACAN	4	CD1A	3	CSRNP2	2	LRP4	2	ANKRD20A8P	1	PDE6A	1
OSMR	19	SLCO3A1	10	NEGR1	7	UFL1	6	SLC38A2	4	GNB5	3	TAGAP	2	CPEB1	2	TBC1D3G	1	AKAP12	1
CITED2	19	IFIT5	10	XYLT1	7	SEC23IP	6	AFF4	4	EGFL7	3	ARHGAP9	2	CNTN1	2	ZNF366	1	HSD17B2	1
IL13RA1	19	HERC5	10	CCR6	7	PRKDC	6	MEF2D	4	NRXN2	3	DOCK8	2	RSPO3	2	FRRS1	1	SCNN1B	1
PMAIP1	19	HERC6	10	TSLP	7	G3BP1	6	TRAF6	4	CLIP3	3	CCDC152	2	LRRC8B	2	HERC2P4	1	ALPI	1
CDC42EP3	19	FOSL2	10	OAS2	7	H2AFV	6	FBXL3	4	TMEM204	3	HAUS2	2	ARHGAP6	2	SPATS2	1	OR51E2	1
DDX3X	19	ARHGEF3	10	LY6E	7	SMAD4	6	HAUS3	4	NDST1	3	TTLL7	2	GFOD1	2	LRRC37A	1	TRPM6	1
ITGB1	19	JMJD1C	10	CSGALNACT2	7	BET1	6	GZMH	4	CRLF3	3	ZBTB46	2	ADO	2	RASGRP4	1	CHRNA1	1
CCNE2	19	STX11	10	RNF11	7	TAF9B	6	KDM2A	4	ST8SIA6	3	IPCEF1	2	SEMA3G	2	SIRPB2	1	MYOT	1
NID1	18	ALOX5	10	TUBB2A	7	FBXO3	6	DCP2	4	USP14	3	ENPP6	2	ARHGDIB	2	BTN2A2	1	TMEM72	1
RSAD2	18	TLR7	10	WTAP	7	AGGF1	6	CREBZF	4	SENP7	3	MCOLN3	2	COPS5	2	GBGT1	1	LRRK2	1
SEPP1	18	SLC11A1	10	BACH2	7	PKN2	6	CTR9	4	STAT2	3	ESRP1	2	CDK4	2	HEATR1	1	LRRN2	1
CCR5	18	MPEG1	10	CHSY1	7	U2SURP	6	CUL2	4	MEFV	3	MGA	2	YBX1	2	NSF	1	SECTM1	1
MS4A4A	18	SLC7A7	10	CTLA4	7	RYK	6	KLHL2	4	ZFAND1	3	TUBA1B	2	DCTN4	2	ATP6V0A2	1	SORCS1	1
ANXA5	18	TLR1	10	KDSR	7	ARMC8	6	TBK1	4	MANBA	3	FMO3	2	UBXN2B	2	DIP2C	1	ANO5	1
STK17A	18	CLEC5A	10	AKAP13	7	PRPF38B	6	MAML1	4	FOXN2	3	BPHL	2	MEAF6	2	ZNF627	1	SCN7A	1
LHFPL2	18	HSD11B1	10	GLCCI1	7	FGF9	6	USP47	4	BBS10	3	METAP1	2	TCP1	2	CCDC117	1	PRIMA1	1
INHBA	18	SPRED2	10	P2RY10	7	CD2AP	6	BIN2	4	TMEM55A	3	ANKRD40	2	PIK3CA	2	CDH20	1	SOX10	1
LTBP2	18	CD84	10	SLC23A2	7	AKAP10	6	ATL1	4	DMXL1	3	PCDH10	2	GNB1	2	FPR2	1	FMN2	1
SPP1	18	CD1E	10	RNF138	7	WNK1	6	NPR2	4	NCAM2	3	ADAM33	2	DEK	2	KRT222	1	FCRLA	1
TNFAIP8	18	ITGB3	10	SEMA4A	7	CDK2	6	SPEG	4	CLDND1	3	B3GALNT2	2	PTGR1	2	LAMB4	1	FCRL3	1
ANXA1	18	PAK1	10	P2RX5	7	FZD6	6	ANKRD6	4	RRAGC	3	CDH17	2	TM9SF1	2	A1CF	1	CCL19	1
ELL2	18	RCBTB2	10	NEU1	7	AGL	6	MID2	4	ENPP5	3	IL22RA2	2	PEX2	2	SIAH3	1	GPX3	1
RGS2	18	CPNE8	10	TREM2	7	TSNAX	6	STX12	4	RASSF9	3	MAP7	2	INTS8	2	SLC8A3	1	SCGN	1
IRF8	18	ENDOD1	10	SNX10	7	COPS2	6	RAB33A	4	TNFAIP1	3	TFDP2	2	RBM12B	2	NLRP14	1	PDZD3	1
SLA	18	ADAMTS1	10	LILRB2	7	ROCK1	6	ZNF678	4	ENTPD7	3	BATF3	2	UBE4B	2	TECTA	1	CHST3	1
CD53	18	DYRK2	10	LILRA2	7	BMI1	6	RMI1	4	TAF5L	3	CHRNA6	2	SOX5	2	XCR1	1	CCDC3	1
WIPF1	18	GMFB	10	NOTCH2	7	SCAF11	6	BARD1	4	ARL13B	3	LILRA5	2	LYSMD3	2	TRIM50	1	FBXO30	1
GNG11	18	SDCBP	10	MDM1	7	SLC9A6	6	LCORL	4	GCLC	3	RXFP1	2	SPG21	2	LDHAL6A	1	AHI1	1
GUCY1A3	18	SEC24B	10	PACSIN2	7	ZNF318	6	ZC3HAV1L	4	E2F8	3	AMMECR1	2	ZNRF2	2	XCL2	1	STXBP4	1
CAV1	18	RBMS3	10	RHOQ	7	ZNF623	6	WDR11	4	KLRF1	3	NAP1L5	2	DBT	2	MAGI1	1	ELOVL4	1
TCF4	18	HDAC9	10	AOAH	7	CREBBP	6	STRBP	4	FAM208A	3	APOBEC3A	2	TOR1B	2	ZNF2	1	CSDC2	1
DCN	18	EXOC5	10	PCSK5	7	NIPBL	6	TCEA1	4	CDKN2AIP	3	CMTM5	2	PDIA4	2	ZFP1	1	ARHGEF25	1
RBMS1	18	CSF1R	10	TSPAN2	7	BPTF	6	RSPRY1	4	EML4	3	TREML1	2	TMEM237	2	MARS2	1	BVES	1
EXT1	18	CD27	10	RAP1A	7	UBR5	6	SNX13	4	NIN	3	CYP19A1	2	FER	2	NOX1	1	GRASP	1
LPP	18	ACSL4	10	SLAMF7	7	SP3	6	ATAD2B	4	THBS4	3	HOXC8	2	GLI3	2	DIMT1	1	KCNIP3	1
IFNGR1	18	TCF12	10	EMILIN1	7	CNOT4	6	SFXN1	4	SH3PXD2A	3	CNIH3	2	CPSF2	2	KCNQ2	1	TSKU	1
STK17B	18	CPVL	10	MATK	7	PMS1	6	CNR1	4	ADAM23	3	MAP4K4	2	SLC35F5	2	TXK	1	PKNOX2	1
PLOD2	18	CLEC4A	10	ZFYVE16	7	TUBGCP3	6	SCAMP1	4	BST1	3	WDR61	2	STAMBP	2	CD160	1	NUDT10	1
XPO1	18	PDE8A	10	IL21R	7	TLK2	6	IRAK3	4	TCN2	3	TULP3	2	LEMD3	2	PTCH1	1	COL23A1	1
OAS1	17	GIMAP6	10	GPR137B	7	FAM179B	6	DNM3	4	FNIP2	3	TTC7A	2	CLPX	2	KLRD1	1	CSPG4	1
IL10RA	17	P2RY14	10	RAMP1	7	BICD1	6	ZFHX4	4	PDZD2	3	EPC1	2	CCAR1	2	APBA2	1	THSD1	1
CXCL9	17	LDHB	10	CCL22	7	ACAP2	6	TSHZ3	4	CD226	3	SMCR8	2	SCARF2	2	BCAS2	1	DZIP1L	1
FCGR2B	17	IGSF6	10	CD80	7	IMPAD1	5	DZIP1	4	DDX59	3	NFATC2	2	FAM168B	2	NFX1	1	DCUN1D3	1
AKT3	17	ZAP70	10	PPP1CB	7	PTPRB	5	MBNL3	4	ZNF250	3	CHORDC1	2	SMC6	2	SAP30L	1	RFX2	1
BCL2	17	LEF1	10	MFAP1	7	CTSO	5	ARHGAP20	4	WDR3	3	MMADHC	2	PAFAH1B2	2	ATG12	1	TTYH2	1
HMOX1	17	TRAF3IP3	10	MRC2	7	PPP2R5E	5	OAT	4	LRRC40	3	SRP72	2	ISG20L2	2	DUSP12	1	EFCAB1	1
CTSS	17	ARHGAP25	10	ARHGAP29	7	RUNDC3B	5	CHRNA5	4	NEO1	3	RBM12	2	FAM172A	2	FCN3	1	ACER2	1
ITGB2	17	DOCK2	10	LILRB3	7	OSBPL8	5	ZWILCH	4	FAM199X	3	POP1	2	RCBTB1	2	PRAM1	1	SCML2	1
KCTD12	17	PDK4	10	CD34	7	FBXO9	5	EPB41L4B	4	NAA30	3	HCCS	2	SNX3	2	EFR3B	1	RASIP1	1
CD3D	17	PID1	10	PTPN14	7	MIS18BP1	5	PNRC1	4	UBR3	3	CNTNAP2	2	HERC1	2	KIAA1324L	1	NOL9	1
PRKCB	17	GLIPR2	10	GSTA4	7	KLHL28	5	EIF4EBP2	4	RFX7	3	PRMT6	2	TRIM24	2	MMGT1	1	DHX33	1
SPOCK1	17	PRSS23	10	CTSG	7	AK2	5	PRPF4	4	VASH2	3	PEX1	2	GOPC	2	ZFYVE1	1	HRCT1	1
FHL1	17	TAGLN	10	ATF1	7	CASP8AP2	5	BACE1	4	GOLIM4	3	MTRR	2	NLRC4	2	VPS37D	1	BBS7	1
PKD2	17	DKK3	10	CUL5	7	DDX46	5	HIP1	4	FOXA1	3	AQR	2	USP3	2	ZNF385D	1	IFT74	1
TGFBR3	17	GAS1	10	NAP1L3	7	SLFN11	5	FAM98A	4	MACC1	3	CYSLTR1	2	GPBP1	2	DACT3	1	ORC4	1
FLRT2	17	MEIS2	10	RAP2C	7	BAALC	5	NLN	4	CBX6	3	SSH2	2	FYTTD1	2	KLHDC10	1	TTC30A	1
MATN2	17	SVEP1	10	HIST1H2BK	7	TMEM38B	5	BZW1	4	AMPD3	3	TMEM67	2	UPF2	2	CD81	1	ATG2B	1
HEG1	17	MEST	10	TFPI2	7	SELPLG	5	PGM2	4	APPL2	3	CR1L	2	ANKMY2	2	FAM69B	1	PDCD1	1
GJA1	17	CCDC80	10	ARRB1	7	GSTM2	5	OCIAD1	4	YPEL2	3	ZPBP2	2	AFAP1	2	SNX29	1	ST3GAL3	1
SNAI2	17	LEPR	10	KLF11	7	PLD4	5	PTPN12	4	FASLG	3	TLR6	2	PHF21A	2	TMEM167B	1	TMEM182	1

ITGAV	17	DPT	10	FGFR1OP2	7	ABCD2	5	PURA	4	ARHGAP28	3	ZBTB25	2	MPP6	2	ZSCAN29	1	HIST1H3E	1
CLIC4	17	FABP5	10	MYCBP2	7	PRKCQ	5	STIL	4	FAM120AOS	3	ITPR2	2	CLASP2	2	ZNF655	1	FAM188A	1
SAMHD1	17	VIM	10	KIAA1715	7	MXI1	5	TNFSF15	4	MTMR9	3	ENOX1	2	AGAP1	2	FAM167B	1	HP1BP3	1
MAP3K8	17	PRICKLE1	10	FAR2	7	CLEC3B	5	SYNJ2	4	GORASP2	3	VGLL4	2	GNPNAT1	2	FRMD3	1	ASCC1	1
BUB1	17	PARVA	10	KDM5B	7	PPP1R3B	5	KDELC2	4	NUP210	3	CEP97	2	B4GALNT1	2	PPM1D	1	BCL2L13	1
SKP2	17	P4HA1	10	HBP1	7	CPA3	5	ATP8B1	4	ARHGAP36	3	TEC	2	GPR84	2	ZNF702P	1	GREB1	1
PAM	16	SELP	10	IPO5	7	SLC18A2	5	IKZF2	4	CEBPZ	3	DIAPH3	2	RFESD	2	SLC39A9	1	GAD1	1
KITLG	16	PLA2G4A	10	MANEA	7	BTLA	5	FZD4	4	DNAJC10	3	CHD8	2	ADAP2	2	DGKE	1	PLK2	1
EIF2AK2	16	CYLD	10	PGRMC2	7	TPSAB1	5	PPP2CB	4	FAR1	3	BTBD7	2	PPP1R15B	2	WDR76	1	PHACTR2	1
STAT1	16	EPHA3	10	BMPR2	7	TPSB2	5	ADAMTS3	4	ASNSD1	3	SYAP1	2	PLEKHA4	2	TXNDC16	1	ERGIC2	1
IFIH1	16	AOC3	10	ATP11C	7	HBD	5	HIVEP3	4	PPIL1	3	NIPAL1	2	TNFSF8	2	OSBPL11	1	LIMS3	1
GPR65	16	GREM1	10	RNF38	7	HBB	5	PRSS35	4	MAP3K7	3	KLRG1	2	ICOSLG	2	JPX	1	TMEM74	1
MEF2C	16	LGALS8	10	SHISA2	7	CNKSR3	5	PRR5L	4	GTF2H1	3	KAT7	2	ETV3	2	LRCH3	1	IGSF1	1
IQGAP2	16	DOCK11	10	ALDH1A3	7	SAMD9	5	ZDHHC21	4	PQLC3	3	PDE2A	2	ZNF800	2	SMG8	1	MAP2	1
LPXN	16	PLSCR4	10	LARP6	7	APOBEC3B	5	GNA14	4	SLC35E1	3	CD244	2	PIGM	2	NCAPD3	1	SPATS2L	1
ATP13A3	16	LMO4	10	PHF20L1	7	SIGLEC1	5	SLC15A2	4	PI4KA	3	IRF5	2	PAPD4	2	ZIK1	1	SARDH	1
TGM2	16	RAB1A	10	RELN	7	ATXN3	5	JPH1	4	GTF3C2	3	CCR8	2	ESAM	2	PAIP2	1	SVIP	1
PDE4DIP	16	MMD	10	REEP1	7	ZFX	5	SMG1	4	TM7SF3	3	SORCS2	2	ITIH3	2	TCEANC	1	AR	1
MAOA	16	LRRC17	10	FMOD	7	NUP98	5	APH1A	4	ASCC3	3	RNF217	2	CCNT1	2	ZNF770	1	NPTX1	1
ACP5	16	YPEL5	10	MAST4	7	SERPINB8	5	COPA	4	NUDT11	3	TUBB2B	2	GTF2IRD2	2	RBM45	1	ARSI	1
CCR7	16	MMP16	10	PCDH17	7	SLC8A1	5	USP10	4	DNAJC13	3	CST4	2	ENOX2	2	GTPBP10	1	KLHL10	1
CLEC2B	16	STRN3	10	PCGF5	7	PIK3CG	5	GRINA	4	LRRC57	3	TEX14	2	MBTD1	2	ZNF229	1	ALMS1	1
TACC1	16	RYBP	10	SCYL2	7	ZBTB1	5	TMEM167A	4	CCNYL1	3	ARHGEF15	2	LOC374443	2	EIF2AK3	1	ATP6V1C2	1
TYROBP	16	DEPDC1	10	RPE	7	PARP8	5	KLHDC2	4	FAM20C	3	PRKG2	2	TMEM156	2	SNX30	1	SYT9	1
FCER1G	16	EID1	10	MCM2	7	MAN1A2	5	WNT4	4	TMEM135	3	FCAR	2	DPY19L4	2	SERAC1	1	CHIC1	1
IFI30	16	LITAF	10	PRIM1	7	ASXL2	5	CDC45	4	UBE2N	3	ONECUT2	2	PRKAG2	2	KLHL15	1	SORCS3	1
STAT4	16	NEDD9	10	PCDH18	7	ZFAND6	5	RAD51	4	ZHX1	3	FBLN7	2	RAB12	2	FAM102B	1	PDZRN4	1
EVI2B	16	ODC1	10	RAMP3	7	FAM76B	5	NCAPG2	4	KIAA0196	3	PIK3R5	2	GLI2	2	LATS1	1	PHEX	1
AIF1	16	HSP90AA1	10	CD4	7	EVI5	5	FAM46A	4	MAL2	3	FOXP3	2	ZNF175	2	WNK3	1	PLBD1	1
CD52	16	SFPQ	10	CKAP2	7	WAC	5	NDFIP2	4	INPP4A	3	LILRB5	2	NBEAL1	2	IPP	1	RRAD	1
SERPING1	16	ANTXR2	10	UCP2	7	LYSMD2	5	ITGAX	4	MAPK8IP1	3	SAMD3	2	LRRC1	2	RAB3GAP2	1	ADAMTSL3	1
FZD7	16	ACTR2	10	CXCR6	7	ITCH	5	NPR1	4	SPOCK2	3	SDS	2	DNAJC22	2	MAP2K5	1	TPM4	1
DPYSL3	16	TRIO	10	STC1	7	MYO1F	5	CHL1	4	BMP5	3	FFAR3	2	RGAG4	2	RC3H2	1	GBP2	1
MAP1B	16	SMAD5	10	PLCB1	7	EGR2	5	TMCC3	4	NTRK1	3	CCL18	2	LPCAT2	2	CTSF	1	FAM13A	1
AHR	16	TPP1	10	SOCS2	7	SLC16A6	5	CSNK1G1	4	CACNA1G	3	RAB42	2	SLCO2A1	2	LYAR	1	ASNS	1
IGFBP3	16	MATR3	10	NRP2	7	CDK5R1	5	RDH10	4	HIST1H4H	3	PPP2R2B	2	TAOK3	2	WDR72	1	HSPB7	1
GNAI1	16	KLF3	10	OLFML2B	7	GIMAP7	5	OLFML2A	4	DACT1	3	APOL4	2	ZNF654	2	FAM126B	1	SLC7A1	1
WWTR1	16	HELLS	10	CYFIP1	7	SGTB	5	GIMAP8	4	NLGN4X	3	DECR1	2	ETFA	2	ASB9	1	ACLY	1
GULP1	16	TNPO1	10	OSR2	7	PLCL2	5	RASA4	4	HMCN1	3	HRH4	2	VTA1	2	PRTFDC1	1	MYO10	1
PPP1R3C	16	SRSF10	10	SCRN1	7	CD300A	5	MEOX1	4	GIMAP2	3	FOLR3	2	TGS1	2	ZNF385A	1	TRIB3	1
TNFSF10	16	DNAJA1	10	TNS1	7	ACAT1	5	PALMD	4	SLC26A2	3	IL3RA	2	SNRNP48	2	GAMT	1	TRIB1	1
PIK3R1	16	HIPK1	10	CNRIP1	7	ZNF804A	5	FOLR2	4	PLA1A	3	ARHGAP18	2	SYNJ2BP	2	LRRCC1	1	EGR1	1
UGCG	16	BIRC2	10	MSRB3	7	NCOA4	5	APLNR	4	TMEM70	3	IDH3A	2	GXYLT1	2	ZCCHC5	1	YARS	1
ST3GAL6	16	RAPGEF5	10	ROR1	7	MFHAS1	5	ACVRL1	4	OLAH	3	DCAF7	2	BRWD3	2	ADHFE1	1	RAP1GDS1	1
PDLIM5	16	ADRB2	10	SSPN	7	TREM1	5	CD247	4	IRAK2	3	MRE11A	2	HCFC2	2	DIRAS2	1	FMO2	1
HTRA1	16	CHD9	10	GAS7	7	CEACAM6	5	CD96	4	GPR3	3	SKIV2L2	2	TIPRL	2	SERHL2	1	TNMD	1
ELK3	16	SMC5	10	OMD	7	RDH11	5	TNFSF13B	4	ZNF644	3	CCNI	2	USP11	2	IKZF4	1	ANG	1
ARHGAP5	16	TSC22D2	10	SLIT3	7	CDS2	5	FIBIN	4	NFYA	3	STX3	2	CDC73	2	HIPK2	1	KLF6	1
HIF1A	15	SOCS5	9	PMP22	7	CREBL2	5	MT1X	4	RNF6	3	ATP6V1B2	2	DNAL1	2	HECTD2	1	UCHL1	1
PHTF2	15	RBFOX2	9	LPAR1	7	YWHAQ	5	ARPC2	4	RPAP3	3	MFSD1	2	TRIP11	2	QPCT	1	SLC2A3	1
PELI1	15	HS3ST3B1	9	SYNE1	7	HEXA	5	PEX13	4	DUSP11	3	SFT2D2	2	AP2M1	2	IFT80	1	ABCC4	1
ITGAM	15	ARPP19	9	AOX1	7	ITGA5	5	AMPH	4	IPMK	3	DOK3	2	PTBP2	2	LRRC8D	1	TRIM25	1
PNP	15	ABCB1	9	PRR16	7	SYPL1	5	ALDOC	4	SLC29A1	3	FMNL1	2	ZNF557	2	ETNK2	1	TBP	1
PECAM1	15	HOXB2	9	TSPAN5	7	GCNT1	5	ISLR	4	KIF1B	3	RNF24	2	ZNF432	2	NAB2	1	GPR68	1
GNS	15	GMFG	9	PLD1	7	S100B	5	ALDH1L2	4	EXOC6	3	DCHS1	2	RIC8B	2	NSL1	1	CCDC47	1
DFNA5	15	CPNE3	9	ATP10A	7	ICAM3	5	FNDC1	4	ABCB4	3	GNG7	2	CCNT2	2	ABCG4	1	CCNY	1
IRF4	15	BCL2L11	9	PDE1A	7	FCER1A	5	FAM19A5	4	WNT2B	3	PANX1	2	ZNF227	2	IER3IP1	1	KIR2DL3	1
SSBP2	15	FLT1	9	HDGFRP3	7	DEPTOR	5	PPP1R12B	4	FLT4	3	DACH2	2	KIF18A	2	NEK9	1	NPFFR2	1
GNA13	15	CDH5	9	YAF2	7	ARHGEF12	5	HSPG2	4	TAL2	3	LCOR	2	KLHL23	2	UBQLN1	1	APBB1	1
LST1	15	DNAJB6	9	SNTB2	7	TP53INP2	5	MYOCD	4	NFAM1	3	GDNF	2	TBC1D12	2	SNX12	1	MICA	1
C5AR1	15	CCDC88A	9	TPM2	7	SIDT2	5	FHOD3	4	VAV1	3	PUS10	2	ZNF45	2	IPO9	1	ZNF43	1
LY96	15	PAG1	9	FZD8	7	ARRDC3	5	PLN	4	PLEKHO1	3	ITM2C	2	STBD1	2	AAGAB	1	SDHC	1
MS4A6A	15	COBLL1	9	PVRL3	7	RPS6KA5	5	ELOVL2	4	DPP4	3	RASGRP2	2	NR2C2	2	MPP2	1	TMED10P1	1
FCGR2A	15	TMEM64	9	ETV5	7	HIPK3	5	PRELP	4	DSCC1	3	RBBP9	2	DNAJC24	2	ZC3H10	1	KLHDC3	1
CYTIP	15	CD28	9	ADAMTS2	7	FAM175B	5	FST	4	IGFLR1	3	MUT	2	TESK2	2	KIAA1586	1	MPV17	1
CCL5	15	RTN1	9	MYO1E	7	PTPN1	5	EML1	4	SASH3	3	PIK3R4	2	ZNF273	2	RINT1	1	SNX31	1
TFEC	15	RASGRP1	9	CORO1C	7	ANKRD28	5	COL6A2	4	DPYSL2	3	CNTNAP1	2	BRAF	2	BRI3BP	1	KCNB2	1
FLI1	15	TFDP1	9	CAP1	7	TAB2	5	MMP2	4	SYNPO2L	3	ATG4C	2	ZBTB6	2	SRFBP1	1	SLC10A5	1
LOX	15	CCL3	9	SCARA3	7	TOR1AIP1	5	TSC22D3	4	SPSB1	3	TMEM86A	2	INTS7	2	INTS2	1	KIAA1429	1
NID2	15	CLEC4E	9	MFGE8	7	IQSEC1	5	PGF	4	LIMD2	3	PLXDC2	2	MCM9	2	GFM1	1	NIPAL2	1
GPM6B	15	SLC30A1	9	ABCD3	7	GNAI3	5	CRABP1	4	KCTD10	3	CD300LF	2	NBAS	2	TLR10	1	HNF4G	1
ANGPTL2	15	CPD	9	UGDH	7	ARID4A	5	FAM110B	4	IMPA2	3	ZNF93	2	LRRC15	2	RPF1	1	GPR63	1
MYLK	15	CPM	9	ETV1	7	NEDD4	5	CRIP2	4	GBP3	3	WSB2	2	STK32B	2	THAP1	1	SPN	1
IRS1	15	DMXL2	9	PRDM1	7	ADAMTS12	5	CYP26B1	4	CTNNB1	3	BCL10	2	ZNF844	2	AP3M2	1	ZNF665	1
RGL1	15	LAIR1	9	KPNA4	7	NDRG4	5	PODXL	4	CACNA1H	3	TNFSF18	2	DTWD2	2	SLC36A1	1	CENPL	1
RND3	15	MT1G	9	RAI2	7	ZBTB8A	5	DHRS7	4	CHST1	3	SAMD8	2	USP45	2	SHROOM4	1	MYO1G	1
HAS2	15	VSIG4	9	ELOVL5	7	FAM49A	5	FANCI	4	POU6F1	3	ZBTB34	2	FAM189A2	2	NIPA2	1	KLHDC8B	1
LAMC1	15	ADAM28	9	NRAS	7	RBBP5	5	SERPINH1	4	EOMES	3	SLC30A8	2	TYRO3	2	AAK1	1	WDPCP	1
AXL	15	TM6SF1	9	PAIP1	7	HACE1	5	FIGN	4	CD40	3	IKZF3	2	DUSP14	2	ZNF614	1	RAB11FIP3	1
ADD3	15	SLC43A3	9	LARP4	7	ARMCX3	5	SCUBE2	4	ODAM	3	GYS2	2	LANCL1	2	ZNF611	1	GPRASP2	1
SLC4A7	15	CD59	9	PTP4A1	7	GTF2A1	5	ARHGAP24	4	RSU1	3	CCR4	2	VPS26A	2	SLC16A10	1	SEC11A	1
TIPARP	15	EED	9	PRCP	7	CAPRIN1	5	GSTA1	4	VAMP1	3	SNAPC1	2	MYO1B	2	ZNF549	1	CDON	1
NDRG1	15	SUZ12	9	FCGRT	7	MPDZ	5	COL4A6	4	GGT5	3	IL20	2	GJA5	2	CBLN2	1	CASD1	1
MCM4	15	CLIC2	9	PITPNB	7	MPPED2	5	ASPA	4	CD6	3	RAB6C	2	N4BP2L1	2	ZNF483	1	INCENP	1
ADAM10	15	EMP3	9	PANK3	7	WASL	5	TMEM100	4	SP140	3	RAB39B	2	KCND3	2	CRCP	1	TBC1D2B	1
IQGAP1	15	SWAP70	9	PTPRG	7	TMTC1	5	CXCL13	4	PDE7A	3	ADAM17	2	RHEB	2	EIF1AD	1	ATF7IP	1
PAPOLA	15	NCF4	9	PPIG	7	TRIM5	5	CDKL1	4	APBB1IP	3	ANKRD13A	2	ARHGAP19	2	TBC1D8B	1	CEPT1	1
EPS8	15	HGF	9	MTDH	7	LRP12	5	RAB3B	4	ARHGAP30	3	LRIG2	2	CSPP1	2	EXT2	1	CUBN	1
SMC4	15	ELMO1	9	UBXN4	7	FGF7	5	AFF3	4	FCGR3A	3	CHMP5	2	STRADB	2	NBPF14	1	ZNF143	1
SQLE	14	ANKRD12	9	RAB2A	7	TRPV2	5	SCARA5	4	ARRDC4	3	ARMCX6	2	ATG4A	2	DENR	1	RASSF3	1
DRAM1	14	SEC23A	9	ITSN1	7	SLC27A2	5	TSHZ2	4	MAB21L2	3	DLG4	2	TCP11L2	2	NBPF3	1	UBN1	1
FKBP5	14	HIST1H2BD	9	FCHSD2	7	KLHL7	5	AKAP2	4	APCDD1L	3	SERPINB9	2	CCP110	2	HNRNPUL1	1	CPSF7	1
HOPX	14	ING3	9	CHST11	7	NUCB2	5	MYC	4	TANC2	3	MED17	2	ARSB	2	DDX52	1	OSBP	1
GPR18	14	CPEB3	9	TNFSF4	7	RASSF8	5	PPP1R18	4	KCTD4	3	WDR44	2	LDHA	2	DUSP19	1	GCC2	1
USP18	14	ZBTB20	9	SFRP2	7	SNAI1	5	NTF3	4	TSHZ1	3	P2RX1	2	SNRNP27	2	USP49	1	SNIP1	1
CDC27	14	CD36	9	CPEB2	7	PBRM1	5	RCSD1	4	CYTH4	3	MAGI3	2	PHF10	2	SAR1B	1	RBM27	1
BCL6	14	MCM10	9	CTNND2	7	TWISTNB	5	PDGFA	4	MFNG	3	LGALSL	2	MPP4	2	TRIM62	1	BIRC6	1
CD38	14	ACSL1	9	EFHD1	7	AP4S1	5	TCF7L1	4	MT1E	3	KIAA1211	2	PDE1C	2	SGSM1	1	VHL	1
MSR1	14	ENAH	9	STXBP3	7	OTUD6B	5	OSBPL6	4	MT2A	3	PARD3B	2	SSR3	2	NIPSNAP3B	1	FOLH1	1
ITPR1	14	PHLDB2	9	SOS1	7	KBTBD6	5	SLC27A6	4	RNF41	3	ITFG1	2	CUX2	2	COL28A1	1	SPECC1L	1
NPL	14	PPM1A	9	MAP3K1	7	SGK3	5	UBE2QL1	4	LINC00472	3	CPPED1	2	OCLN	2	TMEM87B	1	ZFYVE9	1
RIN2	14	LMCD1	9	ARID4B	7	PI4K2B	5	MAP3K14	4	LAMB2	3	PRKAB2	2	PIP5K1B	2	FILIP1	1	CCDC93	1
TPST1	14	IL1RAP	9	SNRK	7	CREB1	5	COQ10B	4	NEURL1B	3	NPIP	2	PDE6B	2	GLTP	1	ZMYM4	1
SH3BP5	14	MYCT1	9	SMC2	7	PHLPP2	5	PARP4	4	NRN1	3	SEL1L	2	UBE2Q2	2	SLC20A1	1	PPM1M	1
LY86	14	ATP11B	9	MLLT10	7	TRIM33	5	RBM7	4	PLAUR	3	KIF3C	2	UBAP1	2	PROM2	1	PISD	1
KYNU	14	PLEKHB2	9	COL10A1	7	POGLUT1	5	KIRREL	4	ABI2	3	CENPI	2	EAF1	2	PLEKHA3	1	HCAR2	1
APOC1	14	PSIP1	9	SOX17	7	NACC2	5	SCPEP1	4	PHACTR1	3	BAG5	2	CCNJ	2	PLEKHA7	1	RDH5	1
SLC1A3	14	FIGNL1	9	TMEM123	7	GALNT3	5	PPP4R1	4	ZNF536	3	SYNJ1	2	MAPKAP1	2	HTR7P1	1	CCL17	1
LAMP3	14	KDR	9	BEX4	7	FAM84B	5	CCDC82	4	SLC19A2	3	RFXAP	2	ASH1L	2	RAB38	1	MITD1	1
STOM	14	STAB1	9	NPTN	7	EFR3A	5	GXYLT2	4	ABCC9	3	DBR1	2	DCBLD1	2	SIK2	1	DCP1A	1
NCF2	14	POU2AF1	9	ACTR3	7	ERBB4	5	TMEM165	4	BCAT1	3	PANK1	2	USP53	2	CHRNA3	1	PIK3R6	1
CD86	14	CORO1A	9	AASDHPPT	7	ZNF91	5	TMEM59	4	CDH6	3	SNAPIN	2	FAM133B	2	GPR85	1	GNG4	1
PCDH7	14	HCLS1	9	TMED5	7	USP8	5	EFNA5	4	JMY	3	EMID1	2	RNF14	2	MCF2L2	1	SLC2A6	1
RUNX1T1	14	SH3BGRL	9	USP46	7	DDHD2	5	PKP2	4	SYNGR1	3	PABPC4L	2	DAZAP2	2	FKBP7	1	DRAM2	1
IGFBP5	14	CLEC7A	9	LIMS1	7	WT1	5	TSPAN18	4	EFEMP2	3	MYO9A	2	LRRC58	2	KRCC1	1	DENND2C	1
BCL2A1	14	TNFRSF17	9	CANX	7	SLFN12	5	SYT1	4	SAMD12	3	ZNF326	2	ASAH2B	2	MKX	1	ATP1B2	1
FCN1	14	RASGRP3	9	HIST1H4C	7	HIST1H2BE	5	HS2ST1	4	SLC6A6	3	AP3M1	2	SNX19	2	CD109	1	MIER1	1
PLEK	14	ELANE	9	ADAMTS6	7	FAM107B	5	HEY1	4	KCNJ8	3	LMBR1	2	DCTN5	2	TMEM108	1	RNF157	1
RNASE6	14	AQP1	9	GPR176	7	PREX1	5	TLR3	4	PODN	3	GPAM	2	DGKI	2	GABBR2	1	RNF168	1
MRC1	14	SPARCL1	9	CUL4B	7	IGDCC4	5	MYLIP	4	ECHDC1	3	UGGT1	2	MANEAL	2	ADD2	1	AFTPH	1
DOCK10	14	PIK3CD	9	TANK	7	GFRA2	5	NELL2	4	CLMP	3	BRMS1L	2	CLSPN	2	SLC22A17	1	STAB2	1
THBD	14	COTL1	9	LYPLA1	7	CD274	5	ACACB	4	SGCD	3	RSF1	2	MCM8	2	SPTLC3	1	YPEL4	1
FAP	14	CD8A	9	TAOK1	7	OPN3	5	SLC1A1	4	KCND2	3	PLEKHG1	2	MCMBP	2	DIP2B	1	PPP6R3	1
CLU	14	CSRP1	9	PCNA	7	UEVLD	5	DOK5	4	OLFML1	3	FAM120A	2	ADAM22	2	DUSP6	1	MAGEL2	1
C1R	14	TES	9	RFC3	7	TFAM	5	IL13	4	CILP	3	SLC25A33	2	ADH5	2	KIAA1456	1	XPO4	1
COL18A1	14	BMP2K	9	RAF1	7	ROBO4	5	CAND1	4	LRP1B	3	USO1	2	COL27A1	2	FREM1	1	XPR1	1
VCL	14	PDGFRB	9	KRIT1	7	SLFN12L	5	MAPK1	4	ENO2	3	AK3	2	ZNF92	2	SLC17A7	1	GAS2L1	1
MYL9	14	ITGBL1	9	CDC6	7	FCGR1A	5	HNRNPK	4	STC2	3	ZNF567	2	SCAI	2	ZCCHC12	1	PIGN	1
CFH	14	COL15A1	9	CDR2	7	BCAP29	5	HSF2	4	AGTR1	3	SUCNR1	2	NAV3	2	HIST1H2BC	1	ITIH1	1
SLIT2	14	CTHRC1	9	UAP1	7	KPNA1	5	PPP3R1	4	DHCR24	3	WNT9B	2	SECISBP2L	2	OPCML	1	HNF4A	1
ARID5B	14	ANTXR1	9	PDCD10	7	CCL11	5	ATF6	4	UBR7	3	EFNB3	2	ZNF146	2	SLCO5A1	1	NUP54	1
KIT	14	GYPC	9	RCN2	7	NOTCH4	5	PRDX3	4	SPIN4	3	FAM26E	2	GTF2E1	2	RIMKLB	1	RAD18	1
DMD	14	SH3GLB1	9	AZIN1	7	IL27RA	5	GPR1	4	TMEM163	3	PDP1	2	SLC25A44	2	SLAIN1	1	SERPINC1	1
MALT1	14	ROBO1	9	CAPZA2	7	SLC2A5	5	CALM2	4	DCLK2	3	FAM89A	2	RNF4	2	COL21A1	1	FECH	1
PROS1	14	CRISPLD2	9	ACVR1	7	FCGR1B	5	RIPK2	4	PBXIP1	3	MTMR3	2	SNRNP40	2	TBXA2R	1	SIN3A	1
MXRA7	14	ADAMTS5	9	OSTM1	7	SLC46A3	5	UBE2W	4	EMB	3	FGD6	2	SPG11	2	NRK	1	MYSM1	1
PLAT	14	CSRP2	9	PSAT1	7	MMP14	5	SLC25A32	4	SCN1B	3	MAP6	2	MFAP3	2	DNAJC12	1	GPR146	1
LOXL2	14	RARRES2	9	UBE2D3	7	TNFRSF19	5	RASSF5	4	TMEM117	3	CYP7B1	2	CGRRF1	2	FRK	1	DHX40	1
SEMA3C	14	FAM198B	9	YME1L1	7	NCKAP1L	5	SPTLC1	4	TMEFF2	3	ERLIN1	2	MTF1	2	TSPAN11	1	CA11	1
ITGA4	14	FGFR2	9	RP2	7	GVINP1	5	PRKAR2A	4	MAP9	3	DNAJB5	2	COG5	2	ALG6	1	ASAP3	1
DPYD	14	RASSF2	9	KDM5A	7	RGS18	5	NCOA7	4	JAK3	3	NGLY1	2	VRK2	2	CSRNP3	1	GRIK3	1
FZD1	14	ABI3BP	9	AFF1	7	LPAR4	5	XRN1	4	NOVA1	3	SLC16A4	2	GTF2A2	2	GRP	1	PRDM2	1
NFIL3	14	TSPAN7	9	BAZ2B	7	SELM	5	ZNF641	4	SMOC2	3	MPP5	2	PHKB	2	CSPG5	1	YLPM1	1
CD83	14	JAM2	9	CDYL	7	EPCAM	5	RAB11FIP2	4	PDE1B	3	TMEM106C	2	SH3RF1	2	ALDH5A1	1	CLCN6	1
KIAA0101	14	TOX	9	PHIP	7	PRG2	5	GABPA	4	CD200	3	PSMD5	2	DCAF6	2	CYYR1	1	ZNF12	1
ASPH	14	SGCB	9	LSM14A	7	HPGD	5	KPNA5	4	TBPL1	3	AFAP1L1	2	RPRD1A	2	TDO2	1	STK38L	1
CXADR	14	BDNF	9	PHF3	7	GLT8D2	5	PTPN21	4	AHNAK	3	FBXW2	2	NUP133	2	ABCA5	1	UBA5	1
TOP1	14	ITGB8	9	CNOT2	7	KCNJ15	5	ZFPM2	4	PGR	3	SCD5	2	NHLRC2	2	PAQR5	1	UBP1	1
BCLAF1	14	NCK1	9	CUL1	7	FOXF1	5	FAM13B	4	ITGA7	3	CHRAC1	2	PEX19	2	STMN4	1	DIAPH2	1
PRKD3	14	MDFIC	9	SMAD1	7	STARD4	5	POLA1	4	LMOD1	3	LGR4	2	SUCLG2	2	CDH4	1	ZSCAN2	1
KIF2A	14	DNAJB4	9	PEA15	6	ELN	5	KCTD20	4	ENTPD3	3	ABL2	2	ATG3	2	ALK	1	CCNDBP1	1
PTK2	14	TBC1D1	9	UBTD2	6	TGFB3	5	DDX21	4	MAMDC2	3	NAALADL1	2	HERC2	2	CADPS2	1	ZNF561	1
TLK1	14	SOCS3	9	UBE3A	6	NOX4	5	NOLC1	4	ID4	3	SLC9A9	2	MORF4L1	2	MMRN2	1	ABHD4	1
PPP1R12A	14	MSN	9	OPA1	6	ADAM12	5	WDR26	4	NTRK3	3	SRSF8	2	RHOT1	2	CLEC1A	1	ACHE	1
RHOH	13	CAV2	9	SLC35B3	6	MXRA5	5	PRKAA1	4	STOX2	3	HSPA14	2	SCAPER	2	PTPN5	1	PCDH1	1
NFKBIA	13	VAMP3	9	PPAT	6	AMIGO2	5	NAA50	4	C6	3	ARL5B	2	ZC3H15	2	SYTL3	1	TBC1D23	1
EPAS1	13	METTL7A	9	SMARCA5	6	COL12A1	5	CTTNBP2NL	4	MT1M	3	IPO11	2	ZC3H13	2	SLC30A6	1	KDM3A	1
CD1D	13	PLEKHF2	9	STXBP6	6	PRICKLE2	5	NEMF	4	GREM2	3	CDK12	2	GMPR2	2	ATAD5	1	SESN2	1
MEIS1	13	PEG3	9	KIAA0125	6	GJC1	5	SRSF7	4	HAPLN1	3	PHAX	2	KATNA1	2	COX11	1	ZBTB37	1
ALDH1A1	13	A2M	9	GBP4	6	ITGA9	5	RPGR	4	SIT1	3	API5	2	RXRB	2	FAM122A	1	SLC9A1	1
ANGPT1	13	RGS5	9	GCA	6	PELO	5	DHX15	4	RERG	3	DDX20	2	MRAS	2	GATC	1	ANO6	1
CX3CR1	13	PLXDC1	9	SH3BGRL2	6	RASL12	5	NMT2	4	DSEL	3	GNL3L	2	PER2	2	LZIC	1	KIF3A	1
IL2RB	13	WWP1	9	CACNB2	6	DKK2	5	NPEPPS	4	UNC5C	3	CHUK	2	GNPTAB	2	INTS4	1	RWDD2A	1
LMO2	13	WSB1	9	ACTC1	6	HDAC4	5	PCMT1	4	CHODL	3	SIGLEC10	2	WWC3	2	RBM44	1	CMYA5	1
CD68	13	RAB6A	9	KALRN	6	PAMR1	5	USP13	4	ADCY2	3	HGSNAT	2	COPB1	2	RFWD3	1	ARHGAP12	1
LAMP2	13	PBX1	9	MX2	6	CYP1B1	5	LAT2	4	SCN9A	3	UNC5D	2	LPCAT3	2	VPS8	1	SGCG	1
CCL8	13	SNCA	9	FOXC1	6	PYGL	5	TIFA	4	RBM24	3	TM2D2	2	WWOX	2	GGN	1	ATG5	1
SLAMF1	13	MTHFD2	9	TMEM140	6	NUPR1	5	TANC1	4	AMPD1	3	FAM73A	2	MTHFD1	2	NHEJ1	1	FBXO22	1
TRIM22	13	FBXO32	9	ZBTB10	6	RASSF4	5	TAL1	4	SLC27A3	3	FAM160B1	2	POLR2B	2	EPT1	1	DHX36	1
PDE4B	13	STON1	9	KBTBD8	6	PLXND1	5	SPTA1	4	RAB34	3	TMEM185B	2	PMCH	2	HARBI1	1	ASB8	1

SOAT1	13
ADAM9	13
GCH1	13
ALOX5AP	13
LDB2	13
SH2D1A	13
GIMAP4	13
ITK	13
SELL	13
RAB27A	13
FILIP1L	13
EDIL3	13
PAPPA	13
SFRP4	13
AEBP1	13
MGP	13
COL11A1	13
PTRF	13
LAMA4	13
MFAP4	13
FBLN1	13
DDR2	13
FAM129A	13
PTX3	13
IL6R	13
ID3	13
CTGF	13
SMAD3	13
PTPRM	13
TGFB1	13
FSTL1	13
LXN	13
TMEM45A	13
NAMPT	13
TMX4	13
ZEB1	13
SAMSN1	13
FAM46C	13
ST8SIA4	13
USP1	13
BACH1	13
B3GALNT1	13
LEPROT	13
FNBP1L	13
USP16	13
NAB1	13
ATRX	13
CDH2	13
GATA3	12
GPR171	12
PRKCH	12
UBASH3B	12
PIP4K2A	12
RGS1	12
CD69	12
ENTPD1	12
IL10	12
CCL7	12
SCARB2	12
MITF	12
CD9	12
WBP5	12
CERS6	12
JAK1	12
RFTN1	12
ATXN1	12
CFP	12
PTPN22	12
B3GNT2	12
ZFP36L1	12
LIFR	12
BNIP3L	12
KLHL24	12
CNN3	12
ZFAND5	12
PICALM	12
CASP3	12
ICAM1	12
BMP2	12
ANGPT2	12
SP100	12
FPR1	12
MNDA	12
PIM2	12
COLEC12	12
GPRC5B	12
LTB	12
APP	12
GZMK	12
TARP	12
ITGAL	12
ARHGAP15	12
RPS6KA2	12
CA12	12
CYBRD1	12
CTSK	12
ASPN	12
THY1	12
LGALS1	12
SERPINF1	12
LTBP1	12
MFAP5	12
COL14A1	12
PDGFD	12
COL8A1	12
KCNMA1	12
SCHIP1	12
CBLB	12
RAI14	12
ENG	12
WLS	12
ATP10D	12
PRKCA	12
TRAM2	12
CALU	12
DCLK1	12
CFI	12
AP1S2	12
CFL2	12
SON	12
PRKACB	12
WNT5A	12
NRG1	12
ABCA1	12
PLAGL1	12
PIM1	12
TIAM1	12
S1PR3	12
CDK17	12
NEK7	12
RRAS2	12
CCNB2	12
EPB41L3	12
RDX	12
MTSS1	12
DHX9	12
TGFBR1	12
YWHAZ	12
RBBP4	12
PRPF4B	12
SNAP23	12
IDS	12
CPEB4	12
RRM1	12
GINS1	12
KIF23	12
SMC1A	12
RABGAP1	12
E2F5	12
GNG2	11

SEC62	9
MPZL2	9
MGAT4A	9
KAT2B	9
RAD21	9
RAD23B	9
RAB5A	9
TPD52	9
TRPC1	9
DNAJB9	9
OLFM1	9
CDC20	9
MELK	9
DLGAP5	9
FUT8	9
ENPEP	9
SERINC5	9
SEC24A	9
ATP2C1	9
SF3B1	9
NXT2	9
FBXW11	9
DLG1	9
USP34	9
POLD3	9
DCK	9
MTF2	9
WDHD1	9
ATAD2	9
NBN	9
ACADM	9
TTK	9
PPP2R1B	9
AMD1	9
ATP2A2	9
TUSC3	9
APC	9
MICB	9
SLC25A36	9
UBL3	9
RBPJ	9
KAT6B	9
FNDC3A	9
ZEB2	9
OXCT1	8
INSIG1	8
SYT11	8
MEGF9	8
TAF2	8
STAG1	8
SMPDL3A	8
BLNK	8
LAPTM4B	8
OLR1	8
IKZF1	8
VWF	8
EMCN	8
TMEM200A	8
TYRP1	8
OAS3	8
LY9	8
MIR22HG	8
COL4A5	8
EPB41	8
TP53INP1	8
LRRC8C	8
CDC42SE2	8
SLC30A7	8
REEP3	8
CNOT6L	8
DENND5A	8
IL1RN	8
CCL4	8
GIMAP5	8
NFKB1	8
MYO5A	8
PSTPIP1	8
AQP9	8
SLC31A1	8
CHST15	8
MAPRE2	8
GNA12	8
KMO	8
TRAF3	8
NCOA3	8
CEP350	8
GFPT1	8
CD1C	8
CD1B	8
MOB1A	8
ANXA4	8
CCDC109B	8
CLEC10A	8
CD7	8
THSD7A	8
ERG	8
DTNA	8
PIP5K1A	8
ATP6V1A	8
CDC42	8
XCL1	8
CEP57	8
HBS1L	8
CALM1	8
HIST1H2AC	8
RIT1	8
ALDH6A1	8
NR1D2	8
SERBP1	8
VPS54	8
ZNF532	8
SAMD9L	8
CTNNAL1	8
BAG2	8
FNBP1	8
PGM2L1	8
SH2B3	8
PRIM2	8
JAM3	8
ESYT2	8
SUPT16H	8
STIP1	8
TIE1	8
S1PR1	8
GNB4	8
LSP1	8
FGR	8
TMEM30A	8
MERTK	8
KIAA1462	8
CTSW	8
IL18R1	8
SPON1	8
HDC	8
MAP4K1	8
SOBP	8
UBE2J1	8
RCAN2	8
BGN	8
SEMA6D	8
BNIP2	8
TUBA1A	8
NEXN	8
SVIL	8
MCAM	8
DST	8
SORBS1	8
ECM2	8
MAN1C1	8
ATP8B4	8

TMX1	6
RBL2	6
NSMAF	6
SNX9	6
IL2RA	6
THEMIS	6
TBC1D15	6
SIK3	6
IFNAR2	6
TTC39B	6
GAS2L3	6
FPR3	6
IL12B	6
CCL13	6
GGTA1P	6
FCGR2C	6
ATP6V0D2	6
SLC15A3	6
EREG	6
CMKLR1	6
COL4A3BP	6
SPTLC2	6
CRTAM	6
LILRB4	6
NLRP3	6
WRN	6
HMGXB4	6
EIF2S1	6
ATP6V1C1	6
UFSP2	6
NPC1	6
ARL6IP5	6
TNFRSF4	6
ATP9B	6
B4GALT5	6
CSF2RA	6
PLD3	6
PGK1	6
LTBP3	6
YAP1	6
TMOD1	6
HPGDS	6
CMTM3	6
CSF3R	6
ABCA6	6
IL16	6
RECQL	6
KLRB1	6
KIAA0430	6
HAT1	6
GIT2	6
DNASE1L3	6
CLOCK	6
SNN	6
GDF3	6
USP25	6
SPIN1	6
TOPBP1	6
PFN2	6
UCHL5	6
TTC33	6
GAPVD1	6
RARB	6
FBXL7	6
DIXDC1	6
PDE5A	6
GLCE	6
FPGT	6
HMGCR	6
SLC35A3	6
ARMCX1	6
GRB14	6
MBD4	6
SEC22B	6
ZFHX3	6
TRIM38	6
HECTD1	6
PPTC7	6
DEGS1	6
CSNK1G3	6
UBXN2A	6
SLC31A2	6
HPSE	6
SPOPL	6
TEK	6
LILRB1	6
FCGR3B	6
CD79A	6
CECR1	6
LIPA	6
GRB10	6
CSF1	6
APOBEC3G	6
CD3G	6
SLC25A24	6
GPR160	6
IL7	6
BTK	6
PRF1	6
ICOS	6
PDZRN3	6
ZNF521	6
PDPN	6
RALB	6
EBI3	6
BCOR	6
FKBP14	6
SGCE	6
JAZF1	6
PDLIM4	6
MRVI1	6
ACTA2	6
KANK2	6
SETBP1	6
BICC1	6
OLFML3	6
STEAP1	6
NAV2	6
PTGFR	6
SLC22A4	6
CDKN3	6
CDKN2B	6
RPS6KA6	6
HSPB8	6
EBF3	6
HLF	6
NTN4	6
PNOC	6
NCAM1	6
CYTL1	6
FAM107A	6
CCL21	6
NAP1L2	6
CDH13	6
CRISPLD1	6
CRLF1	6
FLNC	6
FBLN2	6
FRMD6	6
PNPLA8	6
FAM101B	6
SNX7	6
MTMR2	6
TOX2	6
BNC2	6
WDR1	6
HSPA13	6
TMED2	6
TMEM50B	6
RHOA	6

GAS2	5
FAM20A	5
CD248	5
PRUNE2	5
TMSB15A	5
MLLT11	5
SERPINI1	5
ABCA8	5
CHRDL1	5
MEOX2	5
OSR1	5
SPRED1	5
SLC25A4	5
PPP1R14A	5
ACVR2A	5
FZD2	5
ARNTL2	5
FAM83D	5
GPX8	5
SLC35B4	5
PGRMC1	5
CCPG1	5
APLP2	5
SNX2	5
B4GALT1	5
VGLL3	5
PPFIBP1	5
KBTBD2	5
TSPAN12	5
EIF1AX	5
FAM114A1	5
SLC41A2	5
HHIP	5
IL33	5
SV2B	5
MED14	5
RAB7A	5
KLC1	5
SMNDC1	5
ATXN10	5
TRAM1	5
SNX16	5
IRF2BPL	5
PDIA6	5
HOXD8	5
WRB	5
CERK	5
SPAG9	5
FBXO21	5
DDX5	5
FEM1B	5
SNX1	5
THAP11	5
DDX18	5
PRPF40A	5
CUL3	5
ARIH1	5
CRK	5
FNTA	5
PPP2R3A	5
ST3GAL5	5
SLC25A46	5
CRADD	5
UVRAG	5
WASF3	5
CLIP1	5
GSK3B	5
ARFGEF1	5
WDR43	5
PXK	5
DNAJA2	5
MOB3B	5
MINPP1	5
ZNF281	5
SBNO1	5
N4BP2	5
ZFR	5
CCR9	5
HAUS6	5
KLHL6	5
MORF4L2	5
INPP5F	5
PPP3CA	5
CENPQ	5
GSPT1	5
INTS6	5
ZNF131	5
RAB3GAP1	5
TMEFF1	5
ARNTL	5
CAB39	5
ZNF14	5
ZNF280D	5
CDC40	5
RNF19B	5
IREB2	5
CDCA8	5
MGAT2	5
CSTF2T	5
UNC5B	5
CMAHP	5
STAU2	5
USP24	5
PREPL	5
PLCG2	5
GLUD2	5
MYL12A	5
TAX1BP1	5
FAM63B	5
DHFR	5
ARSJ	5
MEX3C	5
TNKS2	5
PCNP	5
ICK	5
VRK1	5
PRR11	5
ERAP1	5
ETNK1	5
TNFRSF9	5
ZNF267	5
GBAS	5
RIPK1	5
DR1	5
CRKL	5
DICER1	5
PAK2	5
TMED7	5
FBXL5	5
MOB4	5
VBP1	5
NCKAP1	5
HELZ	5
PAK3	5
TRIM37	5
C1D	5
MSH6	5
BDKRB2	5
PCF11	5
SHOC2	5
ZZZ3	5
ZNF451	5
PIK3C3	5
BBX	5
FRYL	5
CDC14A	5
FAM168A	5
UBXN7	5
STMN2	4
CASP12	4

ARMC1	4
CEP76	4
CBL	4
ZNF827	4
ARL5A	4
LZTFL1	4
KDM6A	4
PGM5	4
STAM2	4
SFMBT2	4
GAB1	4
WWC2	4
SERTAD4	4
SYDE2	4
FBXO38	4
LRIF1	4
EPB41L5	4
MBOAT2	4
FRAS1	4
HPS3	4
ZNF467	4
AGTPBP1	4
ZC3H7A	4
NEDD1	4
SLC25A40	4
DCUN1D1	4
NNT	4
PIAS1	4
FNIP1	4
YY1	4
TJP1	4
DNAJC21	4
PDP2	4
KLHL20	4
CHURC1	4
SELT	4
MPP7	4
PPP4R2	4
AKIRIN1	4
NOC3L	4
DUSP3	4
KRR1	4
TM2D1	4
SIKE1	4
TRUB1	4
PAFAH1B1	4
DYNC1LI2	4
GNG10	4
SEMA6A	4
KLHL9	4
AKAP11	4
GYG1	4
UBR2	4
USP9X	4
ELF2	4
CLIC5	4
CREB3L2	4
UGP2	4
SYNPO	4
IARS	4
CKAP2L	4
HOOK3	4
KLHL8	4
NUP160	4
IPW	4
PLCL1	4
DDX6	4
RANBP9	4
RAB5C	4
STYX	4
CTDSPL2	4
ARL6IP1	4
GTF2I	4
STRN	4
GART	4
BRIP1	4
RAD51AP1	4
ARNT2	4
ADD1	4
PRPS1	4
HERPUD1	4
SLC20A2	4
GOLGA7	4
CAPN7	4
ACBD3	4
ACTL6A	4
UBA3	4
SUCLA2	4
APOOL	4
HMG20A	4
TGOLN2	4
ISCA1	4
ZCCHC10	4
FBXO28	4
SLC30A5	4
RAB28	4
NUDT21	4
TDP2	4
ANXA7	4
VPS13C	4
SLC25A12	4
RNF130	4
MED13	4
KIAA0232	4
TRIM23	4
KCNS3	4
WASF1	4
EBAG9	4
SDHD	4
NDUFS1	4
VAMP4	4
MDC1	4
CEP170	4
NCOA2	4
CLASP1	4
PRRC2C	4
PIKFYVE	4
RNF144A	4
PDS5B	4
PLCE1	4
NFATC3	4
ADM	3
ADRA2A	3
GZMA	3
NCAN	3
PVALB	3
MAPK10	3
ENY2	3
CCDC126	3
MMRN1	3
LHX6	3
ZNF639	3
DCUN1D4	3
PGBD1	3
SRPK1	3
C1GALT1	3
EBF1	3
ZCCHC9	3
PUF60	3
RNF139	3
DERL1	3
B2M	3
RFC1	3
KCNMB1	3
RTN2	3
GABARAPL2	3
IFNAR1	3
DENND5B	3
CALN1	3
PCNX	3

AMOTL1	3
LRCH2	3
TSPY26P	3
STARD8	3
SHE	3
PARD6G	3
TSPYL2	3
NGF	3
ARL15	3
HERC3	3
ZC3H12C	3
ZMYM6	3
MCFD2	3
PDE7B	3
F2RL2	3
CRTAP	3
HRH1	3
PLOD1	3
GNAI2	3
TMEM50A	3
ABHD5	3
FADS1	3
TRPC4	3
UTP23	3
MIER3	3
ARL8B	3
MAPK1IP1L	3
KIFAP3	3
RASL11B	3
PIK3IP1	3
UBE2K	3
UXS1	3
PDS5A	3
GAP43	3
THSD4	3
TGFB2	3
CHSY3	3
PLCXD2	3
ISYNA1	3
WFDC1	3
SPRY1	3
MCC	3
NCF1	3
DNAAF2	3
USP33	3
OSGIN2	3
LACTB2	3
RAD54B	3
RASEF	3
PNMAL1	3
UBE2E2	3
SLC30A4	3
EPC2	3
BTF3L4	3
EPM2AIP1	3
UFM1	3
POLE3	3
SGIP1	3
MAPRE1	3
BAZ1B	3
RAB10	3
SIRT1	3
MAP7D3	3
PPP1CC	3
BTAF1	3
HSPA8	3
NUFIP2	3
PPM1B	3
SCAF8	3
ANKRD17	3
GLRB	3
TRIP12	3
AUH	3
ANKS1A	3
PPP2CA	3
CMTM6	3
PRKX	3
PHLDB1	3
NUDCD1	3
SREK1IP1	3
FOXP2	3
VEZT	3
KCNE4	3
ZNF230	3
RPP14	3
LMBRD1	3
FAM65C	3
SLC17A5	3
IRF6	3
DIO2	3
BMF	3
CDKL5	3
KIAA0513	3
QSER1	3
RASA2	3
LONRF3	3
ZDHHC5	3
LNPEP	3
PCMTD1	3
AZI2	3
LIN52	3
YIPF5	3
CHMP2B	3
LRPPRC	3
EIF4G2	3
VCPIP1	3
MB21D1	3
VPS41	3
MAPK8	3
VPS35	3
LTN1	3
DPP8	3
DCTN6	3
PPP3CB	3
TDRD7	3
ZNF468	3
ZNF222	3
RLF	3
NRBF2	3
TMF1	3
DYNC1I1	3
TUBGCP4	3
TAF13	3
DDX10	3
WDR82	3
THAP6	3
TRNT1	3
RAB18	3
PPIP5K2	3
DFFA	3
DCAF13	3
ABI1	3
SKP1	3
MAGI2	3
SRGAP2	3
TSPYL4	3
G2E3	3
STX2	3
P2RY13	3
USP38	3
WNT2	3
SAV1	3
CYTH3	3
VKORC1L1	3
NPAT	3
TTF2	3
CENPJ	3
NUF2	3
CEP135	3
HIVEP1	3

ZFYVE26	2
ZNF493	2
SENP2	2
POC1B	2
TAB3	2
SNAPC3	2
TMX3	2
RABGEF1	2
PHACTR4	2
TMTC3	2
MATN3	2
LRPAP1	2
MTMR12	2
PCYT1A	2
FGF12	2
DAG1	2
SCN3A	2
FAM43A	2
TLL2	2
ATL3	2
BOC	2
JDP2	2
ZSWIM6	2
LRFN5	2
DLG2	2
GABRQ	2
AKAP7	2
TRIM35	2
SCARF1	2
PCDH12	2
PLVAP	2
PITPNC1	2
BDP1	2
SLAIN2	2
ATP6V1E1	2
CD33	2
RNF115	2
CISD2	2
GMCL1	2
ELMOD2	2
POT1	2
PUS7L	2
DIRC2	2
ST7L	2
RQCD1	2
TADA2B	2
AP3S1	2
MTFR1	2
SYNRG	2
NUP205	2
ANGPT4	2
DYRK3	2
CRMP1	2
MKNK1	2
NAGA	2
NINJ1	2
PTAFR	2
PSEN1	2
LAG3	2
FBXW8	2
NTN1	2
FAM49B	2
CD72	2
S100A9	2
HS6ST2	2
TCEAL8	2
AIF1L	2
UBE2E1	2
NR6A1	2
TROVE2	2
VAMP7	2
GOLPH3	2
DCAF5	2
HEY2	2
ITM2B	2
SLC37A4	2
TRIM6	2
SYNDIG1	2
PI15	2
LOC730101	2
NFASC	2
LAX1	2
CORO2B	2
CD3E	2
DCLRE1B	2
KLRC1	2
CACNA1S	2
HOOK1	2
TIRAP	2
PRKCE	2
SELE	2
CCDC102B	2
DOK2	2
KANK3	2
ST3GAL2	2
CYP4F22	2
KCNT2	2
SLC2A9	2
RIOK1	2
VSTM4	2
ITGA11	2
SRGAP1	2
PIEZO2	2
BEND6	2
BDKRB1	2
ZNF738	2
LIG4	2
LATS2	2
FERMT3	2
ADORA2A	2
C4A	2
TCTEX1D1	2
C1QTNF7	2
FAM8A1	2
ZMAT4	2
MEX3B	2
SEMA5B	2
NTM	2
COMP	2
C1QTNF3	2
ISM1	2
GIGYF2	2
GLIS2	2
ITGA8	2
SLMAP	2
EHF	2
EEF2K	2
LAYN	2
GPR161	2
SYDE1	2
RERGL	2
CXCL14	2
PLTP	2
RNASE4	2
SLC5A3	2
CELF2	2
ADH1C	2
MSC	2
CRYAB	2
TNC	2
LRP1	2
MFAP2	2
COL5A1	2
CADM3	2
PTGER2	2
WNT5B	2
RGS17	2
GLRX	2
VAT1L	2
NRXN3	2

PDCD1LG2	2
TOX4	2
TTLL5	2
AGFG1	2
ABCE1	2
RUNX3	1
KCNJ5	1
FNDC9	1
ISLR2	1
SACS	1
CHRNB4	1
PAK6	1
S100Z	1
FAM149A	1
ACTN3	1
MYOZ1	1
KCNA5	1
ZCCHC18	1
SKOR1	1
ELAVL4	1
RND2	1
TMEM26	1
JAKMIP2	1
TRAPPC3	1
RPRD2	1
LRRN4CL	1
CLEC14A	1
RPS6KB1	1
TMEM126A	1
FAM177A1	1
XPOT	1
ZMYM1	1
KIF13A	1
HSPA2	1
BTBD19	1
COG6	1
SLC25A43	1
ZDHHC6	1
CNOT8	1
TBRG1	1
SLC24A2	1
ZNF34	1
LY6H	1
KLHL38	1
CYC1	1
GSDMD	1
ZNF572	1
MED30	1
SPATC1	1
WDYHV1	1
TRAPPC9	1
PYCRL	1
GPR20	1
LYNX1	1
ADARB2	1
PTGES3	1
ADIPOR2	1
TNFSF14	1
SMARCAD1	1
DSTN	1
SOD3	1
PTTG1IP	1
ARAP2	1
PIAS3	1
PRDM5	1
MGAT3	1
ZNF215	1
SBF2	1
NME4	1
FAM60A	1
ZNF585A	1
ADPGK	1
TBC1D19	1
CYP2U1	1
LONP2	1
CRHBP	1
TNFRSF18	1
ARL11	1
STOX1	1
GOLGA5	1
CXXC4	1
ERN1	1
CIDEB	1
NALCN	1
CGGBP1	1
UBASH3A	1
ZNF586	1
TMBIM4	1
P2RY8	1
TBC1D10C	1
ASB7	1
HTR7	1
PRPS1L1	1
HECW2	1
ENTHD1	1
HSFX1	1
RGAG1	1
CRB1	1
ZNF471	1
OPHN1	1
TINAG	1
GPBP1L1	1
B3GALT5	1
SLC12A6	1
COL19A1	1
CTNNA3	1
ARHGAP10	1
APEX1	1
BCL6B	1
INMT	1
TCEAL7	1
JPH4	1
LILRA1	1
GPR82	1
SLITRK4	1
MED18	1
ITGB7	1
FBXL20	1
TDRD9	1
NOG	1
MMP25	1
LILRA3	1
LRRC25	1
ZNF76	1
GRK4	1
SLC4A8	1
CNTNAP3	1
TACR1	1
IQCA1	1
NDST2	1
NIP7	1
IL1B	1
SYT6	1
TNRC6B	1
TRIM2	1
LMAN1	1
HRC	1
CHIC2	1
MPZL3	1
WDR20	1
RSL24D1	1
RNFT1	1
KCNA3	1
EHD1	1
USP31	1
LOXL3	1
TBCK	1
LDOC1L	1
MLC1	1
EPM2A	1

MAGOH	1
XPO5	1
UBTD1	1
OSTF1	1
CLP1	1
TET1	1
WHSC1L1	1
S1PR4	1
LTC4S	1
RXRG	1
TNFSF12	1
DLL4	1
EFNA2	1
TBX1	1
P2RY6	1
PRRC2A	1
PPP2R5D	1
GPSM3	1
ABI3	1
CDC42SE1	1
C1QL1	1
GP5	1
PF4V1	1
SMO	1
SETD7	1
CTXN1	1
NCS1	1
ILK	1
MRPS22	1
VPS4B	1
ATE1	1
CYB5R4	1
TMLHE	1
BTBD1	1
MRRF	1
KIF17	1
ELF1	1
LRRC32	1
ZNF124	1
CCDC144B	1
CPZ	1
PLEKHO2	1
CERS4	1
PIK3CB	1
GPR174	1
PCGF2	1
RRM2B	1
MAPK12	1
GZMM	1
CD79B	1
ACAP1	1
LAMC3	1
LZTS1	1
LILRA4	1
NRIP2	1
CRY2	1
VASH1	1
NCR3	1
FCRL2	1
GLT1D1	1
RALGAPA2	1
VNN2	1
ATP2B2	1
GPR88	1
INHBE	1
PAPLN	1
ADAMTSL1	1
EHBP1	1
ZNF365	1
CREG1	1
PDK1	1
DAAM2	1
HEYL	1
BRPF1	1
RELL1	1
MIAT	1
SOX18	1
ADAMTS18	1
AHRR	1
NFIC	1
RIMKLA	1
RNF152	1
AVPR1A	1
AKAP5	1
ADAMTS8	1
WFIKKN2	1
ERC1	1
CPXM2	1
FAM131A	1
MMP11	1
PRDM6	1
GDAP1	1
ESM1	1
NEFH	1
TXNL1	1
ARL10	1
ADCY4	1
TUB	1
ZCCHC14	1
ICA1L	1
GRHL1	1
S100PBP	1
PREX2	1
CPAMD8	1
SLC47A1	1
COL9A1	1
CCDC136	1
ATP2B4	1
TMEM200B	1
HOXD13	1
ANKRD35	1
CYS1	1
NHS	1
KRT23	1
RAVER2	1
TLE2	1
TMEM35	1
SRSF11	1
KLF8	1
IFFO1	1
GSTM5	1
KCNK1	1
CFB	1
WDR41	1
TRO	1
SHOX2	1
SLC39A14	1
BHMT2	1
HOXC6	1
TNXB	1
MLLT3	1
SRR	1
SV2A	1
RIMS1	1
SLC7A2	1
BPGM	1
TRPA1	1
PRR3	1
ITGA10	1
LRIG1	1
C1QTNF2	1
KCNJ10	1
WDFY3	1
ADAMTS9	1
PLA2G2D	1
ARHGAP42	1
TPO	1
PLCH1	1
SALL2	1
ZNF302	1

YEATS4	1
ZNF562	1
MED19	1
ABCB10	1
ZNF253	1
NBPF1	1
NMD3	1
WIPF2	1
MIS12	1
F8	1
HEATR3	1
POLK	1
CYP20A1	1
RNF111	1
ZNF426	1
GPATCH2	1
FAM53C	1
ZNF136	1
ZNF107	1
ZBTB11	1
ZNF101	1
TTBK2	1
WDR47	1
NECAP1	1
PDCL	1
HIST1H2BN	1
HIST1H2BJ	1
FAF2	1
ZNF254	1
ZNF189	1
NAA25	1
EDEM3	1
DTL	1
MBTPS2	1
PEX26	1
USP44	1
KLB	1
ARHGEF26	1
SYT17	1
PCDHB5	1
ADAMTS7	1
TOR1AIP2	1
LMBRD2	1
ZNF174	1
MGAT5B	1
MEMO1	1
SUSD2	1
PTPN9	1
SURF4	1
GDE1	1
PSMA6	1
NHSL2	1
FBXO34	1
ZNF740	1
STX17	1
LMTK2	1
TAPT1	1
TBCEL	1
ARID3B	1
TFB2M	1
CEP85	1
ATP8B2	1
USP28	1
GABPB1	1
ZNF35	1
APLN	1
ZNF436	1
PEAK1	1
TAF3	1
ZNF41	1
TM4SF18	1
ENHO	1
CEP120	1
CNOT1	1
PDE12	1
POLDIP3	1
CPNE5	1
DONSON	1
E2F3	1
ECT2	1
IKBIP	1
ZBED3	1
RBBP8	1
GORAB	1
ATL2	1
DNA2	1
ZNF260	1
CDC7	1
MAST1	1
HTR6	1
SRF	1
FADD	1
VEGFC	1
THUMPD1	1
GPR107	1
WAS	1
TMEM109	1
TGFBRAP1	1
KIAA0391	1
SALL1	1
UNC119B	1
YWHAH	1
EI24	1
FANCD2	1
ARIH2	1
ACTR10	1
MKL2	1
MOCS2	1
TFCP2	1
SNX5	1
IKBKAP	1
UTP14C	1
TRMT1L	1
TMEM126B	1
ATMIN	1
DPF2	1
PARP2	1
UTP20	1
ZFP37	1
ZW10	1
VPS36	1
POF1B	1
MRPL3	1
STT3A	1
GOSR1	1
ERH	1
TBL2	1
HPS5	1
UBE4A	1
DSCR3	1
TOR1A	1
BMP8B	1
ERCC4	1
FANCM	1
PURG	1
DNAJC18	1
HSD17B4	1
ZKSCAN4	1
ZNF287	1
MAPKBP1	1
TMEM106B	1
BTRC	1
GPATCH8	1
SFMBT1	1

Supplementary Table 2.

GSEA, leading edge and IPA pathway analyses performed on tumor samples from a selected TCGA-COAD cohort stratified according to miR-27a levels.

2F) overrepresented pathways in the set of genes identified by leading-edge-analysis in high-miR-27a CRCs;

Â© 2000-2017 QIAGEN. All rights reserved.

Ingenuity Canonical Pathways	-log(B-H p-value)	Ratio	z-score	Molecules
**Oxidative Phosphorylation**	6,17E+01	3,76E-01	NaN	NDUFA4,ATP5G1,ATP5H,COX6A1,NDUFA7,ATP5D,COX6C,UQCRH,COX5B,NDUFB5,COX8A,ATP5L,NDUFB8,NDUFA1,ATP5E,NDUFA2,NDUFS6,NDUFS2,NDUFAB1,ATP5I,COX4I1,ATP5J,SDHA,ATP5G2,COX6B1,NDUFS7,COX7C,ATP5O,SURF1,NDUFB1,UQCR11,ATP5C1,NDUFV2,NDUFS8,UQCR10,NDUFB7,NDUFA3,NDUFS3,UQCRC1,NDUFB2,UQCRQ
**Mitochondrial Dysfunction**	5,64E+01	2,51E-01	NaN	NDUFA4,ATP5G1,ATP5H,COX6A1,ATP5D,NDUFA7,COX6C,UQCRH,COX5B,NDUFB5,COX8A,ATP5L,NDUFB8,NDUFA1,ATP5E,NDUFA2,VDAC2,NDUFS6,NDUFS2,GPX4,NDUFAB1,ATP5I,COX4I1,ATP5J,SDHA,ATP5G2,COX6B1,NDUFS7,COX7C,ATP5O,SURF1,NDUFB1,UQCR11,ATP5C1,NDUFV2,NDUFS8,UQCR10,NDUFB7,NDUFA3,NDUFS3,UQCRC1,NDUFB2,UQCRQ
**EIF2 Signaling**	1,43E+01	8,60E-02	NaN	RPS19,RPL36A,RPL29,RPLP2,RPL35A,RPL7A,RPL28,EIF3G,EIF3F,RPS16,RPL18A,RPL13,RPS25,RPS2,RPL36,RPS12,RPL18,EIF3K,RPS14
**Regulation of eIF4 and p70S6K Signaling**	5,70E+00	6,37E-02	NaN	EIF3G,RPS19,EIF3F,PPP2R1A,RPS16,RPS25,RPS2,RPS12,RPS14,EIF3K
**mTOR Signaling**	4,83E+00	5,03E-02	NaN	EIF3G,RPS19,EIF3F,PPP2R1A,RPS16,RPS25,RPS2,RPS12,RPS14,EIF3K
**Thiosulfate Disproportionation III (Rhodanese**	2,44E+00	6,67E-01	NaN	MPST,TST
DNA damage-induced 14-3-3Ïƒ Signaling	8,15E-01	1,05E-01	NaN	TP53,SFN
Alanine Biosynthesis III	8,15E-01	1,00E+00	NaN	NFS1
Pyrimidine Deoxyribonucleotides De Novo Bio:	8,15E-01	8,70E-02	NaN	NME3,NME2
Glutathione Redox Reactions I	8,15E-01	8,33E-02	NaN	GPX4,GSTP1
Tryptophan Degradation X (Mammalian, via Tr	8,15E-01	8,00E-02	NaN	ALDH4A1,AKR1A1
Aryl Hydrocarbon Receptor Signaling	8,15E-01	2,86E-02	NaN	TP53,ALDH4A1,GSTP1,SMARCA4
Proline Degradation	8,15E-01	5,00E-01	NaN	ALDH4A1
L-cysteine Degradation III	8,15E-01	5,00E-01	NaN	MPST
4-hydroxyproline Degradation I	8,15E-01	5,00E-01	NaN	ALDH4A1
Adenine and Adenosine Salvage I	8,15E-01	5,00E-01	NaN	APRT
Apoptosis Signaling	7,37E-01	3,37E-02	NaN	TP53,ENDOG,LMNA
Regulation of Actin-based Motility by Rho	7,37E-01	3,30E-02	NaN	PFN1,BAIAP2,ARHGDIA
Methylglyoxal Degradation I	7,37E-01	3,33E-01	NaN	HAGHL
D-glucuronate Degradation I	7,37E-01	3,33E-01	NaN	AKR1A1
Ethanol Degradation II	7,05E-01	5,41E-02	NaN	ALDH4A1,AKR1A1
tRNA Charging	7,05E-01	5,13E-02	NaN	FARSA,QARS
Arginine Degradation I (Arginase Pathway)	7,05E-01	2,50E-01	NaN	ALDH4A1
Molybdenum Cofactor Biosynthesis	7,05E-01	2,50E-01	NaN	NFS1
Noradrenaline and Adrenaline Degradation	7,05E-01	5,00E-02	NaN	ALDH4A1,AKR1A1
Mechanisms of Viral Exit from Host Cells	7,02E-01	4,88E-02	NaN	CHMP2A,ACTG1
Pyrimidine Ribonucleotides Interconversion	6,79E-01	4,65E-02	NaN	NME3,NME2
NRF2-mediated Oxidative Stress Response	6,77E-01	2,07E-02	NaN	AKR1A1,TXN,ACTG1,GSTP1
Pyrimidine Ribonucleotides De Novo Biosynthe	6,77E-01	4,44E-02	NaN	NME3,NME2
ILK Signaling	6,77E-01	2,04E-02	NaN	PPP2R1A,TMSB10/TMSB4X,ACTG1,NACA
Cell Cycle: G2/M DNA Damage Checkpoint Reg	6,57E-01	4,08E-02	NaN	TP53,SFN
Thioredoxin Pathway	6,57E-01	1,67E-01	NaN	TXN
Pentose Phosphate Pathway (Non-oxidative Br	6,57E-01	1,67E-01	NaN	TALDO1
RhoA Signaling	6,47E-01	2,46E-02	NaN	PFN1,BAIAP2,ACTG1
PI3K/AKT Signaling	6,42E-01	2,42E-02	NaN	TP53,PPP2R1A,SFN
Role of CHK Proteins in Cell Cycle Checkpoint C	6,09E-01	3,64E-02	NaN	TP53,PPP2R1A
Actin Cytoskeleton Signaling	5,66E-01	1,75E-02	NaN	PFN1,BAIAP2,TMSB10/TMSB4X,ACTG1
Sucrose Degradation V (Mammalian)	5,46E-01	1,11E-01	NaN	ALDOA
Hereditary Breast Cancer Signaling	5,45E-01	2,11E-02	NaN	TP53,SFN,SMARCA4
Huntington's Disease Signaling	5,45E-01	1,66E-02	NaN	TP53,ATP5J,SDHA,RACK1
Epithelial Adherens Junction Signaling	5,45E-01	2,05E-02	NaN	BAIAP2,JUP,ACTG1
Pentose Phosphate Pathway	5,45E-01	1,00E-01	NaN	TALDO1
Myc Mediated Apoptosis Signaling	5,00E-01	2,86E-02	NaN	TP53,SFN
Acyl-CoA Hydrolysis	4,99E-01	8,33E-02	NaN	ACOT7
BER pathway	4,99E-01	8,33E-02	NaN	POLG
Serotonin Degradation	4,87E-01	2,70E-02	NaN	ALDH4A1,AKR1A1
Tight Junction Signaling	4,80E-01	1,80E-02	NaN	F11R,PPP2R1A,ACTG1
Role of BRCA1 in DNA Damage Response	4,80E-01	2,56E-02	NaN	TP53,SMARCA4
Cyclins and Cell Cycle Regulation	4,80E-01	2,56E-02	NaN	TP53,PPP2R1A
Isoleucine Degradation I	4,80E-01	7,14E-02	NaN	ECHS1
Vitamin-C Transport	4,67E-01	6,67E-02	NaN	TXN
RhoGDI Signaling	4,67E-01	1,73E-02	NaN	RACK1,ARHGDIA,ACTG1
Granzyme B Signaling	4,63E-01	6,25E-02	NaN	ENDOG
Methylglyoxal Degradation III	4,63E-01	6,25E-02	NaN	AKR1A1
Sertoli Cell-Sertoli Cell Junction Signaling	4,63E-01	1,69E-02	NaN	F11R,JUP,ACTG1
HIPPO signaling	4,52E-01	2,30E-02	NaN	PPP2R1A,SFN
Valine Degradation I	4,46E-01	5,56E-02	NaN	ECHS1
Acute Myeloid Leukemia Signaling	4,46E-01	2,20E-02	NaN	IDH2,JUP
Death Receptor Signaling	4,46E-01	2,17E-02	NaN	LMNA,ACTG1
GADD45 Signaling	4,46E-01	5,26E-02	NaN	TP53
Histamine Degradation	4,46E-01	5,26E-02	NaN	ALDH4A1
Prostate Cancer Signaling	4,46E-01	2,13E-02	NaN	TP53,GSTP1
Salvage Pathways of Pyrimidine Ribonucleotid	4,46E-01	2,11E-02	NaN	NME3,NME2
DNA Methylation and Transcriptional Repressi	4,46E-01	5,00E-02	NaN	MBD3
Sumoylation Pathway	4,46E-01	2,08E-02	NaN	TP53,ARHGDIA
Oxidative Ethanol Degradation III	4,34E-01	4,76E-02	NaN	ALDH4A1
Breast Cancer Regulation by Stathmin1	4,34E-01	1,48E-02	NaN	TP53,PPP2R1A,RACK1
Fatty Acid Î±-oxidation	4,34E-01	4,55E-02	NaN	ALDH4A1
Antioxidant Action of Vitamin C	4,34E-01	1,94E-02	NaN	TXN,PAFAH1B3
VEGF Signaling	4,34E-01	1,94E-02	NaN	SFN,ACTG1
TCA Cycle II (Eukaryotic)	4,34E-01	4,35E-02	NaN	SDHA
Putrescine Degradation III	4,34E-01	4,35E-02	NaN	ALDH4A1
Tumoricidal Function of Hepatic Natural Killer	4,31E-01	4,17E-02	NaN	ENDOG
Ethanol Degradation IV	4,31E-01	4,00E-02	NaN	ALDH4A1
Glioma Signaling	4,31E-01	1,82E-02	NaN	TP53,IDH2
Amyotrophic Lateral Sclerosis Signaling	4,31E-01	1,80E-02	NaN	TP53,SSR4
p53 Signaling	4,31E-01	1,80E-02	NaN	TP53,SFN
Telomerase Signaling	4,31E-01	1,80E-02	NaN	TP53,PPP2R1A
Glycolysis I	4,31E-01	3,85E-02	NaN	ALDOA
Gluconeogenesis I	4,31E-01	3,85E-02	NaN	ALDOA
HIF1Î± Signaling	4,15E-01	1,74E-02	NaN	TP53,ELOB
p38 MAPK Signaling	4,09E-01	1,71E-02	NaN	TP53,MKNK2
Glutathione-mediated Detoxification	3,90E-01	3,33E-02	NaN	GSTP1
G Protein Signaling Mediated by Tubby	3,74E-01	3,12E-02	NaN	RACK1
Fatty Acid Î²-oxidation I	3,74E-01	3,12E-02	NaN	ECHS1
Inhibition of Angiogenesis by TSP1	3,64E-01	2,94E-02	NaN	TP53

p70S6K Signaling	3,64E-01	1,53E-02	NaN	PPP2R1A,SFN
Signaling by Rho Family GTPases	3,64E-01	1,21E-02	NaN	RACK1,BAIAP2,ACTG1
Cell Cycle Regulation by BTG Family Proteins	3,64E-01	2,86E-02	NaN	PPP2R1A
Dopamine Degradation	3,64E-01	2,86E-02	NaN	ALDH4A1
Cardiac Î²-adrenergic Signaling	3,49E-01	1,46E-02	NaN	PPP2R1A,RACK1
Role of PKR in Interferon Induction and Antivir	3,35E-01	2,50E-02	NaN	TP53
Thyroid Cancer Signaling	3,35E-01	2,50E-02	NaN	TP53
Phagosome Maturation	3,35E-01	1,39E-02	NaN	DYNLL1,PRDX2
MIF Regulation of Innate Immunity	3,35E-01	2,44E-02	NaN	TP53
Stearate Biosynthesis I (Animals)	3,35E-01	2,44E-02	NaN	ACOT7
Synaptic Long Term Depression	3,35E-01	1,37E-02	NaN	PPP2R1A,PAFAH1B3
UVC-Induced MAPK Signaling	3,31E-01	2,38E-02	NaN	TP53
Role of p14/p19ARF in Tumor Suppression	3,26E-01	2,33E-02	NaN	TP53
Role of Oct4 in Mammalian Embryonic Stem C	3,05E-01	2,17E-02	NaN	TP53
Xenobiotic Metabolism Signaling	2,97E-01	1,05E-02	NaN	ALDH4A1,PPP2R1A,GSTP1
Cancer Drug Resistance By Drug Efflux	2,90E-01	2,04E-02	NaN	TP53
Wnt/Î²-catenin Signaling	2,71E-01	1,18E-02	NaN	TP53,PPP2R1A
Tec Kinase Signaling	2,71E-01	1,18E-02	NaN	RACK1,ACTG1
Germ Cell-Sertoli Cell Junction Signaling	2,65E-01	1,16E-02	NaN	JUP,ACTG1
Melanoma Signaling	2,64E-01	1,82E-02	NaN	TP53
Actin Nucleation by ARP-WASP Complex	2,61E-01	1,79E-02	NaN	BAIAP2
MSP-RON Signaling Pathway	2,54E-01	1,69E-02	NaN	ACTG1
Induction of Apoptosis by HIV1	2,54E-01	1,67E-02	NaN	TP53
Retinoic acid Mediated Apoptosis Signaling	2,54E-01	1,64E-02	NaN	DAP3
Phospholipases	2,54E-01	1,61E-02	NaN	PAFAH1B3
AMPK Signaling	2,54E-01	1,06E-02	NaN	PPP2R1A,SMARCA4
ERK5 Signaling	2,54E-01	1,59E-02	NaN	SFN
PCP pathway	2,54E-01	1,59E-02	NaN	PFN1
RAR Activation	2,54E-01	1,05E-02	NaN	RPL7A,SMARCA4
Endometrial Cancer Signaling	2,54E-01	1,56E-02	NaN	TP53
Cell Cycle: G1/S Checkpoint Regulation	2,54E-01	1,56E-02	NaN	TP53
Hypoxia Signaling in the Cardiovascular Systen	2,54E-01	1,54E-02	NaN	TP53
Mitotic Roles of Polo-Like Kinase	2,54E-01	1,52E-02	NaN	PPP2R1A
UVB-Induced MAPK Signaling	2,54E-01	1,52E-02	NaN	TP53
Eicosanoid Signaling	2,54E-01	1,49E-02	NaN	PAFAH1B3
ERK/MAPK Signaling	2,54E-01	1,01E-02	NaN	PPP2R1A,MKNK2
CCR5 Signaling in Macrophages	2,54E-01	1,45E-02	NaN	RACK1
Agrin Interactions at Neuromuscular Junction	2,54E-01	1,45E-02	NaN	ACTG1
Remodeling of Epithelial Adherens Junctions	2,54E-01	1,45E-02	NaN	ACTG1
Caveolar-mediated Endocytosis Signaling	2,51E-01	1,41E-02	NaN	ACTG1
Basal Cell Carcinoma Signaling	2,51E-01	1,39E-02	NaN	TP53
Role of MAPK Signaling in the Pathogenesis of	2,51E-01	1,39E-02	NaN	PAFAH1B3
Ephrin B Signaling	2,51E-01	1,37E-02	NaN	RACK1
GM-CSF Signaling	2,51E-01	1,37E-02	NaN	RACK1
Leukocyte Extravasation Signaling	2,50E-01	9,52E-03	NaN	F11R,ACTG1
Antiproliferative Role of Somatostatin Recepto	2,49E-01	1,33E-02	NaN	RACK1
Non-Small Cell Lung Cancer Signaling	2,46E-01	1,30E-02	NaN	TP53
Dopamine Receptor Signaling	2,46E-01	1,30E-02	NaN	PPP2R1A
Integrin Signaling	2,42E-01	9,13E-03	NaN	PFN1,ACTG1
ATM Signaling	2,42E-01	1,25E-02	NaN	TP53
LPS/IL-1 Mediated Inhibition of RXR Function	2,42E-01	9,01E-03	NaN	ALDH4A1,GSTP1
Renal Cell Carcinoma Signaling	2,42E-01	1,23E-02	NaN	ELOB
Systemic Lupus Erythematosus Signaling	2,39E-01	8,89E-03	NaN	SNRNP70,SNRPD2
Small Cell Lung Cancer Signaling	2,39E-01	1,19E-02	NaN	TP53
PEDF Signaling	2,39E-01	1,19E-02	NaN	TP53
Î±-Adrenergic Signaling	2,36E-01	1,15E-02	NaN	RACK1
Bladder Cancer Signaling	2,36E-01	1,15E-02	NaN	TP53
G Beta Gamma Signaling	2,36E-01	1,14E-02	NaN	RACK1
HER-2 Signaling in Breast Cancer	2,36E-01	1,14E-02	NaN	TP53
Crosstalk between Dendritic Cells and Natural	2,35E-01	1,12E-02	NaN	ACTG1
IL-1 Signaling	2,32E-01	1,10E-02	NaN	RACK1
Reelin Signaling in Neurons	2,32E-01	1,09E-02	NaN	PAFAH1B3
Ceramide Signaling	2,32E-01	1,08E-02	NaN	PPP2R1A
FcÎ³ Receptor-mediated Phagocytosis in Macro	2,32E-01	1,08E-02	NaN	ACTG1
Colorectal Cancer Metastasis Signaling	2,26E-01	8,10E-03	NaN	TP53,RACK1
CTLA4 Signaling in Cytotoxic T Lymphocytes	2,24E-01	1,01E-02	NaN	PPP2R1A
CDK5 Signaling	2,24E-01	1,01E-02	NaN	PPP2R1A
FAK Signaling	2,24E-01	1,01E-02	NaN	ACTG1
Virus Entry via Endocytic Pathways	2,20E-01	9,80E-03	NaN	ACTG1
UVA-Induced MAPK Signaling	2,20E-01	9,80E-03	NaN	TP53
Chronic Myeloid Leukemia Signaling	2,19E-01	9,62E-03	NaN	TP53
SAPK/JNK Signaling	2,19E-01	9,62E-03	NaN	TP53
Mouse Embryonic Stem Cell Pluripotency	2,19E-01	9,43E-03	NaN	TP53
IGF-1 Signaling	2,19E-01	9,43E-03	NaN	SFN
GÎ±s Signaling	2,13E-01	9,17E-03	NaN	RACK1
Androgen Signaling	2,10E-01	9,01E-03	NaN	RACK1
Paxillin Signaling	2,08E-01	8,85E-03	NaN	ACTG1
Rac Signaling	2,08E-01	8,55E-03	NaN	BAIAP2
NGF Signaling	2,08E-01	8,55E-03	NaN	TP53
Axonal Guidance Signaling	2,08E-01	6,68E-03	NaN	PFN1,RACK1,BAIAP2
Pancreatic Adenocarcinoma Signaling	2,08E-01	8,47E-03	NaN	TP53
GÎ±i Signaling	2,08E-01	8,33E-03	NaN	RACK1
LXR/RXR Activation	2,08E-01	8,26E-03	NaN	ECHS1
fMLP Signaling in Neutrophils	2,08E-01	8,26E-03	NaN	RACK1
Role of NANOG in Mammalian Embryonic Sten	2,08E-01	8,20E-03	NaN	TP53
Role of Tissue Factor in Cancer	2,08E-01	8,20E-03	NaN	TP53
Sperm Motility	2,04E-01	8,00E-03	NaN	PAFAH1B3
Atherosclerosis Signaling	2,04E-01	7,87E-03	NaN	PAFAH1B3
Estrogen Receptor Signaling	2,04E-01	7,81E-03	NaN	SMARCA4
14-3-3-mediated Signaling	2,04E-01	7,69E-03	NaN	SFN
CCR3 Signaling in Eosinophils	2,04E-01	7,69E-03	NaN	RACK1
Cellular Effects of Sildenafil (Viagra)	2,04E-01	7,69E-03	NaN	ACTG1
P2Y Purigenic Receptor Signaling Pathway	2,02E-01	7,58E-03	NaN	RACK1
Adipogenesis pathway	2,00E-01	7,46E-03	NaN	TP53
Ovarian Cancer Signaling	1,83E-01	6,94E-03	NaN	TP53
Relaxin Signaling	1,71E-01	6,58E-03	NaN	RACK1
Glioblastoma Multiforme Signaling	1,62E-01	6,29E-03	NaN	TP53
GÎ±q Signaling	1,62E-01	6,25E-03	NaN	RACK1
Dopamine-DARPP32 Feedback in cAMP Signali	1,61E-01	6,17E-03	NaN	PPP2R1A
CXCR4 Signaling	1,59E-01	6,06E-03	NaN	RACK1

Supplementary Table 2.

GSEA, leading edge and IPA pathway analyses performed on tumor samples from a selected TCGA-COAD cohort stratified according to miR-27a levels.

2G) overrepresented pathways in the set of genes identified by leading-edge-analysis in lowmiR- 27a CRCs. In bold are represented the significantly overrepresented pathways (p <0.05; Benjiamini Hochberg). Ratio, fraction of genes selected for analysis over total genes annotated by IPA in the considered pathway.

Â© 2000-2017 QIAGEN. All rights reserved.

Ingenuity Canonical Pathways	-log(B-H p-value)	Ratio	z-score	Molecules
**Molecular Mechanisms of Cancer**	2,35E+01	2,81E-01	NaN	RAF1,TGFBR1,PIK3R1,SOS2,TAB2,RBL1,PAK1,PIK3CG,PLCB1,GNA13,PRKD3,PRKD1,ITGA4,CCNE2,PRKCQ,TFDP1,CREBBP,RALB,ITGA5,FGFR2,RAP1A,MAX,IRS1,ARHGEF6,PIK3CD,FYN,TCF4,BMPR2,CRK,FZD1,CHEK1,PIK3C3,SOS1,E2F5,ARHGEF3,ITGB1,PRKDC,PMAIP1,GNA12,GNAQ,GNAI3,PAK3,RASGRP1,RBPJ,BMP7,PRKCB,BIRC2,FZD7,MAP2K4,PRKACB,JAK1,SMAD3,PTK2,TGFBR2,TGFB1,GSK3B,BIRC3,SMAD1,FZD2,CASP3,FGFR1,RHOJ,APC,PIK3R3,ADCY9,RHOQ,PTPN11,RND3,CDC42,RHOA,FZD6,TGFB3,PRKCH,LEF1,CDK2,FNBP1,BMP2,HIF1A,SMAD5,NFKB1,RHOH,CDKN2B,BCL2,NFKBIA,BMPR1A,SMAD4,AKT3,MAP2K1,PRKCA,ARHGEF12,NRAS,PIK3C2A,HAT1,GNAI1,NBN,FZD8,RRAS2,PRKCI,PRKAR2B,MAPK14,FOXO1,NF1,PAK2,BCL2L11,WNT5A
**Hepatic Fibrosis / Hepatic Stellate Cell Activa**	2,24E+01	3,72E-01	NaN	CCR5,TGFBR1,CTGF,SMAD3,COL8A1,IL6,CXCL9,TGFBR2,TGFB1,IL1RAP,PDGFRB,TIMP2,COL4A1,FGFR1,IL6R,FGFR2,IGFBP5,IFNGR1,IFNAR2,MYL9,COL6A3,ACTA2,TGFB3,IGFBP3,IL10RA,CD14,PDGFD,TNF,CCR7,COL3A1,ICAM1,COL4A5,FN1,LEPR,COL10A1,CCL5,COL4A2,NFKB1,PDGFC,COL15A1,BCL2,COL1A2,COL6A1,CCL2,IGF1,HGF,PDGFRA,SMAD4,COL18A1,STAT1,EGFR,COL5A2,VCAM1,FLT1,EDNRB,IL10,COL12A1,IL1R1,FGF1,COL1A1,LY96,CSF1,CCL21,EDNRA,COL11A1,KDR,A2M,MMP9
**Leukocyte Extravasation Signaling**	2,23E+01	3,48E-01	NaN	MAP2K4,MMP16,PIK3R1,WASL,JAM2,CXCL12,ITGB3,ROCK2,PTK2,PIK3CG,CYBB,DLC1,PRKD3,PRKD1,ITK,TIMP2,ITGA4,PRKCQ,CRKL,FGFR1,RDX,THY1,ITGA5,FGFR2,NCF4,RAP1A,ARHGAP5,PIK3R3,ITGAM,PTPN11,ACTA2,CDH5,ICAM3,CDC42,JAM3,RHOA,PLCG2,IRS1,NCF2,PECAM1,ITGA1,PRKCH,PIK3CD,ICAM1,MMP14,CRK,RHOH,PIK3C3,VCL,MMP12,ACTC1,ACTN1,PRKCA,ITGB1,TIMP3,VCAM1,PIK3C2A,CXCR4,GNAI1,ITGAL,SELPLG,BTK,ROCK1,ITGB2,GNAI3,WIPF1,EDIL3,PRKCI,MAPK14,RASGRP1,MMP9,MSN,PRKCB
**Integrin Signaling**	2,12E+01	3,33E-01	NaN	MAP2K4,RAF1,PIK3R1,SOS2,WASL,NCK1,ITGB3,PTEN,PTK2,PAK1,ITGA9,PIK3CG,CAV1,ITGAV,GSK3B,ITGA4,ACTR2,TSPAN5,CRKL,FGFR1,RALB,ITGA5,FGFR2,TSPAN2,RHOJ,RAP1A,ARHGAP5,PIK3R3,MYL9,RHOQ,ITGAM,PTPN11,RND3,ACTA2,CDC42,RHOA,PLCG2,IRS1,ITGA1,PIK3CD,FNBP1,FYN,TSPAN7,PPP1CB,CRK,MYLK,ITGB8,RHOH,ACTR3,PPP1R12A,PIK3C3,SOS1,PFN2,AKT3,VCL,MAP2K1,ACTC1,ACTN1,MYL12A,ITGB1,PARVA,NRAS,PIK3C2A,ASAP1,ITGAL,ROCK1,ITGB2,WIPF1,RRAS2,PAK3,LIMS1,PAK2,NEDD9
**Axonal Guidance Signaling**	2,12E+01	2,49E-01	NaN	SLIT3,KLC1,RAF1,ITSN1,PIK3R1,SOS2,NCK1,ADAMTS2,GNB4,PAK1,PIK3CG,PLCB1,GNA13,PRKD3,PRKD1,GNG12,ITGA4,ACTR2,PRKCQ,KALRN,CRKL,TUBB2A,ITGA5,FGFR2,PLCL2,RAP1A,MYL9,ADAMTS6,IRS1,PLCG2,ARHGEF6,PIK3CD,PDGFD,NRP1,FYN,BDNF,CRK,FZD1,EIF4E,ACTR3,IGF1,PIK3C3,SOS1,PFN2,PPP3CA,MYL12A,ITGB1,NRP2,GNA12,GNAQ,EPHA3,SLIT2,GNAI3,WIPF1,TUBA1A,PAK3,ADAM10,BMP7,SEMA3C,FZD7,PRKCB,PRKACB,WASL,UNC5B,CXCL12,PTK2,ROCK2,SEMA6D,CFL2,ADAM28,GSK3B,ADAMTS5,FZD2,PAPPA,ADAMTS1,FGFR1,GNG2,HHIP,PIK3R3,SDCBP,PTPN11,ADAM12,CDC42,RHOA,FZD6,PRKCH,ENPEP,BMP2,ROBO1,PDGFC,GNG11,SDC2,AKT3,SEMA4A,MAP2K1,PRKCA,PLXNC1,ARHGEF12,NRAS,PIK3C2A,CXCR4,GNAI1,PLXND1,ROCK1,FZD8,RRAS2,PRKCI,PRKAR2B,PAK2,ADAM9,MMP9,WNT5A
**Role of NFAT in Regulation of the Immune R**	1,95E+01	3,46E-01	NaN	FCGR2C,RAF1,CD4,PIK3R1,SOS2,FCER1A,FCGR1A,CD28,GNB4,PIK3CG,PLCB1,XPO1,GNA13,GSK3B,GNG12,ITK,PRKCQ,FGFR1,GNG2,FGFR2,CD79A,ITPR1,ATF2,PIK3R3,CD3G,PTPN11,PLCG2,IRS1,ZAP70,CD86,PIK3CD,LCP2,BLNK,FYN,FCGR2B,NFKB1,GNG11,NFKBIA,PIK3C3,SOS1,AKT3,MAP2K1,FCGR3A/FCGR3B,PPP3CA,FCGR1B,NRAS,PIK3C2A,FCGR2A,GNA12,CSNK1G3,MEF2A,GNAQ,GNAI1,CD3D,BTK,GNAI3,RCAN1,CALM1 (includes others),RRAS2,CD80,FCER1G,LYN,MEF2C,RCAN2
**Role of Macrophages, Fibroblasts and Endot**	1,95E+01	2,78E-01	NaN	MAP2K4,RAF1,SOCS3,TRAF3,PIK3R1,CXCL12,LTB,IL6,FCGR1A,IL18R1,ROCK2,TGFB1,PIK3CG,TLR1,PLCB1,DKK2,GSK3B,IL1RAP,FZD2,PRKD3,PRKD1,SFRP4,PRKCQ,FGFR1,CREBBP,IL6R,FGFR2,PLCL2,IL7,APC,ATF2,IL33,PIK3R3,PTPN11,IL1RN,RHOA,PLCG2,IRS1,FZD6,PRKCH,PIK3CD,LEF1,PDGFD,TNF,IL6ST,TCF4,ICAM1,SFRP2,FN1,FRZB,FZD1,CCL5,NFKB1,PDGFC,NFKBIA,CCL2,DKK3,PIK3C3,CREB1,TLR7,AKT3,MAP2K1,FCGR3A/FCGR3B,PPP3CA,PRKCA,VCAM1,C5AR1,NRAS,PIK3C2A,IL10,DAAM1,GNAQ,IL1R1,ROCK1,IL16,FZD8,CALM1 (includes others),RRAS2,PRKCI,RIPK1,MAPK14,CSF1,FZD7,PRKCB,RYK,WNT5A
**Phospholipase C Signaling**	1,95E+01	3,08E-01	NaN	RAF1,FCGR2C,SOS2,GNB4,PLCB1,GNA13,PRKD3,PRKD1,GNG12,ITGA4,ITK,HDAC4,PLD3,PRKCQ,GNG2,RALB,CREBBP,ITGA5,PPP1R14A,CD79A,RHOJ,ITPR1,RAP1A,PLD1,ATF2,MYL9,ADCY9,CD3G,MARCKS,RHOQ,RND3,RHOA,PLCG2,ZAP70,ARHGEF6,PRKCH,FNBP1,LCP2,BLNK,FYN,RPS6KA3,PPP1CB,FCGR2B,NFKB1,RHOH,TGM2,HMOX1,GNG11,PPP1R12A,SOS1,CREB1,ARHGEF3,MAP2K1,PPP3CA,PRKCA,MYL12A,ITGB1,HDAC9,NRAS,ARHGEF12,FCGR2A,MEF2A,GNAQ,CD3D,PLD4,BTK,PLA2G4A,CALM1 (includes others),RRAS2,PRKCI,FCER1G,LYN,MEF2C,PRKCB
**IL-8 Signaling**	1,95E+01	3,35E-01	NaN	MAP2K4,RAF1,PIK3R1,ITGB3,ROCK2,PTK2,GNB4,PIK3CG,ITGAV,CYBB,GNA13,PRKD3,PRKD1,TEK,GNG12,NOX4,PLD3,PRKCQ,FGFR1,GNG2,FGFR2,RHOJ,PLD1,PIK3R3,MYL9,ITGAM,RHOQ,RND3,PTPN11,RHOA,IRS1,NCF2,PRKCH,PIK3CD,FNBP1,ANGPT2,ICAM1,NFKB1,IQGAP1,PDGFC,RHOH,BCL2,HMOX1,GNG11,PIK3C3,AKT3,MAP2K1,PRKCA,EGFR,VCAM1,NRAS,ANGPT1,FLT1,PIK3C2A,GNA12,GNAI1,PLD4,ROCK1,GNAI3,ITGB2,RRAS2,PRKCI,PAK2,KDR,MMP9,PRKCB
**Actin Cytoskeleton Signaling**	1,90E+01	3,11E-01	NaN	RAF1,F2R,PIK3R1,SOS2,WASL,SSH1,ROCK2,PTK2,PAK1,CFL2,PIK3CG,GNA13,GNG12,MATK,ITGA4,ACTR2,TIAM1,FGF9,CRKL,FGFR1,RDX,ITGA5,FGFR2,APC,PIK3R3,MYL9,PIP5K1A,PTPN11,ACTA2,CYFIP1,CDC42,RHOA,IRS1,ARHGEF6,CD14,PIK3CD,PDGFD,PIP4K2A,FN1,TRIO,PPP1CB,CRK,MYLK,IQGAP1,PDGFC,IQGAP2,ACTR3,PPP1R12A,FLNA,PIK3C3,SOS1,PFN2,VCL,FGF7,MAP2K1,ACTC1,ACTN1,NCKAP1,MYL12A,ITGB1,NRAS,ARHGEF12,PIK3C2A,GNA12,FGF1,ROCK1,RRAS2,PAK3,PAK2,NCKAP1L,MSN
**Signaling by Rho Family GTPases**	1,81E+01	2,96E-01	NaN	MAP2K4,RAF1,PIK3R1,WASL,CLIP1,ROCK2,PTK2,GNB4,PAK1,CFL2,PIK3CG,CYBB,GNA13,CDH13,GNG12,ITGA4,ACTR2,NOX4,FGFR1,GNG2,RDX,ITGA5,FGFR2,RHOJ,PLD1,PIK3R3,MYL9,PIP5K1A,CDH2,RHOQ,PTPN11,RND3,ACTA2,CDH5,CYFIP1,CDC42,RHOA,IRS1,ARHGEF6,NCF2,PIK3CD,PIP4K2A,FNBP1,WASF3,MYLK,NFKB1,IQGAP1,RHOH,CDH11,ACTR3,GNG11,PPP1R12A,PIK3C3,ARHGEF3,MAP2K1,ACTC1,MYL12A,ITGB1,NEDD4,ARHGEF12,PIK3C2A,GNA12,GNAQ,GNAI1,VIM,CDC42EP3,ROCK1,GNAI3,WIPF1,PRKCI,PAK3,PAK2,MSN
**Tec Kinase Signaling**	1,68E+01	3,35E-01	NaN	MAP2K4,JAK1,PIK3R1,FCER1A,PTK2,GNB4,PAK1,PIK3CG,GNA13,PRKD3,PRKD1,GNG12,FGR,ITGA4,ITK,PRKCQ,FGFR1,GNG2,ITGA5,FGFR2,RHOJ,PIK3R3,RHOQ,RND3,ACTA2,PTPN11,RHOA,PLCG2,IRS1,PRKCH,PIK3CD,TNF,FNBP1,FYN,TNFSF10,NFKB1,RHOH,GNG11,PIK3C3,HCK,STAT1,ACTC1,PRKCA,ITGB1,PIK3C2A,GNA12,GNAQ,GNAI1,BTK,STAT4,GNAI3,PRKCI,PAK3,PAK2,FCER1G,LYN,PRKCB
**Th1 and Th2 Activation Pathway**	1,63E+01	3,19E-01	NaN	SOCS3,CCR5,TGFBR1,TNFRSF4,JAK1,TGFBR3,CD4,PIK3R1,MAF,IL6,CD8A,IL18R1,TGFBR2,CD28,TGFB1,PIK3CG,CD274,IL2RB,PRKCQ,FGFR1,IL6R,FGFR2,IFNGR1,IL33,PIK3R3,CD3G,PTPN11,IRS1,IL10RA,CXCR6,CD86,PIK3CD,TNFSF4,ICAM1,BMPR2,NFKB1,NFIL3,NOTCH2,PIK3C3,IL27RA,STAT1,CCR1,PIK3C2A,CXCR4,IL10,IKZF1,ACVR1,CD3D,TSLP,STAT4,ITGB2,NOTCH4,CD80,IL12B,ICOS,S1PR1,IL2RA,GATA3,ACVR2A
**CXCR4 Signaling**	1,61E+01	3,33E-01	NaN	MAP2K4,RAF1,CD4,PIK3R1,CXCL12,PTK2,ROCK2,GNB4,PAK1,PIK3CG,PLCB1,GNA13,PRKD3,PRKD1,GNG12,PRKCQ,FGFR1,GNG2,FGFR2,RHOJ,ITPR1,MYL9,PIK3R3,ADCY9,RHOQ,RND3,PTPN11,RHOA,IRS1,PRKCH,PIK3CD,ELMO1,FNBP1,CRK,RHOH,GNG11,PIK3C3,AKT3,MAP2K1,PRKCA,MYL12A,NRAS,PIK3C2A,CXCR4,GNA12,GNAQ,GNAI1,ROCK1,GNAI3,PRKCI,RRAS2,PAK3,PAK2,LYN,PRKCB
**Role of Osteoblasts, Osteoclasts and Chondr**	1,61E+01	2,89E-01	NaN	MAP2K4,PIK3R1,TAB2,ACP5,IL6,ITGB3,IL18R1,TGFB1,RUNX2,PIK3CG,DKK2,GSK3B,IL1RAP,ADAMTS5,BIRC3,SMAD1,FZD2,SFRP4,SPP1,FGFR1,ITGA5,FGFR2,IL7,APC,IL33,PIK3R3,PTPN11,IL1RN,IRS1,FZD6,LEF1,PIK3CD,TNF,TCF4,SFRP2,FRZB,MMP14,BMP2,BMPR2,FZD1,SMAD5,NFKB1,BCL2,NFKBIA,IGF1,BMPR1A,DKK3,PIK3C3,SMAD4,AKT3,PPP3CA,ITGB1,CTSK,PIK3C2A,IL10,IL1R1,CSF1R,FZD8,COL1A1,CALM1 (includes others),MAPK14,FOXO1,CSF1,BMP7,BIRC2,WNT5A,FZD7
**Phagosome Formation**	1,58E+01	3,77E-01	NaN	MRC1,FCGR2C,FN1,MSR1,PIK3R1,FCER1A,FCGR2B,FCGR1A,RHOH,PIK3CG,PIK3C3,TLR1,TLR7,PLCB1,PRKD3,FCGR3A/FCGR3B,PRKD1,FCGR1B,ITGA4,PRKCA,ITGB1,PRKCQ,PIK3C2A,FCGR2A,MRC2,FGFR1,ITGA5,FGFR2,RHOJ,PLCL2,PIK3R3,MARCKS,CLEC7A,RHOQ,PRKCI,RND3,PTPN11,SCARA3,RHOA,PLCG2,IRS1,FCER1G,PIK3CD,PRKCH,FNBP1,PRKCB
**Thrombin Signaling**	1,55E+01	3,00E-01	NaN	RAF1,F2R,PIK3R1,GATA2,ROCK2,PTK2,GNB4,PIK3CG,PLCB1,GNA13,PRKD3,PRKD1,GNG12,PRKCQ,FGFR1,GNG2,FGFR2,RHOJ,PLCL2,ITPR1,PIK3R3,MYL9,ADCY9,RHOQ,RND3,PTPN11,RHOA,PLCG2,IRS1,ARHGEF6,PRKCH,PIK3CD,FNBP1,PPP1CB,MYLK,NFKB1,RHOH,GNG11,PPP1R12A,PIK3C3,SOS1,CREB1,AKT3,ARHGEF3,MAP2K1,PRKCA,MYL12A,EGFR,NRAS,ARHGEF12,PIK3C2A,GNA12,GNAQ,GNAI1,ROCK1,GNAI3,RRAS2,MAPK14,PRKCI,GATA3,PRKCB
**Ephrin Receptor Signaling**	1,50E+01	3,16E-01	NaN	RAF1,ITSN1,WASL,SOS2,CXCL12,NCK1,PTK2,ROCK2,GNB4,PAK1,CFL2,PIK3CG,GNA13,GNG12,ITGA4,ACTR2,KALRN,CRKL,GNG2,CREBBP,ITGA5,RAP1A,ATF2,SDCBP,PTPN11,CDC42,RHOA,PDGFD,FYN,CRK,PDGFC,ACTR3,GNG11,SDC2,SORBS1,SOS1,CREB1,AKT3,MAP2K1,ITGB1,NRAS,ANGPT1,CXCR4,GNA12,GNAQ,GNAI1,EPHA3,FGF1,ROCK1,GNAI3,WIPF1,RRAS2,PAK3,PAK2,ADAM10
**Macropinocytosis Signaling**	1,50E+01	4,44E-01	NaN	MRC1,PIK3R1,ITGB8,PDGFC,ITGB3,RAB5A,PAK1,HGF,PIK3CG,PIK3C3,PRKD3,PRKD1,PRKCA,ITGB1,NRAS,PRKCQ,PIK3C2A,FGFR1,ITGA5,FGFR2,CSF1R,PIK3R3,ITGB2,PRKCI,RRAS2,PTPN11,CSF1,CDC42,RHOA,PLCG2,IRS1,CD14,PIK3CD,PRKCH,PDGFD,PRKCB

**Role of NFAT in Cardiac Hypertrophy**	1,49E+01	3,02E-01	NaN	MAP2K4,PRKACB,RAF1,TGFBR1,PIK3R1,SOS2,IL6,TGFBR2,GNB4,TGFB1,PIK3CG,PLCB1,GSK3B,PRKD3,PRKD1,GNG12,PRKCQ,HDAC4,FGFR1,GNG2,FGFR2,PLCL2,ITPR1,PIK3R3,ADCY9,PTPN11,PLCG2,IRS1,TGFB3,PRKCH,PIK3CD,SLC8A1,IL6ST,GNG11,IGF1,PIK3C3,SOS1,AKT3,MAP2K1,PPP3CA,PRKCA,HDAC9,NRAS,PIK3C2A,MAP3K1,MEF2A,GNAQ,GNAI1,RCAN1,GNAI3,CALM1 (includes others),PRKAR2B,MAPK14,PRKCI,RRAS2,MEF2C,RCAN2,PRKCB
**HGF Signaling**	1,47E+01	3,74E-01	NaN	MAP2K4,RAF1,PIK3R1,SOS2,IL6,PTK2,PAK1,HGF,PIK3CG,PIK3C3,SOS1,AKT3,MAP2K1,PRKD3,PRKD1,ITGA4,MAP3K2,PRKCA,ETS1,ITGB1,NRAS,PRKCQ,PIK3C2A,CRKL,FGFR1,MAP3K1,ITGA5,FGFR2,RAP1A,ATF2,PIK3R3,PRKCI,RRAS2,PTPN11,CDC42,PLCG2,IRS1,PIK3CD,MAP3K8,PRKCH,ELK3,CDK2,PRKCB
**ILK Signaling**	1,45E+01	2,96E-01	NaN	MAP2K4,PIK3R1,ITGB3,PTEN,PTK2,CFL2,PIK3CG,GSK3B,CASP3,FGFR1,CREBBP,FERMT2,SNAI1,FGFR2,RHOJ,ATF2,MYL9,PIK3R3,RHOQ,RND3,ACTA2,PTPN11,CDC42,PPP2R3A,RHOA,IRS1,ARHGEF6,LEF1,PIK3CD,TNF,FNBP1,SNAI2,FN1,BMP2,HIF1A,ITGB8,NFKB1,PDGFC,RHOH,PPP1R12A,FLNA,PIK3C3,CREB1,AKT3,VCL,ACTC1,ACTN1,ITGB1,PARVA,PIK3C2A,VIM,RPS6KA5,ITGB2,FLNC,LIMS1,PPP2R5E,PPP2R1B,MMP9
**Breast Cancer Regulation by Stathmin1**	1,44E+01	2,91E-01	NaN	PRKACB,RAF1,PIK3R1,SOS2,ROCK2,GNB4,PAK1,PIK3CG,PLCB1,GNA13,PRKD3,PRKD1,GNG12,CCNE2,PRKCQ,FGFR1,GNG2,TUBB2A,FGFR2,PPP1R14A,ITPR1,PIK3R3,ADCY9,PTPN11,CDC42,PPP2R3A,RHOA,IRS1,ARHGEF6,PRKCH,PIK3CD,UHMK1,CDK2,PPP1R3C,PPP1CB,GNG11,PPP1R12A,PIK3C3,SOS1,E2F5,RB1CC1,ARHGEF3,MAP2K1,PRKCA,NRAS,ARHGEF12,PIK3C2A,GNAQ,GNAI1,ROCK1,GNAI3,CALM1 (includes others),RRAS2,TUBA1A,PRKAR2B,PRKCI,PPP2R5E,PPP2R1B,PRKCB
**Colorectal Cancer Metastasis Signaling**	1,42E+01	2,67E-01	NaN	MAP2K4,PRKACB,JAK1,TGFBR1,SMAD3,MMP16,PIK3R1,SOS2,IL6,TGFBR2,GNB4,TGFB1,PIK3CG,TLR1,GSK3B,FZD2,GNG12,CASP3,FGFR1,GNG2,IL6R,FGFR2,IFNGR1,RHOJ,APC,PIK3R3,ADCY9,RHOQ,PTPN11,RND3,RHOA,IRS1,TGFB3,FZD6,LEF1,PIK3CD,TNF,FNBP1,IL6ST,TCF4,PTGER3,MMP14,FZD1,NFKB1,PDGFC,RHOH,ARRB1,GNG11,PIK3C3,SOS1,TLR7,SMAD4,AKT3,STAT1,MMP12,MAP2K1,EGFR,NRAS,PIK3C2A,FZD8,RRAS2,PRKAR2B,MSH6,MMP9,WNT5A,FZD7
**Th2 Pathway**	1,41E+01	3,27E-01	NaN	SOCS3,CCR5,JAK1,TGFBR1,TNFRSF4,CD4,PIK3R1,TGFBR3,MAF,TGFBR2,CD28,TGFB1,PIK3CG,IL2RB,PRKCQ,FGFR1,FGFR2,PIK3R3,IL33,CD3G,PTPN11,IRS1,CXCR6,CD86,PIK3CD,TNFSF4,ICAM1,BMPR2,NFKB1,NOTCH2,PIK3C3,CCR1,PIK3C2A,CXCR4,IL10,IKZF1,ACVR1,TSLP,CD3D,STAT4,ITGB2,NOTCH4,CD80,IL12B,ICOS,S1PR1,IL2RA,GATA3,ACVR2A
**NF-Î°B Activation by Viruses**	1,41E+01	4,19E-01	NaN	RAF1,CCR5,PIK3R1,CD4,NFKB1,ITGB3,NFKBIA,PIK3CG,PIK3C3,ITGAV,AKT3,PRKD3,PRKD1,ITGA4,PRKCA,ITGB1,NRAS,PRKCQ,PIK3C2A,FGFR1,MAP3K1,ITGA5,FGFR2,ITGAL,PIK3R3,ITGB2,RRAS2,PRKCI,RIPK1,PTPN11,IRS1,ITGA1,PIK3CD,PRKCH,EIF2AK2,PRKCB
**Regulation of the Epithelial-Mesenchymal Tr**	1,41E+01	2,96E-01	NaN	MAP2K4,RAF1,JAK1,TGFBR1,SMAD3,PIK3R1,SOS2,TGFBR2,TGFB1,PIK3CG,GSK3B,FZD2,PDGFRB,ETS1,FGF9,FGFR1,SNAI1,FGFR2,ZEB1,APC,PIK3R3,CDH2,PTPN11,RHOA,IRS1,FZD6,TGFB3,LEF1,PIK3CD,PDGFD,LOX,TCF4,SNAI2,HIF1A,FZD1,NFKB1,NOTCH2,PIK3C3,HGF,SOS1,SMAD4,AKT3,MAP2K1,FGF7,EGFR,NRAS,PIK3C2A,FGF1,FZD8,NOTCH4,RRAS2,ZEB2,RBPJ,MMP9,WNT5A,FZD7
**PAK Signaling**	1,39E+01	3,86E-01	NaN	MAP2K4,RAF1,PIK3R1,WASL,SOS2,MYLK,NCK1,PDGFC,PTK2,PAK1,CFL2,PIK3CG,PIK3C3,SOS1,PDGFRA,MAP2K1,ITGA4,MYL12A,PDGFRB,ITGB1,NRAS,CASP3,PIK3C2A,FGFR1,ITGA5,FGFR2,EPHA3,MYL9,PIK3R3,RRAS2,PAK3,PTPN11,CDC42,IRS1,ARHGEF6,PAK2,PIK3CD,PDGFD,TNF
**Factors Promoting Cardiogenesis in Vertebra**	1,39E+01	4,02E-01	NaN	TCF4,TGFBR1,BMP2,TGFBR3,BMPR2,SMAD5,FZD1,TGFBR2,BMPR1A,TGFB1,SMAD4,GSK3B,SMAD1,FZD2,PRKD3,PRKD1,PRKCA,NOX4,CCNE2,PRKCQ,CDC6,ACVR1,APC,ATF2,FZD8,MAPK14,PRKCI,FZD6,TGFB3,MEF2C,PRKCH,BMP7,LEF1,CDK2,ACVR2A,FZD7,PRKCB
**Natural Killer Cell Signaling**	1,37E+01	3,52E-01	NaN	RAF1,FYN,PIK3R1,KLRB1,SOS2,NCK1,CD300A,PAK1,SH2D1A,PIK3CG,PIK3C3,SOS1,AKT3,PRKD3,MAP2K1,FCGR3A/FCGR3B,PRKD1,PRKCA,NRAS,PRKCQ,KLRC4-KLRK1/KLRK1,LAIR1,PIK3C2A,FCGR2A,TYROBP,FGFR1,FGFR2,LILRB1,PIK3R3,RRAS2,PRKCI,INPP5F,PAK3,PTPN11,PLCG2,IRS1,PAK2,ZAP70,FCER1G,PIK3CD,PRKCH,LCP2,PRKCB
**Rac Signaling**	1,37E+01	3,59E-01	NaN	MAP2K4,RAF1,PIK3R1,IQGAP1,NFKB1,CDK5R1,PTK2,PAK1,IQGAP2,ACTR3,CFL2,PIK3CG,PIK3C3,CYBB,MAP2K1,NCKAP1,ITGA4,ITGB1,ACTR2,NOX4,TIAM1,NRAS,PIK3C2A,FGFR1,MAP3K1,ITGA5,FGFR2,PLD1,PIK3R3,PIP5K1A,PRKCI,RRAS2,PAK3,PTPN11,CDC42,CYFIP1,RHOA,IRS1,NCF2,PAK2,PIK3CD,PIP4K2A
**Paxillin Signaling**	1,36E+01	3,63E-01	NaN	MAP2K4,PIK3R1,SOS2,CRK,ITGB8,NCK1,ITGB3,PTK2,PAK1,ITGA9,PIK3CG,PIK3C3,SOS1,ITGAV,VCL,ACTC1,ACTN1,ITGA4,ITGB1,PARVA,NRAS,PIK3C2A,FGFR1,ITGA5,FGFR2,ITGAL,GIT2,PIK3R3,ITGB2,RRAS2,MAPK14,ITGAM,ACTA2,PAK3,PTPN11,CDC42,IRS1,PAK2,ARHGEF6,ITGA1,PIK3CD
**Dendritic Cell Maturation**	1,34E+01	2,89E-01	NaN	MAP2K4,FCGR2C,PIK3R1,LTB,IL6,FCGR1A,CD1D,PIK3CG,PLCB1,FGFR1,CREBBP,CD1B,FGFR2,PLCL2,ATF2,PIK3R3,IL33,PTPN11,IL1RN,IRS1,PLCG2,CD86,PIK3CD,IRF8,TNF,CCR7,COL3A1,ICAM1,LEPR,COL10A1,CD83,FCGR2B,NFKB1,COL1A2,NFKBIA,DDR2,PIK3C3,CREB1,AKT3,CD1C,STAT1,COL18A1,FCGR3A/FCGR3B,FCGR1B,PIK3C2A,IL10,FCGR2A,TYROBP,STAT4,COL1A1,MAPK14,CD80,IL12B,TREM2,FCER1G
**Germ Cell-Sertoli Cell Junction Signaling**	1,34E+01	3,01E-01	NaN	MAP2K4,TGFBR1,PIK3R1,WASL,PTK2,TGFBR2,PAK1,CFL2,TGFB1,PIK3CG,MTMR2,MAP3K2,FGFR1,TUBB2A,FGFR2,RHOJ,PLS1,PIK3R3,CDH2,RHOQ,RND3,ACTA2,PTPN11,CDC42,RHOA,IRS1,TGFB3,PIK3CD,TNF,FNBP1,NECTIN3,IQGAP1,RHOH,AGGF1,SORBS1,PIK3C3,VCL,MAP2K1,ACTC1,RAB8B,ACTN1,ITGB1,NRAS,PIK3C2A,MAP3K1,TUBA1A,MAPK14,RRAS2,PAK3,PAK2,MAP3K8,A2M
**B Cell Receptor Signaling**	1,34E+01	2,92E-01	NaN	MAP2K4,RAF1,FCGR2C,PIK3R1,SOS2,BCL6,PTEN,PTPRC,PTK2,CFL2,PIK3CG,GSK3B,MAP3K2,ETS1,PRKCQ,FGFR1,CREBBP,FGFR2,CD79A,MALT1,RAP1A,ATF2,PIK3R3,PTPN11,INPP5F,CDC42,PLCG2,IRS1,PIK3CD,BCL2A1,BLNK,FCGR2B,NFKB1,NFKBIA,PIK3C3,CREB1,SOS1,AKT3,MAP2K1,PPP3CA,NRAS,PIK3C2A,FCGR2A,MAP3K1,BTK,CALM1 (includes others),MAPK14,RRAS2,FOXO1,PAG1,LYN,MAP3K8,MEF2C,PRKCB
**Renin-Angiotensin Signaling**	1,34E+01	3,50E-01	NaN	MAP2K4,PRKACB,RAF1,PIK3R1,SOS2,CCL5,NFKB1,PTK2,PAK1,CCL2,PIK3CG,PIK3C3,SOS1,STAT1,PRKD3,MAP2K1,PRKD1,PRKCA,NRAS,PRKCQ,PIK3C2A,FGFR1,MAP3K1,GNAQ,FGFR2,ITPR1,ATF2,PIK3R3,ADCY9,RRAS2,PRKAR2B,MAPK14,PRKCI,PAK3,PTPN11,PLCG2,IRS1,PAK2,PIK3CD,PRKCH,TNF,PRKCB
**Role of Pattern Recognition Receptors in Re**	1,32E+01	3,28E-01	NaN	MAP2K4,PIK3R1,C1QC,C1QA,C1QB,CCL5,IL6,NFKB1,EIF2S1,IFIH1,TGFB1,PIK3CG,PIK3C3,TLR1,CREB1,TLR7,PRKD3,PRKD1,PRKCA,PTX3,OAS1,NLRP3,PRKCQ,C5AR1,OAS2,PIK3C2A,IL10,FGFR1,FGFR2,OAS3,PIK3R3,CLEC7A,PRKCI,PTPN11,IL12B,PLCG2,IRS1,DDX58,TGFB3,PIK3CD,PRKCH,EIF2AK2,C3AR1,TNF,PRKCB
**Production of Nitric Oxide and Reactive Oxy**	1,32E+01	2,85E-01	NaN	MAP2K4,JAK1,PIK3R1,PIK3CG,CYBB,PRKD3,PRKD1,MAP3K2,PRKCQ,FGFR1,CREBBP,FGFR2,PPP1R14A,RHOJ,IFNGR1,NCF4,RAP1A,PIK3R3,RHOQ,RND3,PTPN11,PPP2R3A,RHOA,IRS1,PLCG2,NCF2,PRKCH,PIK3CD,IRF8,TNF,FNBP1,APOD,APOE,PPP1R3C,PPP1CB,NFKB1,RHOH,NFKBIA,PPP1R12A,PIK3C3,AKT3,STAT1,MAP2K1,PRKCA,PIK3C2A,MAP3K1,MAPK14,PRKCI,CAT,APOC1,MAP3K8,PPP2R5E,PPP2R1B,CLU,PRKCB
**Cardiac Hypertrophy Signaling**	1,32E+01	2,64E-01	NaN	MAP2K4,PRKACB,RAF1,TGFBR1,PIK3R1,IL6,TGFBR2,ROCK2,GNB4,TGFB1,PIK3CG,PLCB1,GNA13,GSK3B,GNG12,MAP3K2,FGFR1,GNG2,IL6R,CREBBP,FGFR2,RHOJ,PLCL2,ATF2,MYL9,PIK3R3,ADCY9,RHOQ,RND3,PTPN11,RHOA,PLCG2,IRS1,TGFB3,PIK3CD,FNBP1,RHOH,EIF4E,GNG11,IGF1,PIK3C3,SOS1,CREB1,MAP2K1,PPP3CA,MYL12A,ADRB2,NRAS,PIK3C2A,GNA12,MAP3K1,MEF2A,GNAQ,GNAI1,ROCK1,GNAI3,CALM1 (includes others),PRKAR2B,MAPK14,RRAS2,MEF2C,MAP3K8
**Virus Entry via Endocytic Pathways**	1,31E+01	3,73E-01	NaN	FYN,ITSN1,PIK3R1,ITGB8,ITGB3,FLNA,PIK3CG,PIK3C3,CAV1,PRKD3,ACTC1,PRKD1,ITGA4,PRKCA,ITGB1,NRAS,PRKCQ,PIK3C2A,FGFR1,ITGA5,FGFR2,ITGAL,PIK3R3,ITGB2,RRAS2,PRKCI,ACTA2,PTPN11,CDC42,FLNC,PLCG2,IRS1,TFRC,ITGA1,PIK3CD,PRKCH,CXADR,PRKCB
**Glucocorticoid Receptor Signaling**	1,31E+01	2,44E-01	NaN	MAP2K4,PRKACB,RAF1,JAK1,TGFBR1,SGK1,SMAD3,PIK3R1,SOS2,PBX1,IL6,HSPA5,FCGR1A,TGFBR2,TGFB1,PIK3CG,FGFR1,CREBBP,FGFR2,MED14,NCOA3,TAF9B,PIK3R3,CD3G,PTPN11,IL1RN,SMARCA2,IRS1,TGFB3,NCOA1,HSP90AA1,PIK3CD,TNF,ESR1,PBRM1,ICAM1,CCL5,NFKB1,CCL3,GTF2A1,NR3C1,BCL2,CCL13,KAT2B,NFKBIA,CCL2,PIK3C3,ANXA1,SOS1,CREB1,SMAD4,AKT3,STAT1,FKBP5,TAF2,MAP2K1,PPP3CA,ADRB2,VCAM1,NRAS,PIK3C2A,IL10,MAP3K1,CCL11,CD3D,RRAS2,MAPK14,NRIP1,SMARCC1,A2M
**FcÎ³ Receptor-mediated Phagocytosis in Mac**	1,30E+01	3,87E-01	NaN	FYN,PIK3R1,CRK,NCK1,FCGR1A,PTEN,HMOX1,PAK1,ACTR3,PIK3CG,HCK,VAMP3,AKT3,ACTC1,PRKD3,FCGR3A/FCGR3B,PRKD1,FGR,PRKCA,ACTR2,PRKCQ,PLD3,FCGR2A,FYB,PLD1,MYO5A,PLD4,PIK3R3,PIP5K1A,PRKCI,ACTA2,CDC42,LYN,PRKCH,LCP2,PRKCB
**ErbB Signaling**	1,30E+01	3,78E-01	NaN	MAP2K4,RAF1,PIK3R1,SOS2,NCK1,NRG1,PAK1,PIK3CG,ERBB4,PIK3C3,SOS1,GSK3B,PRKD3,MAP2K1,PRKD1,PRKCA,EGFR,NRAS,PRKCQ,PIK3C2A,FGFR1,FGFR2,PIK3R3,RRAS2,MAPK14,PRKCI,PAK3,PTPN11,FOXO1,CDC42,PLCG2,IRS1,PAK2,PIK3CD,PRKCH,EREG,PRKCB
**FAK Signaling**	1,28E+01	3,74E-01	NaN	RAF1,FYN,PIK3R1,HMMR,SOS2,CRK,PTEN,PTK2,PAK1,PIK3CG,PIK3C3,SOS1,AKT3,VCL,MAP2K1,ACTC1,ITGA4,EGFR,ITGB1,NRAS,PIK3C2A,ASAP1,FGFR1,ITGA5,FGFR2,GIT2,PIK3R3,RRAS2,ACTA2,PAK3,PTPN11,PLCG2,IRS1,ARHGEF6,PAK2,PIK3CD,TNS1
**CCR3 Signaling in Eosinophils**	1,28E+01	3,31E-01	NaN	RAF1,PIK3R1,PPP1CB,MYLK,ROCK2,GNB4,PAK1,GNG11,CFL2,PPP1R12A,PIK3CG,PIK3C3,PLCB1,PRKD3,MAP2K1,PRKD1,GNG12,PRKCA,NRAS,PRKCQ,PIK3C2A,FGFR1,GNG2,GNAI1,FGFR2,ITPR1,CCL11,PIK3R3,ROCK1,CALM1 (includes others),GNAI3,PLA2G4A,RRAS2,MAPK14,PRKCI,PAK3,PTPN11,RHOA,IRS1,PAK2,PIK3CD,PRKCH,PRKCB
**NF-Î°B Signaling**	1,28E+01	2,89E-01	NaN	PRKACB,RAF1,TRAF3,TGFBR1,TGFBR3,PIK3R1,TAB2,TNFRSF17,TGFBR2,PIK3CG,TLR1,GSK3B,PDGFRB,PRKCQ,FGFR1,CREBBP,FGFR2,MALT1,PIK3R3,IL33,PTPN11,IL1RN,PLCG2,IRS1,ZAP70,PIK3CD,TNF,BMP2,TNFAIP3,BMPR2,NFKB1,TANK,NFKBIA,BMPR1A,PIK3C3,TLR7,PDGFRA,AKT3,EGFR,NRAS,FLT1,PIK3C2A,MAP3K1,IL1R1,RIPK1,RRAS2,PELI1,FCER1G,MAP3K8,EIF2AK2,KDR,PRKCB
**Glioma Signaling**	1,27E+01	3,55E-01	NaN	RAF1,PIK3R1,SOS2,RBL1,CDKN2B,PDGFC,IDH1,PTEN,IGF1,PIK3CG,PIK3C3,SOS1,PDGFRA,E2F5,AKT3,PRKD3,MAP2K1,PRKD1,PRKCA,PDGFRB,EGFR,RBL2,NRAS,PRKCQ,TFDP1,PIK3C2A,FGFR1,FGFR2,PIK3R3,CALM1 (includes others),RRAS2,PRKCI,PTPN11,PLCG2,IRS1,PIK3CD,PRKCH,PDGFD,PRKCB

**fMLP Signaling in Neutrophils**	1,26E+01	3,39E-01	NaN	FPR3,RAF1,PIK3R1,NFKB1,GNB4,ACTR3,GNG11,NFKBIA,PIK3CG,PIK3C3,CYBB,PLCB1,PRKD3,MAP2K1,PRKD1,GNG12,PPP3CA,PRKCA,ACTR2,NOX4,NRAS,PRKCQ,PIK3C2A,FGFR1,GNG2,GNAI1,FGFR2,ITPR1,FPR1,PIK3R3,CALM1 (includes others),GNAI3,RRAS2,PRKCI,PTPN11,CDC42,IRS1,NCF2,PIK3CD,PRKCH,PRKCB
**Glioblastoma Multiforme Signaling**	1,26E+01	3,02E-01	NaN	RAF1,PIK3R1,SOS2,FZD1,PDGFC,RHOH,PTEN,IGF1,PIK3CG,PIK3C3,SOS1,PDGFRA,E2F5,PLCB1,AKT3,GSK3B,FZD2,MAP2K1,EGFR,PDGFRB,NRAS,PIK3C2A,FGFR1,FGFR2,RHOJ,PLCL2,ITPR1,APC,PIK3R3,FZD8,RRAS2,RHOQ,RND3,FOXO1,PTPN11,NF1,CDC42,RHOA,PLCG2,IRS1,FZD6,LEF1,PIK3CD,PDGFD,CDK2,FNBP1,WNT5A,FZD7
**Cholecystokinin/Gastrin-mediated Signaling**	1,26E+01	3,66E-01	NaN	MAP2K4,RAF1,SOS2,RHOH,PTK2,ROCK2,SOS1,PLCB1,GNA13,PRKD3,MAP2K1,PRKD1,PRKCA,EGFR,NRAS,PRKCQ,GNA12,MEF2A,GNAQ,RHOJ,ITPR1,ATF2,ROCK1,IL33,RRAS2,RHOQ,MAPK14,PRKCI,RND3,IL1RN,RHOA,CREM,MEF2C,PRKCH,TNF,FNBP1,PRKCB
**Gî±q Signaling**	1,25E+01	3,00E-01	NaN	RAF1,RGS18,PIK3R1,NFKB1,RHOH,ROCK2,HMOX1,GNB4,GNG11,NFKBIA,PIK3CG,PIK3C3,PLCB1,AKT3,GSK3B,PRKD3,MAP2K1,PRKD1,GNG12,PPP3CA,PRKCA,RGS2,PRKCQ,PLD3,PIK3C2A,FGFR1,GNG2,GNAQ,FGFR2,RHOJ,ITPR1,PLD1,PLD4,BTK,PIK3R3,ROCK1,CALM1 (includes others),PRKCI,RHOQ,RND3,PTPN11,RHOA,PLCG2,IRS1,PRKCH,PIK3CD,FNBP1,PRKCB
**Protein Kinase A Signaling**	1,24E+01	2,14E-01	NaN	PRKACB,RAF1,TGFBR1,PTPN14,SMAD3,AKAP9,PTEN,PTPRC,ROCK2,PTK2,TGFBR2,YWHAQ,GNB4,TGFB1,PLCB1,GNA13,GSK3B,PRKD3,PRKD1,GNG12,SMPDL3A,PTPRG,PRKCQ,GNG2,CREBBP,YWHAZ,PPP1R14A,PLCL2,PDE4B,ITPR1,PTP4A1,RAP1A,PTPRM,ATF2,PDE8A,MYL9,ADCY9,AKAP13,PTPN11,RHOA,PLCG2,PTPRB,CREM,TGFB3,PRKCH,LEF1,EBI3,TCF4,PPP1R3C,UBASH3B,PPP1CB,MYLK,PDE1A,NFKB1,CDKN3,NFKBIA,GNG11,PPP1R12A,FLNA,CREB1,PTPN1,SMAD4,PDE4D,MAP2K1,PPP3CA,PRKCA,MYL12A,ATF1,MAP3K1,GNAQ,GNAI1,PYGL,ROCK1,CALM1 (includes others),GNAI3,PRKCI,PRKAR2B,ADD3,FLNC,AKAP10,PDE5A,PTPN22,CDC27,PRKCB
**T Cell Receptor Signaling**	1,21E+01	3,49E-01	NaN	MAP2K4,RAF1,FYN,PIK3R1,CD4,SOS2,NFKB1,CD8A,CTLA4,PTPRC,CD28,NFKBIA,PIK3CG,PIK3C3,SOS1,MAP2K1,PPP3CA,ITK,NRAS,PRKCQ,PIK3C2A,FGFR1,MAP3K1,FGFR2,MALT1,CD3D,BTK,PIK3R3,CD3G,CALM1 (includes others),RRAS2,PTPN11,RASGRP1,IRS1,PAG1,ZAP70,PIK3CD,LCP2
**p70S6K Signaling**	1,20E+01	3,21E-01	NaN	RAF1,JAK1,F2R,PIK3R1,SOS2,YWHAQ,PIK3CG,PIK3C3,SOS1,PLCB1,AKT3,PRKD3,MAP2K1,PRKD1,PRKCA,EGFR,NRAS,PRKCQ,PIK3C2A,FGFR1,YWHAZ,GNAI1,GNAQ,FGFR2,CD79A,PLCL2,PLD1,BTK,PIK3R3,GNAI3,RRAS2,PRKCI,PTPN11,PPP2R3A,PLCG2,IRS1,LYN,PIK3CD,PRKCH,PPP2R5E,PPP2R1B,PRKCB
**Granulocyte Adhesion and Diapedesis**	1,19E+01	2,82E-01	NaN	SELL,MMP16,CXCL12,CXCL9,ITGB3,CXCL10,IL1RAP,ITGA4,CSF3R,RDX,ITGA5,THY1,FPR1,IL33,ITGAM,CCL4,SELP,CDH5,JAM3,IL1RN,PECAM1,ITGA1,TNF,FPR3,ICAM1,MMP14,CCL22,CCL5,CCL3,CCL13,CCL8,CCL2,SDC2,XCL1,MMP12,ITGB1,VCAM1,C5AR1,CXCR4,GNAI1,IL1R1,CCL11,ITGAL,SELPLG,ITGB2,GNAI3,CCL21,MMP9,CCL7,MSN
**Human Embryonic Stem Cell Pluripotency**	1,19E+01	3,08E-01	NaN	TCF4,TGFBR1,BDNF,PIK3R1,BMP2,SMAD3,BMPR2,SMAD5,FZD1,PDGFC,TGFBR2,BMPR1A,TGFB1,PIK3CG,PIK3C3,PDGFRA,SPHK1,AKT3,SMAD4,GSK3B,SMAD1,FZD2,PDGFRB,PIK3C2A,FGFR1,ACVR1,FGFR2,APC,INHBA,PIK3R3,S1PR3,FZD8,FOXO1,PTPN11,IRS1,TGFB3,S1PR1,FZD6,LEF1,BMP7,PIK3CD,PDGFD,WNT5A,FZD7
**Mouse Embryonic Stem Cell Pluripotency**	1,19E+01	3,49E-01	NaN	IL6ST,RAF1,TCF4,JAK1,PIK3R1,SOS2,BMPR2,SMAD5,FZD1,LIFR,BMPR1A,PIK3CG,PIK3C3,SOS1,SMAD4,AKT3,GSK3B,SMAD1,MAP2K1,FZD2,NRAS,PIK3C2A,FGFR1,CREBBP,FGFR2,ID3,APC,PIK3R3,FZD8,MAPK14,RRAS2,PTPN11,IRS1,FZD6,PIK3CD,LEF1,FZD7
**Role of Tissue Factor in Cancer**	1,18E+01	3,28E-01	NaN	FYN,CTGF,PIK3R1,RPS6KA3,EIF4E,PTEN,ITGB3,PAK1,ARRB1,CFL2,PIK3CG,PIK3C3,ITGAV,HCK,PLCB1,AKT3,RPS6KA2,GNA13,FGR,PRKCA,EGFR,ITGB1,NRAS,CASP3,PIK3C2A,GNA12,FGFR1,GNAQ,FGFR2,RPS6KA5,PIK3R3,RPS6KA6,RRAS2,MAPK14,PTPN11,CSF1,CDC42,IRS1,LYN,PIK3CD
**RhoGDI Signaling**	1,18E+01	2,83E-01	NaN	WASL,ROCK2,GNB4,PAK1,CFL2,GNA13,DLC1,CDH13,GNG12,ITGA4,ACTR2,GNG2,CREBBP,RDX,ITGA5,RHOJ,MYL9,ARHGAP5,CDH2,PIP5K1A,RHOQ,CDH5,ACTA2,RND3,CDC42,RHOA,ARHGEF6,PIP4K2A,ESR1,FNBP1,RHOH,CDH11,GNG11,ACTR3,PPP1R12A,ARHGEF3,ACTC1,MYL12A,PRKCA,ITGB1,ARHGEF12,GNA12,GNAQ,GNAI1,ROCK1,GNAI3,PAK3,PAK2,MSN
**G Beta Gamma Signaling**	1,16E+01	3,75E-01	NaN	PRKACB,RAF1,CAV2,SOS2,GNB4,PAK1,GNG11,PIK3CG,SOS1,CAV1,AKT3,GNA13,PRKD3,PRKD1,GNG12,PRKCA,EGFR,NRAS,PRKCQ,GNA12,GNG2,GNAI1,GNAQ,BTK,GNAI3,PRKAR2B,PRKCI,RRAS2,CDC42,PLCG2,ARHGEF6,PRKCH,PRKCB
**VEGF Signaling**	1,16E+01	3,50E-01	NaN	RAF1,PIK3R1,SOS2,HIF1A,EIF2S1,PDGFC,BCL2,ARNT,PTK2,ROCK2,PIK3CG,PIK3C3,SOS1,AKT3,VCL,MAP2K1,ACTC1,ACTN1,PRKCA,NRAS,PIK3C2A,FLT1,FGFR1,FGFR2,ROCK1,PIK3R3,RRAS2,ACTA2,PTPN11,FOXO1,PLCG2,IRS1,PIK3CD,KDR,EIF1AX,PRKCB
**ERK/MAPK Signaling**	1,16E+01	2,66E-01	NaN	PRKACB,RAF1,PIK3R1,SOS2,PTK2,YWHAQ,PAK1,PIK3CG,ITGA4,ETS1,CRKL,FGFR1,CREBBP,YWHAZ,ITGA5,FGFR2,PPP1R14A,RAP1A,ATF2,PIK3R3,PTPN11,PPP2R3A,PLCG2,IRS1,PIK3CD,ELK3,ESR1,FYN,PPP1R3C,PPP1CB,CRK,EIF4E,PPP1R12A,PIK3C3,CREB1,SOS1,STAT1,MAP2K1,PRKCA,ITGB1,NRAS,ATF1,PIK3C2A,RPS6KA5,PLA2G4A,PRKAR2B,PRKCI,RRAS2,PAK3,PAK2,PPP2R5E,PPP2R1B,PRKCB
**Gap Junction Signaling**	1,15E+01	2,82E-01	NaN	PRKACB,RAF1,PIK3R1,SOS2,SP3,PIK3CG,PIK3C3,SOS1,CAV1,PLCB1,AKT3,PRKD3,ACTC1,MAP2K1,PRKD1,PPP3CA,GUCY1B3,MAP3K2,PRKCA,EGFR,NRAS,PRKCQ,PIK3C2A,GUCY1A3,FGFR1,CSNK1G3,TUBB2A,GNAI1,GNAQ,FGFR2,PLCL2,ITPR1,PIK3R3,ADCY9,GNAI3,NOV,RRAS2,PRKCI,PRKAR2B,TUBA1A,LPAR1,ACTA2,PTPN11,PLCG2,IRS1,PRKCH,PIK3CD,PRKCB
**PDGF Signaling**	1,13E+01	3,67E-01	NaN	MAP2K4,RAF1,JAK1,PIK3R1,SOS2,CRK,PDGFC,PIK3CG,PIK3C3,SOS1,CAV1,SPHK1,PDGFRA,STAT1,MAP2K1,PRKCA,PDGFRB,NRAS,PIK3C2A,FGFR1,CRKL,MAP3K1,FGFR2,PIK3R3,RRAS2,PTPN11,INPP5F,PLCG2,IRS1,PIK3CD,EIF2AK2,PDGFD,PRKCB
**IGF-1 Signaling**	1,12E+01	3,40E-01	NaN	PRKACB,SOCS3,RAF1,JAK1,CTGF,PIK3R1,SOS2,IGFBP7,PTK2,YWHAQ,IGF1,PIK3CG,PIK3C3,SOS1,SOCS2,AKT3,MAP2K1,NEDD4,NRAS,PIK3C2A,FGFR1,YWHAZ,FGFR2,IGFBP5,GRB10,PIK3R3,NOV,PRKAR2B,PRKCI,RRAS2,PTPN11,FOXO1,IRS1,IGFBP3,PIK3CD,SOCS5
**Reelin Signaling in Neurons**	1,10E+01	3,59E-01	NaN	MAP2K4,APOE,FYN,PIK3R1,CDK5R1,ITGB3,PIK3CG,PIK3C3,HCK,MAP4K1,GSK3B,ARHGEF3,FGR,ITGA4,ITGB1,ARHGEF12,PIK3C2A,FGFR1,CRKL,ITGA5,FGFR2,VLDLR,RELN,ITGAL,APP,PIK3R3,ITGB2,PTPN11,IRS1,ARHGEF6,LYN,ITGA1,PIK3CD
**Epithelial Adherens Junction Signaling**	1,10E+01	2,95E-01	NaN	TCF4,SNAI2,TGFBR1,TGFBR3,WASL,NECTIN3,BMPR2,CRK,IQGAP1,CLIP1,PTEN,TGFBR2,NOTCH2,AGGF1,ACTR3,SORBS1,HGF,AKT3,VCL,ACTC1,ACTN1,EGFR,ACTR2,NRAS,FGFR1,SNAI1,TUBB2A,ACVR1,PTPRM,RAP1A,APC,FGF1,MYL9,NOTCH4,CDH2,RRAS2,TUBA1A,ACTA2,CDC42,RHOA,SSX2IP,LEF1,ACVR2A
**Prolactin Signaling**	1,09E+01	3,73E-01	NaN	FYN,SOCS3,RAF1,PIK3R1,SOS2,NR3C1,PIK3CG,PIK3C3,SOS1,SOCS2,STAT1,MAP2K1,PRKD3,PRKD1,PRKCA,NRAS,PRKCQ,PIK3C2A,FGFR1,CREBBP,FGFR2,PIK3R3,PRKCI,RRAS2,PTPN11,PLCG2,IRS1,PIK3CD,PRKCH,SOCS5,PRKCB
**Neuregulin Signaling**	1,09E+01	3,64E-01	NaN	RAF1,PIK3R1,SOS2,CRK,CDK5R1,PTEN,NRG1,ERBB4,SOS1,AKT3,MAP2K1,PRKD3,PRKD1,ITGA4,PRKCA,EGFR,MATK,ITGB1,NRAS,PRKCQ,DCN,CRKL,ITGA5,PIK3R3,PRKCI,RRAS2,PTPN11,PLCG2,HSP90AA1,PRKCH,EREG,PRKCB
**HER-2 Signaling in Breast Cancer**	1,09E+01	3,64E-01	NaN	PIK3R1,SOS2,ITGB8,ITGB3,NRG1,PIK3CG,PIK3C3,SOS1,AKT3,GSK3B,PRKD3,PRKD1,PRKCA,EGFR,ITGB1,CCNE2,NRAS,PRKCQ,PIK3C2A,FGFR1,FGFR2,PIK3R3,ITGB2,PRKCI,RRAS2,PTPN11,FOXO1,CDC42,IRS1,PIK3CD,PRKCH,PRKCB
**P2Y Purigenic Receptor Signaling Pathway**	1,07E+01	3,03E-01	NaN	PRKACB,RAF1,PIK3R1,NFKB1,ITGB3,GNB4,GNG11,PIK3CG,PIK3C3,CREB1,PLCB1,AKT3,PRKD3,MAP2K1,PRKD1,GNG12,PRKCA,NRAS,PRKCQ,PIK3C2A,FGFR1,GNG2,CREBBP,GNAI1,GNAQ,FGFR2,PLCL2,ATF2,PIK3R3,ADCY9,GNAI3,RRAS2,PRKAR2B,PRKCI,PTPN11,PLCG2,IRS1,PIK3CD,PRKCH,PRKCB
**Melanocyte Development and Pigmentatior**	1,06E+01	3,47E-01	NaN	PRKACB,RAF1,PIK3R1,SOS2,RPS6KA3,CRK,BCL2,PIK3CG,PIK3C3,SOS1,CREB1,KIT,RPS6KA2,MAP2K1,NRAS,PIK3C2A,MITF,FGFR1,CREBBP,FGFR2,RPS6KA5,ATF2,KITLG,TYRP1,PIK3R3,ADCY9,RPS6KA6,PRKAR2B,RRAS2,PTPN11,PLCG2,IRS1,PIK3CD
**Chemokine Signaling**	1,05E+01	3,94E-01	NaN	RAF1,CCR5,CXCL12,PPP1CB,CCL5,ROCK2,PTK2,CCL13,CCL2,PPP1R12A,PIK3CG,PLCB1,MAP2K1,PRKCA,NRAS,CXCR4,GNAQ,GNAI1,CCL11,GNAI3,CALM1 (includes others),CCL4,MAPK14,RRAS2,PLCG2,RHOA,PRKCB,CCL7
**NGF Signaling**	1,05E+01	3,16E-01	NaN	MAP2K4,RAF1,PIK3R1,SOS2,RPS6KA3,TRIO,CRK,NFKB1,ROCK2,PIK3CG,PIK3C3,SOS1,CREB1,AKT3,RPS6KA2,MAP2K1,MAP3K2,NRAS,PIK3C2A,FGFR1,MAP3K1,CREBBP,FGFR2,RPS6KA5,RAP1A,ATF2,PIK3R3,ROCK1,RPS6KA6,RRAS2,PTPN11,CDC42,RHOA,PLCG2,IRS1,PIK3CD,MAP3K8
**LPS-stimulated MAPK Signaling**	1,04E+01	3,60E-01	NaN	MAP2K4,RAF1,PIK3R1,NFKB1,PAK1,NFKBIA,PIK3CG,PIK3C3,CREB1,MAP2K1,PRKD3,PRKD1,PRKCA,NRAS,PRKCQ,ATF1,PIK3C2A,FGFR1,FGFR2,ATF2,PIK3R3,MAPK14,PRKCI,RRAS2,PTPN11,CDC42,IRS1,CD14,PIK3CD,PRKCH,PRKCB
**Th1 Pathway**	1,04E+01	2,96E-01	NaN	SOCS3,CCR5,ICAM1,JAK1,PIK3R1,CD4,IL6,NFKB1,CD8A,IL18R1,CD28,NFIL3,NOTCH2,PIK3CG,PIK3C3,CD274,IL27RA,STAT1,PRKCQ,PIK3C2A,IL10,FGFR1,IL6R,FGFR2,IFNGR1,CD3D,PIK3R3,STAT4,CD3G,ITGB2,NOTCH4,PTPN11,CD80,IL12B,IRS1,ICOS,IL10RA,CD86,PIK3CD,GATA3
**PTEN Signaling**	1,03E+01	3,11E-01	NaN	RAF1,TGFBR1,TGFBR3,PIK3R1,SOS2,BMPR2,NFKB1,BCL2,PTEN,TGFBR2,PTK2,BMPR1A,PIK3CG,SOS1,PDGFRA,AKT3,GSK3B,MAP2K1,ITGA4,PDGFRB,EGFR,ITGB1,NRAS,CASP3,FLT1,FGFR1,ITGA5,FGFR2,CNKSR3,PIK3R3,RRAS2,FOXO1,INPP5F,CDC42,PIK3CD,KDR,BCL2L11
**IL-3 Signaling**	1,02E+01	3,61E-01	NaN	RAF1,JAK1,PIK3R1,PAK1,PIK3CG,PIK3C3,SOS1,AKT3,STAT1,MAP2K1,PRKD3,PRKD1,PPP3CA,PRKCA,NRAS,PRKCQ,PIK3C2A,FGFR1,CRKL,FGFR2,PIK3R3,CSF2RB,PRKCI,RRAS2,PTPN11,FOXO1,IRS1,PIK3CD,PRKCH,PRKCB
**HMGB1 Signaling**	9,96E+00	2,93E-01	NaN	MAP2K4,ICAM1,PIK3R1,IL6,NFKB1,RHOH,KAT2B,CCL2,TGFB1,PIK3CG,PIK3C3,AKT3,MAP2K1,PLAT,VCAM1,NRAS,PIK3C2A,HAT1,FGFR1,FGFR2,RHOJ,IFNGR1,IL1R1,PIK3R3,KAT6B,RRAS2,RHOQ,MAPK14,PTPN11,RND3,CDC42,IL12B,KAT6A,RHOA,IRS1,TGFB3,PIK3CD,TNF,FNBP1
**Sphingosine-1-phosphate Signaling**	9,81E+00	3,01E-01	NaN	PIK3R1,PDGFC,RHOH,PTK2,PIK3CG,PIK3C3,PDGFRA,SPHK1,PLCB1,AKT3,GNA13,PDGFRB,CASP3,PIK3C2A,GNA12,FGFR1,GNAI1,GNAQ,FGFR2,RHOJ,PLCL2,ASAH1,PIK3R3,S1PR3,ADCY9,GNAI3,ACER3,RHOQ,PTPN11,RND3,RHOA,PLCG2,IRS1,S1PR1,PIK3CD,PDGFD,FNBP1
**Pancreatic Adenocarcinoma Signaling**	9,74E+00	3,05E-01	NaN	MAP2K4,RAF1,JAK1,TGFBR1,PIK3R1,SMAD3,NFKB1,CDKN2B,PDGFC,BCL2,TGFBR2,HMOX1,TGFB1,PIK3CG,PIK3C3,E2F5,SMAD4,AKT3,STAT1,MAP2K1,EGFR,PLD3,TFDP1,PIK3C2A,FGFR1,FGFR2,PLD1,PLD4,PIK3R3,PTPN11,CDC42,IRS1,TGFB3,PIK3CD,CDK2,MMP9

**Growth Hormone Signaling**	9,72E+00	3,58E-01	NaN	SOCS3,PIK3R1,RPS6KA3,IGF1,PIK3CG,PIK3C3,SOCS2,RPS6KA2,STAT1,PRKD3,PRKD1,PRKCA,PRKCQ,PIK3C2A,FGFR1,FGFR2,RPS6KA5,PIK3R3,RPS6KA6,PRKCI,PTPN11,PLCG2,IRS1,IGFBP3,PIK3CD,PRKCH,A2M,SOCS5,PRKCB
**Renal Cell Carcinoma Signaling**	9,72E+00	3,58E-01	NaN	RAF1,PIK3R1,SOS2,CRK,HIF1A,ARNT,PAK1,TGFB1,HGF,PIK3CG,PIK3C3,SOS1,AKT3,MAP2K1,ETS1,NRAS,PIK3C2A,FGFR1,CREBBP,FGFR2,RAP1A,PIK3R3,RRAS2,PAK3,PTPN11,CDC42,IRS1,PAK2,PIK3CD
**GÎ±12/13 Signaling**	9,68E+00	2,92E-01	NaN	MAP2K4,RAF1,LPAR4,F2R,PIK3R1,NFKB1,CDH11,PTK2,ROCK2,NFKBIA,PIK3CG,PIK3C3,AKT3,GNA13,MAP2K1,CDH13,MYL12A,NRAS,PIK3C2A,GNA12,FGFR1,MAP3K1,MEF2A,FGFR2,MYL9,BTK,ROCK1,PIK3R3,CDH2,RRAS2,CDH5,LPAR1,PTPN11,CDC42,RHOA,IRS1,MEF2C,PIK3CD
**Fc Epsilon RI Signaling**	9,64E+00	3,03E-01	NaN	MAP2K4,RAF1,FYN,PIK3R1,SOS2,FCER1A,PIK3CG,PIK3C3,SOS1,AKT3,PRKD3,MAP2K1,PRKD1,PRKCA,NRAS,PRKCQ,PIK3C2A,FGFR1,FGFR2,BTK,PIK3R3,PLA2G4A,MAPK14,PRKCI,RRAS2,INPP5F,PTPN11,PLCG2,IRS1,LYN,FCER1G,PIK3CD,PRKCH,TNF,LCP2,PRKCB
**ErbB4 Signaling**	9,60E+00	3,75E-01	NaN	RAF1,PIK3R1,SOS2,NRG1,ERBB4,PIK3C3,PIK3CG,SOS1,MAP2K1,PRKD3,PRKD1,PRKCA,NRAS,PRKCQ,YAP1,PIK3C2A,FGFR1,FGFR2,PIK3R3,PRKCI,RRAS2,PTPN11,IRS1,PLCG2,PIK3CD,PRKCH,PRKCB
**Angiopoietin Signaling**	9,59E+00	3,64E-01	NaN	ANGPT2,GRB14,PIK3R1,CRK,NCK1,NFKB1,PTK2,PAK1,NFKBIA,PIK3CG,PIK3C3,SOS1,AKT3,TEK,NRAS,PIK3C2A,ANGPT1,FGFR1,FGFR2,TIE1,PIK3R3,RRAS2,PTPN11,PAK3,FOXO1,IRS1,PAK2,PIK3CD
**CD28 Signaling in T Helper Cells**	9,59E+00	2,90E-01	NaN	MAP2K4,FYN,PIK3R1,CD4,NFKB1,CTLA4,PTPRC,CD28,PAK1,ACTR3,NFKBIA,PIK3CG,PIK3C3,AKT3,MAP2K1,PPP3CA,ITK,ACTR2,PRKCQ,PIK3C2A,FGFR1,MAP3K1,FGFR2,MALT1,ITPR1,CD3D,PIK3R3,CD3G,CALM1 (includes others),PTPN11,CD80,CDC42,IRS1,ZAP70,FCER1G,CD86,PIK3CD,LCP2
**VEGF Family Ligand-Receptor Interactions**	9,47E+00	3,41E-01	NaN	RAF1,PIK3R1,SOS2,PIK3CG,PIK3C3,SOS1,AKT3,MAP2K1,PRKD3,PRKD1,PRKCA,NRAS,PRKCQ,NRP2,PIK3C2A,FLT1,FGFR1,FGFR2,PIK3R3,PLA2G4A,PRKCI,RRAS2,PTPN11,PLCG2,IRS1,PIK3CD,PRKCH,KDR,NRP1,PRKCB
**GM-CSF Signaling**	9,46E+00	3,70E-01	NaN	RAF1,PIK3R1,SOS2,PIM1,CSF2RA,PIK3C3,PIK3CG,SOS1,HCK,AKT3,STAT1,MAP2K1,PPP3CA,ETS1,NRAS,PIK3C2A,FGFR1,FGFR2,PIK3R3,CSF2RB,RRAS2,PTPN11,IRS1,LYN,PIK3CD,BCL2A1,PRKCB
**Atherosclerosis Signaling**	9,41E+00	2,91E-01	NaN	APOE,ICAM1,MSR1,CD36,CXCL12,COL10A1,IL6,CCR2,NFKB1,PDGFC,PLA2G7,COL1A2,CCL2,TGFB1,COL18A1,ITGA4,PNPLA8,VCAM1,CXCR4,CCL11,TPSAB1/TPSB2,SELPLG,IL33,ITGB2,COL1A1,PLA2G4A,SELP,CSF1,IL1RN,APOC1,ALOX5,PDGFD,TNF,CLU,MMP9,APOD,COL3A1
**Prostate Cancer Signaling**	9,39E+00	3,30E-01	NaN	RAF1,PIK3R1,SOS2,NFKB1,BCL2,PTEN,NFKBIA,PIK3CG,PIK3C3,SOS1,CREB1,AKT3,GSK3B,MAP2K1,CCNE2,NRAS,TFDP1,PIK3C2A,FGFR1,CREBBP,FGFR2,ATF2,PIK3R3,RRAS2,PTPN11,FOXO1,IRS1,HSP90AA1,PIK3CD,LEF1,CDK2
**Role of NANOG in Mammalian Embryonic St**	9,34E+00	2,95E-01	NaN	IL6ST,RAF1,JAK1,PIK3R1,BMP2,SOS2,BMPR2,SMAD5,FZD1,LIFR,RIF1,BMPR1A,PIK3CG,PIK3C3,SOS1,SMAD4,AKT3,GSK3B,SMAD1,MAP2K1,FZD2,NRAS,PIK3C2A,FGFR1,FGFR2,APC,PIK3R3,FZD8,RRAS2,PTPN11,IRS1,FZD6,PIK3CD,BMP7,WNT5A,FZD7
**Erythropoietin Signaling**	9,32E+00	3,54E-01	NaN	SOCS3,RAF1,PIK3R1,SOS2,NFKB1,NFKBIA,PIK3CG,PIK3C3,SOS1,AKT3,MAP2K1,PRKD3,PRKD1,PRKCA,NRAS,PRKCQ,PIK3C2A,FGFR1,FGFR2,PIK3R3,PRKCI,RRAS2,PTPN11,IRS1,PLCG2,PIK3CD,PRKCH,PRKCB
**Agranulocyte Adhesion and Diapedesis**	9,31E+00	2,49E-01	NaN	AOC3,SELL,ICAM1,FN1,MMP14,MMP16,CXCL12,CCL22,CCL5,CXCL9,CCL3,CXCL10,CCL13,CCL2,CCL8,XCL1,MMP12,ACTC1,ITGA4,ITGB1,VCAM1,C5AR1,CXCR4,RDX,GNAI1,ITGA5,IL1R1,CCL11,SELPLG,MYL9,IL33,GNAI3,ITGB2,CCL4,SELP,CDH5,ACTA2,JAM3,IL1RN,CCL21,PECAM1,ITGA1,CD34,TNF,MMP9,MSN,CCL7
**IL-12 Signaling and Production in Macrophag**	9,29E+00	2,74E-01	NaN	MAP2K4,APOE,PIK3R1,MAF,NFKB1,TGFB1,PIK3CG,PIK3C3,AKT3,STAT1,PRKD3,MAP2K1,PRKD1,PRKCA,PRKCQ,PIK3C2A,IL10,FGFR1,RAB7A,FGFR2,IFNGR1,PIK3R3,STAT4,MAPK14,PRKCI,PTPN11,IL12B,IRS1,NCOA1,TGFB3,APOC1,PIK3CD,MAP3K8,PRKCH,IRF8,REL,TNF,CLU,APOD,PRKCB
**Insulin Receptor Signaling**	9,20E+00	2,77E-01	NaN	PRKACB,RAF1,SOCS3,FYN,JAK1,SGK1,PIK3R1,PPP1R3C,SOS2,PPP1CB,CRK,NCK1,EIF4E,PTEN,PPP1R12A,PIK3CG,PIK3C3,PTPN1,SOS1,AKT3,GSK3B,MAP2K1,NRAS,PIK3C2A,CRKL,FGFR1,PPP1R14A,FGFR2,GRB10,PIK3R3,RRAS2,PRKAR2B,RHOQ,PRKCI,INPP5F,PTPN11,FOXO1,IRS1,PIK3CD
**Glioma Invasiveness Signaling**	9,19E+00	3,71E-01	NaN	TIMP3,NRAS,PIK3C2A,F2R,HMMR,FGFR1,PIK3R1,FGFR2,RHOJ,RHOH,ITGB3,PTK2,PIK3R3,RRAS2,RHOQ,RND3,PTPN11,RHOA,PIK3CG,PIK3C3,IRS1,ITGAV,PIK3CD,FNBP1,MMP9,TIMP2
**Actin Nucleation by ARP-WASP Complex**	9,16E+00	4,11E-01	NaN	ITGB1,ACTR2,NRAS,GNA12,SOS2,WASL,ITGA5,RHOJ,NCK1,RHOH,ROCK1,ROCK2,WIPF1,ACTR3,RRAS2,RHOQ,RND3,PPP1R12A,CDC42,RHOA,SOS1,FNBP1,ITGA4
**14-3-3-mediated Signaling**	9,13E+00	2,85E-01	NaN	MAP2K4,RAF1,PIK3R1,YWHAQ,SRPK2,PIK3CG,PIK3C3,AKT3,PLCB1,GSK3B,PRKD3,MAP2K1,PRKD1,PRKCA,NRAS,PRKCQ,YAP1,PIK3C2A,FGFR1,TUBB2A,YWHAZ,VIM,FGFR2,PLCL2,PIK3R3,RRAS2,TUBA1A,PRKCI,PTPN11,FOXO1,PLCG2,IRS1,PIK3CD,PRKCH,SNCA,TNF,PRKCB
**Acute Myeloid Leukemia Signaling**	9,12E+00	3,30E-01	NaN	MAP2K4,RAF1,TCF4,PIK3R1,SOS2,NFKB1,IDH1,PIM1,CSF2RA,PIK3CG,PIK3C3,SOS1,AKT3,KIT,MAP2K1,CSF3R,NRAS,PIK3C2A,FGFR1,FGFR2,CSF1R,KITLG,PIK3R3,CSF2RB,RRAS2,PTPN11,IRS1,PIK3CD,LEF1,PIM2
**Nitric Oxide Signaling in the Cardiovascular S**	9,11E+00	3,01E-01	NaN	PRKACB,PIK3R1,PDE1A,PDGFC,ATP2A2,BDKRB2,PIK3CG,PIK3C3,CAV1,AKT3,PRKD3,MAP2K1,PRKD1,GUCY1B3,PRKCA,PRKCQ,GUCY1A3,PIK3C2A,FLT1,FGFR1,FGFR2,ITPR1,PIK3R3,CALM1 (includes others),PRKAR2B,PRKCI,PTPN11,IRS1,PDE5A,HSP90AA1,PIK3CD,PRKCH,KDR,PRKCB
**NRF2-mediated Oxidative Stress Response**	9,02E+00	2,44E-01	NaN	MAP2K4,RAF1,DNAJB4,PIK3R1,MAF,HSPB8,DNAJA1,CUL3,HMOX1,GSTM2,PIK3CG,PIK3C3,DNAJA2,GSK3B,FKBP5,PRKD3,MAP2K1,ACTC1,PRKD1,PRKCA,NRAS,PRKCQ,PIK3C2A,GSTA4,GSTM3,FGFR1,MAP3K1,CREBBP,FGFR2,DNAJB9,DNAJB14,TXNRD1,BACH1,PIK3R3,RRAS2,MAPK14,PRKCI,ACTA2,PTPN11,IRS1,STIP1,CAT,PIK3CD,PRKCH,AOX1,DNAJB6,PRKCB
**Endothelin-1 Signaling**	8,99E+00	2,46E-01	NaN	RAF1,PIK3R1,PLA2G7,HMOX1,PIK3CG,PIK3C3,SOS1,PLCB1,GNA13,PRKD3,PRKD1,GUCY1B3,PRKCA,PNPLA8,NRAS,PRKCQ,PLD3,CASP3,EDNRB,PIK3C2A,GUCY1A3,GNA12,FGFR1,PTGS1,MAPK6,GNAI1,GNAQ,FGFR2,PLCL2,ITPR1,PLD1,PLD4,PIK3R3,ADCY9,PLA2G4A,GNAI3,RRAS2,PRKCI,MAPK14,PTPN11,PLCG2,IRS1,EDNRA,PRKCH,PIK3CD,PRKCB
**TGF-Î² Signaling**	8,96E+00	3,33E-01	NaN	MAP2K4,RAF1,TGFBR1,SMAD3,BMP2,SOS2,BMPR2,SMAD5,BCL2,TGFBR2,RUNX2,TGFB1,BMPR1A,SOS1,SMAD4,MAP4K1,SMAD1,MAP2K1,NRAS,CREBBP,ACVR1,INHBA,MAPK14,RRAS2,CDC42,TGFB3,SMURF2,BMP7,ACVR2A
**Ovarian Cancer Signaling**	8,94E+00	2,71E-01	NaN	PRKACB,RAF1,TCF4,PIK3R1,FZD1,PDGFC,BCL2,PTEN,ARRB1,PIK3CG,PIK3C3,AKT3,GSK3B,FZD2,MAP2K1,EGFR,GJA1,NRAS,TFDP1,PIK3C2A,FGF9,FGFR1,PTGS1,FGFR2,APC,PIK3R3,FZD8,RRAS2,PRKAR2B,PTPN11,IRS1,MSH6,FZD6,EDNRA,LEF1,PIK3CD,MMP9,WNT5A,FZD7
**Chronic Myeloid Leukemia Signaling**	8,87E+00	3,08E-01	NaN	RAF1,TGFBR1,PIK3R1,SMAD3,SOS2,CRK,RBL1,NFKB1,TGFBR2,TGFB1,PIK3CG,PIK3C3,SOS1,E2F5,SMAD4,AKT3,MAP2K1,HDAC9,RBL2,NRAS,HDAC4,TFDP1,PIK3C2A,CRKL,FGFR1,FGFR2,PIK3R3,RRAS2,PTPN11,IRS1,TGFB3,PIK3CD
**GNRH Signaling**	8,65E+00	2,79E-01	NaN	MAP2K4,PRKACB,RAF1,SOS2,NFKB1,PTK2,PAK1,SOS1,CREB1,PLCB1,PRKD3,MAP2K1,PRKD1,MAP3K2,PRKCA,EGFR,NRAS,PRKCQ,MAP3K1,CREBBP,GNAI1,GNAQ,ITPR1,ATF2,ADCY9,GNAI3,PRKAR2B,MAPK14,PRKCI,RRAS2,PAK3,CDC42,PAK2,MAP3K8,PRKCH,PRKCB
**CCR5 Signaling in Macrophages**	8,61E+00	3,62E-01	NaN	MAP2K4,CCR5,PRKCQ,CD4,GNG2,GNAI1,CCL5,CCL3,CD3D,CD3G,GNB4,CALM1 (includes others),GNAI3,CCL4,PRKCI,MAPK14,GNG11,PLCG2,FCER1G,PRKCH,PRKD3,PRKD1,GNG12,PRKCA,PRKCB
**FGF Signaling**	8,58E+00	3,22E-01	NaN	RAF1,PIK3R1,SOS2,CRK,HGF,PIK3CG,PIK3C3,CREB1,SOS1,AKT3,MAP2K1,FGF7,PRKCA,PIK3C2A,FGF9,FGFR1,CRKL,MAP3K1,CREBBP,FGFR2,RPS6KA5,ITPR1,FGF1,ATF2,PIK3R3,MAPK14,PTPN11,IRS1,PIK3CD
**FLT3 Signaling in Hematopoietic Progenitor (**	8,54E+00	3,29E-01	NaN	RAF1,PIK3R1,SOS2,RPS6KA3,EIF4E,PIK3CG,PIK3C3,CREB1,SOS1,AKT3,RPS6KA2,STAT1,MAP2K1,NRAS,PIK3C2A,FGFR1,CREBBP,FGFR2,RPS6KA5,ATF2,STAT4,PIK3R3,RPS6KA6,MAPK14,RRAS2,PTPN11,IRS1,PIK3CD
**eNOS Signaling**	8,51E+00	2,58E-01	NaN	PRKACB,LPAR4,PIK3R1,HSPA5,PDGFC,BDKRB2,PIK3CG,PIK3C3,CAV1,AKT3,PRKD3,PRKD1,GUCY1B3,PRKCA,PRKCQ,CASP3,GUCY1A3,PIK3C2A,FLT1,FGFR1,GNAQ,FGFR2,AQP1,ITPR1,PIK3R3,ADCY9,CALM1 (includes others),AQP9,PRKAR2B,PRKCI,LPAR1,PTPN11,PLCG2,IRS1,HSP90AA1,PIK3CD,PRKCH,KDR,ESR1,PRKCB
**Thrombopoietin Signaling**	8,48E+00	3,69E-01	NaN	RAF1,NRAS,PRKCQ,PIK3C2A,FGFR1,PIK3R1,FGFR2,PIK3R3,PRKCI,RRAS2,PTPN11,PIK3C3,PIK3CG,PLCG2,IRS1,SOS1,PRKCH,PIK3CD,STAT1,PRKD3,MAP2K1,PRKD1,PRKCB,PRKCA
**Caveolar-mediated Endocytosis Signaling**	8,33E+00	3,52E-01	NaN	ITGB1,FYN,ITSN1,CD48,ITGA5,ITGB8,ITGAL,ITGB3,ITGB2,RAB5A,ITGAM,ACTA2,ITGA9,FLNA,FLNC,PTPN1,CAV1,ITGAV,ITGA1,PTRF,ACTC1,ITGA4,MAP3K2,PRKCA,EGFR
**UVB-Induced MAPK Signaling**	8,33E+00	3,64E-01	NaN	MAP2K4,PRKCQ,PIK3C2A,FGFR1,PIK3R1,RPS6KA3,FGFR2,RPS6KA5,EIF4E,PIK3R3,PRKCI,MAPK14,PTPN11,PIK3C3,PIK3CG,IRS1,PRKCH,PIK3CD,PRKD3,MAP2K1,PRKD1,PRKCB,PRKCA,EGFR
**IL-6 Signaling**	8,28E+00	2,76E-01	NaN	MAP2K4,IL6ST,RAF1,SOCS3,PIK3R1,SOS2,IL6,NFKB1,NFKBIA,PIK3CG,PIK3C3,SOS1,AKT3,MAP2K1,IL1RAP,ABCB1,NRAS,TNFAIP6,PIK3C2A,FGFR1,IL6R,FGFR2,IL1R1,PIK3R3,IL33,COL1A1,RRAS2,MAPK14,PTPN11,IL1RN,IRS1,CD14,PIK3CD,A2M,TNF
**RAR Activation**	8,27E+00	2,37E-01	NaN	MAP2K4,PBRM1,PRKACB,PIK3R1,BMP2,SMAD3,NR2F2,SMAD5,NFKB1,PTEN,NR2F1,KAT2B,ALDH1A1,ALDH1A3,TGFB1,PIK3CG,RARB,SMAD4,AKT3,SMAD1,PRKD3,MAP2K1,PRKD1,CITED2,PRKCA,PRKCQ,RDH11,MAP3K1,CREBBP,PIK3R3,ADCY9,CSF2RB,PRKAR2B,MAPK14,PRKCI,SMARCA2,NCOA1,TGFB3,IGFBP3,PIK3CD,PRKCH,SMARCC1,NRIP1,REL,PRKCB
**CREB Signaling in Neurons**	8,23E+00	2,39E-01	NaN	PRKACB,RAF1,PIK3R1,SOS2,GNB4,GNG11,PIK3CG,PIK3C3,SOS1,CREB1,PLCB1,AKT3,GNA13,PRKD3,MAP2K1,PRKD1,GNG12,PRKCA,NRAS,PRKCQ,PIK3C2A,FGFR1,GNA12,GNG2,CREBBP,GNAI1,GNAQ,FGFR2,PLCL2,ITPR1,ATF2,PIK3R3,ADCY9,CALM1 (includes others),GNAI3,RRAS2,PRKCI,PRKAR2B,PTPN11,PLCG2,IRS1,PRKCH,PIK3CD,PRKCB
**JAK/Stat Signaling**	8,13E+00	3,25E-01	NaN	SOCS3,RAF1,JAK1,PIK3R1,SOS2,IL6,NFKB1,PIK3CG,PIK3C3,PTPN1,SOS1,AKT3,SOCS2,STAT1,MAP2K1,NRAS,PIK3C2A,FGFR1,GNAQ,FGFR2,STAT4,PIK3R3,RRAS2,PTPN11,IRS1,PIK3CD,SOCS5
**p53 Signaling**	8,13E+00	2,88E-01	NaN	GADD45B,SNAI2,PIK3R1,PLAGL1,HIF1A,CHEK1,BCL2,PTEN,KAT2B,THBS1,STAG1,PIK3CG,PIK3C3,AKT3,GSK3B,WT1,PRKDC,HDAC9,PMAIP1,TP53INP1,PIK3C2A,TOPBP1,FGFR1,FGFR2,PIK3R3,PCNA,MAPK14,PTPN11,IRS1,PIK3CD,CDK2,DRAM1

3-phosphoinositide Biosynthesis	8,11E+00	2,31E-01	NaN	SOCS3,FYN,MINPP1,PIK3R1,TRAT1,ACP5,PTEN,SACM1L,PTPRC,MTMR6,CD28,PPP1R12A,PIK3CG,PIK3C3,ERBB4,PTPN1,PDGFRA,KIT,MTMR2,PI4K2B,PPP3CA,EGFR,PDGFRB,PPFIA1,PIK3C2A,FGFR1,PPTC7,PPP1R14A,FGFR2,PAWR,PTPRM,PIK3R3,TNS3,PIP5K1A,INPP5F,PTPN11,CD80,PPP2R3A,PPP1R16B,IRS1,ICOS,CD86,PIK3CD,PPP2R5E,PIP4K2A,PTPN22
CNTF Signaling	8,05E+00	3,65E-01	NaN	IL6ST,RAF1,NRAS,JAK1,PIK3C2A,FGFR1,PIK3R1,RPS6KA3,FGFR2,RPS6KA5,LIFR,PIK3R3,RPS6KA6,RRAS2,PTPN11,PIK3C3,PIK3CG,IRS1,SOS1,PIK3CD,RPS6KA2,STAT1,MAP2K1
ERK5 Signaling	8,05E+00	3,65E-01	NaN	IL6ST,NRAS,SGK1,GNA12,CREBBP,MEF2A,YWHAZ,GNAQ,RPS6KA3,RPS6KA5,ATF2,YWHAQ,RPS6KA6,RRAS2,PTPN11,CREB1,MAP3K8,MEF2C,RPS6KA2,GNA13,WNK1,MAP3K2,EGFR
Regulation of IL-2 Expression in Activated ar	7,97E+00	3,29E-01	NaN	MAP2K4,FYN,RAF1,TGFBR1,SMAD3,SOS2,NFKB1,TGFBR2,CD28,NFKBIA,TGFB1,SOS1,SMAD4,MAP2K1,PPP3CA,NRAS,MAP3K1,MALT1,CD3D,CALM1 (includes others),CD3G,RRAS2,CD80,PLCG2,ZAP70,TGFB3
Agrin Interactions at Neuromuscular Junctio	7,91E+00	3,48E-01	NaN	ITGB1,MAP2K4,NRAS,LAMA2,ITGA5,ITGAL,ITGB3,PTK2,LAMC1,NRG1,ITGB2,PAK1,RRAS2,PAK3,ACTA2,CDC42,ERBB4,ARHGEF6,PAK2,UTRN,ITGA1,ACTC1,ITGA4,EGFR
UVA-Induced MAPK Signaling	7,87E+00	2,94E-01	NaN	MAP2K4,PIK3R1,RPS6KA3,PARP9,TIPARP,PIK3C3,PIK3CG,PLCB1,RPS6KA2,STAT1,PRKCA,EGFR,NRAS,CASP3,PIK3C2A,FGFR1,ZC3HAV1,PARP8,FGFR2,RPS6KA5,TNKS2,PLCL2,PIK3R3,RPS6KA6,MAPK14,RRAS2,PTPN11,IRS1,PLCG2,PIK3CD
Regulation of Actin-based Motility by Rho	7,84E+00	3,08E-01	NaN	WASL,PPP1CB,MYLK,RHOH,PAK1,ACTR3,PPP1R12A,PFN2,ACTC1,ITGA4,MYL12A,ITGB1,ACTR2,ITGA5,RHOJ,ROCK1,MYL9,PIP5K1A,WIPF1,RHOQ,RND3,PAK3,ACTA2,CDC42,RHOA,PAK2,PIP4K2A,FNBP1
Clathrin-mediated Endocytosis Signaling	7,78E+00	2,28E-01	NaN	APOE,EPS15,F2R,PICALM,PIK3R1,WASL,NUMB,SH3GLB1,ITGB8,PDGFC,ITGB3,RAB5A,CD2AP,ACTR3,SNX9,ARRB1,IGF1,PIK3CG,PIK3C3,DAB2,FGF7,ACTC1,PPP3CA,ITGB1,ACTR2,PIK3C2A,FGF9,FGFR1,RAB7A,ITGA5,FGFR2,FGF1,PIK3R3,ITGB2,ACTA2,PTPN11,CDC42,IRS1,APOC1,TFRC,PIK3CD,PDGFD,MYO1E,CLU,APOD
PPARî±/RXRî± Activation	7,72E+00	2,36E-01	NaN	MAP2K4,PRKACB,RAF1,TGFBR1,CD36,TGFBR3,SMAD3,SOS2,BMPR2,IL6,NFKB1,ABCA1,NR2F1,TGFBR2,NFKBIA,TGFB1,SOS1,CLOCK,SMAD4,PLCB1,MAP2K1,IL1RAP,PRKCA,NRAS,CREBBP,ACVR1,GNAQ,PLCL2,IL1R1,NCOA3,ADCY9,PRKAR2B,MAPK14,RRAS2,GK,PLCG2,IRS1,TGFB3,HSP90AA1,MEF2C,ACVR2A,PRKCB
Adipogenesis pathway	7,66E+00	2,61E-01	NaN	BMP2,SMAD3,NR2F2,BMPR2,HIF1A,SMAD5,FZD1,EGR2,KAT2B,BMPR1A,TGFB1,EZH2,CLOCK,SMAD1,FZD2,HDAC9,ARNTL,HDAC4,HAT1,FGFR1,FGFR2,FGF1,FZD8,NR1D2,KAT6B,RUNX1T1,FOXO1,KAT6A,FZD6,BMP7,KLF3,TNF,FZD7,WNT5A,RBBP4
Ceramide Signaling	7,62E+00	3,01E-01	NaN	MAP2K4,RAF1,NRAS,PIK3C2A,FGFR1,PIK3R1,MAP3K1,FGFR2,CERK,NFKB1,BCL2,PIK3R3,S1PR3,RRAS2,PTPN11,PPP2R3A,PIK3CG,PIK3C3,IRS1,S1PR1,SPHK1,AKT3,PIK3CD,PPP2R5E,PPP2R1B,TNF,MAP2K1,NSMAF
Xenobiotic Metabolism Signaling	7,43E+00	1,99E-01	NaN	MAP2K4,RAF1,PIK3R1,MAF,IL6,CHST15,ARNT,CHST2,ALDH1A1,GSTM2,PIK3CG,CHST11,PRKD3,PRKD1,ALDH6A1,AHR,MAP3K2,PRKCQ,HDAC4,GSTM3,FGFR1,CREBBP,FGFR2,PIK3R3,HS3ST3B1,PTPN11,PPP2R3A,IRS1,NCOA1,HSP90AA1,PRKCH,PIK3CD,TNF,NFKB1,CUL3,HMOX1,ALDH1A3,PIK3C3,MAP2K1,CITED2,PRKCA,ABCB1,NRAS,PIK3C2A,GSTA4,MAP3K1,CYP1B1,MAPK14,PRKCI,RRAS2,CAT,MAP3K8,PPP2R5E,NRIP1,PPP2R1B,PRKCB,MAOA
Acute Phase Response Signaling	7,41E+00	2,37E-01	NaN	MAP2K4,IL6ST,RAF1,SOCS3,SERPING1,TCF4,FN1,PIK3R1,SOS2,IL6,NFKB1,NR3C1,C1R,HMOX1,NFKBIA,PIK3CG,SOS1,SOCS2,AKT3,OSMR,MAP2K1,IL1RAP,NRAS,C1S,MAP3K1,IL6R,SERPINF1,VWF,IL1R1,PIK3R3,IL33,RRAS2,MAPK14,RIPK1,PTPN11,IL1RN,PIK3CD,SOCS5,A2M,TNF
PEDF Signaling	7,37E+00	3,10E-01	NaN	RAF1,TCF4,BDNF,PIK3R1,NFKB1,BCL2,TCF12,ROCK2,NFKBIA,PIK3C3,PIK3CG,AKT3,NRAS,PIK3C2A,FGFR1,SERPINF1,FGFR2,ZEB1,ROCK1,PIK3R3,MAPK14,RRAS2,PTPN11,IRS1,RHOA,PIK3CD
EGF Signaling	7,35E+00	3,38E-01	NaN	MAP2K4,RAF1,JAK1,PIK3C2A,PIK3R1,FGFR1,MAP3K1,SOS2,FGFR2,ITPR1,PIK3R3,MAPK14,PTPN11,PIK3C3,PIK3CG,IRS1,SOS1,AKT3,PIK3CD,STAT1,MAP2K1,PRKCA,EGFR
Fcî³RIIB Signaling in B Lymphocytes	7,33E+00	3,77E-01	NaN	BLNK,MAP2K4,NRAS,PIK3C2A,FGFR1,PIK3R1,FGFR2,CD79A,FCGR2B,PIK3R3,BTK,RRAS2,PTPN11,PIK3C3,IRS1,PLCG2,PIK3CG,SOS1,LYN,PIK3CD
PKCÎ¸ Signaling in T Lymphocytes	7,30E+00	2,58E-01	NaN	MAP2K4,FYN,PIK3R1,CD4,SOS2,NFKB1,CD28,NFKBIA,PIK3CG,PIK3C3,SOS1,PPP3CA,MAP3K2,NRAS,PRKCQ,PIK3C2A,FGFR1,MAP3K1,FGFR2,MALT1,CD3D,PIK3R3,CD3G,RRAS2,PTPN11,CD80,PLCG2,IRS1,ZAP70,FCER1G,CD86,PIK3CD,MAP3K8,LCP2
Sertoli Cell-Sertoli Cell Junction Signaling	7,24E+00	2,30E-01	NaN	MAP2K4,PRKACB,RAF1,TGFBR3,NECTIN3,JAM2,PTEN,AGGF1,SORBS1,AKT3,MTMR2,VCL,GSK3B,ACTC1,MAP2K1,RAB8B,ACTN1,GUCY1B3,ITGA4,MAP3K2,ITGB1,DLG1,NRAS,GUCY1A3,MAP3K1,TUBB2A,ITGA5,PLS1,ATF2,EPB41,RRAS2,PRKAR2B,MAPK14,TUBA1A,ACTA2,JAM3,CDC42,TGFB3,MAP3K8,A2M,TNF
Relaxin Signaling	7,23E+00	2,43E-01	NaN	PRKACB,PIK3R1,PDE1A,NFKB1,GNB4,GNG11,NFKBIA,PIK3CG,PIK3C3,CREB1,AKT3,PDE4D,GNA13,MAP2K1,GNG12,GUCY1B3,SMPDL3A,PIK3C2A,GUCY1A3,FGFR1,GNA12,GNG2,GNAI1,GNAQ,FGFR2,PDE4B,RAP1A,PDE8A,PIK3R3,ADCY9,GNAI3,PRKAR2B,PTPN11,IRS1,PDE5A,PIK3CD,MMP9
Superpathway of Inositol Phosphate Compo	7,18E+00	2,09E-01	NaN	SOCS3,MINPP1,PIK3R1,TRAT1,ACP5,PTEN,SACM1L,PTPRC,CD28,PIK3CG,PLCB1,MTMR2,PDGFRB,FGFR1,PPTC7,FGFR2,PPP1R14A,PAWR,PTPRM,PIK3R3,PIP5K1A,PTPN11,INPP5F,PPP2R3A,IRS1,PLCG2,CD86,PIK3CD,PIP4K2A,FYN,MTMR6,PPP1R12A,PIK3C3,ERBB4,PTPN1,PDGFRA,KIT,PI4K2B,PPP3CA,EGFR,PPFIA1,PIK3C2A,PLD4,TNS3,CD80,PPP1R16B,ICOS,PPP2R5E,PTPN22
MSP-RON Signaling Pathway	7,18E+00	3,56E-01	NaN	PIK3C2A,FGFR1,PIK3R1,FGFR2,CCR2,PIK3R3,CSF2RB,ITGB2,ITGAM,CCL2,PTPN11,ACTA2,IL12B,CSF1,PIK3C3,PIK3CG,IRS1,PIK3CD,RPS6KA2,TNF,ACTC1
iCOS-iCOSL Signaling in T Helper Cells	7,10E+00	2,62E-01	NaN	PIK3R1,CD4,TRAT1,NFKB1,PTEN,PTPRC,CD28,NFKBIA,PIK3CG,PIK3C3,AKT3,PPP3CA,IL2RB,ITK,PRKCQ,PIK3C2A,FGFR1,FGFR2,ITPR1,CD3D,PIK3R3,CD3G,CALM1 (includes others),PTPN11,CD80,IRS1,ICOS,ZAP70,FCER1G,IL2RA,PIK3CD,LCP2
RhoA Signaling	7,10E+00	2,62E-01	NaN	LPAR4,PPP1CB,MYLK,ROCK2,PTK2,ACTR3,CFL2,PPP1R12A,IGF1,PFN2,GNA13,DLC1,ACTC1,MYL12A,ACTR2,NEDD4,ARHGEF12,NRP2,GNA12,RDX,CDC42EP3,PLD1,ROCK1,ARHGAP5,MYL9,PIP5K1A,ACTA2,LPAR1,RND3,RHOA,PIP4K2A,MSN
Î±-Adrenergic Signaling	7,05E+00	2,99E-01	NaN	PRKACB,RAF1,GNB4,GNG11,MAP2K1,PRKD3,GNG12,PRKD1,PRKCA,PRKCQ,NRAS,GNG2,GNAQ,GNAI1,PYGL,ITPR1,ADCY9,GNAI3,CALM1 (includes others),PRKAR2B,PRKCI,RRAS2,PLCG2,PRKCH,SLC8A1,PRKCB
CTLA4 Signaling in Cytotoxic T Lymphocytes	7,00E+00	2,83E-01	NaN	FYN,PIK3R1,TRAT1,CD8A,CTLA4,CD28,PIK3CG,PIK3C3,AKT3,PIK3C2A,AP1S2,FGFR1,FGFR2,CD3D,PIK3R3,CD3G,PTPN11,CD80,PPP2R3A,IRS1,ZAP70,FCER1G,CD86,PIK3CD,PPP2R5E,PPP2R1B,PTPN22,LCP2
Telomerase Signaling	7,00E+00	2,70E-01	NaN	RAF1,PIK3R1,SOS2,PIK3CG,PIK3C3,SOS1,AKT3,MAP2K1,TERF1,IL2RB,EGFR,ETS1,HDAC9,NRAS,HDAC4,PIK3C2A,FGFR1,FGFR2,PIK3R3,RRAS2,PTPN11,PPP2R3A,IRS1,HSP90AA1,TPP1,IL2RA,PIK3CD,PPP2R5E,ELK3,PPP2R1B
Small Cell Lung Cancer Signaling	6,75E+00	2,98E-01	NaN	TRAF3,CCNE2,TFDP1,PIK3C2A,FGFR1,PIK3R1,FGFR2,NFKB1,CDKN2B,BCL2,SKP2,PTEN,PTK2,PIK3R3,MAX,NFKBIA,PTPN11,PIK3CG,PIK3C3,IRS1,RARB,AKT3,PIK3CD,CDK2,BIRC2
T Helper Cell Differentiation	6,74E+00	3,15E-01	NaN	IL6ST,TGFBR1,IL10,IL21R,IL6R,IFNGR1,IL6,BCL6,IL18R1,TGFBR2,STAT4,CD28,CD80,IL12B,TGFB1,ICOS,FCER1G,IL10RA,CD86,IL2RA,STAT1,GATA3,TNF
STAT3 Pathway	6,74E+00	3,15E-01	NaN	MAP2K4,SOCS3,RAF1,TGFBR1,NRAS,FLT1,FGFR1,TGFBR3,BMPR2,FGFR2,BCL2,TGFBR2,MAPK14,RRAS2,PIM1,BMPR1A,PDGFRA,SOCS2,KDR,MAP2K1,SOCS5,PDGFRB,EGFR
Aldosterone Signaling in Epithelial Cells	6,68E+00	2,29E-01	NaN	RAF1,DNAJB4,SGK1,PIK3R1,SOS2,HSPB8,HSPA5,DNAJA1,PIK3CG,PIK3C3,SOS1,PLCB1,MAP2K1,PRKD3,PRKD1,PRKCA,NEDD4,PRKCQ,PIK3C2A,FGFR1,FGFR2,PLCL2,ITPR1,DNAJB9,DNAJB14,PIK3R3,PIP5K1A,PRKCI,PTPN11,PLCG2,IRS1,HSPA13,HSP90AA1,PIK3CD,PRKCH,DNAJB6,PIP4K2A,PRKCB
Inhibition of Angiogenesis by TSP1	6,63E+00	4,41E-01	NaN	MAP2K4,FYN,TGFBR1,CASP3,GUCY1A3,CD36,TGFBR2,MAPK14,TGFB1,SDC2,THBS1,AKT3,KDR,MMP9,GUCY1B3
PI3K Signaling in B Lymphocytes	6,59E+00	2,50E-01	NaN	BLNK,FYN,RAF1,PIK3R1,FCGR2B,NFKB1,PTEN,PTPRC,NFKBIA,PIK3CG,CREB1,AKT3,PLCB1,MAP2K1,PPP3CA,NRAS,ATF1,CD79A,PLCL2,MALT1,ITPR1,ATF2,BTK,CALM1 (includes others),PRKCI,RRAS2,CD180,PLCG2,IRS1,LYN,PIK3CD,PRKCB
SAPK/JNK Signaling	6,52E+00	2,69E-01	NaN	MAP2K4,PIK3R1,SOS2,CRK,GNG11,PIK3C3,PIK3CG,SOS1,MAP4K1,GNA13,MAP3K2,NRAS,PIK3C2A,GNA12,FGFR1,CRKL,GNG2,MAP3K1,FGFR2,ATF2,PIK3R3,RIPK1,RRAS2,PTPN11,CDC42,IRS1,FCER1G,PIK3CD
Dopamine-DARPP32 Feedback in cAMP Sign	6,50E+00	2,28E-01	NaN	PRKACB,PPP1R3C,PPP1CB,ATP2A2,PPP1R12A,CREB1,PLCB1,PRKD3,PRKD1,PPP3CA,GUCY1B3,PRKCA,PRKCQ,ATF1,GUCY1A3,CSNK1G3,KCNJ15,CREBBP,GNAI1,GNAQ,PPP1R14A,PAWR,PLCL2,ITPR1,ATF2,ADCY9,CALM1 (includes others),GNAI3,PRKAR2B,PRKCI,PPP2R3A,PLCG2,CREM,PRKCH,PPP2R5E,PPP2R1B,PRKCB
PI3K/AKT Signaling	6,40E+00	2,50E-01	NaN	RAF1,JAK1,PIK3R1,SOS2,NFKB1,EIF4E,BCL2,PTEN,YWHAQ,NFKBIA,PIK3CG,SOS1,AKT3,GSK3B,MAP2K1,ITGA4,ITGB1,NRAS,YWHAZ,ITGA5,PIK3R3,RRAS2,FOXO1,INPP5F,LIMS1,PPP2R3A,HSP90AA1,PIK3CD,MAP3K8,PPP2R5E,PPP2R1B
IL-15 Signaling	6,40E+00	3,03E-01	NaN	RAF1,VCAM1,NRAS,JAK1,PIK3C2A,PIK3R1,FGFR1,FGFR2,IL6,NFKB1,PTK2,PIK3R3,MAPK14,RRAS2,PTPN11,PIK3C3,PIK3CG,IRS1,AKT3,PIK3CD,TNF,MAP2K1,IL2RB
GDNF Family Ligand-Receptor Interactions	6,40E+00	3,03E-01	NaN	MAP2K4,RAF1,NRAS,PIK3C2A,PIK3R1,FGFR1,SOS2,FGFR2,GFRA2,NCK1,ITPR1,PIK3R3,RRAS2,PTPN11,CDC42,PIK3C3,PIK3CG,PLCG2,IRS1,SOS1,CREB1,PIK3CD,MAP2K1
Ephrin A Signaling	6,35E+00	3,33E-01	NaN	FYN,PIK3C2A,FGFR1,PIK3R1,FGFR2,EPHA3,ROCK1,PIK3R3,ROCK2,PTK2,PAK1,PTPN11,CFL2,CDC42,PIK3C3,RHOA,PIK3CG,IRS1,ADAM10,PIK3CD
Role of JAK1 and JAK3 in î³c Cytokine Signali	6,33E+00	3,10E-01	NaN	BLNK,SOCS3,NRAS,JAK1,PIK3C2A,FGFR1,IL21R,PIK3R1,FGFR2,TSLP,IL7,IL7R,PIK3R3,RRAS2,PTPN11,PIK3C3,PIK3CG,IRS1,PIK3CD,IL2RA,STAT1,IL2RB
Type II Diabetes Mellitus Signaling	6,16E+00	2,44E-01	NaN	MAP2K4,SOCS3,SLC27A2,CD36,PIK3R1,NFKB1,NFKBIA,PIK3C3,PIK3CG,ACSL4,AKT3,SOCS2,NSMAF,PRKD3,PRKD1,PRKCA,PRKCQ,PIK3C2A,FGFR1,MAP3K1,FGFR2,PIK3R3,PRKCI,PTPN11,IRS1,PIK3CD,PRKCH,SOCS5,TNF,ACSL1,PRKCB
Ephrin B Signaling	6,10E+00	3,01E-01	NaN	KALRN,ITSN1,CXCR4,GNA12,GNG2,GNAQ,GNAI1,CXCL12,ROCK1,ROCK2,PTK2,GNAI3,GNB4,PAK1,GNG11,CFL2,CDC42,RHOA,ITSN2,CAP1,GNA13,GNG12
Systemic Lupus Erythematosus Signaling	6,07E+00	2,00E-01	NaN	FCGR2C,PIK3R1,SOS2,IL6,FCGR2B,FCGR1A,PTPRC,BDKRB2,CD28,LSM14A,PRPF4B,PIK3CG,PIK3C3,SOS1,TLR7,PRPF40A,AKT3,FCGR3A/FCGR3B,FCGR1B,NRAS,PIK3C2A,IL10,FCGR2A,FGFR1,IL6R,FGFR2,CD79A,PRPF38B,CD3D,PIK3R3,IL33,CD3G,RRAS2,PTPN11,CD80,IL1RN,PLCG2,IRS1,CREM,LYN,FCER1G,CD86,PIK3CD,TNF,PIM2

**IL-17 Signaling**	6,06E+00	2,82E-01	NaN	MAP2K4,NRAS,JAK1,PIK3C2A,PIK3R1,FGFR1,FGFR2,IL6,CCL11,NFKB1,ATF2,PIK3R3,CXCL10,MAPK14,RRAS2,CCL2,PTPN11,PIK3C3,PIK3CG,IRS1,AKT3,PIK3CD,GSK3B,MAP2K1
**IL-10 Signaling**	6,04E+00	3,09E-01	NaN	MAP2K4,CCR1,SOCS3,FCGR2C,CCR5,JAK1,IL10,FCGR2A,IL1R1,IL6,FCGR2B,NFKB1,IL33,HMOX1,MAPK14,NFKBIA,IL1RN,IL10RA,CD14,IL1RAP,TNF
**Wnt/Î²-catenin Signaling**	6,03E+00	2,19E-01	NaN	TCF4,SFRP2,TGFBR1,FRZB,TGFBR3,BMPR2,TLE1,FZD1,SOX17,TGFBR2,TGFB1,DKK3,RARB,DKK2,AKT3,MAP4K1,GSK3B,FZD2,SFRP4,GJA1,CSNK1G3,CREBBP,ACVR1,GNAQ,APC,FZD8,CDH2,CDH5,PPP2R3A,FZD6,TGFB3,LEF1,PPP2R5E,PPP2R1B,ACVR2A,WNT5A,FZD7
**Docosahexaenoic Acid (DHA) Signaling**	6,03E+00	3,46E-01	NaN	CASP3,PIK3C2A,FGFR1,PIK3R1,SERPINF1,FGFR2,BCL2,APP,PIK3R3,FOXO1,PTPN11,PIK3C3,IRS1,PIK3CG,AKT3,PIK3CD,GSK3B,BCL2A1
**G-Protein Coupled Receptor Signaling**	5,94E+00	1,88E-01	NaN	PRKACB,RAF1,PIK3R1,SOS2,PIK3CG,RGS10,PLCB1,SMPDL3A,FGFR1,CREBBP,FGFR2,PDE4B,RAP1A,ATF2,FPR1,PIK3R3,S1PR3,PDE8A,ADCY9,PTPN11,IRS1,PIK3CD,FYN,RGS18,PTGER3,PDE1A,NFKB1,NFKBIA,PIK3C3,CREB1,SOS1,AKT3,PDE4D,MAP2K1,PRKCA,ADRB2,RGS2,NRAS,PIK3C2A,GNAQ,GNAI1,GNAI3,PRKAR2B,RRAS2,P2RY14,LPAR1,RASGRP1,S1PR1,PDE5A,MAP3K8,PRKCB
**Corticotropin Releasing Hormone Signaling**	5,92E+00	2,52E-01	NaN	PRKACB,RAF1,BDNF,CREB1,MAP2K1,PRKD3,PRKD1,GUCY1B3,PRKCA,PRKCQ,GUCY1A3,CREBBP,MEF2A,GNAI1,GNAQ,ITPR1,RAP1A,ATF2,ADCY9,CALM1 (includes others),GNAI3,PRKAR2B,MAPK14,PRKCI,PLCG2,PRKCH,MEF2C,PRKCB
**Androgen Signaling**	5,92E+00	2,52E-01	NaN	PRKACB,SMAD3,NFKB1,GTF2A1,NCOA4,GNB4,GNG11,KAT2B,GNA13,PRKD3,TAF2,PRKD1,GNG12,PRKCA,PRKCQ,GNA12,GNG2,CREBBP,GNAI1,GNAQ,GNAI3,CALM1 (includes others),PRKAR2B,PRKCI,NCOA1,HSP90AA1,PRKCH,PRKCB
**Semaphorin Signaling in Neurons**	5,90E+00	3,40E-01	NaN	ITGB1,FYN,ARHGEF12,DPYSL3,RHOJ,RHOH,ROCK1,ROCK2,PTK2,PAK1,RHOQ,RND3,CFL2,PAK3,RHOA,PAK2,FNBP1,NRP1
**Endometrial Cancer Signaling**	5,87E+00	3,12E-01	NaN	RAF1,NRAS,PIK3C2A,PIK3R1,FGFR1,SOS2,FGFR2,PTEN,PIK3R3,RRAS2,PTPN11,PIK3C3,PIK3CG,IRS1,SOS1,AKT3,PIK3CD,LEF1,GSK3B,MAP2K1
**Cell Cycle: G1/S Checkpoint Regulation**	5,87E+00	3,12E-01	NaN	HDAC9,CCNE2,RBL2,HDAC4,TFDP1,BMI1,SMAD3,CUL1,RBL1,CDKN2B,SKP2,NRG1,MAX,FOXO1,TGFB1,E2F5,TGFB3,SMAD4,GSK3B,CDK2
**IL-2 Signaling**	5,87E+00	3,12E-01	NaN	RAF1,NRAS,JAK1,PIK3C2A,PIK3R1,FGFR1,SOS2,FGFR2,PIK3R3,RRAS2,PTPN11,PIK3C3,PIK3CG,IRS1,SOS1,AKT3,PIK3CD,IL2RA,MAP2K1,IL2RB
**Myc Mediated Apoptosis Signaling**	5,83E+00	3,00E-01	NaN	MAP2K4,NRAS,CASP3,PIK3C2A,FGFR1,PIK3R1,SOS2,YWHAZ,FGFR2,BCL2,YWHAQ,PIK3R3,RRAS2,PTPN11,IGF1,PIK3C3,PIK3CG,IRS1,SOS1,AKT3,PIK3CD
**Neurotrophin/TRK Signaling**	5,80E+00	2,89E-01	NaN	MAP2K4,RAF1,NRAS,PIK3C2A,BDNF,PIK3R1,FGFR1,CREBBP,SOS2,FGFR2,ATF2,PIK3R3,RRAS2,PTPN11,CDC42,PIK3C3,PIK3CG,IRS1,SOS1,CREB1,PIK3CD,MAP2K1
**BMP signaling pathway**	5,80E+00	2,89E-01	NaN	MAP2K4,PRKACB,RAF1,NRAS,BMP2,CREBBP,BMPR2,GREM1,SMAD5,NFKB1,ATF2,MAPK14,RRAS2,PRKAR2B,RUNX2,BMPR1A,CREB1,SOS1,SMAD4,BMP7,SMAD1,MAP2K1
**Role of IL-17F in Allergic Inflammatory Airwa**	5,74E+00	3,64E-01	NaN	RAF1,CREBBP,RPS6KA3,RPS6KA5,IL6,NFKB1,ATF2,CXCL10,RPS6KA6,CCL4,CCL2,IGF1,CREB1,RPS6KA2,MAP2K1,CCL7
**RANK Signaling in Osteoclasts**	5,72E+00	2,57E-01	NaN	MAP2K4,RAF1,PIK3R1,TAB2,NFKB1,NFKBIA,PIK3C3,PIK3CG,AKT3,MAP2K1,BIRC3,PPP3CA,MAP3K2,PIK3C2A,MITF,FGFR1,MAP3K1,FGFR2,PIK3R3,CALM1 (includes others),MAPK14,PTPN11,IRS1,PIK3CD,MAP3K8,BIRC2
**Non-Small Cell Lung Cancer Signaling**	5,70E+00	2,86E-01	NaN	RAF1,NRAS,TFDP1,PIK3C2A,FGFR1,PIK3R1,SOS2,FGFR2,ITPR1,PIK3R3,RRAS2,PTPN11,PIK3C3,PIK3CG,IRS1,RARB,SOS1,AKT3,PIK3CD,MAP2K1,PRKCA,EGFR
**Crosstalk between Dendritic Cells and Natur**	5,70E+00	2,70E-01	NaN	KLRC4-KLRK1/KLRK1,TYROBP,CD69,LTB,TNFSF10,CD83,IL6,NFKB1,ITGAL,CD28,CSF2RB,PRF1,CD80,MICB,ACTA2,IL12B,ICAM3,TREM2,TLR7,CD86,TNF,ACTC1,CCR7,IL2RB
**IL-9 Signaling**	5,59E+00	3,56E-01	NaN	SOCS3,JAK1,PIK3C2A,FGFR1,PIK3R1,FGFR2,NFKB1,PIK3R3,PTPN11,PIK3C3,IRS1,PIK3CG,SOCS2,PIK3CD,STAT1,TNF
**mTOR Signaling**	5,52E+00	2,01E-01	NaN	PIK3R1,RPS6KA3,HIF1A,PDGFC,RHOH,EIF4E,HMOX1,PIK3CG,PIK3C3,AKT3,RPS6KA2,PRKD3,PRKD1,PRKCA,NRAS,PRKCQ,PLD3,PIK3C2A,FGFR1,FGFR2,RHOJ,RPS6KA5,PLD1,PLD4,PIK3R3,RPS6KA6,RRAS2,RHOQ,PRKCI,PTPN11,RND3,PPP2R3A,RHOA,IRS1,PIK3CD,PRKCH,PPP2R5E,PPP2R1B,FNBP1,PRKCB
**Pathogenesis of Multiple Sclerosis**	5,40E+00	7,78E-01	NaN	CCR1,CXCL10,CCR5,CCL4,CCL5,CCL3,CXCL9
**ErbB2-ErbB3 Signaling**	5,32E+00	2,90E-01	NaN	RAF1,NRAS,PIK3C2A,PIK3R1,FGFR1,SOS2,FGFR2,PTEN,PIK3R3,NRG1,RRAS2,FOXO1,PTPN11,PIK3C3,PIK3CG,IRS1,SOS1,PIK3CD,GSK3B,MAP2K1
**HIPPO signaling**	5,32E+00	2,64E-01	NaN	DLG1,YAP1,FAT4,SMAD3,PPP1R3C,WWTR1,CUL1,YWHAZ,PPP1CB,PPP1R14A,FRMD6,SMAD5,STK3,SKP2,ITCH,YWHAQ,PPP1R12A,PPP2R3A,SMAD4,MOB1A,PPP2R5E,PPP2R1B,SMAD1
**Synaptic Long Term Potentiation**	5,23E+00	2,33E-01	NaN	PRKACB,RAF1,PPP1R3C,PPP1CB,PPP1R12A,CREB1,PLCB1,MAP2K1,PRKD3,PRKD1,PPP3CA,PRKCA,PRKCQ,NRAS,CREBBP,GNAQ,PPP1R14A,PLCL2,ITPR1,RAP1A,ATF2,CALM1 (includes others),PRKAR2B,PRKCI,RRAS2,PLCG2,PRKCH,PRKCB
**Role of PI3K/AKT Signaling in the Pathogene**	5,21E+00	2,76E-01	NaN	CCR5,PIK3C2A,PIK3R1,FGFR1,CRKL,GNAI1,FGFR2,CRK,CCL5,NFKB1,PIK3R3,GNAI3,NFKBIA,PTPN11,PIK3C3,PIK3CG,IRS1,AKT3,PIK3CD,GSK3B,MAP2K1
**Pyridoxal 5'-phosphate Salvage Pathway**	5,13E+00	2,92E-01	NaN	MAP2K4,DAPK1,PRKCQ,SGK1,MAPK6,TTK,PAK1,CDK8,PAK3,PIM1,PRPF4B,PAK2,PRKCH,MAP3K8,EIF2AK2,HIPK1,MAP2K1,CDK2,ACVR2A
**Melatonin Signaling**	5,12E+00	2,82E-01	NaN	MAP2K4,PRKACB,RAF1,PRKCQ,GNAQ,GNAI1,PLCL2,CALM1 (includes others),GNAI3,PRKCI,PRKAR2B,RORA,PLCG2,PLCB1,PRKCH,PRKD3,MAP2K1,PRKD1,PRKCB,PRKCA
**Estrogen-Dependent Breast Cancer Signaling**	5,12E+00	2,73E-01	NaN	NRAS,PIK3C2A,FGFR1,PIK3R1,CREBBP,HSD17B11,FGFR2,NFKB1,ATF2,PIK3R3,RRAS2,PTPN11,IGF1,PIK3C3,PIK3CG,IRS1,CREB1,AKT3,PIK3CD,ESR1,EGFR
**IL-17A Signaling in Airway Cells**	5,12E+00	2,73E-01	NaN	MAP2K4,JAK1,PIK3C2A,PIK3R1,FGFR1,FGFR2,IL6,NFKB1,CCL11,PTEN,PIK3R3,NFKBIA,MAPK14,PTPN11,PIK3C3,PIK3CG,IRS1,AKT3,PIK3CD,GSK3B,MAP2K1
**HIF1Î± Signaling**	5,12E+00	2,35E-01	NaN	MMP14,MMP16,PIK3R1,HIF1A,PDGFC,LDHB,ARNT,PIK3C3,PIK3CG,AKT3,MMP12,SLC2A5,NRAS,PIK3C2A,FGFR1,CREBBP,MAPK6,FGFR2,PIK3R3,MAPK14,RRAS2,PTPN11,IRS1,NCOA1,HSP90AA1,PIK3CD,MMP9
**TNFR1 Signaling**	5,06E+00	3,27E-01	NaN	MAP2K4,CASP3,MAP3K1,TNFAIP3,NFKB1,TANK,PAK1,RIPK1,NFKBIA,CRADD,PAK3,CDC42,PAK2,TNF,BIRC3,BIRC2
**CD40 Signaling**	5,03E+00	2,69E-01	NaN	MAP2K4,TRAF3,ICAM1,PIK3C2A,ATF1,FGFR1,PIK3R1,PTGS1,TNFAIP3,FGFR2,NFKB1,TANK,PIK3R3,NFKBIA,MAPK14,PTPN11,PIK3C3,PIK3CG,IRS1,PIK3CD,MAP2K1
**Melanoma Signaling**	5,00E+00	3,09E-01	NaN	RAF1,NRAS,PIK3C2A,MITF,FGFR1,PIK3R1,FGFR2,PTEN,PIK3R3,RRAS2,PTPN11,PIK3C3,PIK3CG,IRS1,AKT3,PIK3CD,MAP2K1
**Lymphotoxin Î² Receptor Signaling**	4,94E+00	2,84E-01	NaN	TRAF3,VCAM1,CASP3,PIK3C2A,FGFR1,PIK3R1,CREBBP,LTB,FGFR2,NFKB1,PIK3R3,NFKBIA,PTPN11,PIK3C3,PIK3CG,IRS1,AKT3,PIK3CD,BIRC2
**Role of IL-17A in Arthritis**	4,94E+00	2,84E-01	NaN	MAP2K4,PIK3C2A,FGFR1,PIK3R1,FGFR2,CCL5,NFKB1,ATF2,PIK3R3,NFKBIA,MAPK14,CCL2,PTPN11,PIK3C3,PIK3CG,IRS1,PIK3CD,MAP2K1,CCL7
**Huntington's Disease Signaling**	4,91E+00	1,83E-01	NaN	MAP2K4,SGK1,BDNF,PIK3R1,SOS2,HSPA5,CDK5R1,TGM2,GNB4,GNG11,IGF1,PIK3CG,PIK3C3,SOS1,CREB1,VAMP3,PLCB1,AKT3,PRKD3,PRKD1,GNG12,PRKCA,EGFR,HDAC9,PRKCQ,HDAC4,CASP3,PIK3C2A,FGFR1,GNG2,CREBBP,GNAQ,FGFR2,ITPR1,TAF9B,ATF2,PIK3R3,PRKCI,PTPN11,IRS1,PIK3CD,PRKCH,SNCA,PRKCB
**Cdc42 Signaling**	4,89E+00	2,04E-01	NaN	MAP2K4,RAF1,WASL,PPP1CB,MYLK,IQGAP1,CLIP1,IQGAP2,PAK1,ACTR3,CFL2,PPP1R12A,EXOC5,GSK3B,ITGA4,MYL12A,ITK,ITGB1,ACTR2,ITGA5,CD3D,APC,ATF2,MYL9,CD3G,WIPF1,MAPK14,PRKCI,PAK3,CDC42,ARHGEF6,PAK2,FCER1G,FNBP1L
**PPAR Signaling**	4,81E+00	2,47E-01	NaN	RAF1,NRAS,CREBBP,SOS2,IL1R1,NFKB1,PDGFC,IL33,NR2F1,RRAS2,NFKBIA,IL1RN,SOS1,NCOA1,PDGFRA,HSP90AA1,NRIP1,PDGFD,IL1RAP,TNF,MAP2K1,CITED2,PDGFRB
**Antiproliferative Role of TOB in T Cell Signal**	4,78E+00	4,23E-01	NaN	TGFBR2,CCNE2,TGFBR1,TGFB1,SMAD3,CUL1,TGFB3,SMAD4,TWSG1,CDK2,SKP2
**Wnt/Ca+ pathway**	4,78E+00	2,98E-01	NaN	CREBBP,PLCL2,FZD1,NFKB1,ROR1,ATF2,FZD8,PLCG2,CREB1,FZD6,PLCB1,GSK3B,FZD2,PPP3CA,WNT5A,FZD7,PRKCA
**TREM1 Signaling**	4,75E+00	2,67E-01	NaN	ITGB1,TREM1,ICAM1,NLRP3,IL10,TYROBP,ITGA5,CD83,IL6,CCL3,NFKB1,FCGR2B,CCL2,PLCG2,TLR1,TLR7,CD86,AKT3,TNF,CCL7
**UVC-Induced MAPK Signaling**	4,59E+00	3,33E-01	NaN	MAP2K4,RAF1,PRKCQ,NRAS,PRKCI,MAPK14,RRAS2,PRKCH,PRKD3,MAP2K1,PRKD1,EGFR,PRKCA,PRKCB
**Complement System**	4,56E+00	3,51E-01	NaN	SERPING1,CD59,C5AR1,C1S,C1QA,C1QC,C1QB,C1R,ITGB2,ITGAM,CFI,CFH,C3AR1
**Altered T Cell and B Cell Signaling in Rheuma**	4,54E+00	2,44E-01	NaN	TRAF3,SPP1,SLAMF1,IL10,LTB,CD79A,IL6,NFKB1,TNFRSF17,IL33,CD28,CD80,IL12B,CSF1,TGFB1,IL1RN,TLR1,TLR7,FCER1G,CCL21,CD86,TNF
**p38 MAPK Signaling**	4,51E+00	2,22E-01	NaN	MAP2K4,TGFBR1,TAB2,RPS6KA3,TGFBR2,TGFB1,CREB1,MAP4K1,RPS6KA2,STAT1,IL1RAP,ATF1,CREBBP,MEF2A,RPS6KA5,IL1R1,ATF2,IL33,PLA2G4A,RPS6KA6,MAX,MAPK14,IL1RN,TGFB3,MEF2C,TNF
**VDR/RXR Activation**	4,48E+00	2,56E-01	NaN	WT1,SPP1,PRKCQ,CCNC,IGFBP5,CCL5,THBD,NCOA3,CXCL10,PRKCI,FOXO1,RUNX2,IGFBP3,NCOA1,CD14,PRKCH,PRKD3,PRKD1,PRKCB,PRKCA
**Synaptic Long Term Depression**	4,45E+00	2,05E-01	NaN	RAF1,PLA2G7,IGF1,PLCB1,GNA13,MAP2K1,PRKD3,PRKD1,GUCY1B3,PRKCA,PNPLA8,NRAS,PRKCQ,GUCY1A3,GNA12,GNAQ,GNAI1,PLCL2,ITPR1,GNAI3,PLA2G4A,PRKCI,RRAS2,PPP2R3A,PLCG2,LYN,PRKCH,PPP2R5E,PPP2R1B,PRKCB
**Leptin Signaling in Obesity**	4,43E+00	2,47E-01	NaN	PRKACB,SOCS3,PIK3C2A,LEPR,FGFR1,PIK3R1,FGFR2,PLCL2,PIK3R3,ADCY9,PRKAR2B,FOXO1,PTPN11,PIK3C3,PIK3CG,PLCG2,IRS1,PLCB1,AKT3,PIK3CD,MAP2K1
**Aryl Hydrocarbon Receptor Signaling**	4,39E+00	2,07E-01	NaN	IL6,RBL1,NFKB1,CHEK1,ARNT,TGM2,NR2F1,GSTM2,ALDH1A1,ALDH1A3,TGFB1,RARB,ALDH6A1,AHR,RBL2,CCNE2,TFDP1,GSTM3,GSTA4,NCOA3,CYP1B1,TGFB3,HSP90AA1,DHFR,NRIP1,TNF,ESR1,CDK2,MCM7
**Inhibition of Matrix Metalloproteases**	4,29E+00	3,33E-01	NaN	TIMP3,MMP14,RECK,MMP16,TFPI2,ADAM12,SDC2,THBS2,ADAM10,MMP12,A2M,MMP9,TIMP2
**Neuropathic Pain Signaling In Dorsal Horn N**	4,26E+00	2,19E-01	NaN	PRKACB,BDNF,PIK3R1,PIK3C3,PIK3CG,CREB1,PLCB1,PRKD3,PRKD1,PRKCA,PRKCQ,PIK3C2A,FGFR1,FGFR2,PLCL2,ITPR1,PIK3R3,PRKAR2B,PRKCI,PTPN11,IRS1,PLCG2,PIK3CD,PRKCH,PRKCB

**Antiproliferative Role of Somatostatin Recep**	4,20E+00	2,53E-01	NaN	NRAS,PIK3C2A,GUCY1A3,FGFR1,PIK3R1,GNG2,FGFR2,PIK3R3,GNB4,RRAS2,GNG11,PTPN11,PIK3C3,PIK3CG,IRS1,PIK3CD,MAP2K1,GNG12,GUCY1B3
**Role of JAK family kinases in IL-6-type Cytok**	4,13E+00	4,00E-01	NaN	IL6ST,MAP2K4,SOCS3,JAK1,MAPK14,PTPN11,IL6R,OSMR,IL6,STAT1
**Communication between Innate and Adapti**	4,12E+00	2,36E-01	NaN	IL10,CD4,CD83,CCL5,IL6,CCL3,CD8A,TNFRSF17,IL33,CXCL10,CD28,CCL4,CD80,IL12B,IL1RN,TLR1,TLR7,FCER1G,CD86,TNF,CCR7
**Sumoylation Pathway**	4,09E+00	2,29E-01	NaN	ETS1,MAP2K4,SERBP1,CREBBP,RHOJ,ZEB1,RFC5,NFKB1,NR3C1,RHOH,SP100,TDG,PCNA,SP3,RHOQ,NFKBIA,RND3,RHOA,SMAD4,SNCA,FNBP1,RFC3
**Type I Diabetes Mellitus Signaling**	4,07E+00	2,18E-01	NaN	MAP2K4,SOCS3,JAK1,CASP3,IFNGR1,IL1R1,NFKB1,CD3D,BCL2,CD3G,CD28,PRF1,RIPK1,MAPK14,NFKBIA,CD80,IL12B,FCER1G,CD86,SOCS2,STAT1,IL1RAP,TNF,SOCS5
**Role of MAPK Signaling in the Pathogenesis**	3,92E+00	2,50E-01	NaN	PNPLA8,MAP2K4,RAF1,NRAS,CASP3,CCL5,PLA2G7,BCL2,ATF2,CXCL10,PLA2G4A,MAPK14,RRAS2,CCL2,AKT3,TNF,MAP2K1,PRKCA
**GPCR-Mediated Nutrient Sensing in Enteroe**	3,92E+00	2,35E-01	NaN	PRKACB,PRKCQ,GNG2,GNAI1,GNAQ,PLCL2,ITPR1,ADCY9,GNAI3,PRKCI,GNG11,PRKAR2B,PLCG2,PLCB1,PRKCH,PRKD3,PRKD1,GNG12,PRKCB,PRKCA
**Death Receptor Signaling**	3,90E+00	2,28E-01	NaN	GAS2,MAP2K4,CASP3,ZC3HAV1,TNFSF10,PARP8,TNKS2,NFKB1,PARP9,BCL2,ROCK1,TANK,RIPK1,NFKBIA,ACTA2,CRADD,TIPARP,TNF,ACTC1,BIRC3,BIRC2
**ATM Signaling**	3,80E+00	2,38E-01	NaN	MAP2K4,SMC3,GADD45B,ATF1,TOPBP1,CREBBP,CCNB2,ZEB1,CHEK1,NBN,ATF2,SMC1A,NFKBIA,MAPK14,SMC2,CREB1,TLK1,TLK2,CDK2
**Cancer Drug Resistance By Drug Efflux**	3,78E+00	2,86E-01	NaN	PIK3R3,RAF1,ABCB1,NRAS,RRAS2,FOXO1,PIK3CG,ABCG2,PIK3R1,AKT3,PIK3CD,NFKB1,MAP2K1,PTEN
**Differential Regulation of Cytokine Producti**	3,73E+00	4,44E-01	NaN	CCL4,CCL2,IL12B,IL10,IL6,CCL5,CCL3,TNF
**Salvage Pathways of Pyrimidine Ribonucleot**	3,69E+00	2,21E-01	NaN	MAP2K4,APOBEC3B,DAPK1,PRKCQ,SGK1,MAPK6,TTK,APOBEC3G,PAK1,CDK8,PAK3,PIM1,PRPF4B,PAK2,PRKCH,MAP3K8,EIF2AK2,HIPK1,MAP2K1,CDK2,ACVR2A
**IL-4 Signaling**	3,64E+00	2,25E-01	NaN	IRF4,NRAS,JAK1,PIK3C2A,IL13RA1,FGFR1,PIK3R1,SOS2,FGFR2,NR3C1,PIK3R3,RRAS2,PTPN11,INPP5F,PIK3C3,PIK3CG,IRS1,SOS1,AKT3,PIK3CD
**PCP pathway**	3,62E+00	2,54E-01	NaN	MAP2K4,DAAM1,CTHRC1,FZD1,ATF2,ROCK1,ROCK2,FZD8,SDC2,RHOA,FZD6,PFN2,PRICKLE1,FZD2,WNT5A,FZD7
**Oncostatin M Signaling**	3,56E+00	3,24E-01	NaN	IL6ST,TIMP3,RAF1,EPAS1,JAK1,RRAS2,NRAS,SOS1,OSMR,STAT1,MAP2K1
**Role of PKR in Interferon Induction and Anti**	3,51E+00	3,00E-01	NaN	TRAF3,NFKBIA,MAPK14,CASP3,TAB2,EIF2AK2,STAT1,NFKB1,EIF2S1,TNF,FCGR1A,ATF2
**IL-1 Signaling**	3,50E+00	2,20E-01	NaN	MAP2K4,PRKACB,GNA12,GNG2,MAP3K1,TAB2,GNAQ,GNAI1,IL1R1,NFKB1,ADCY9,GNB4,GNAI3,MAPK14,GNG11,PRKAR2B,NFKBIA,GNA13,IL1RAP,GNG12
**Hereditary Breast Cancer Signaling**	3,47E+00	1,90E-01	NaN	PBRM1,GADD45B,PIK3R1,CHEK1,PTEN,PIK3C3,PIK3CG,AKT3,HDAC9,NRAS,HDAC4,PIK3C2A,FGFR1,WEE1,CREBBP,FGFR2,RFC5,NBN,PIK3R3,RRAS2,PTPN11,SMARCA2,IRS1,MSH6,PIK3CD,SMARCC1,RFC3
**GÎ±i Signaling**	3,47E+00	2,00E-01	NaN	PRKACB,RAF1,NRAS,PTGER3,GNG2,RALB,SOS2,GNAI1,RAP1A,FPR1,S1PR3,ADCY9,GNAI3,GNB4,RRAS2,GNG11,PRKAR2B,P2RY14,LPAR1,RGS10,SOS1,S1PR1,CAV1,GNG12
**Cyclins and Cell Cycle Regulation**	3,47E+00	2,31E-01	NaN	HDAC9,RAF1,CCNE2,HDAC4,TFDP1,WEE1,CUL1,CCNB2,CDKN2B,SKP2,TGFB1,PPP2R3A,E2F5,TGFB3,GSK3B,PPP2R5E,PPP2R1B,CDK2
**Estrogen Receptor Signaling**	3,43E+00	1,95E-01	NaN	RAF1,PRKDC,NRAS,CCNC,SOS2,CREBBP,RBFOX2,GTF2A1,NR3C1,NCOA3,MED14,TAF9B,DDX5,CDK8,RRAS2,KAT2B,RUNX2,SOS1,MED21,NCOA1,MED13L,NRIP1,TAF2,MAP2K1,ESR1
**Mechanisms of Viral Exit from Host Cells**	3,40E+00	2,93E-01	NaN	NEDD4,PRKCI,PRKCQ,ACTA2,SH3GLB1,XPO1,PRKCH,ACTC1,PRKD3,PRKD1,PRKCB,PRKCA
**Calcium-induced T Lymphocyte Apoptosis**	3,38E+00	2,42E-01	NaN	PRKCQ,CD4,ITPR1,ATP2A2,CD3D,CALM1 (includes others),CD3G,PRKCI,ZAP70,FCER1G,PRKCH,PRKD3,PRKD1,PPP3CA,PRKCA,PRKCB
**Mitotic Roles of Polo-Like Kinase**	3,38E+00	2,42E-01	NaN	KIF23,SMC3,CDC20,WEE1,CCNB2,SMC1A,PLK4,TGFB1,PPP2R3A,FBXO5,HSP90AA1,PPP2R5E,PPP2R1B,CDC27,RAD21,STAG2
**Role of Hypercytokinemia/hyperchemokine**	3,20E+00	2,79E-01	NaN	IL33,CCR1,CXCL10,CCR5,CCL4,CCL2,IL12B,IL1RN,IL6,CCL5,CCL3,TNF
**Apoptosis Signaling**	3,19E+00	2,13E-01	NaN	GAS2,MAP2K4,RAF1,PRKCQ,NRAS,CASP3,NFKB1,BCL2,ROCK1,RRAS2,NFKBIA,PLCG2,BCL2A1,TNF,MAP2K1,BIRC3,BCL2L11,BIRC2,PRKCA
**Activation of IRF by Cytosolic Pattern Recog**	3,18E+00	2,42E-01	NaN	MAP2K4,TRAF3,IL10,CREBBP,IL6,NFKB1,ATF2,TANK,IFIH1,RIPK1,NFKBIA,DDX58,IFIT2,STAT1,TNF
**Amyotrophic Lateral Sclerosis Signaling**	3,16E+00	1,98E-01	NaN	CASP3,PIK3C2A,FGFR1,PIK3R1,FGFR2,PDGFC,BCL2,PIK3R3,PAK1,RAB5A,PTPN11,IGF1,PIK3C3,PIK3CG,IRS1,CAT,AKT3,PIK3CD,BIRC3,RNF19A,PPP3CA,BIRC2
**Regulation of eIF4 and p70S6K Signaling**	3,13E+00	1,78E-01	NaN	RAF1,PIK3R1,SOS2,EIF2S1,EIF4E,PIK3C3,PIK3CG,PAIP1,SOS1,AKT3,MAP2K1,ITGA4,ITGB1,NRAS,PIK3C2A,FGFR1,ITGA5,FGFR2,PIK3R3,MAPK14,RRAS2,PTPN11,PPP2R3A,IRS1,PIK3CD,PPP2R5E,PPP2R1B,EIF1AX
**AMPK Signaling**	3,11E+00	1,69E-01	NaN	PBRM1,PRKACB,CAB39,PIK3R1,KAT2B,TBC1D1,PIK3CG,PIK3C3,CREB1,AKT3,PPM1A,ADRB2,PIK3C2A,FGFR1,CREBBP,FGFR2,ATF2,PIK3R3,MAPK14,PRKAR2B,FOXO1,PTPN11,SMARCA2,PPP2R3A,IRS1,PIK3CD,PPP2R5E,SMARCC1,HMGCR,AK2,PPP2R1B,PPAT
**Tight Junction Signaling**	3,03E+00	1,74E-01	NaN	PRKACB,TGFBR1,NECTIN3,JAM2,MYLK,NFKB1,PTEN,TGFBR2,MPDZ,TGFB1,VAMP3,AKT3,VCL,ACTC1,TIAM1,CNKSR3,MYL9,EPB41,PRKAR2B,PRKCI,ACTA2,JAM3,PPP2R3A,CDC42,RHOA,TGFB3,PPP2R5E,TNF,PPP2R1B
**CDK5 Signaling**	3,02E+00	2,02E-01	NaN	ITGB1,PRKACB,RAF1,NRAS,BDNF,PPP1R3C,MAPK6,PPP1CB,PPP1R14A,CDK5R1,LAMC1,ADCY9,MAPK14,RRAS2,PRKAR2B,PPP1R12A,PPP2R3A,PPP2R5E,PPP2R1B,MAP2K1
**Cardiac Î²-adrenergic Signaling**	2,98E+00	1,82E-01	NaN	PRKACB,PPP1R3C,AKAP9,PPP1CB,PDE1A,ATP2A2,GNB4,GNG11,PPP1R12A,PDE4D,GNG12,SMPDL3A,GNG2,PPP1R14A,PDE4B,PDE8A,AKAP13,ADCY9,PRKAR2B,PPP2R3A,AKAP10,PDE5A,PPP2R5E,SLC8A1,PPP2R1B
**TNFR2 Signaling**	2,83E+00	3,10E-01	NaN	MAP2K4,TANK,NFKBIA,MAP3K1,TNFAIP3,NFKB1,BIRC3,TNF,BIRC2
**Sperm Motility**	2,82E+00	1,84E-01	NaN	PNPLA8,PRKACB,PRKCQ,GUCY1A3,PLCL2,ITPR1,PDE4B,PDE1A,PLA2G7,PTK2,PLA2G4A,CALM1 (includes others),PRKCI,PRKAR2B,PLCG2,PLCB1,PRKCH,PDE4D,PRKD3,PRKD1,GUCY1B3,PRKCB,PRKCA
**Toll-like Receptor Signaling**	2,80E+00	2,16E-01	NaN	MAP2K4,MAP3K1,TAB2,TNFAIP3,NFKB1,IL33,LY96,MAPK14,NFKBIA,IL12B,IL1RN,TLR1,TLR7,CD14,EIF2AK2,TNF
**Antioxidant Action of Vitamin C**	2,80E+00	1,94E-01	NaN	PNPLA8,MAP2K4,SLC2A5,PLD3,PLCL2,NFKB1,PLA2G7,TXNRD1,PLD1,PLD4,PLA2G4A,HMOX1,CSF2RB,SLC23A2,MAPK14,NFKBIA,CSF2RA,PLCG2,PLCB1,TNF
**Role of CHK Proteins in Cell Cycle Checkpoin**	2,71E+00	2,36E-01	NaN	RFC5,CHEK1,RAD1,NBN,PCNA,PPP2R3A,E2F5,TLK1,PPP2R5E,TLK2,PPP2R1B,CDK2,RFC3
**Hepatic Cholestasis**	2,71E+00	1,70E-01	NaN	MAP2K4,PRKACB,IL6,NFKB1,NFKBIA,TGFB1,IL1RAP,PRKD3,PRKD1,PRKCA,ABCB1,PRKCQ,IL1R1,IL33,ADCY9,LY96,PRKAR2B,PRKCI,SLCO3A1,IL1RN,IL12B,TGFB3,CD14,PRKCH,TNF,ESR1,PRKCB
**Remodeling of Epithelial Adherens Junction**	2,68E+00	2,17E-01	NaN	ACTR2,TUBB2A,RAB7A,IQGAP1,CLIP1,MAPRE2,APC,RAB5A,ACTR3,TUBA1A,ACTA2,HGF,VCL,ACTC1,ACTN1
**D-myo-inositol-5-phosphate Metabolism**	2,62E+00	1,68E-01	NaN	SOCS3,MINPP1,ACP5,PTEN,SACM1L,PTPRC,MTMR6,PPP1R12A,PTPN1,PLCB1,MTMR2,PPP3CA,PPFIA1,PPTC7,PPP1R14A,PAWR,PTPRM,PLD4,TNS3,PTPN11,INPP5F,PPP1R16B,PPP2R3A,PLCG2,PPP2R5E,PIP4K2A,PTPN22
**Cardiomyocyte Differentiation via BMP Rece**	2,59E+00	3,50E-01	NaN	BMPR1A,BMP2,SMAD4,BMPR2,BMP7,MEF2C,ATF2
**Cell Cycle Control of Chromosomal Replicati**	2,51E+00	2,63E-01	NaN	PCNA,PRIM1,MCM2,CDC6,TOP2A,PRIM2,DBF4,MCM4,CDK2,MCM7
**Bladder Cancer Signaling**	2,46E+00	1,95E-01	NaN	RAF1,DAPK1,NRAS,TFDP1,MMP14,MMP16,FGF9,RPS6KA5,PDGFC,FGF1,RRAS2,THBS1,MMP12,FGF7,MAP2K1,MMP9,EGFR
**Role of JAK2 in Hormone-like Cytokine Signa**	2,31E+00	2,65E-01	NaN	SOCS3,JAK1,PTPN11,IRS1,SH2B3,PTPN1,SOCS2,STAT1,SOCS5
**Hematopoiesis from Pluripotent Stem Cells**	2,30E+00	2,34E-01	NaN	KITLG,CD3G,IL12B,IL10,CSF1,CD4,FCER1G,IL6,CD8A,IL7,CD3D
**LXR/RXR Activation**	2,30E+00	1,74E-01	NaN	APOE,MSR1,CD36,SERPINF1,IL1R1,IL6,NFKB1,ABCA1,IL33,LY96,CCL2,IL1RN,APOC1,CD14,HMGCR,IL1RAP,TNF,CLU,MMP9,CCL7,APOD
**TR/RXR Activation**	2,28E+00	1,84E-01	NaN	UCP2,PIK3C2A,FGFR1,PIK3R1,FGFR2,HIF1A,NCOA3,NCOA4,PIK3R3,PTPN11,COL6A3,PIK3C3,PIK3CG,IRS1,NCOA1,AKT3,PIK3CD,RCAN2
**Coagulation System**	2,22E+00	2,57E-01	NaN	BDKRB2,F2R,PROS1,F13A1,VWF,THBD,TFPI,A2M,PLAT
**cAMP-mediated signaling**	2,21E+00	1,48E-01	NaN	PRKACB,RAF1,RGS18,PTGER3,AKAP9,PDE1A,RGS10,CREB1,PDE4D,MAP2K1,PPP3CA,ADRB2,SMPDL3A,RGS2,CREBBP,GNAI1,PDE4B,RAP1A,ATF2,FPR1,PDE8A,S1PR3,ADCY9,CALM1 (includes others),AKAP13,GNAI3,P2RY14,PRKAR2B,LPAR1,CREM,AKAP10,S1PR1,PDE5A
**Differential Regulation of Cytokine Producti**	2,21E+00	3,04E-01	NaN	CCL4,CCL2,IL12B,IL10,CCL5,CCL3,TNF
**Intrinsic Prothrombin Activation Pathway**	2,20E+00	2,76E-01	NaN	COL1A2,COL1A1,PROS1,F13A1,COL10A1,COL18A1,THBD,COL3A1
**Interferon Signaling**	2,14E+00	2,50E-01	NaN	IFIT3,IFIT1,OAS1,JAK1,IFNGR1,IFNAR2,STAT1,MED14,BCL2
**Role of p14/p19ARF in Tumor Suppression**	2,10E+00	2,33E-01	NaN	PIK3R3,PTPN11,PIK3C2A,PIK3CG,PIK3C3,PIK3R1,IRS1,FGFR1,FGFR2,PIK3CD
**Estrogen-mediated S-phase Entry**	2,10E+00	2,92E-01	NaN	CCNE2,TFDP1,E2F5,RBL1,ESR1,CDK2,SKP2
**D-myo-inositol (1,4,5,6)-Tetrakisphosphate I**	2,07E+00	1,61E-01	NaN	PPFIA1,SOCS3,MINPP1,PPTC7,PPP1R14A,PAWR,ACP5,PTPRM,SACM1L,PTEN,MTMR6,PTPRC,TNS3,PTPN11,INPP5F,PPP1R12A,PPP1R16B,PPP2R3A,PTPN1,MTMR2,PPP2R5E,PTPN22,PPP3CA
**D-myo-inositol (3,4,5,6)-tetrakisphosphate E**	2,07E+00	1,61E-01	NaN	PPFIA1,SOCS3,MINPP1,PPTC7,PPP1R14A,PAWR,ACP5,PTPRM,SACM1L,PTEN,MTMR6,PTPRC,TNS3,PTPN11,INPP5F,PPP1R12A,PPP1R16B,PPP2R3A,PTPN1,MTMR2,PPP2R5E,PTPN22,PPP3CA
**iNOS Signaling**	2,04E+00	2,27E-01	NaN	CALM1 (includes others),LY96,NFKBIA,JAK1,MAPK14,CREBBP,CD14,IFNGR1,STAT1,NFKB1
**IL-17A Signaling in Gastric Cells**	2,00E+00	2,80E-01	NaN	CXCL10,MAP2K4,MAPK14,CCL5,NFKB1,TNF,EGFR
**Nur77 Signaling in T Lymphocytes**	1,98E+00	2,03E-01	NaN	HDAC9,CD3G,CALM1 (includes others),CD28,CASP3,CD80,FCER1G,CD86,CD3D,PPP3CA,BCL2,MAP3K2
**Cellular Effects of Sildenafil (Viagra)**	1,95E+00	1,62E-01	NaN	PRKACB,GUCY1A3,PPP1CB,MYLK,PLCL2,ITPR1,PDE4B,PDE1A,MYL9,ADCY9,CALM1 (includes others),PRKAR2B,PPP1R12A,ACTA2,PLCG2,PLCB1,PDE5A,PDE4D,ACTC1,GUCY1B3,MYL12A
**3-phosphoinositide Degradation**	1,88E+00	1,53E-01	NaN	PPFIA1,SOCS3,MINPP1,PPTC7,PPP1R14A,PAWR,ACP5,PTPRM,SACM1L,PTEN,MTMR6,PTPRC,TNS3,PTPN11,INPP5F,PPP1R12A,PPP1R16B,PPP2R3A,PTPN1,MTM1,MTMR2,PPP2R5E,PTPN22,PPP3CA
**Role of Wnt/GSK-3Î² Signaling in the Pathog**	1,87E+00	1,84E-01	NaN	TCF4,CSNK1G3,FZD1,NCOA3,APC,NCOA4,FZD8,FZD6,NCOA1,LEF1,GSK3B,FZD2,WNT5A,FZD7
**Calcium Transport I**	1,79E+00	4,00E-01	NaN	ATP2B1,ATP2C1,ANXA5,ATP2A2

**EIF2 Signaling**	1,79E+00	1,40E-01	NaN	RAF1,PIK3R1,SOS2,PPP1CB,HSPA5,EIF2S1,EIF4E,BCL2,PIK3CG,PIK3C3,PAIP1,SOS1,EIF5,AKT3,GSK3B,MAP2K1,ACTC1,NOX4,NRAS,PIK3C2A,FGFR1,FGFR2,PIK3R3,WARS,RRAS2,ACTA2,PTPN11,IRS1,PIK3CD,EIF2AK2,EIF1AX
**Role of BRCA1 in DNA Damage Response**	1,77E+00	1,79E-01	NaN	PBRM1,RBL2,ATF1,TOPBP1,RBL1,RFC5,CHEK1,NBN,SMARCA2,E2F5,MSH6,SMARCC1,STAT1,RFC3
**Primary Immunodeficiency Signaling**	1,77E+00	2,08E-01	NaN	BLNK,PTPRC,BTK,IL7R,CD4,ZAP70,ICOS,CD79A,CD8A,CD3D
**LPS/IL-1 Mediated Inhibition of RXR Functio**	1,76E+00	1,40E-01	NaN	MAP2K4,APOE,SLC27A2,CHST15,ABCA1,CHST2,GSTM2,ALDH1A1,ALDH1A3,CHST11,ACSL4,XPO1,FABP5,IL1RAP,ALDH6A1,ABCB1,GSTA4,GSTM3,MAP3K1,IL1R1,IL33,HS3ST3B1,LY96,IL1RN,CAT,APOC1,NCOA1,CD14,TNF,ACSL1,MAOA
**Calcium Signaling**	1,76E+00	1,46E-01	NaN	PRKACB,HDAC9,TRPC1,HDAC4,ATP2B1,ATP2C1,CREBBP,MEF2A,TPM1,ITPR1,TPM2,ATP2A2,RAP1A,ATF2,MYL9,RCAN1,CALM1 (includes others),PRKAR2B,ACTA2,CREB1,MEF2C,ASPH,SLC8A1,RCAN2,ACTC1,PPP3CA
**Cell Cycle: G2/M DNA Damage Checkpoint R**	1,71E+00	2,04E-01	NaN	YWHAQ,PRKDC,KAT2B,WEE1,CUL1,YWHAZ,TOP2A,CCNB2,CHEK1,SKP2
**RAN Signaling**	1,71E+00	3,12E-01	NaN	KPNA4,TNPO1,XPO1,KPNA1,IPO5
**Basal Cell Carcinoma Signaling**	1,69E+00	1,81E-01	NaN	TCF4,BMP2,HHIP,FZD1,APC,FZD8,FZD6,BMP7,LEF1,GSK3B,FZD2,WNT5A,FZD7
**Hypoxia Signaling in the Cardiovascular Syst**	1,66E+00	1,85E-01	NaN	NFKBIA,UBE2J1,CREBBP,CREB1,HSP90AA1,UBE2V2,HIF1A,UBE2D1,UBE2D3,ARNT,PTEN,ATF2
**Induction of Apoptosis by HIV1**	1,52E+00	1,83E-01	NaN	MAP2K4,RIPK1,NFKBIA,SLC25A4,CASP3,CXCR4,NFKB1,BIRC3,TNF,BCL2,BIRC2
**IL-22 Signaling**	1,52E+00	2,50E-01	NaN	MAP2K4,SOCS3,JAK1,MAPK14,AKT3,STAT1
**Role of JAK1, JAK2 and TYK2 in Interferon Si**	1,52E+00	2,50E-01	NaN	RAF1,JAK1,IFNGR1,IFNAR2,STAT1,NFKB1
**4-1BB Signaling in T Lymphocytes**	1,50E+00	2,26E-01	NaN	MAP2K4,TNFRSF9,NFKBIA,MAPK14,NFKB1,MAP2K1,ATF2
**Dopamine Receptor Signaling**	1,47E+00	1,69E-01	NaN	PRKACB,ADCY9,GCH1,PRKAR2B,PPP1R12A,PPP2R3A,PPP1R3C,PPP1CB,PPP1R14A,PPP2R5E,SLC18A2,PPP2R1B,MAOA
**G Protein Signaling Mediated by Tubby**	1,43E+00	2,19E-01	NaN	GNB4,GNG11,PLCG2,GNG2,GNAQ,PLCB1,GNG12
**nNOS Signaling in Neurons**	1,41E+00	1,91E-01	NaN	CALM1 (includes others),PRKCI,PRKCQ,PRKCH,PRKD3,PPP3CA,PRKD1,PRKCB,PRKCA
**DNA damage-induced 14-3-3Ïƒ Signaling**	1,39E+00	2,63E-01	NaN	CCNE2,CCNB2,AKT3,CDK2,RAD1
**Choline Biosynthesis III**	1,38E+00	3,08E-01	NaN	PLD4,HMOX1,PLD3,PLD1
**Circadian Rhythm Signaling**	1,37E+00	2,12E-01	NaN	PER3,ARNTL,CREBBP,CREB1,CLOCK,CRY1,ATF2
**Lipid Antigen Presentation by CD1**	1,37E+00	2,31E-01	NaN	CD1D,CD1B,FCER1G,CANX,PSAP,CD1C
**Graft-versus-Host Disease Signaling**	1,36E+00	1,88E-01	NaN	IL33,CD28,PRF1,CD80,IL1RN,FCER1G,CD86,IL6,TNF
**TWEAK Signaling**	1,30E+00	2,06E-01	NaN	TRAF3,RIPK1,NFKBIA,CASP3,NFKB1,BIRC3,BIRC2
**PXR/RXR Activation**	1,30E+00	1,69E-01	NaN	PRKACB,ABCB1,GSTM2,PRKAR2B,ALDH1A1,FOXO1,NCOA1,AKT3,IL6,NR3C1,TNF
**Regulation of Cellular Mechanics by Calpain**	1,30E+00	1,75E-01	NaN	PTK2,ITGB1,RRAS2,NRAS,ITGA5,VCL,ACTN1,CDK2,ITGA4,EGFR
**IL-15 Production**	1,30E+00	2,22E-01	NaN	PTK2,PRKCI,JAK1,IL6,STAT1,NFKB1
**D-myo-inositol (1,4,5)-Trisphosphate Biosyn**	1,30E+00	2,22E-01	NaN	PLD4,PIP5K1A,PLCG2,PLCB1,PI4K2B,PIP4K2A
DNA Double-Strand Break Repair by Non-Hor	1,28E+00	2,86E-01	NaN	PRKDC,WRN,XRCC5,NBN
IL-17A Signaling in Fibroblasts	1,25E+00	2,00E-01	NaN	NFKBIA,MAPK14,CCL2,IL6,GSK3B,NFKB1,CCL7
Phagosome Maturation	1,25E+00	1,39E-01	NaN	CTSK,NOX4,CTSO,ATP6V1C1,CTSW,TUBB2A,RAB7A,CTSG,CANX,ATP6V0D2,ATP6V1A,LAMP2,RAB5A,TUBA1A,CTSS,PIK3C3,NCF2,CYBB,VAMP3,CTSC
GÎ±s Signaling	1,24E+00	1,47E-01	NaN	PRKACB,RGS2,GNG2,CREBBP,RAP1A,ATF2,ADCY9,GNB4,GNG11,PRKAR2B,ADD3,CREB1,HCK,MAP2K1,GNG12,ADRB2
Protein Ubiquitination Pathway	1,24E+00	1,25E-01	NaN	USP24,USP12,USP18,CDC20,DNAJB4,CUL1,HSPB8,UBE2V2,HSPA5,DNAJA1,USP8,USP16,UCHL5,BIRC3,NEDD4,USP15,USP1,DNAJB9,UBE2D1,DNAJB14,UBE3A,SKP2,UBE2J1,HSPA13,HSP90AA1,USP46,SMURF2,DNAJB6,USP34,USP25,UBE2D3,BIRC2
Role of RIG1-like Receptors in Antiviral Innate	1,23E+00	1,86E-01	NaN	IFIH1,TANK,TRAF3,RIPK1,NFKBIA,CREBBP,DDX58,NFKB1
Telomere Extension by Telomerase	1,19E+00	2,67E-01	NaN	TNKS2,TERF1,XRCC5,NBN
Prostanoid Biosynthesis	1,18E+00	3,33E-01	NaN	PTGIS,PTGS1,HPGDS
autophagy	1,18E+00	1,67E-01	NaN	LAMP2,CTSK,CTSO,CTSS,PIK3C3,CTSW,CTSG,RB1CC1,CTSC,BCL2
CD27 Signaling in Lymphocytes	1,18E+00	1,73E-01	NaN	MAP2K4,NFKBIA,CASP3,MAP3K1,MAP3K8,CD27,NFKB1,MAP2K1,MAP3K2
Polyamine Regulation in Colon Cancer	1,17E+00	2,27E-01	NaN	TCF4,MAX,AZIN1,APC,ODC1
Phospholipases	1,10E+00	1,61E-01	NaN	PLD4,PNPLA8,PLA2G4A,HMOX1,PLD3,PLCG2,PLCB1,PLCL2,PLA2G7,PLD1
Extrinsic Prothrombin Activation Pathway	1,10E+00	2,50E-01	NaN	PROS1,F13A1,THBD,TFPI
Mismatch Repair in Eukaryotes	1,10E+00	2,50E-01	NaN	PCNA,MSH6,RFC5,RFC3
April Mediated Signaling	1,10E+00	1,84E-01	NaN	MAP2K4,TRAF3,NFKBIA,MAPK14,MAP3K1,NFKB1,TNFRSF17
GPCR-Mediated Integration of Enteroendocri	1,05E+00	1,53E-01	NaN	PRKACB,ADCY9,GNAI3,PRKAR2B,PLCG2,GNAQ,GNAI1,PLCB1,PLCL2,ITPR1,ADRB2
Tumoricidal Function of Hepatic Natural Kille	1,03E+00	2,08E-01	NaN	PRF1,ICAM1,CASP3,SRGN,ITGAL
Cytotoxic T Lymphocyte-mediated Apoptosis	1,00E+00	1,88E-01	NaN	CD3G,PRF1,CASP3,FCER1G,CD3D,BCL2
B Cell Activating Factor Signaling	1,00E+00	1,75E-01	NaN	MAP2K4,TRAF3,NFKBIA,MAPK14,MAP3K1,NFKB1,TNFRSF17
Thyroid Cancer Signaling	1,00E+00	1,75E-01	NaN	TCF4,RRAS2,NRAS,BDNF,CXCL12,LEF1,MAP2K1
Myo-inositol Biosynthesis	9,80E-01	4,00E-01	NaN	IMPAD1,INPP5F
Ethanol Degradation IV	9,73E-01	2,00E-01	NaN	TYRP1,ALDH1A1,ALDH1A3,CAT,ACSL1
MIF-mediated Glucocorticoid Regulation	9,56E-01	1,82E-01	NaN	PLA2G4A,LY96,NFKBIA,CD14,NFKB1,NR3C1
Eicosanoid Signaling	9,29E-01	1,49E-01	NaN	PTGIS,PNPLA8,PTGFR,PLA2G4A,PTGER3,PTGS1,ALOX5AP,HPGDS,ALOX5,PLA2G7
GADD45 Signaling	8,82E-01	2,11E-01	NaN	PCNA,CCNE2,GADD45B,CDK2
Hematopoiesis from Multipotent Stem Cells	8,77E-01	2,50E-01	NaN	KITLG,CSF1,IL7
Acyl Carrier Protein Metabolism	8,67E-01	1,00E+00	NaN	AASDHPPT
Xanthine and Xanthosine Salvage	8,67E-01	1,00E+00	NaN	PNP
Histamine Biosynthesis	8,67E-01	1,00E+00	NaN	HDC
Cell Cycle Regulation by BTG Family Proteins	8,67E-01	1,71E-01	NaN	CCNE2,PPP2R3A,E2F5,PPP2R5E,PPP2R1B,CDK2
B Cell Development	8,67E-01	1,71E-01	NaN	PTPRC,IL7R,CD80,CD86,CD79A,IL7
Gustation Pathway	8,62E-01	1,29E-01	NaN	SMPDL3A,PRKACB,LPAR4,GNG2,P2RX5,PDE4B,ITPR1,PDE1A,PDE8A,ADCY9,P2RY10,GNG11,PRKAR2B,P2RY14,PDE5A,PDE4D
Chondroitin and Dermatan Biosynthesis	8,47E-01	3,33E-01	NaN	CSGALNACT2,CHSY1
UDP-N-acetyl-D-glucosamine Biosynthesis II	8,47E-01	3,33E-01	NaN	GFPT1,UAP1
Purine Nucleotides Degradation II (Aerobic)	8,29E-01	2,00E-01	NaN	CECR1,GDA,PNP,AOX1
Superpathway of Cholesterol Biosynthesis	8,19E-01	1,79E-01	NaN	SQLE,IDI1,ACAT1,HMGCR,LBR
Fatty Acid Activation	8,09E-01	2,31E-01	NaN	SLC27A2,ACSL4,ACSL1
Mevalonate Pathway I	8,09E-01	2,31E-01	NaN	IDI1,ACAT1,HMGCR
Guanosine Nucleotides Degradation III	8,09E-01	2,31E-01	NaN	GDA,PNP,AOX1
Unfolded protein response	7,94E-01	1,48E-01	NaN	UBXN4,INSIG1,EDEM1,CANX,DNAJA2,DNAJB9,HSPA5,BCL2
Role of Oct4 in Mammalian Embryonic Stem	7,70E-01	1,52E-01	NaN	NR2F1,KDM5B,SPP1,BMI1,NR2F2,MEF2A,SH3GLB1
Ceramide Biosynthesis	7,40E-01	2,86E-01	NaN	SPTLC2,KDSR
Adenine and Adenosine Salvage III	7,40E-01	2,86E-01	NaN	CECR1,PNP
Ceramide Degradation	7,40E-01	2,86E-01	NaN	ACER3,ASAH1
Netrin Signaling	7,12E-01	1,54E-01	NaN	PRKACB,PRKAR2B,UNC5B,NCK1,PPP3CA,ENAH
tRNA Splicing	7,12E-01	1,54E-01	NaN	SMPDL3A,PDE8A,PDE5A,PDE4D,PDE4B,PDE1A
Neuroprotective Role of THOP1 in Alzheimer'	6,77E-01	1,50E-01	NaN	PRKACB,PRKAR2B,CREB1,PNOC,MMP9,APP
Transcriptional Regulatory Network in Embry	6,77E-01	1,50E-01	NaN	RIF1,CDYL,KAT6A,MEIS1,FOXC1,ZFHX3
nNOS Signaling in Skeletal Muscle Cells	6,77E-01	2,00E-01	NaN	SNTB2,CALM1 (includes others),DMD
Airway Pathology in Chronic Obstructive Pulm	6,54E-01	2,50E-01	NaN	TNF,MMP9
Superoxide Radicals Degradation	6,54E-01	2,50E-01	NaN	TYRP1,CAT
Tryptophan Degradation to 2-amino-3-carbo:	6,54E-01	2,50E-01	NaN	KMO,KYNU
Purine Ribonucleosides Degradation to Ribos	6,54E-01	2,50E-01	NaN	CECR1,PNP
Salvage Pathways of Pyrimidine Deoxyribonu	6,54E-01	2,50E-01	NaN	APOBEC3B,APOBEC3G
Sphingomyelin Metabolism	6,54E-01	2,50E-01	NaN	SGMS2,SGMS1
MIF Regulation of Innate Immunity	6,50E-01	1,46E-01	NaN	MAP2K4,PLA2G4A,LY96,NFKBIA,CD14,NFKB1
Superpathway of D-myo-inositol (1,4,5)-trispl	6,37E-01	1,67E-01	NaN	MINPP1,IMPAD1,INPP5F,PTEN
Granzyme B Signaling	6,36E-01	1,88E-01	NaN	PRKDC,PRF1,CASP3
Parkinson's Signaling	6,36E-01	1,88E-01	NaN	MAPK14,CASP3,SNCA
Dermatan Sulfate Biosynthesis	6,36E-01	1,33E-01	NaN	CHST2,HS3ST3B1,CSGALNACT2,XYLT1,CHSY1,CHST11,CHST15,DSE
UDP-D-xylose and UDP-D-glucuronate Biosyn	6,36E-01	5,00E-01	NaN	UGDH
Epoxysqualene Biosynthesis	6,36E-01	5,00E-01	NaN	SQLE
Î²-alanine Degradation I	6,36E-01	5,00E-01	NaN	ALDH6A1
Guanine and Guanosine Salvage I	6,36E-01	5,00E-01	NaN	PNP
Spermine Biosynthesis	6,36E-01	5,00E-01	NaN	AMD1
GDP-L-fucose Biosynthesis II (from L-fucose)	6,36E-01	5,00E-01	NaN	FPGT
Spermidine Biosynthesis I	6,36E-01	5,00E-01	NaN	AMD1

Adenine and Adenosine Salvage I	6,36E-01	5,00E-01	NaN	PNP
Putrescine Biosynthesis III	6,36E-01	5,00E-01	NaN	ODC1
Glutamate Biosynthesis II	6,36E-01	5,00E-01	NaN	GLUD2
Glutamate Degradation X	6,36E-01	5,00E-01	NaN	GLUD2
Amyloid Processing	6,36E-01	1,37E-01	NaN	PRKACB,PRKAR2B,MAPK14,AKT3,GSK3B,CDK5R1,APP
OX40 Signaling Pathway	6,27E-01	1,24E-01	NaN	MAP2K4,CD3G,TRAF3,TNFSF4,NFKBIA,TNFRSF4,CD4,FCER1G,NFKB1,CD3D,BCL2
Retinoic acid Mediated Apoptosis Signaling	6,17E-01	1,31E-01	NaN	CASP3,TIPARP,RARB,ZC3HAV1,PARP8,TNFSF10,TNKS2,PARP9
Sphingosine and Sphingosine-1-phosphate M	5,96E-01	2,22E-01	NaN	ACER3,ASAH1
Ketolysis	5,96E-01	2,22E-01	NaN	ACAT1,OXCT1
Î³-linolenate Biosynthesis II (Animals)	5,96E-01	1,76E-01	NaN	SLC27A2,ACSL4,ACSL1
Mitochondrial L-carnitine Shuttle Pathway	5,96E-01	1,76E-01	NaN	SLC27A2,ACSL4,ACSL1
Adenosine Nucleotides Degradation II	5,96E-01	1,76E-01	NaN	CECR1,PNP,AOX1
Superpathway of Geranylgeranyldiphosphate	5,96E-01	1,76E-01	NaN	IDI1,ACAT1,HMGCR
Role of Cytokines in Mediating Communicatio	5,60E-01	1,30E-01	NaN	IL33,IL12B,IL10,TGFB1,IL1RN,IL6,TNF
D-myo-inositol (1,3,4)-trisphosphate Biosynth	5,00E-01	1,58E-01	NaN	MINPP1,INPP5F,PTEN
Autoimmune Thyroid Disease Signaling	4,97E-01	1,28E-01	NaN	CD28,PRF1,CD80,IL10,FCER1G,CD86
5-aminoimidazole Ribonucleotide Biosynthes	4,97E-01	3,33E-01	NaN	PPAT
Tetrahydrobiopterin Biosynthesis I	4,97E-01	3,33E-01	NaN	GCH1
Tetrahydrobiopterin Biosynthesis II	4,97E-01	3,33E-01	NaN	GCH1
Chondroitin Sulfate Biosynthesis	4,65E-01	1,21E-01	NaN	CHST2,HS3ST3B1,CSGALNACT2,XYLT1,CHSY1,CHST11,CHST15
Glutathione-mediated Detoxification	4,40E-01	1,33E-01	NaN	GSTM2,GSTA4,GSTM3,HPGDS
Sonic Hedgehog Signaling	4,40E-01	1,33E-01	NaN	PRKACB,PRKAR2B,HHIP,GSK3B
Oxidative Ethanol Degradation III	4,29E-01	1,43E-01	NaN	ALDH1A1,ALDH1A3,ACSL1
Endoplasmic Reticulum Stress Pathway	4,29E-01	1,43E-01	NaN	CASP3,HSPA5,EIF2S1
Inflammasome pathway	4,29E-01	1,43E-01	NaN	NLRP3,NEK7,NFKB1
Chondroitin Sulfate Biosynthesis (Late Stages	4,28E-01	1,20E-01	NaN	CHST2,HS3ST3B1,CSGALNACT2,CHSY1,CHST11,CHST15
Glycogen Degradation II	4,27E-01	1,67E-01	NaN	PYGL,AGL
Airway Inflammation in Asthma	4,09E-01	2,50E-01	NaN	TNF
Arsenate Detoxification I (Glutaredoxin)	4,09E-01	2,50E-01	NaN	PNP
Uracil Degradation II (Reductive)	4,09E-01	2,50E-01	NaN	DPYD
Heme Degradation	4,09E-01	2,50E-01	NaN	HMOX1
Phenylethylamine Degradation I	4,09E-01	2,50E-01	NaN	AOC3
Thymine Degradation	4,09E-01	2,50E-01	NaN	DPYD
Melatonin Degradation II	4,09E-01	2,50E-01	NaN	MAOA
Acetate Conversion to Acetyl-CoA	4,09E-01	2,50E-01	NaN	ACSL1
Fatty Acid Î²-oxidation I	3,95E-01	1,25E-01	NaN	SLC27A2,ACSL4,ACADM,ACSL1
Oleate Biosynthesis II (Animals)	3,93E-01	1,54E-01	NaN	UFSP2,ALDH6A1
Cholesterol Biosynthesis I	3,93E-01	1,54E-01	NaN	SQLE,LBR
Cholesterol Biosynthesis II (via 24,25-dihydro	3,93E-01	1,54E-01	NaN	SQLE,LBR
Cholesterol Biosynthesis III (via Desmosterol)	3,93E-01	1,54E-01	NaN	SQLE,LBR
Pyrimidine Deoxyribonucleotides De Novo Bi	3,77E-01	1,30E-01	NaN	RRM2,APOBEC3G,RRM1
Putrescine Degradation III	3,77E-01	1,30E-01	NaN	ALDH1A1,ALDH1A3,MAOA
Pyrimidine Ribonucleotides Interconversion	3,76E-01	1,16E-01	NaN	SMARCA1,ENTPD1,ANXA1,RECQL,DDX3X
FXR/RXR Activation	3,75E-01	1,03E-01	NaN	MAP2K4,APOE,CREBBP,SERPINF1,VLDLR,IL33,FOXO1,IL1RN,APOC1,AKT3,TNF,CLU,APOD
DNA Double-Strand Break Repair by Homolog	3,59E-01	1,43E-01	NaN	ATRX,NBN
Glycogen Degradation III	3,59E-01	1,43E-01	NaN	PYGL,AGL
Urate Biosynthesis/Inosine 5'-phosphate Deg	3,59E-01	1,43E-01	NaN	PNP,AOX1
Phenylalanine Degradation IV (Mammalian, v	3,59E-01	1,43E-01	NaN	SLC27A2,MAOA
Retinoate Biosynthesis I	3,56E-01	1,18E-01	NaN	ALDH1A1,ALDH1A3,RDH11,BMP2
Glutathione Redox Reactions I	3,56E-01	1,25E-01	NaN	CLIC2,GSTM2,GPX8
Tryptophan Degradation III (Eukaryotic)	3,56E-01	1,25E-01	NaN	KMO,ACAT1,KYNU
Tetrahydrofolate Salvage from 5,10-metheny	3,54E-01	2,00E-01	NaN	MTHFD2
Serine Biosynthesis	3,54E-01	2,00E-01	NaN	PSAT1
Lysine Degradation II	3,54E-01	2,00E-01	NaN	AASDHPPT
Lysine Degradation V	3,54E-01	2,00E-01	NaN	AASDHPPT
Trans, trans-farnesyl Diphosphate Biosynthes	3,54E-01	2,00E-01	NaN	IDI1
dTMP De Novo Biosynthesis	3,54E-01	2,00E-01	NaN	DHFR
Pyrimidine Ribonucleotides De Novo Biosynth	3,48E-01	1,11E-01	NaN	SMARCA1,ENTPD1,ANXA1,RECQL,DDX3X
Tryptophan Degradation X (Mammalian, via T	3,35E-01	1,20E-01	NaN	ALDH1A1,ALDH1A3,MAOA
NAD biosynthesis II (from tryptophan)	3,34E-01	1,33E-01	NaN	KMO,KYNU
Vitamin-C Transport	3,34E-01	1,33E-01	NaN	SLC23A2,TXNRD1
Dermatan Sulfate Biosynthesis (Late Stages)	3,12E-01	1,06E-01	NaN	CHST2,HS3ST3B1,CHST11,CHST15,DSE
Dermatan Sulfate Degradation (Metazoa)	3,08E-01	1,25E-01	NaN	IDS,HEXA
Arginine Biosynthesis IV	3,08E-01	1,67E-01	NaN	GLUD2
Pyruvate Fermentation to Lactate	3,08E-01	1,67E-01	NaN	LDHB
Thioredoxin Pathway	3,08E-01	1,67E-01	NaN	TXNRD1
Pentose Phosphate Pathway (Non-oxidative I	3,08E-01	1,67E-01	NaN	RPE
Glycerol Degradation I	3,08E-01	1,67E-01	NaN	GK
Zymosterol Biosynthesis	3,08E-01	1,67E-01	NaN	LBR
Glycogen Biosynthesis II (from UDP-D-Glucos	3,08E-01	1,67E-01	NaN	GBE1
Rapoport-Luebering Glycolytic Shunt	3,08E-01	1,67E-01	NaN	MINPP1
NAD Biosynthesis III	3,08E-01	1,67E-01	NaN	NAMPT
Notch Signaling	2,91E-01	1,05E-01	NaN	NOTCH2,NOTCH4,NUMB,RBPJ
Heparan Sulfate Biosynthesis	2,69E-01	9,64E-02	NaN	CHST2,HS3ST3B1,XYLT1,CHST11,EXT1,GLCE,EXTL2,CHST15
Superpathway of Serine and Glycine Biosynth	2,65E-01	1,43E-01	NaN	PSAT1
Acetyl-CoA Biosynthesis I (Pyruvate Dehydrog	2,65E-01	1,43E-01	NaN	DLAT
Glycoaminoglycan-protein Linkage Region Bio	2,65E-01	1,43E-01	NaN	XYLT1
D-myo-inositol (1,4,5)-trisphosphate Degrada	2,59E-01	1,11E-01	NaN	IMPAD1,INPP5F
Histamine Degradation	2,34E-01	1,05E-01	NaN	ALDH1A1,ALDH1A3
Phototransduction Pathway	2,31E-01	9,43E-02	NaN	PRKACB,PRKAR2B,GUCY1A3,OPN3,GUCY1B3
Histidine Degradation III	2,29E-01	1,25E-01	NaN	MTHFD2
Heparan Sulfate Biosynthesis (Late Stages)	2,26E-01	9,21E-02	NaN	CHST2,HS3ST3B1,CHST11,EXT1,GLCE,EXTL2,CHST15
DNA Methylation and Transcriptional Repres	2,16E-01	1,00E-01	NaN	ARID4B,RBBP4
Granzyme A Signaling	2,16E-01	1,00E-01	NaN	PRF1,CREBBP
Serotonin Receptor Signaling	2,16E-01	9,30E-02	NaN	ADCY9,GCH1,SLC18A2,MAOA
Phosphatidylethanolamine Biosynthesis II	2,02E-01	1,11E-01	NaN	ETNK1
Leucine Degradation I	2,02E-01	1,11E-01	NaN	ACADM
Folate Transformations I	2,02E-01	1,11E-01	NaN	MTHFD2
Fatty Acid Î±-oxidation	1,81E-01	9,09E-02	NaN	ALDH1A1,ALDH1A3
Ketogenesis	1,77E-01	1,00E-01	NaN	ACAT1
Pentose Phosphate Pathway	1,77E-01	1,00E-01	NaN	RPE
Purine Nucleotides De Novo Biosynthesis II	1,54E-01	9,09E-02	NaN	PPAT
UDP-N-acetyl-D-galactosamine Biosynthesis I	1,54E-01	9,09E-02	NaN	UAP1

Supplemetary Table 3
Predicted and/or validated metabolic genes targets of miR-27a as from the analysis of mirwalk 2.0 and mirmap tools.

Pathways	Valid./Pred.	Gene target	EntrezID	RefseqID	3P GENE																			5P GENE												cds GENE												reference
					mirmap	miRWalk					Comparative													miRWalk					Comparative							miRWalk					Comparative							
					miRmap score	Start position of miRNA seed	Seed length	Seed Start	Seed End	Pvalue	miRWalk	Microt4	miRanda	mirbridge	miRDB	miRMap	miRNAMap	Pictar2	PITA	RNA22	RNAhybrid	Targetscan	SUM	Start position of miRNA seed	Seed length	Seed Start	Seed End	Pvalue	miRWalk	miRanda	PITA	RNA22	RNAhybrid	Targetscan	SUM	Start position of miRNA seed	Seed length	Seed Start	Seed End	Pvalue	miRWalk	Microt4	miRanda	RNA22	RNAhybrid	Targetscan	SUM	
Glycolysis	P	ALDOA	226	NM_000034	45.777	1	7	2327	2321	0.0137	1	-	-	1	-	1	-	-	-	1	1	1	6	-	-	-	-	-	1	-	-	-	1	-	2	-	-	-	-	-	-	-	-	-	1	-	1	
	P	ALDOA	226	NM_184043	45.777	1	7	1488	1482	0.0137	1	-	-	-	-	1	-	-	-	-	1	1	4	-	-	-	-	-	-	-	-	-	-	-	-	-	-	-	-	-	-	-	-	-	1	-	1	
	P	ALDOA	226	NM_184041	45.777	1	7	1516	1510	0.0137	1	-	-	-	-	1	-	-	-	-	1	1	4	-	-	-	-	-	-	-	-	-	-	-	-	-	-	-	-	-	-	-	-	-	1	-	1	
	P	ALDOA	226	NM_001127617	45.777	1	7	1549	1543	0.0137	1	-	-	-	-	1	-	-	-	-	1	1	4	-	-	-	-	-	-	-	-	-	-	-	-	-	-	-	-	-	-	-	-	-	1	-	1	
	P	ALDOA	226	NM_001243177	45.777	1	7	1670	1664	0.0137	1	-	-	-	-	1	-	-	-	-	1	1	4	-	-	-	-	-	-	-	-	-	-	-	-	-	-	-	-	-	-	-	-	-	1	-	1	
	P	ALDOA	226	XM_005255170	45.777	1	7	2190	2184	0.0123	1	-	-	-	-	1	-	-	-	-	1	1	4	-	-	-	-	-	1	-	-	-	-	-	1	-	-	-	-	-	-	-	-	-	1	-	1	
	P	DLAT	1737	NM_001931	-	-	-	-	-	-	-	-	-	-	-	-	-	-	1	-	1	-	2	-	-	-	-	-	-	-	-	-	-	-	-	-	-	-	-	-	1	-	-	-	1	-	2	
	P	DLAT	1737	XM_005271421	-	-	-	-	-	-	-	-	-	-	-	-	-	-	-	-	1	-	1	-	-	-	-	-	-	-	-	-	-	-	-	-	-	-	-	-	1	-	-	-	1	-	2	
	P	DLAT	1737	XM_005277720	-	-	-	-	-	-	-	-	-	-	-	-	-	-	-	-	1	-	1	-	-	-	-	-	-	-	-	-	-	-	-	-	-	-	-	-	1	-	-	-	1	-	2	
	P	DLAT	1737	XM_005271420	-	-	-	-	-	-	-	-	-	-	-	-	-	-	-	-	1	-	1	-	-	-	-	-	-	-	-	-	-	-	-	-	-	-	-	-	1	-	-	-	1	-	2	
	P	DLAT	1737	XM_005277719	-	-	-	-	-	-	-	-	-	-	-	-	-	-	-	-	1	-	1	-	-	-	-	-	-	-	-	-	-	-	-	-	-	-	-	-	1	-	-	-	1	-	2	
	P	HK1	3098	NM_033500	-	-	-	-	-	-	1	-	-	-	-	-	1	-	-	1	1	-	4	-	-	-	-	-	-	-	-	-	1	-	1	1	9	1138	1130	0.0103	1	-	1	1	1	1	5	
	P	HK1	3098	NM_033496	-	-	-	-	-	-	1	-	-	-	-	-	1	-	-	-	1	-	3	-	-	-	-	-	-	-	-	-	1	-	1	1	9	773	765	0.0104	1	-	1	-	1	1	4	
	P	HK1	3098	NM_000188	-	-	-	-	-	-	1	-	-	-	-	-	1	-	-	-	1	-	3	-	-	-	-	-	-	-	-	-	-	-	-	1	9	776	768	0.0105	1	-	1	-	1	1	4	
	P	HK1	3098	XM_005269735	-	-	-	-	-	-	1	-	-	-	-	-	1	-	-	-	1	-	3	-	-	-	-	-	-	-	-	-	-	-	-	1	9	804	796	0.0109	1	-	1	-	1	1	4	
	P	HK1	3098	XM_005269736	-	-	-	-	-	-	1	-	-	-	-	-	1	-	-	-	1	-	3	-	-	-	-	-	-	-	-	-	-	-	-	1	9	885	877	0.0105	1	-	1	-	1	1	4	
	P	HK1	3098	NM_033497	-	-	-	-	-	-	1	-	-	-	-	-	1	-	-	-	1	-	3	-	-	-	-	-	-	-	-	-	-	-	-	1	9	991	983	0.0105	1	-	1	-	1	1	4	
	P	HK1	3098	XM_005269737	-	-	-	-	-	-	1	-	-	-	-	-	1	-	-	-	1	-	3	-	-	-	-	-	-	-	-	-	-	-	-	1	9	1047	1039	0.0101	1	-	1	-	1	1	4	
	P	HK1	3098	NM_033498	-	-	-	-	-	-	1	-	-	-	-	-	1	-	-	-	1	-	3	-	-	-	-	-	-	-	-	-	-	-	-	1	9	1045	1037	0.0105	1	-	1	-	1	1	4	
	P	HK2	3099	NM_000189	56.3844	-	-	-	-	-	-	1	-	-	-	1	-	-	1	1	1	1	6	-	-	-	-	-	1	-	-	-	1	-	2	1	9	3945	3937	0.0105	1	1	1	1	1	1	6	
	P	HK2	3099	XM_005264280	56.3844	-	-	-	-	-	-	-	-	-	-	1	-	-	-	-	1	1	3	-	-	-	-	-	-	-	-	-	1	-	1	1	9	2488	2480	0.0107	1	-	1	-	1	1	4	
	P	HK3	3101	NM_002115	-	-	-	-	-	-	-	-	-	-	-	-	-	-	-	-	-	-	-	-	-	-	-	-	1	-	-	1	1	-	3	-	-	-	-	-	1	-	-	1	1	1	4	
	P	GCK	2645	NM_000162	-	-	-	-	-	-	-	-	-	-	-	-	-	-	-	1	1	-	2	-	-	-	-	-	1	1	-	-	1	-	3	-	-	-	-	-	1	-	-	-	1	-	2	
	P	GCK	2645	NM_033507	-	-	-	-	-	-	-	-	-	-	-	-	-	-	-	-	1	-	1	-	-	-	-	-	-	-	-	-	-	-	-	-	-	-	-	-	1	-	-	-	1	-	2	
	P	GCK	2645	NM_033508	-	-	-	-	-	-	-	-	-	-	-	-	-	-	-	-	1	-	1	-	-	-	-	-	-	-	-	-	-	-	-	-	-	-	-	-	1	-	-	-	1	-	2	
	P	TPI1	7167	XM_005253762	-	-	-	-	-	-	-	1	-	-	-	-	-	-	-	1	1	-	3	-	-	-	-	-	-	-	-	-	-	-	-	-	-	-	-	-	-	-	-	1	1	-	2	
	P	TPI1	7167	XM_005277757	-	-	-	-	-	-	-	1	-	-	-	-	-	-	-	1	1	-	3	-	-	-	-	-	-	-	-	-	-	-	-	-	-	-	-	-	-	-	-	1	1	-	2	
	P	TPI1	7167	NM_000365	-	-	-	-	-	-	-	-	-	-	-	-	-	-	-	-	1	-	1	-	-	-	-	-	-	-	-	-	-	-	-	-	-	-	-	-	-	-	-	-	1	-	1	
	P	TPI1	7167	NM_001159287	-	-	-	-	-	-	-	-	-	-	-	-	-	-	-	-	1	-	1	-	-	-	-	-	-	-	-	-	-	-	-	-	-	-	-	-	-	-	-	-	1	-	1	
	P	TPI1	7167	NM_001258026	-	-	-	-	-	-	-	-	-	-	-	-	-	-	-	-	1	-	1	-	-	-	-	-	-	-	-	-	1	-	1	-	-	-	-	-	-	-	-	-	1	-	1	
	P	GAPDHS	26330	NM_014364	-	-	-	-	-	-	-	-	-	-	-	-	-	-	-	-	-	-	-	-	-	-	-	-	-	-	-	-	1	-	1	-	-	-	-	-	1	-	-	1	1	1	4	
	P	GAPDHS	26330	XM_005258732	-	-	-	-	-	-	-	-	-	-	-	-	-	-	-	-	-	-	-	-	-	-	-	-	-	-	-	-	-	-	-	-	-	-	-	-	1	-	-	-	1	1	3	
	P	PGK1	5230	NM_000291	-	-	-	-	-	-	-	-	-	-	-	-	-	-	-	-	-	-	-	-	-	-	-	-	-	-	-	-	-	-	-	-	-	-	-	-	1	-	-	-	1	-	2	
	P	PGK1	5230	XM_005262144	-	-	-	-	-	-	-	-	-	-	-	-	-	-	-	-	-	-	-	-	-	-	-	-	-	-	-	-	-	-	-	-	-	-	-	-	1	-	-	-	1	-	2	
	P	PGK1	5230	XM_005277808	-	-	-	-	-	-	-	-	-	-	-	-	-	-	-	-	-	-	-	-	-	-	-	-	-	-	-	-	-	-	-	-	-	-	-	-	1	-	-	-	1	-	2	
	P	PGK1	5230	XM_005262143	-	-	-	-	-	-	-	-	-	-	-	-	-	-	-	-	-	-	-	-	-	-	-	-	-	-	-	-	-	-	-	-	-	-	-	-	1	-	-	-	1	-	2	
	P	PGK1	5230	XM_005277807	-	-	-	-	-	-	-	-	-	-	-	-	-	-	-	-	-	-	-	-	-	-	-	-	-	-	-	-	-	-	-	-	-	-	-	-	1	-	-	-	1	-	2	
	P	PGK2	5232	NM_138733	-	-	-	-	-	-	-	-	-	-	-	-	-	-	-	-	-	-	-	-	-	-	-	-	-	-	-	-	-	-	-	-	-	-	-	-	1	0	0	0	1	0	2	
	P	PGAM4	441531	NM_001029891	-	-	-	-	-	-	-	-	-	-	-	-	-	-	-	1	1	-	2	-	-	-	-	-	-	-	-	-	-	-	-	-	-	-	-	-	-	-	-	1	1	-	2	
	P	PGAM5	192111	NM_001170543	26.7958	-	-	-	-	-	-	-	-	-	-	1	-	-	-	1	1	-	3	-	-	-	-	-	-	-	-	-	-	-	-	-	-	-	-	-	1	0	0	0	0	0	1	
	P	PGAM5	192111	NM_138575	26.7958	-	-	-	-	-	-	-	-	-	-	1	-	-	-	-	1	-	2	-	-	-	-	-	-	-	-	-	-	-	-	-	-	-	-	-	1	0	0	0	0	0	1	
	P	PGAM5	192111	NM_001170544	26.7958	-	-	-	-	-	-	-	-	-	-	1	-	-	-	-	1	-	2	-	-	-	-	-	-	-	-	-	-	-	-	-	-	-	-	-	1	0	0	0	0	0	1	
Autophagy	P	ATG5	9474	NM_004849	86.1449	-	-	-	-	-	-	1	-	-	-	1	-	-	1	-	1	-	4	-	-	-	-	-	-	-	-	-	1	-	1	-	-	-	-	-	-	-	-	-	1	-	1	
	P	ATG5	9474	XM_005267231	86.1449	-	-	-	-	-	-	-	-	-	-	1	-	-	-	-	1	-	2	-	-	-	-	-	-	-	-	-	1	-	1	-	-	-	-	-	-	-	-	-	1	-	1	
	P	ATG7	10533	NM_006395	81.2882	-	-	-	-	-	1	1	-	-	-	1	-	-	-	1	1	-	5	-	-	-	-	-	-	-	-	-	1	-	1	-	-	-	-	-	1	1	1	1	1	1	6	
	P	ATG7	10533	XM_005264803	81.2882	1	7	2222	2216	0.0104	1	-	-	-	-	1	-	-	-	-	1	1	4	-	-	-	-	-	-	-	-	-	1	-	1	-	-	-	-	-	1	-	1	-	1	1	4	
	P	ATG7	10533	XM_001144912	81.2882	-	-	-	-	-	1	-	-	-	-	1	-	-	-	-	1	-	3	-	-	-	-	-	-	-	-	-	1	-	1	-	-	-	-	-	1	-	1	-	1	1	4	
	P	ATG7	10533	NM_001136031	81.2882	-	-	-	-	-	1	-	-	-	-	1	-	-	-	-	1	-	3	-	-	-	-	-	-	-	-	-	1	-	1	-	-	-	-	-	1	-	1	-	1	1	4	
	P	ATG7	10533	XM_005264805	81.2882	-	-	-	-	-	-	-	-	-	-	1	-	-	-	-	1	-	2	-	-	-	-	-	-	-	-	-	1	-	1	-	-	-	-	-	1	-	1	-	1	1	4	
	P	ATG7	10533	XM_005264802	81.2882	-	-	-	-	-	-	-	-	-	-	1	-	-	-	-	1	-	2	-	-	-	-	-	-	-	-	-	-	-	-	-	-	-	-	-	1	-	1	-	1	1	4	
	P	ATG7	10533	XM_005264804	81.2882	-	-	-	-	-	-	-	-	-	-	1	-	-	-	-	1	-	2	-	-	-	-	-	-	-	-	-	1	-	1	-	-	-	-	-	1	-	1	-	1	1	4	
	P	ATG7	10533	XM_005264800	81.2882	-	-	-	-	-	-	-	-	-	-	1	-	-	-	-	1	-	2	-	-	-	-	-	-	-	-	-	1	-	1	-	-	-	-	-	1	-	1	-	1	1	4	
	P	ATG7	10533	XM_005264801	81.2882	-	-	-	-	-	-	-	-	-	-	1	-	-	-	-	1	-	2	-	-	-	-	-	-	-	-	-	-	-	-	-	-	-	-	-	1	-	1	-	1	1	4	
	P	BECN1	8678	NM_003766	-	-	-	-	-	-	-	-	-	-	-	-	-	-	-	-	-	-	-	-	-	-	-	-	-	-	-	-	-	-	-	-	-	-	-	-	-	-	1	-	-	-	1	
	P	BECN1	8678	XM_005257759	-	-	-	-	-	-	-	-	-	-	-	-	-	-	-	-	-	-	-	-	-	-	-	-	-	-	-	-	-	-	-	-	-	-	-	-	-	-	1	-	-	-	1	
	P	BECN1	8678	XM_005257760	-	-	-	-	-	-	-	-	-	-	-	-	-	-	-	-	-	-	-	-	-	-	-	-	-	-	-	-	-	-	-	-	-	-	-	-	-	-	1	-	-	-	1	
	P	BECN1	8678	XM_005257758	-	-	-	-	-	-	-	-	-	-	-	-	-	-	-	-	-	-	-	-	-	-	-	-	-	-	-	-	-	-	-	-	-	-	-	-	-	-	1	-	-	-	1	
Glutaminolysis	P	GLS	2744	NM_014905	-	-	-	-	-	-	-	-	-	-	-	-	-	-	-	-	-	-	-	-	-	-	-	-	-	-	-	-	-	-	-	-	-	-	-	-	-	-	-	-	1	-	1	
	P	GLS	2744	XM_005246468	-	-	-	-	-	-	-	-	-	-	-	-	-	-	-	-	-	-	-	-	-	-	-	-	-	-	-	-	-	-	-	-	-	-	-	-	-	-	-	-	1	-	1	
	P	GLS	2744	XM_005246467	-	-	-	-	-	-	-	-	-	-	-	-	-	-	-	-	-	-	-	-	-	-	-	-	-	-	-	-	-	-	-	-	-	-	-	-	-	-	-	-	1	-	1	
	P	GLS	2744	NM_001256310	-	-	-	-	-	-	-	-	-	-	-	-	-	-	-	-	-	-	-	-	-	-	-	-	-	-	-	-	-	-	-	-	-	-	-	-	-	-	-	-	1	-	1	
	P	GLUD2	2747	NM_012084	-	-	-	-	-	-	-	-	-	-	-	-	-	-	-	-	1	-	1	-	-	-	-	-	-	-	-	-	-	-	-	-	-	-	-	-	1	1	1	-	1	1	5	
Fatty acid biosynthesis & β-Oxidation	P	ACAD9	28976	NM_014049	-	-	-	-	-	-	-	-	-	-	-	-	-	-	-	1	-	-	1	-	-	-	-	-	-	-	-	-	-	-	-	-	-	-	-	-	1	-	1	-	1	1	4	
	P	ACAD8	27034	NM_014384	70.333	-	-	-	-	-	-	-	-	-	-	1	-	-	1	-	1	-	3	-	-	-	-	-	-	-	-	-	-	-	-	-	-	-	-	-	-	-	-	-	1	-	1	
	P	ACAD8	27034	XM_005271504	70.333	-	-	-	-	-	-	-	-	-	-	1	-	-	-	-	1	-	2	-	-	-	-	-	-	-	-	-	-	-	-	-	-	-	-	-	-	-	-	-	1	-	1	
	P	ACAD8	27034	XM_005271501	70.333	-	-	-	-	-	-	-	-	-	-	1	-	-	-	-	-	-	1	-	-	-	-	-	-	-	-	-	-	-	-	-	-	-	-	-	-	-	-	-	1	-	1	
	P	ACAD8	27034	XM_005271505	70.333	-	-	-	-	-	-	-	-	-	-	1	-	-	-	-	-	-	1	-	-	-	-	-	-	-	-	-	-	-	-	-	-	-	-	-	-	-	-	-	1	-	1	
	P	ACAD8	27034	XM_005271503	70.333	-	-	-	-	-	-	-	-	-	-	1	-	-	-	-	-	-	1	-	-	-	-	-	-	-	-	-	1	-	1	-	-	-	-	-	-	-	-	-	1	-	1	
	P	ACAD8	27034	XM_005271502	70.333	-	-	-	-	-	-	-	-	-	-	1	-	-	-	-	-	-	1	-	-	-	-	-	-	-	-	-	1	-	1	-	-	-	-	-	-	-	-	-	1	-	1	
	P	CPT1A	1374	NM_001876	15.8568	-	-	-	-	-	1	1	-	-	-	1	-	-	1	1	1	1	7	-	-	-	-	-	-	-	-	-	-	-	-	-	-	-	-	-	1	1	1	1	1	1	6	
	P	CPT1A	1374	XM_005273762	15.8568	-	-	-	-	-	1	-	-	-	-	1	-	-	-	-	1	1	4	-	-	-	-	-	-	-	-	-	-	-	-	-	-	-	-	-	1	-	1	-	1	1	4	
	P	CPT1A	1374	XM_005273764	15.8568	-	-	-	-	-	1	-	-	-	-	1	-	-	-	-	1	1	4	-	-	-	-	-	-	-	-	-	-	-	-	-	-	-	-	-	1	-	1	-	1	1	4	
	P	CPT1A	1374	XM_005273763	15.8568	-	-	-	-	-	-	-	-	-	-	1	-	-	-	-	-	-	1	-	-	-	-	-	-	-	-	-	-	-	-	-	-	-	-	-	1	-	1	-	1	1	4	
	P	CPT1A	1374	NM_001031847	15.8568	-	-	-	-	-	-	-	-	-	-	1	-	-	-	-	-	-	1	-	-	-	-	-	-	-	-	-	-	-	-	-	-	-	-	-	1	-	1	-	1	1	4	
	P	CPT1B	1375	NM_152245	-	-	-	-	-	-	-	-	-	-	-	-	-	-	-	-	1	-	1	-	-	-	-	-	-	-	-	-	-	-	-	-	-	-	-	-	-	-	-	-	1	-	1	
	P	CPT1B	1375	NM_001145136	-	-	-	-	-	-	-	-	-	-	-	-	-	-	-	-	-	-	-	-	-	-	-	-	-	-	-	-	-	-	-	-	-	-	-	-	-	-	-	-	1	-	1	
	P	CPT1B	1375	NM_001145134	-	-	-	-	-	-	-	-	-	-	-	-	-	-	-	-	-	-	-	-	-	-	-	-	-	-	-	-	-	-	-	-	-	-	-	-	-	-	-	-	1	-	1	
	P	CPT1B	1375	NM_152246	-	-	-	-	-	-	-	-	-	-	-	-	-	-	-	-	-	-	-	-	-	-	-	-	-	-	-	-	-	-	-	-	-	-	-	-	-	-	-	-	1	-	1	
	P	CPT1B	1375	NM_001145135	-	-	-	-	-	-	-	-	-	-	-	-	-	-	-	-	-	-	-	-	-	-	-	-	-	-	-	-	-	-	-	-	-	-	-	-	-	-	-	-	1	-	1	
	P	CPT1B	1375	NM_001145137	-	-	-	-	-	-	-	-	-	-	-	-	-	-	-	-	-	-	-	-	-	-	-	-	-	-	-	-	-	-	-	-	-	-	-	-	-	-	-	-	1	-	1	
	P	CPT1B	1375	NM_004377	-	-	-	-	-	-	-	-	-	-	-	-	-	-	-	-	1	-	1	-	-	-	-	-	-	-	-	-	-	-	-	-	-	-	-	-	-	-	-	-	1	-	1	
	P	CPT1C	126129	NM_001199752	-	-	-	-	-	-	-	-	-	-	-	-	-	-	-	-	-	-	-	-	-	-	-	-	-	-	-	-	-	-	-	-	-	-	-	-	1	-	-	-	1	-	2	
	P	CPT1C	126129	XM_005258506	-	-	-	-	-	-	-	-	-	-	-	-	-	-	-	-	-	-	-	-	-	-	-	-	-	-	-	-	-	-	-	-	-	-	-	-	1	-	-	-	1	-	2	
	P	CPT1C	126129	XM_005258507	-	-	-	-	-	-	-	-	-	-	-	-	-	-	-	-	-	-	-	-	-	-	-	-	-	-	-	-	-	-	-	-	-	-	-	-	1	-	-	-	1	-	2	
	P	CPT1C	126129	NM_152359	-	-	-	-	-	-	-	-	-	-	-	-	-	-	-	-	-	-	-	-	-	-	-	-	-	-	-	-	-	-	-	-	-	-	-	-	1	-	-	-	1	-	2	
	P	CPT1C	126129	NM_001136052	-	-	-	-	-	-	-	-	-	-	-	-	-	-	-	-	-	-	-	-	-	-	-	-	-	-	-	-	-	-	-	-	-	-	-	-	1	-	-	-	1	-	2	
	P	CPT1C	126129	NM_001199753	-	-	-	-	-	-	-	-	-	-	-	-	-	-	-	-	-	-	-	-	-	-	-	-	-	-	-	-	-	-	-	-	-	-	-	-	1	-	-	-	1	-	2	
	P	CPT1C	126129	XM_005258505	-	-	-	-	-	-	-	-	-	-	-	-	-	-	-	-	-	-	-	-	-	-	-	-	-	-	-	-	-	-	-	-	-	-	-	-	1	-	-	-	1	-	2	
	P	CPT1C	126129	XM_005258509	-	-	-	-	-	-	-	-	-	-	-	-	-	-	-	-	-	-	-	-	-	-	-	-	-	-	-	-	-	-	-	-	-	-	-	-	1	-	-	-	-	-	1	
	P	CPT1C	126129	XM_005258508	-	-	-	-	-	-	-	-	-	-	-	-	-	-	-	-	-	-	-	-	-	-	-	-	-	-	-	-	-	-	-	-	-	-	-	-	1	-	-	-	-	-	1	
	P	CPT2	1376	NM_000098	58.8508	-	-	-	-	-	1	1	-	-	-	1	-	-	1	-	1	1	6	-	-	-	-	-	-	-	-	-	-	-	-	-	-	-	-	-	-	-	-	1	1	-	2	
	P	CPT2	1376	XM_005270484	58.8508	-	-	-	-	-	1	-	-	-	-	1	-	-	-	-	1	1	4	-	-	-	-	-	-	-	-	-	-	-	-	-	-	-	-	-	-	-	-	-	1	-	1	
	P	ACACA	31	NM_198839	96.2127	-	-	-	-	-	1	-	1	-	-	1	1	-	-	1	1	1	7	-	-	-	-	-	-	-	-	1	1	-	2	-	-	-	-	-	1	1	1	1	1	1	6	
	P	ACACA	31	XM_005257268	96.2127	-	-	-	-	-	1	-	1	-	-	1	1	-	-	-	1	1	6	-	-	-	-	-	-	-	-	-	-	-	-	-	-	-	-	-	1	-	1	-	1	1	4	
	P	ACACA	31	XM_005257266	96.2127	-	-	-	-	-	1	-	1	-	-	1	1	-	-	-	1	1	6	-	-	-	-	-	-	-	-	-	-	-	-	-	-	-	-	-	1	-	1	-	1	1	4	
	P	ACACA	31	XM_005257267	96.2127	-	-	-	-	-	1	-	1	-	-	1	1	-	-	-	1	1	6	-	-	-	-	-	-	-	-	-	-	-	-	-	-	-	-	-	1	-	1	-	1	1	4	
	P	ACACA	31	NM_198836	96.2127	-	-	-	-	-	1	-	1	-	-	1	1	-	-	-	1	1	6	-	-	-	-	-	-	-	-	-	-	-	-	-	-	-	-	-	1	-	1	-	1	1	4	
	P	ACACA	31	NM_198837	96.2127	-	-	-	-	-	1	-	1	-	-	1	1	-	-	-	1	1	6	-	-	-	-	-	-	-	-	-	-	-	-	-	-	-	-	-	1	-	1	-	1	1	4	
	P	ACACA	31	NM_198838	96.2127	-	-	-	-	-	1	-	1	-	-	1	1	-	-	-	1	1	6	-	-	-	-	-	-	-	-	-	-	-	-	-	-	-	-	-	1	-	1	-	1	1	4	
	P	ACACA	31	NM_198834	96.2127	-	-	-	-	-	1	-	1	-	-	1	1	-	-	-	1	1	6	-	-	-	-	-	-	-	-	-	-	-	-	-	-	-	-	-	1	-	1	-	1	1	4	
	P	ACACB	32	XM_005253876	17.9667	-	-	-	-	-	-	-	-	-	-	1	-	-	-	1	1	-	3	-	-	-	-	-	-	-	-	-	-	-	-	-	-	-	-	-	1	-	-	1	1	-	3	
	P	ACACB	32	NM_001093	17.9667	-	-	-	-	-	-	-	-	-	-	1	-	-	1	-	1	-	3	-	-	-	-	-	-	-	-	-	-	-	-	-	-	-	-	-	1	-	-	-	1	-	2	
	P	ACACB	32	XM_005253877	17.9667	-	-	-	-	-	-	-	-	-	-	1	-	-	-	-	1	-	2	-	-	-	-	-	1	-	-	-	-	-	1	-	-	-	-	-	1	-	-	-	1	-	2	
	P	ECHS1	1892	NM_004092	14.5556	-	-	-	-	-	-	-	-	-	-	1	-	-	1	1	1	-	4	-	-	-	-	-	-	-	-	-	-	-	-	-	-	-	-	-	1	-	-	1	1	-	3	
	P	FAS	355	NM_000043	86.7197	-	-	-	-	-	1	1	1	-	1	1	-	-	-	-	1	1	7	-	-	-	-	-	-	-	-	-	-	-	-	-	-	-	-	-	1	1	1	-	1	1	5	
	P	FAS	355	NM_152871	86.7197	-	-	-	-	-	1	-	1	-	1	1	-	-	-	-	1	1	6	-	-	-	-	-	-	-	-	-	-	-	-	-	-	-	-	-	1	-	1	-	1	1	4	
	P	FAS	355	NM_152872	86.7197	-	-	-	-	-	1	-	1	-	1	1	-	-	-	-	1	1	6	-	-	-	-	-	-	-	-	-	-	-	-	-	-	-	-	-	1	-	1	-	1	1	4	
	V	FASN	2194	NM_004104	12.0728	1	7	7886	7880	0.0493	1	1	-	-	-	1	-	1	1	1	1	1	8	-	-	-	-	-	-	-	-	-	-	-	-	-	-	-	-	-	-	1	-	1	1	1	4	doi:10.1038/s41598-017-15141-x
	P	OXSM	54995	NM_017897	-	-	-	-	-	-	-	-	-	-	-	-	-	-	-	-	-	-	-	-	-	-	-	-	-	-	-	-	1	-	1	-	-	-	-	-	-	-	-	1	1	-	2	
	P	OXSM	54995	NM_001145391	-	-	-	-	-	-	-	-	-	-	-	-	-	-	-	-	-	-	-	-	-	-	-	-	-	-	-	-	1	-	1	-	-	-	-	-	-	-	-	-	1	-	1	
	P	ACSBG1	23205	NM_015162	72.7881	-	-	-	-	-	-	1	-	-	-	-	-	-	-	-	1	-	2	-	-	-	-	-	-	-	-	-	-	-	-	-	-	-	-	-	1	-	-	-	1	-	2	
	P	ACSBG1	23205	NM_001199377	72.7881	-	-	-	-	-	-	-	-	-	-	-	-	-	-	-	1	-	1	-	-	-	-	-	-	-	-	-	-	-	-	-	-	-	-	-	1	-	-	-	1	-	2	

	P	ACSBG2	81616	XM_005259653	-	1	7	1795	1789	0.0059	-	-	-	-	-	-	-	-	-	-	1	1	3	-	-	-	-	-	-	-	-	-	1	-	1	-	-	-	-	-	-	-	-	-	1	-	1	
	P	ACSBG2	81616	XM_005259652	-	-	-	-	-	-	-	-	-	-	-	-	-	-	-	-	1	-	1	-	-	-	-	-	-	-	-	1	1	-	2	-	-	-	-	-	1	-	-	1	1	1	4	
	P	ACSBG2	81616	XM_005259654	-	-	-	-	-	-	-	-	-	-	-	-	-	-	-	-	1	-	1	-	-	-	-	-	-	-	-	-	1	-	1	-	-	-	-	-	1	-	-	-	1	1	3	
	P	ACSBG2	81616	XM_005259651	-	-	-	-	-	-	-	-	-	-	-	-	-	-	-	-	1	-	1	-	-	-	-	-	-	-	-	-	1	-	1	-	-	-	-	-	1	-	-	-	1	1	3	
	P	ACSBG2	81616	XM_005259649	-	-	-	-	-	-	-	-	-	-	-	-	-	-	-	-	1	-	1	-	-	-	-	-	-	-	-	-	1	-	1	-	-	-	-	-	1	-	-	-	1	1	3	
	P	ACSBG2	81616	NM_030924	-	-	-	-	-	-	-	-	-	-	-	-	-	-	-	-	1	-	1	-	-	-	-	-	-	-	-	-	1	-	1	-	-	-	-	-	1	-	-	-	1	1	3	
	P	ACSBG2	81616	XM_005259650	-	-	-	-	-	-	-	-	-	-	-	-	-	-	-	-	1	-	1	-	-	-	-	-	-	-	-	-	1	-	1	-	-	-	-	-	1	-	-	-	1	1	3	
	P	ACSL1	2180	XM_005262831	74.2929	-	-	-	-	-	-	1	-	-	-	1	1	-	-	1	1	-	5	-	-	-	-	-	-	-	-	1	-	-	1	-	-	-	-	-	1	-	-	1	1	-	3	
	P	ACSL1	2180	NM_001995	74.2929	-	-	-	-	-	-	-	-	-	-	1	1	-	1	-	1	-	4	-	-	-	-	-	-	-	-	-	-	-	-	-	-	-	-	-	1	-	-	-	1	-	2	
	P	ACSL1	2180	XM_005262833	74.2929	-	-	-	-	-	-	-	-	-	-	1	1	-	-	-	1	-	3	-	-	-	-	-	-	-	-	-	-	-	-	-	-	-	-	-	1	-	-	-	1	-	2	
	P	ACSL1	2180	XM_005262828	74.2929	-	-	-	-	-	-	-	-	-	-	1	1	-	-	-	1	-	3	-	-	-	-	-	-	-	-	-	-	-	-	-	-	-	-	-	1	-	-	-	1	-	2	
	P	ACSL1	2180	XM_005262832	74.2929	-	-	-	-	-	-	-	-	-	-	1	1	-	-	-	1	-	3	-	-	-	-	-	-	-	-	-	-	-	-	-	-	-	-	-	1	-	-	-	1	-	2	
	P	ACSL1	2180	XM_005262829	74.2929	-	-	-	-	-	-	-	-	-	-	1	1	-	-	-	1	-	3	-	-	-	-	-	-	-	-	-	-	-	-	-	-	-	-	-	1	-	-	-	1	-	2	
	P	ACSL1	2180	XM_005262830	74.2929	-	-	-	-	-	-	-	-	-	-	1	1	-	-	-	1	-	3	-	-	-	-	-	-	-	-	-	-	-	-	-	-	-	-	-	1	-	-	-	1	-	2	
	P	ACSL1	2180	XM_005262827	74.2929	-	-	-	-	-	-	-	-	-	-	1	1	-	-	-	1	-	3	-	-	-	-	-	-	-	-	-	-	-	-	-	-	-	-	-	1	-	-	-	1	-	2	
	P	ACSL3	2181	NM_004457	97.8647	-	-	-	-	-	-	1	-	-	-	-	-	-	-	-	1	-	2	-	-	-	-	-	-	-	-	-	1	-	1	-	-	-	-	-	1	1	-	-	1	1	4	
	P	ACSL3	2181	NM_203372	97.8647	-	-	-	-	-	-	-	-	-	-	-	-	-	-	-	1	-	1	-	-	-	-	-	-	-	-	-	1	-	1	-	-	-	-	-	1	-	-	-	1	1	3	
	P	ACSL4	2182	NM_022977	-	-	-	-	-	-	-	1	-	-	-	-	-	-	-	-	1	-	2	-	-	-	-	-	-	-	-	-	-	-	-	-	-	-	-	-	1	1	-	1	1	1	5	
	P	ACSL4	2182	XM_005262110	-	-	-	-	-	-	-	-	-	-	-	-	-	-	-	-	1	-	1	-	-	-	-	-	-	-	-	-	-	-	-	-	-	-	-	-	1	-	-	-	1	1	3	
	P	ACSL4	2182	NM_004458	-	-	-	-	-	-	-	-	-	-	-	-	-	-	-	-	1	-	1	-	-	-	-	-	-	-	-	-	-	-	-	-	-	-	-	-	1	-	-	-	1	1	3	
	P	ACSL4	2182	XM_005262109	-	-	-	-	-	-	-	-	-	-	-	-	-	-	-	-	1	-	1	-	-	-	-	-	-	-	-	-	-	-	-	-	-	-	-	-	1	-	-	-	1	1	3	
	P	ACSL4	2182	XM_005262108	-	-	-	-	-	-	-	-	-	-	-	-	-	-	-	-	1	-	1	-	-	-	-	-	-	-	-	-	-	-	-	-	-	-	-	-	1	-	-	-	1	1	3	
	P	ACSL5	51703	NM_203380	51.2876	-	-	-	-	-	1	1	-	-	-	1	-	-	-	1	1	1	6	-	-	-	-	-	-	-	-	-	1	-	1	-	-	-	-	-	-	-	-	-	1	-	1	
	P	ACSL5	51703	XM_005269898	51.2876	-	-	-	-	-	1	-	-	-	-	1	-	-	-	-	1	1	4	-	-	-	-	-	-	-	-	-	-	-	-	-	-	-	-	-	-	-	-	-	1	-	1	
	P	ACSL5	51703	XM_005269899	51.2876	-	-	-	-	-	1	-	-	-	-	1	-	-	-	-	1	1	4	-	-	-	-	-	-	-	-	-	-	-	-	-	-	-	-	-	-	-	-	-	1	-	1	
	P	ACSL5	51703	XM_005269897	51.2876	-	-	-	-	-	1	-	-	-	-	1	-	-	-	-	1	1	4	-	-	-	-	-	-	-	-	-	-	-	-	-	-	-	-	-	-	-	-	-	1	-	1	
	P	ACSL5	51703	NM_203379	51.2876	-	-	-	-	-	1	-	-	-	-	1	-	-	-	-	1	1	4	-	-	-	-	-	-	-	-	-	1	-	1	-	-	-	-	-	-	-	-	-	1	-	1	
	P	ACSL5	51703	NM_016234	51.2876	-	-	-	-	-	1	-	-	-	-	1	-	-	-	-	1	1	4	-	-	-	-	-	-	-	-	-	-	-	-	-	-	-	-	-	-	-	-	-	1	-	1	
	P	ACSL6	23305	XM_005271939	-	-	-	-	-	-	1	1	-	-	-	-	-	-	-	-	1	-	3	-	-	-	-	-	-	-	-	-	-	-	-	-	-	-	-	-	-	-	-	1	1	-	2	
	P	ACSL6	23305	XM_005271941	-	-	-	-	-	-	1	-	1	-	-	-	-	-	-	-	1	1	3	-	-	-	-	-	-	-	-	-	-	-	-	-	-	-	-	-	-	-	-	-	1	-	1	
	P	ACSL6	23305	XM_005271940	-	-	-	-	-	-	1	-	-	-	-	-	-	-	-	-	1	-	2	-	-	-	-	-	-	-	-	-	-	-	-	-	-	-	-	-	-	-	-	-	1	-	1	
	P	ACSL6	23305	NM_001205251	-	-	-	-	-	-	1	-	-	-	-	-	-	-	-	-	1	-	2	-	-	-	-	-	-	-	-	-	-	-	-	-	-	-	-	-	-	-	-	-	1	-	1	
	P	ACSL6	23305	NM_001205247	-	-	-	-	-	-	1	-	-	-	-	-	-	-	-	-	1	-	2	-	-	-	-	-	-	-	-	-	-	-	-	-	-	-	-	-	-	-	-	-	1	-	1	
	P	ACSL6	23305	NM_001205248	-	-	-	-	-	-	1	-	-	-	-	-	-	-	-	-	1	-	2	-	-	-	-	-	-	-	-	-	-	-	-	-	-	-	-	-	-	-	-	-	1	-	1	
	P	ACSL6	23305	NM_001205250	-	-	-	-	-	-	1	-	-	-	-	-	-	-	-	-	1	-	2	-	-	-	-	-	-	-	-	-	-	-	-	-	-	-	-	-	-	-	-	-	1	-	1	
	P	ACSL6	23305	NM_001009185	-	-	-	-	-	-	1	-	-	-	-	-	-	-	-	-	1	-	2	-	-	-	-	-	-	-	-	-	-	-	-	-	-	-	-	-	-	-	-	-	1	-	1	
	P	ACSL6	23305	NM_015256	-	-	-	-	-	-	1	-	-	-	-	-	-	-	-	-	1	-	2	-	-	-	-	-	-	-	-	-	-	-	-	-	-	-	-	-	-	-	-	-	1	-	1	
cytochrome c oxidase	P	COX15	1355	NM_078470	76.8855	-	-	-	-	-	-	-	1	-	-	-	-	-	1	-	1	1	5	-	-	-	-	-	-	-	-	-	1	-	1	-	-	-	-	-	-	-	-	-	1	-	1	
	P	COX15	1355	NM_004376	76.8855	-	-	-	-	-	1	-	-	-	-	-	-	-	-	-	1	1	3	-	-	-	-	-	-	-	-	-	1	-	1	-	-	-	-	-	-	-	-	-	1	-	1	
	P	COX15	1355	XM_005269539	76.8855	-	-	-	-	-	-	-	-	-	-	-	-	-	-	-	-	-	-	-	-	-	-	-	-	-	-	-	1	-	1	-	-	-	-	-	-	-	-	-	1	-	1	
	P	COX10	1352	NM_001303	21.5792	-	-	-	-	-	-	-	-	-	-	1	-	-	1	1	1	-	4	-	-	-	-	-	-	-	-	-	-	-	-	-	-	-	-	-	1	-	-	-	1	-	2	
	P	COX10	1352	XM_005256459	21.5792	-	-	-	-	-	-	-	-	-	-	1	-	-	-	-	-	-	1	-	-	-	-	-	-	-	-	-	-	-	-	-	-	-	-	-	-	-	-	-	-	-	-	
	P	COX10	1352	XM_005256458	21.5792	-	-	-	-	-	-	-	-	-	-	1	-	-	-	-	-	-	1	-	-	-	-	-	-	-	-	-	-	-	-	-	-	-	-	-	-	-	-	-	-	-	-	
	P	COX11	1353	NM_004375	53.8338	-	-	-	-	-	1	1	-	-	-	1	-	-	1	1	1	1	7	-	-	-	-	-	-	-	-	-	-	-	-	-	-	-	-	-	-	-	-	-	1	-	1	
	P	COX11	1353	NM_001162862	53.8338	-	-	-	-	-	-	-	-	-	-	1	-	-	-	-	-	-	1	-	-	-	-	-	-	-	-	-	-	-	-	-	-	-	-	-	-	-	-	-	1	-	1	
	P	COX11	1353	NM_001162861	53.8338	-	-	-	-	-	-	-	-	-	-	1	-	-	-	-	-	-	1	-	-	-	-	-	-	-	-	-	-	-	-	-	-	-	-	-	-	-	-	-	1	-	1	
	P	COX5B	1329	NM_001862	-	-	-	-	-	-	-	-	-	-	-	-	-	-	-	-	-	-	-	-	-	-	-	-	-	-	-	-	-	-	-	1	7	311	305	0.0235	1	0	0	0	1	1	3	
	P	COX6C	1345	NM_004374	-	-	-	-	-	-	-	-	1	-	-	-	-	-	-	-	1	1	3	-	-	-	-	-	-	-	-	-	-	-	-	-	-	-	-	-	-	-	-	-	-	-	-	
	P	COX7A2	1347	NM_001865	-	-	-	-	-	-	-	-	-	-	-	-	-	-	-	-	1	-	1	-	-	-	-	-	-	-	-	-	-	-	-	-	-	-	-	-	1	-	-	-	1	-	2	
	P	COX7A2	1347	NM_001865	-	-	-	-	-	-	-	-	-	-	-	-	-	-	-	-	1	-	1	-	-	-	-	-	-	-	-	-	-	-	-	-	-	-	-	-	1	-	-	-	2	-	3	
	P	COX7A2L	9167	NM_004718	-	-	-	-	-	-	-	-	-	-	-	1	-	-	1	-	1	-	3	-	-	-	-	-	-	-	-	-	-	-	-	-	-	-	-	-	-	-	-	-	1	-	1	
	P	COX7A2L	9167	XM_005264636	-	-	-	-	-	-	-	-	-	-	-	1	-	-	-	-	1	-	2	-	-	-	-	-	-	-	-	1	-	-	1	-	-	-	-	-	-	-	-	-	1	-	1	
	P	COX8A	1351	NM_004074	64.1644	-	-	-	-	-	-	-	1	-	-	1	-	-	1	-	1	1	5	-	-	-	-	-	-	-	-	-	1	-	1	-	-	-	-	-	-	-	-	-	-	-	-	
NADH dehydrogenase complex I	P	NDUFA10	4705	NM_004544	50.9791	-	-	-	-	-	1	-	-	-	-	1	-	-	1	1	1	1	6	-	-	-	-	-	-	-	-	-	-	-	-	-	-	-	-	-	-	-	-	1	-	-	1	
	P	NDUFA10	4705	XM_005247008	50.9791	1	7	1393	1387	0.0229	1	-	-	-	-	1	-	-	-	-	1	1	4	-	-	-	-	-	-	-	-	-	-	-	-	-	-	-	-	-	-	-	-	-	-	-	-	
	P	NDUFA10	4705	XM_005247007	50.9791	-	-	-	-	-	1	-	-	-	-	1	-	-	-	-	1	-	3	-	-	-	-	-	-	-	-	-	-	-	-	-	-	-	-	-	-	-	-	-	-	-	-	
	P	NDUFA10	4705	XM_005247006	50.9791	-	-	-	-	-	1	-	-	-	-	1	-	-	-	-	1	-	3	-	-	-	-	-	-	-	-	-	-	-	-	-	-	-	-	-	-	-	-	-	-	-	-	
	P	NDUFA10	4705	XM_005247005	50.9791	-	-	-	-	-	-	-	-	-	-	1	-	-	-	-	-	-	1	-	-	-	-	-	-	-	-	-	-	-	-	-	-	-	-	-	-	-	-	-	-	-	-	
	P	NDUFA11	126328	NM_001193375	23.0813	-	-	-	-	-	-	-	-	-	-	1	-	-	-	-	1	-	2	-	-	-	-	-	-	-	-	-	-	-	-	-	-	-	-	-	1	-	-	-	-	-	1	
	P	NDUFA11	126328	NM_175614	23.0813	-	-	-	-	-	-	-	-	-	-	1	-	-	-	-	-	-	1	-	-	-	-	-	-	-	-	-	-	-	-	-	-	-	-	-	-	-	-	-	-	-	-	
	P	NDUFA4	4697	NM_002489	-	-	-	-	-	-	1	-	-	-	-	-	-	-	-	-	1	-	2	-	-	-	-	-	-	-	-	-	-	-	-	-	-	-	-	-	-	-	-	-	-	-	-	
	P	NDUFA5	4698	NM_005000	86.4294	-	-	-	-	-	1	-	-	-	-	1	-	-	1	-	1	1	5	-	-	-	-	-	-	-	-	-	-	-	-	-	-	-	-	-	-	-	-	-	1	-	1	
	P	NDUFA5	4698	XM_005250371	86.4294	-	-	-	-	-	1	-	-	-	-	1	-	-	-	-	1	1	4	-	-	-	-	-	-	-	-	1	-	-	1	-	-	-	-	-	-	-	-	-	1	-	1	
	P	NDUFA6	4700	NM_002490	-	-	-	-	-	-	-	-	-	-	-	-	-	-	1	-	1	-	2	-	-	-	-	-	-	-	-	-	-	-	-	1	9	310	302	0.0018	1	1	1	1	1	1	6	
	P	NDUFA7	4701	NM_005001	-	-	-	-	-	-	-	-	-	-	-	-	-	-	-	-	-	-	-	-	-	-	-	-	-	-	-	-	-	-	-	-	-	-	-	-	1	-	-	-	1	-	2	
	P	NDUFB11	54539	NM_019056	-	-	-	-	-	-	-	-	-	-	-	-	-	-	-	-	-	-	-	-	-	-	-	-	-	-	-	-	-	-	-	-	-	-	-	-	-	-	-	1	1	-	2	
	P	NDUFB11	54539	NM_001135998	-	-	-	-	-	-	-	-	-	-	-	-	-	-	-	-	-	-	-	-	-	-	-	-	-	-	-	-	-	-	-	-	-	-	-	-	-	-	-	-	1	-	1	
	P	NDUFB4	4710	NM_004547	92.8055	-	-	-	-	-	-	-	-	-	-	1	-	-	-	-	-	-	1	-	-	-	-	-	-	-	-	-	-	-	-	-	-	-	-	-	-	-	-	-	-	-	-	
	P	NDUFB4	4710	NM_001168331	92.8055	-	-	-	-	-	1	-	-	-	-	1	-	-	-	1	-	-	3	-	-	-	-	-	-	-	-	-	-	-	-	-	-	-	-	-	-	-	-	1	-	-	1	
	P	NDUFA5	4711	NM_002492	86.4294	-	-	-	-	-	-	1	-	-	-	-	-	-	-	1	1	-	3	-	-	-	-	-	-	-	-	-	-	-	-	-	-	-	-	-	-	-	-	-	-	-	-	
	P	NDUFA5	4711	NM_001199957	86.4294	-	-	-	-	-	-	-	-	-	-	-	-	-	-	-	1	-	1	-	-	-	-	-	-	-	-	-	-	-	-	-	-	-	-	-	-	-	-	-	-	-	-	
	P	NDUFA5	4711	NM_001199958	86.4294	-	-	-	-	-	-	-	-	-	-	-	-	-	-	-	1	-	1	-	-	-	-	-	-	-	-	-	-	-	-	-	-	-	-	-	-	-	-	-	-	-	-	
	P	NDUFB9	4715	NM_001278645	-	-	-	-	-	-	-	-	-	-	-	-	-	-	-	-	-	-	-	-	-	-	-	-	-	-	-	-	-	-	-	-	-	-	-	-	1	-	-	-	1	-	2	
	P	NDUFB9	4715	NM_001278646	-	-	-	-	-	-	-	-	-	-	-	-	-	-	-	-	-	-	-	-	-	-	-	-	-	-	-	-	-	-	-	-	-	-	-	-	1	-	-	-	1	-	2	
	P	NDUFB9	4715	NM_005005	-	-	-	-	-	-	-	-	-	-	-	-	-	-	-	-	-	-	-	-	-	-	-	-	-	-	-	-	-	-	-	-	-	-	-	-	1	-	-	-	1	-	2	
	P	NDUFC1	4717	NM_001184990	-	-	-	-	-	-	-	-	-	-	-	-	-	-	-	-	1	-	1	-	-	-	-	-	-	-	-	-	-	-	-	-	-	-	-	-	1	-	-	-	1	-	2	
	P	NDUFC1	4717	NM_001184987	-	-	-	-	-	-	-	-	-	-	-	-	-	-	-	-	1	-	1	-	-	-	-	-	-	-	-	-	-	-	-	-	-	-	-	-	1	-	-	-	1	-	2	
	P	NDUFC1	4717	NM_001184988	-	-	-	-	-	-	-	-	-	-	-	-	-	-	-	-	1	-	1	-	-	-	-	-	-	-	-	-	1	-	1	-	-	-	-	-	1	-	-	-	1	-	2	
	P	NDUFC1	4717	NM_001184989	-	-	-	-	-	-	-	-	-	-	-	-	-	-	-	-	1	-	1	-	-	-	-	-	-	-	-	-	-	-	-	-	-	-	-	-	1	-	-	-	1	-	2	
	P	NDUFC1	4717	NM_002494	-	-	-	-	-	-	-	-	-	-	-	-	-	-	-	-	1	-	1	-	-	-	-	-	1	-	-	-	-	-	1	-	-	-	-	-	1	-	-	-	1	-	2	
	P	NDUFC1	4717	NM_001184991	-	-	-	-	-	-	-	-	-	-	-	-	-	-	-	-	1	-	1	-	-	-	-	-	1	-	-	-	1	-	2	-	-	-	-	-	1	-	-	-	1	-	2	
	P	NDUFC1	4717	NM_001184986	-	-	-	-	-	-	-	-	-	-	-	-	-	-	-	1	1	-	2	-	-	-	-	-	1	-	-	-	1	-	2	-	-	-	-	-	1	-	-	-	1	-	2	
	P	NDUFC2	4718	NM_004549	40.1559	-	-	-	-	-	-	-	-	-	-	1	-	-	1	1	1	-	4	-	-	-	-	-	-	-	-	-	1	-	1	-	-	-	-	-	-	-	-	1	1	-	2	
	P	NDUFC2	4718	NM_001204054	40.1559	-	-	-	-	-	-	-	-	-	-	1	-	-	-	-	1	-	2	-	-	-	-	-	-	-	-	-	1	-	1	-	-	-	-	-	-	-	-	-	-	-	-	
	P	NDUFC2	4718	NM_001204055	40.1559	-	-	-	-	-	-	-	-	-	-	1	-	-	-	-	1	-	2	-	-	-	-	-	-	-	-	-	1	-	1	-	-	-	-	-	-	-	-	-	1	-	1	
	P	NDUFS1	4719	NM_005006	-	-	-	-	-	-	-	1	-	-	-	-	-	-	1	-	1	-	3	-	-	-	-	-	-	-	-	-	-	-	-	-	-	-	-	-	1	-	-	-	1	-	2	
	P	NDUFS1	4719	NM_001199982	-	-	-	-	-	-	-	-	-	-	-	-	-	-	-	-	1	-	1	-	-	-	-	-	-	-	-	-	-	-	-	-	-	-	-	-	1	-	-	-	1	-	2	
	P	NDUFS1	4719	NM_001199981	-	-	-	-	-	-	-	-	-	-	-	-	-	-	-	-	1	-	1	-	-	-	-	-	-	-	-	-	-	-	-	-	-	-	-	-	1	-	-	-	1	-	2	
	P	NDUFS1	4719	NM_001199983	-	-	-	-	-	-	-	-	-	-	-	-	-	-	-	-	1	-	1	-	-	-	-	-	-	-	-	-	1	-	1	-	-	-	-	-	1	-	-	-	1	-	2	
	P	NDUFS1	4719	NM_001199984	-	-	-	-	-	-	-	-	-	-	-	-	-	-	-	-	1	-	1	-	-	-	-	-	-	-	-	-	-	-	-	-	-	-	-	-	1	-	-	-	1	-	2	
	P	NDUFS2	4720	NM_001166159	-	-	-	-	-	-	-	-	-	-	-	-	1	-	-	-	1	-	2	-	-	-	-	-	1	-	-	-	1	-	2	-	-	-	-	-	-	-	-	1	1	-	2	
	P	NDUFS2	4720	XM_005245209	-	-	-	-	-	-	-	-	-	-	-	-	1	-	-	-	1	-	2	-	-	-	-	-	-	-	-	-	1	-	1	-	-	-	-	-	-	-	-	-	1	-	1	
	P	NDUFS2	4720	XM_005245208	-	-	-	-	-	-	-	-	-	-	-	-	1	-	-	-	1	-	2	-	-	-	-	-	1	-	-	-	1	-	2	-	-	-	-	-	-	-	-	-	1	-	1	
	P	NDUFS2	4720	NM_004550	-	-	-	-	-	-	-	-	-	-	-	-	1	-	-	-	1	-	2	-	-	-	-	-	1	-	-	-	1	-	2	-	-	-	-	-	-	-	-	-	1	-	1	
	P	NDUFS3	4722	NM_004551	-	-	-	-	-	-	-	-	-	-	-	-	-	-	-	1	-	-	1	-	-	-	-	-	-	-	-	-	-	-	-	-	-	-	-	-	1	-	-	1	1	-	3	
	V	NDUFS4	4724	NM_002495	87.1064	1	8	590	583	0.0018	1	-	1	-	1	1	-	1	1	1	1	1	9	-	-	-	-	-	-	-	-	-	-	-	-	-	-	-	-	-	-	-	-	-	1	-	1	doi.org/10.1016/j.bbadis.2014.11.019
	V	NDUFS4	4724	XM_005248525	87.1064	1	8	517	510	0.0032	1	-	1	-	-	1	-	-	-	-	1	1	5	-	-	-	-	-	-	-	-	-	-	-	-	-	-	-	-	-	-	-	-	-	1	-	1	
	P	NDUFS6	4726	NM_004553	19.408	-	-	-	-	-	-	-	-	-	-	-	-	-	1	-	1	-	2	-	-	-	-	-	-	-	-	-	-	-	-	-	-	-	-	-	-	-	-	-	1	-	1	
	P	NDUFV1	4723	NM_007103	-	-	-	-	-	-	-	-	-	-	-	-	-	-	-	-	-	-	-	-	-	-	-	-	-	-	-	-	-	-	-	-	-	-	-	-	1	-	-	1	1	-	3	
	P	NDUFV1	4723	NM_001166102	-	-	-	-	-	-	-	-	-	-	-	-	-	-	-	-	-	-	-	-	-	-	-	-	-	-	-	-	-	-	-	-	-	-	-	-	1	-	-	-	1	-	2	
	P	NDUFV2	4729	NM_021074	-	-	-	-	-	-	-	-	-	-	-	-	-	-	-	-	-	-	-	-	-	-	-	-	-	-	-	-	-	-	-	-	-	-	-	-	-	-	-	-	1	-	1	
	P	NDUFV2	4729	XM_005258106	-	-	-	-	-	-	-	-	-	-	-	-	-	-	-	-	-	-	-	-	-	-	-	-	-	-	-	-	-	-	-	-	-	-	-	-	-	-	-	-	1	-	1	
	P	NDUFV3	4731	NM_021075	-	-	-	-	-	-	1	-	-	-	-	-	-	-	-	-	-	-	1	-	-	-	-	-	-	-	-	-	1	-	1	-	-	-	-	-	1	-	-	-	1	1	3	
	P	NDUFV3	4731	NM_001001503	-	-	-	-	-	-	1	-	-	-	-	-	-	-	-	-	-	-	1	-	-	-	-	-	-	-	-	-	1	-	1	-	-	-	-	-	-	-	-	-	-	-	-	
succinate dehydrogenase/fumarate reductase	P	SDHA	6389	NM_004168	-	-	-	-	-	-	-	-	-	-	-	-	-	-	-	-	1	-	1	-	-	-	-	-	-	-	-	-	-	-	-	-	-	-	-	-	1	-	-	-	1	-	2	
	P	SDHA	6389	XM_005248331	-	-	-	-	-	-	-	-	-	-	-	-	-	-	-	-	1	-	1	-	-	-	-	-	-	-	-	-	-	-	-	-	-	-	-	-	1	-	-	-	1	-	2	
	P	SDHA	6389	XM_005248330	-	-	-	-	-	-	-	-	-	-	-	-	-	-	-	-	1	-	1	-	-	-	-	-	-	-	-	-	-	-	-	-	-	-	-	-	1	-	-	-	1	-	2	
	P	SDHA	6389	XM_005248329	-	-	-	-	-	-	-	-	-	-	-	-	-	-	-	-	-	-	-	-	-	-	-	-	-	-	-	-	-	-	-	-	-	-	-	-	1	-	1	-	1	1	4	
	P	SDHC	6391	NM_003001	-	-	-	-	-	-	1	-	-	-	-	-	-	-	-	-	1	-	2	-	-	-	-	-	-	-	-	-	-	-	-	-	-	-	-	-	-	-	-	-	1	-	1	
	P	SDHC	6391	NM_001278172	-	-	-	-	-	-	1	-	-	-	-	-	-	-	-	-	1	-	2	-	-	-	-	-	-	-	-	-	-	-	-	-	-	-	-	-	-	-	-	-	-	-	-	
	P	SDHC	6391	NM_001035511	-	-	-	-	-	-	1	-	-	-	-	-	-	-	-	-	1	-	2	-	-	-	-	-	-	-	-	-	-	-	-	-	-	-	-	-	-	-	-	-	-	-	-	
	P	SDHC	6391	NM_001035513	-	-	-	-	-	-	1	-	-	-	-	-	-	-	-	-	1	-	2	-	-	-	-	-	-	-	-	-	-	-	-	-	-	-	-	-	-	-	-	-	1	-	1	
	P	SDHC	6391	NM_001035512	-	-	-	-	-	-	1	-	-	-	-	-	-	-	-	-	1	-	2	-	-	-	-	-	-	-	-	-	-	-	-	-	-	-	-	-	-	-	-	-	1	-	1	
	P	SDHD	6392	NM_001276506	-	-	-	-	-	-	-	1	-	-	-	1	-	-	-	1	1	-	4	-	-	-	-	-	-	-	-	-	-	-	-	-	-	-	-	-	-	-	-	1	-	-	1	
	P	SDHD	6392	NM_001276503	-	-	-	-	-	-	-	-	-	-	-	1	-	-	-	-	1	-	2	-	-	-	-	-	-	-	-	-	-	-	-	-	-	-	-	-	-	-	-	-	-	-	-	
	P	SDHD	6392	NM_001276504	-	-	-	-	-	-	-	-	-	-	-	1	-	-	-	-	1	-	2	-	-	-	-	-	-	-	-	-	-	-	-	-	-	-	-	-	-	-	-	-	1	-	1	
	P	SDHD	6392	NM_003002	-	-	-	-	-	-	-	-	-	-	-	1	-	-	-	-	1	-	2	-	-	-	-	-	-	-	-	-	-	-	-	-	-	-	-	-	-	-	-	-	1	-	1	
	P	SDHD	6392	XM_005271644	-	-	-	-	-	-	-	-	-	-	-	1	-	-	-	-	-	-	1	-	-	-	-	-	-	-	-	-	-	-	-	-	-	-	-	-	-	-	-	-	-	-	-	
	P	ATP5A1	498	NM_001257334	-	-	-	-	-	-	-	-	-	-	-	-	-	-	-	-	1	-	1	-	-	-	-	-	-	-	-	-	-	-	-	-	-	-	-	-	1	-	-	-	1	-	2	
	P	ATP5A1	498	XM_005258282	-	-	-	-	-	-	-	-	-	-	-	-	-	-	-	-	1	-	1	-	-	-	-	-	-	-	-	-	-	-	-	-	-	-	-	-	1	-	-	-	1	-	2	
	P	ATP5A1	498	NM_004046	-	-	-	-	-	-	-	-	-	-	-	-	-	-	-	-	1	-	1	-	-	-	-	-	-	-	-	-	-	-	-	-	-	-	-	-	1	-	-	-	1	-	2	

ATP synthase complex V	P	*ATP5A1*	498	NM_001001937	-	-	-	-	-	-	-	-	-	-	-	-	-	-	-	-	1	-	1	-	-	-	-	-	-	-	-	-	-	-	-	-	-	-	-	-	-	1	-	-	-	1	-	2	
	P	*ATP5A1*	498	NM_001001935	-	-	-	-	-	-	-	-	-	-	-	-	-	-	-	-	1	-	1	-	-	-	-	-	-	-	-	-	1	-	1	-	-	-	-	-	1	-	-	-	1	-	2	
	P	*ATP5A1*	498	NM_001257335	-	-	-	-	-	-	-	-	-	-	-	-	-	-	-	1	1	-	2	-	-	-	-	-	-	-	-	1	1	-	2	-	-	-	-	-	1	-	-	1	1	-	3	
	P	*ATP5B*	506	NM_001686	-	-	-	-	-	-	-	-	-	-	-	-	-	-	-	-	-	-	-	-	-	-	-	-	-	-	-	-	-	-	-	-	-	-	-	-	1	0	0	1	1	0	3	
	P	*ATP5G2*	517	NM_001002031	-	-	-	-	-	-	-	1	-	-	-	-	-	-	-	1	-	-	2	-	-	-	-	-	-	-	-	-	-	-	-	-	-	-	-	-	-	-	-	1	-	-	1	
	V	*ATP5G3*	518	NM_001190329	92.3095	-	-	-	-	-	1	1	1	-	-	-	-	-	-	-	1	1	5	-	-	-	-	-	-	-	-	-	-	-	-	-	-	-	-	-	-	-	-	-	-	-	-	
	V	*ATP5G3*	518	NM_001689	92.3095	-	-	-	-	-	1	-	1	-	-	-	-	-	-	-	1	1	4	-	-	-	-	-	-	-	-	-	-	-	-	-	-	-	-	-	-	-	-	-	-	-	-	***doi.org/10.1016/j.bbadis.2014.11.019***
	V	*ATP5G3*	518	NM_001002258	92.3095	-	-	-	-	-	1	-	1	-	-	-	-	-	-	-	1	1	4	-	-	-	-	-	-	-	-	-	-	-	-	-	-	-	-	-	-	-	-	-	-	-	-	
	P	*ATP5L*	10632	NM_006476	-	-	-	-	-	-	-	-	-	-	-	-	-	-	-	1	-	-	1	-	-	-	-	-	-	-	-	-	-	-	-	-	-	-	-	-	1	-	-	-	1	-	2	
	P	*ATP5L2*	267020	NM_001165877	-	-	-	-	-	-	-	-	-	-	-	-	-	-	-	-	-	-	-					-	1	-	-	-	-	-	1	-	-	-	-	-	1	-	-	-	1	-	2	
	P	*ATP5S*	27109	XM_005267538	-	-	-	-	-	-	-	-	-	-	-	-	-	-	-	-	-	-	-	-	-	-	-	-	-	-	-	-	-	-	-	-	-	-	-	-	-	-	-	1	1	-	2	
	P	*ATP5S*	27109	XM_005267535	-	-	-	-	-	-	-	-	-	-	-	-	-	-	-	-	-	-	-	-	-	-	-	-	-	-	-	-	-	-	-	-	-	-	-	-	-	-	-	-	1	-	1	
	P	*ATP5S*	27109	XM_005267539	-	-	-	-	-	-	-	-	-	-	-	-	-	-	-	-	-	-	-	-	-	-	-	-	-	-	-	-	1	-	1	-	-	-	-	-	-	-	-	-	1	-	1	
	P	*ATP5S*	27109	NM_015684	-	-	-	-	-	-	-	-	-	-	-	-	-	-	-	-	-	-	-	-	-	-	-	-	-	-	-	-	1	-	1	-	-	-	-	-	-	-	-	-	1	-	1	
	P	*ATP5S*	27109	XM_005267537	-	-	-	-	-	-	-	-	-	-	-	-	-	-	-	-	-	-	-	-	-	-	-	-	-	-	-	-	-	-	-	-	-	-	-	-	-	-	-	-	1	-	1	
	P	*ATP5S*	27109	NM_001003805	-	-	-	-	-	-	-	-	-	-	-	-	-	-	-	-	-	-	-	-	-	-	-	-	-	-	-	-	1	-	1	-	-	-	-	-	-	-	-	-	1	-	1	
	P	*ATP5S*	27109	NM_001003803	-	-	-	-	-	-	-	-	-	-	-	-	-	-	-	-	-	-	-	-	-	-	-	-	-	-	-	-	1	-	1	-	-	-	-	-	-	-	-	-	1	-	1	
	P	*ATP5SL*	55101	NM_001167867	-	-	-	-	-	-	-	-	-	-	-	-	-	-	-	-	-	-	-	-	-	-	-	-	-	-	-	-	-	-	-	1	7	454	448	0.0472	1	-	1	1	1	1	5	
	P	*ATP5SL*	55101	NM_001167870	-	-	-	-	-	-	-	-	-	-	-	-	-	-	-	-	-	-	-	-	-	-	-	-	-	-	-	-	-	-	-	1	7	327	321	0.0287	1	-	1	-	1	1	4	
	P	*ATP5SL*	55101	NM_001167869	-	-	-	-	-	-	-	-	-	-	-	-	-	-	-	-	-	-	-	-	-	-	-	-	-	-	-	-	-	-	-	1	7	408	402	0.0335	1	-	1	-	1	1	4	
	P	*ATP5SL*	55101	NM_001167868	-	-	-	-	-	-	-	-	-	-	-	-	-	-	-	-	-	-	-	-	-	-	-	-	-	-	-	-	-	-	-	1	7	454	448	0.0345	1	-	1	-	1	1	4	
	P	*ATP5SL*	55101	NM_001167871	-	-	-	-	-	-	-	-	-	-	-	-	-	-	-	-	-	-	-	-	-	-	-	-	-	-	-	-	-	-	-	1	7	327	321	0.0414	1	-	1	-	1	1	4	
	P	*ATP5SL*	55101	NM_018035	-	-	-	-	-	-	-	-	-	-	-	-	-	-	-	-	-	-	-	-	-	-	-	-	-	-	-	-	-	-	-	1	7	408	402	0.0461	1	-	1	-	1	1	4	
ubiquinol-cytochrome c reductase	P	*UQCRC1*	7384	XM_005265436	-	-	-	-	-	-	-	-	-	-	-	-	-	-	-	-	1	-	1	-	-	-	-	-	-	-	-	-	1	-	1	-	-	-	-	-	-	-	-	1	1	-	2	
	P	*UQCRC1*	7384	NM_003365	-	-	-	-	-	-	-	-	-	-	-	-	-	-	-	-	1	-	1	-	-	-	-	-	-	-	-	-	-	-	-	-	-	-	-	-	-	-	-	-	1	-	1	
	P	*UQCR10*	29796	NM_001003684	-	-	-	-	-	-	-	-	-	-	-	-	-	-	-	-	-	-	-	1	8	25	18	0.0005	1	1	-	-	1	-	3	-	-	-	-	-	-	-	-	1	-	-	1	
	P	*UQCR10*	29796	NM_013387	-	-	-	-	-	-	-	-	-	-	-	-	-	-	-	-	-	-	-	1	8	25	18	0.0005	1	1	-	-	1	-	3	-	-	-	-	-	-	-	-	-	1	-	1	
	P	*UQCR11*	10975	NM_006830	64.0243	-	-	-	-	-	-	-	1	-	-	1	-	-	1	-	1	1	5	-	-	-	-	-	-	-	-	-	-	-	-	-	-	-	-	-	-	-	-	-	1	-	1	
	P	*UQCRH*	7388	XM_005271167	-	-	-	-	-	-	-	-	-	-	-	-	-	-	-	-	-	-	-	-	-	-	-	-	-	-	-	-	-	-	-	-	-	-	-	-	-	-	-	1	1	-	2	
	P	*UQCRH*	7388	NM_006004	-	-	-	-	-	-	-	-	-	-	-	-	-	-	-	-	-	-	-	-	-	-	-	-	-	-	-	-	-	-	-	-	-	-	-	-	-	-	-	-	1	-	1	
	P	*UQCRHL*	440567	NM_001089591	-	-	-	-	-	-	-	-	-	-	-	-	-	-	1	-	1	-	2	-	-	-	-	-	-	-	-	-	-	-	-	-	-	-	-	-	-	-	-	-	-	-	-	
ATP synthase F0 subunit 8	P	*ATP8A1*	10396	NM_006095	-	-	-	-	-	-	-	1	-	-	-	-	-	-	-	1	1	-	3	-	-	-	-	-	-	-	-	-	-	-	-	-	-	-	-	-	-	-	-	-	1	-	1	
	P	*ATP8A1*	10396	XM_005248043	-	-	-	-	-	-	-	1	-	-	-	-	-	-	-	1	1	-	3	-	-	-	-	-	-	-	-	-	-	-	-	-	-	-	-	-	-	-	-	-	1	-	1	
	P	*ATP8A1*	10396	NM_001105529	-	-	-	-	-	-	-	-	-	-	-	-	-	-	-	-	1	-	1	-	-	-	-	-	-	-	-	-	-	-	-	-	-	-	-	-	-	-	-	-	1	-	1	
	P	*ATP8A2*	51761	XM_005266419	30.243	-	-	-	-	-	1	1	-	-	-	1	-	-	-	-	1	1	5	-	-	-	-	-	-	-	-	-	-	-	-	-	-	-	-	-	-	-	-	1	1	-	2	
	P	*ATP8A2*	51761	XM_005266420	30.243	-	-	-	-	-	1	-	-	-	-	1	-	-	-	-	1	1	4	-	-	-	-	-	-	-	-	-	-	-	-	-	-	-	-	-	-	-	-	-	1	-	1	
	P	*ATP8A2*	51761	NM_016529	30.243	-	-	-	-	-	-	-	-	-	-	1	-	-	1	-	1	-	3	-	-	-	-	-	-	-	-	-	-	-	-	-	-	-	-	-	-	-	-	-	1	-	1	
	P	*ATP8B1*	5205	NM_005603	55.4333	-	-	-	-	-	1	1	-	-	-	1	-	-	1	-	1	1	6	-	-	-	-	-	-	-	-	-	-	-	-	-	-	-	-	-	-	-	-	1	1	-	2	
	P	*ATP8B2*	57198	NM_020452	76.6048	-	-	-	-	-	-	-	1	-	1	1	-	-	1	-	1	1	6	-	-	-	-	-	-	-	-	-	-	-	-	-	-	-	-	-	1	0	0	0	1	1	3	
	P	*ATP8B2*	57198	XM_005245355	76.6048	-	-	-	-	-	-	-	1	-	-	1	-	-	-	1	1	1	5	-	-	-	-	-	-	-	-	-	-	-	-	-	-	-	-	-	1	1	0	1	1	1	5	
	P	*ATP8B2*	57198	XM_005245356	76.6048	-	-	-	-	-	-	-	1	-	-	1	-	-	-	-	1	1	4	-	-	-	-	-	-	-	-	-	-	-	-	-	-	-	-	-	1	0	0	0	1	1	3	
	P	*ATP8B2*	57198	NM_001005855	76.6048	-	-	-	-	-	-	-	-	-	-	1	-	-	-	-	1	-	2	-	-	-	-	-	-	-	-	-	-	-	-	-	-	-	-	-	1	0	0	0	1	1	3	
	P	*ATP8B3*	148229	NM_138813	-	-	-	-	-	-	-	-	-	-	-	-	-	-	-	-	1	-	1	-	-	-	-	-	-	-	-	-	-	-	-	-	-	-	-	-	1	0	0	1	1	1	4	
	P	*ATP8B3*	148229	NM_001178002	-	-	-	-	-	-	-	-	-	-	-	-	-	-	-	-	1	-	1	-	-	-	-	-	-	-	-	-	-	-	-	-	-	-	-	-	1	0	0	0	1	1	3	
	P	*ATP8B4*	79895	NM_024837	-	-	-	-	-	-	-	1	-	-	-	1	-	-	-	-	1	-	3	-	-	-	-	-	-	-	-	-	-	-	-	-	-	-	-	-	1	0	1	1	1	1	5	
ATPase H+	P	*ATP6V0A1*	535	NM_001130020	15.189	-	-	-	-	-	1	-	1	-	-	1	-	-	-	1	1	1	6	-	-	-	-	-	-	-	-	-	1	-	1	-	-	-	-	-	1	1	-	1	1	1	5	
	P	*ATP6V0A1*	535	NM_005177	15.189	-	-	-	-	-	1	-	1	-	-	1	-	-	1	-	1	1	6	-	-	-	-	-	-	-	-	-	1	-	1	-	-	-	-	-	1	-	-	-	1	1	3	
	P	*ATP6V0A1*	535	XM_005257462	15.189	-	-	-	-	-	1	-	1	-	-	1	-	-	-	-	1	1	5	-	-	-	-	-	-	-	-	-	1	-	1	-	-	-	-	-	1	-	-	-	1	1	3	
	P	*ATP6V0A1*	535	XM_005257460	15.189	-	-	-	-	-	1	-	1	-	-	1	-	-	-	-	1	1	5	-	-	-	-	-	-	-	-	-	1	-	1	-	-	-	-	-	1	-	-	-	1	1	3	
	P	*ATP6V0A1*	535	XM_005257463	15.189	-	-	-	-	-	1	-	1	-	-	1	-	-	-	-	1	1	5	-	-	-	-	-	-	-	-	-	1	-	1	-	-	-	-	-	1	-	-	-	1	1	3	
	P	*ATP6V0A1*	535	XM_005257461	15.189	-	-	-	-	-	1	-	1	-	-	1	-	-	-	-	1	1	5	-	-	-	-	-	-	-	-	-	1	-	1	-	-	-	-	-	1	-	-	-	1	1	3	
	P	*ATP6V0A1*	535	XM_005257459	15.189	-	-	-	-	-	1	-	1	-	-	1	-	-	-	-	1	1	5	-	-	-	-	-	-	-	-	-	1	-	1	-	-	-	-	-	1	-	-	-	1	1	3	
	P	*ATP6V0A1*	535	NM_001130021	15.189	-	-	-	-	-	1	-	1	-	-	1	-	-	-	-	1	1	5	-	-	-	-	-	-	-	-	-	1	-	1	-	-	-	-	-	1	-	-	-	1	1	3	
	P	*ATP6V0A2*	23545	NM_012463	-	-	-	-	-	-	-	-	-	-	-	-	-	-	1	1	1	-	3	-	-	-	-	-	-	-	-	-	1	-	1	-	-	-	-	-	1	-	-	1	1	-	3	
	P	*ATP6V0A2*	23545	XM_005253564	-	-	-	-	-	-	-	-	-	-	-	-	-	-	-	-	1	-	1	-	-	-	-	-	-	-	-	-	-	-	-	-	-	-	-	-	1	-	-	-	1	-	2	
	P	*ATP6V0A2*	23545	XM_005253563	-	-	-	-	-	-	-	-	-	-	-	-	-	-	-	-	1	-	1	-	-	-	-	-	-	-	-	-	-	-	-	-	-	-	-	-	-	-	-	-	1	-	1	
	P	*ATP6V0A4*	50617	XM_005250393	-	-	-	-	-	-	-	-	-	-	-	-	-	-	-	-	1	-	1	-	-	-	-	-	-	-	-	-	1	-	1	-	-	-	-	-	1	-	-	1	1	-	3	
	P	*ATP6V0A4*	50617	NM_130840	-	-	-	-	-	-	-	-	-	-	-	-	-	-	-	-	1	-	1	-	-	-	-	-	-	-	-	-	-	-	-	-	-	-	-	-	1	-	-	-	1	-	2	
	P	*ATP6V0A4*	50617	XM_005250394	-	-	-	-	-	-	-	-	-	-	-	-	-	-	-	-	1	-	1	-	-	-	-	-	-	-	-	-	-	-	-	-	-	-	-	-	1	-	-	-	1	-	2	
	P	*ATP6V0A4*	50617	NM_130841	-	-	-	-	-	-	-	-	-	-	-	-	-	-	-	-	1	-	1	-	-	-	-	-	-	-	-	-	-	-	-	-	-	-	-	-	1	-	-	-	1	-	2	
	P	*ATP6V0A4*	50617	NM_020632	-	-	-	-	-	-	-	-	-	-	-	-	-	-	-	-	1	-	1	-	-	-	-	-	-	-	-	-	-	-	-	-	-	-	-	-	1	-	-	-	1	-	2	
	P	*ATP6V0B*	533	XM_005270944	-	-	-	-	-	-	-	-	-	-	-	-	-	-	-	1	1	-	2	-	-	-	-	-	1	-	-	-	-	-	1	-	-	-	-	-	-	-	-	-	1	-	1	
	P	*ATP6V0B*	533	NM_001039457	-	-	-	-	-	-	-	-	-	-	-	-	-	-	-	-	1	-	1	-	-	-	-	-	-	-	-	-	-	-	-	-	-	-	-	-	-	-	-	-	1	-	1	
	P	*ATP6V0B*	533	NM_004047	-	-	-	-	-	-	-	-	-	-	-	-	-	-	-	-	1	-	1	-	-	-	-	-	-	-	-	-	-	-	-	-	-	-	-	-	-	-	-	-	1	-	1	
	P	*ATP6V0D1*	9114	NM_004691	11.2057	-	-	-	-	-	-	-	-	-	-	1	-	-	1	-	1	-	3	-	-	-	-	-	-	-	-	-	-	-	-	-	-	-	-	-	1	-	-	1	1	-	3	
	P	*ATP6V0D1*	9114	XM_005256231	11.2057	-	-	-	-	-	-	-	-	-	-	1	-	-	-	-	1	-	2	-	-	-	-	-	-	-	-	-	-	-	-	-	-	-	-	-	1	-	-	-	1	-	2	
	P	*ATP6V0D1*	9114	XM_005256232	11.2057	-	-	-	-	-	-	-	-	-	-	1	-	-	-	-	1	-	2	-	-	-	-	-	-	-	-	-	1	-	1	-	-	-	-	-	1	-	-	-	1	-	2	
	P	*ATP6V0D2*	245972	NM_152565	-	-	-	-	-	-	1	-	-	-	-	-	-	-	-	-	-	-	1	-	-	-	-	-	-	-	-	-	-	-	-	-	-	-	-	-	1	1	-	1	1	1	5	
	P	*ATP6V0E1*	8992	NM_003945	-	-	-	-	-	-	-	-	-	-	-	-	-	-	-	-	1	-	1	-	-	-	-	-	-	-	-	-	-	-	-	-	-	-	-	-	1	-	-	-	1	-	2	
	P	*ATP6V0E2*	155066	NM_145230	-	-	-	-	-	-	1	1	-	-	-	-	-	-	1	1	1	-	5	-	-	-	-	-	1	-	-	-	-	-	1	-	-	-	-	-	-	-	-	1	1	-	2	
	P	*ATP6V0E2*	155066	NM_001100592	-	-	-	-	-	-	-	-	-	-	-	-	-	-	-	-	-	-	-	-	-	-	-	-	1	-	-	-	-	-	1	-	-	-	-	-	1	-	-	-	1	-	2	
	P	*ATP6V0E2*	155066	XM_005249958	-	-	-	-	-	-	-	-	-	-	-	-	-	-	-	-	-	-	-	-	-	-	-	-	1	-	-	-	-	-	1	-	-	-	-	-	1	-	-	-	1	-	2	
	P	*ATP6V1A*	523	XM_005247528	94.8646	-	-	-	-	-	1	1	1	-	-	1	-	-	-	1	1	1	7	-	-	-	-	-	-	-	-	-	1	-	1	-	-	-	-	-	-	-	-	-	1	-	1	
	P	*ATP6V1A*	523	NM_001690	94.8646	-	-	-	-	-	1	-	1	-	-	1	-	1	1	-	1	1	7	-	-	-	-	-	-	-	-	-	-	-	-	-	-	-	-	-	-	-	-	-	1	-	1	
	P	*ATP6V1B1*	525	XM_005264368	-	-	-	-	-	-	-	-	-	-	-	-	-	-	-	-	-	-	-	-	-	-	-	-	1	-	-	-	1	-	2	-	-	-	-	-	-	-	-	-	-	-	-	
	P	*ATP6V1B1*	525	XM_005278323	-	-	-	-	-	-	-	-	-	-	-	-	-	-	-	-	-	-	-	-	-	-	-	-	1	-	-	-	1	-	2	-	-	-	-	-	-	-	-	-	-	-	-	
	P	*ATP6V1B2*	526	NM_001693	-	-	-	-	-	-	-	1	-	-	-	-	-	-	-	-	1	-	2	-	-	-	-	-	-	-	-	1	-	-	1	-	-	-	-	-	-	-	-	1	1	-	2	
	P	*ATP6V1C1*	528	NM_001695	35.1562	-	-	-	-	-	1	1	1	-	-	-	-	-	1	1	1	1	7	-	-	-	-	-	-	-	-	-	-	-	-	-	-	-	-	-	-	-	-	-	1	-	1	
	P	*ATP6V1C1*	528	XM_005250975	35.1562	-	-	-	-	-	1	-	1	-	-	-	-	-	-	-	1	1	4	-	-	-	-	-	-	-	-	-	-	-	-	-	-	-	-	-	-	-	-	-	1	-	1	
	P	*ATP6V1C1*	528	XM_005250974	35.1562	-	-	-	-	-	1	-	1	-	-	-	-	-	-	-	1	1	4	-	-	-	-	-	-	-	-	-	-	-	-	-	-	-	-	-	-	-	-	-	1	-	1	
	P	*ATP6V1C2*	245973	NM_001039362	-	-	-	-	-	-	1	-	-	-	-	-	-	-	-	-	1	-	2	-	-	-	-	-	-	-	-	-	-	-	-	-	-	-	-	-	1	-	1	1	1	1	5	
	P	*ATP6V1C2*	245973	NM_144583	-	-	-	-	-	-	1	-	-	-	-	-	-	-	-	-	1	-	2	-	-	-	-	-	-	-	-	-	-	-	-	-	-	-	-	-	1	-	1	-	1	1	4	
	P	*ATP6V1D*	51382	NM_015994	-	-	-	-	-	-	-	1	-	-	-	-	-	-	1	-	1	-	3	-	-	-	-	-	1	-	-	-	1	-	2	-	-	-	-	-	-	-	-	-	-	-	-	
	P	*ATP6V1E1*	529	NM_001696	41.0132	-	-	-	-	-	-	-	-	-	-	1	-	-	-	-	1	-	2	-	-	-	-	-	-	-	-	-	-	-	-	-	-	-	-	-	-	-	-	-	-	-	-	
	P	*ATP6V1E1*	529	NM_001039367	41.0132	-	-	-	-	-	-	-	-	-	-	1	-	-	-	-	1	-	2	-	-	-	-	-	-	-	-	-	-	-	-	-	-	-	-	-	-	-	-	-	-	-	-	
	P	*ATP6V1E1*	529	NM_001039366	41.0132	-	-	-	-	-	-	-	-	-	-	1	-	-	-	-	1	-	2	-	-	-	-	-	-	-	-	-	-	-	-	-	-	-	-	-	-	-	-	-	-	-	-	
	P	*ATP6V1E2*	90423	XM_005264633	-	-	-	-	-	-	-	-	-	-	-	-	-	-	-	-	-	-	-	-	-	-	-	-	1	-	-	1	1	-	3	1	7	1966	1960	0.0407	1	-	-	1	1	1	4	
	P	*ATP6V1E2*	90423	XM_005264634	-	-	-	-	-	-	-	-	-	-	-	-	-	-	-	-	-	-	-	-	-	-	-	-	1	-	-	-	1	-	2	1	7	1859	1853	0.0407	1	-	-	-	1	1	3	
	P	*ATP6V1E2*	90423	NM_080653	-	-	-	-	-	-	-	-	-	-	-	-	-	-	-	-	-	-	-	-	-	-	-	-	-	-	-	-	1	-	1	1	7	1641	1635	0.0407	1	-	-	-	1	1	3	
	P	*ATP6V1G2*	534	NM_130463	96.9377	-	-	-	-	-	1	1	-	-	-	1	-	-	-	1	1	1	6	-	-	-	-	-	-	-	-	-	-	-	-	-	-	-	-	-	-	-	-	-	-	-	-	
	P	*ATP6V1G2*	534	NM_138282	96.9377	-	-	-	-	-	1	-	-	-	-	1	-	-	-	-	1	1	4	-	-	-	-	-	-	-	-	-	-	-	-	-	-	-	-	-	-	-	-	-	-	-	-	
	P	*ATP6V1G2*	534	NM_001204078	96.9377	-	-	-	-	-	1	-	-	-	-	1	-	-	-	-	1	1	4	-	-	-	-	-	-	-	-	-	-	-	-	-	-	-	-	-	-	-	-	-	-	-	-	
	P	*ATP6V1H*	51606	XM_005251258	-	-	-	-	-	-	-	-	-	-	-	-	1	-	-	1	-	-	2	-	-	-	-	-	-	-	-	1	1	-	2	-	-	-	-	-	-	-	-	1	1	-	2	
	P	*ATP6V1H*	51606	NM_213620	-	-	-	-	-	-	-	-	-	-	-	-	1	-	-	-	-	-	1	-	-	-	-	-	-	-	-	-	-	-	-	-	-	-	-	-	-	-	-	-	1	-	1	
	P	*ATP6V1H*	51606	XM_005251259	-	-	-	-	-	-	-	-	-	-	-	-	1	-	-	-	-	-	1	1	7	133	127	0.0208	1	-	-	-	1	-	2	-	-	-	-	-	-	-	-	-	1	-	1	
	P	*ATP6V1H*	51606	NM_213619	-	-	-	-	-	-	-	-	-	-	-	-	1	-	-	-	-	-	1	-	-	-	-	-	-	-	-	-	1	-	1	-	-	-	-	-	-	-	-	-	1	-	1	
	P	*ATP6V1H*	51606	NM_015941	-	-	-	-	-	-	-	-	-	-	-	-	1	-	-	-	-	-	1	-	-	-	-	-	-	-	-	-	1	-	1	-	-	-	-	-	-	-	-	-	1	-	1	
	P	*ATP6V1H*	51606	XM_005251257	-	-	-	-	-	-	-	-	-	-	-	-	1	-	-	-	-	-	1	-	-	-	-	-	-	-	-	-	-	-	-	-	-	-	-	-	-	-	-	-	1	-	1	
	P	*ATP6V1G3*	127124	XM_005244892	-	-	-	-	-	-	-	-	-	-	-	1	-	-	-	-	-	-	1	-	-	-	-	-	1	-	-	-	-	-	1	-	-	-	-	-	-	-	-	-	1	-	1	
	P	*ATP6V1G3*	127124	XM_005276749	-	-	-	-	-	-	-	-	-	-	-	1	-	-	-	-	-	-	1	-	-	-	-	-	1	-	-	-	-	-	1	-	-	-	-	-	-	-	-	-	1	-	1	
	P	*ATP6V1G3*	127124	NM_133262	-	-	-	-	-	-	-	-	-	-	-	1	-	-	-	-	-	-	1	-	-	-	-	-	1	-	-	-	-	-	1	-	-	-	-	-	-	-	-	-	1	-	1	
	P	*ATP6V1G3*	127124	NM_133326	-	-	-	-	-	-	-	-	-	-	-	-	-	-	-	-	-	-	-	-	-	-	-	-	1	-	-	-	-	-	1	-	-	-	-	-	-	-	-	-	-	-	-	
	P	*ACO1*	48	NM_001278352	-	-	-	-	-	-	-	-	-	-	-	-	-	-	-	-	-	-	-	-	-	-	-	-	-	-	-	1	-	-	1	1	8	2086	2079	0.0399	1	1	1	1	1	1	6	
	P	*ACO1*	48	XM_005251477	-	-	-	-	-	-	-	-	-	-	-	-	-	-	-	-	-	-	-	-	-	-	-	-	-	-	-	-	-	-	-	1	8	1936	1929	0.0399	1	-	1	-	1	1	4	
	P	*ACO1*	48	XM_005251476	-	-	-	-	-	-	-	-	-	-	-	-	-	-	-	-	-	-	-	-	-	-	-	-	-	-	-	-	-	-	-	1	8	2024	2017	0.0399	1	-	1	-	1	1	4	
	P	*ACO1*	48	NM_002197	-	-	-	-	-	-	-	-	-	-	-	-	-	-	-	-	-	-	-	-	-	-	-	-	-	-	-	-	-	-	-	1	8	2014	2007	0.0399	1	-	1	-	1	1	4	
	P	*ACO2*	50	NM_001098	-	1	7	2742	2736	0.0229	1	-	-	-	-	-	1	-	1	-	1	1	5	-	-	-	-	-	-	-	-	-	-	-	-	-	-	-	-	-	1	-	1	1	1	1	5	
	P	*ACO2*	50	XM_005261622	-	-	-	-	-	-	-	-	-	-	-	-	1	-	-	-	1	-	2	-	-	-	-	-	-	-	-	-	-	-	-	-	-	-	-	-	1	-	-	-	1	-	2	
	P	*ACO2*	50	XM_005261621	-	-	-	-	-	-	-	-	-	-	-	-	1	-	-	-	1	-	2	-	-	-	-	-	1	-	-	-	-	-	1	-	-	-	-	-	1	-	1	-	1	1	4	
	P	*CS*	1431	XM_005268664	-	-	-	-	-	-	-	1	-	-	-	-	-	-	-	-	1	-	2	-	-	-	-	-	1	-	-	-	1	-	2	-	-	-	-	-	-	-	-	1	1	-	2	
	P	*CS*	1431	XM_005268663	-	-	-	-	-	-	-	-	-	-	-	-	-	-	-	-	1	-	1	-	-	-	-	-	-	-	-	-	-	-	-	-	-	-	-	-	1	-	-	-	1	-	2	
	P	*CS*	1431	NM_004077	-	-	-	-	-	-	-	-	-	-	-	-	-	-	-	-	1	-	1	-	-	-	-	-	-	-	-	-	-	-	-	-	-	-	-	-	1	-	-	-	1	-	2	
	P	*CS*	1431	XM_005268662	-	-	-	-	-	-	-	-	-	-	-	-	-	-	-	-	1	-	1	-	-	-	-	-	-	-	-	-	-	-	-	-	-	-	-	-	1	-	-	-	1	-	2	
	P	*ACLY*	47	NM_001096	72.7517	-	-	-	-	-	1	1	-	-	-	1	1	-	-	1	1	1	7	-	-	-	-	-	-	-	-	-	1	-	1	-	-	-	-	-	1	-	-	-	1	-	2	
	P	*ACLY*	47	XM_005257397	72.7517	-	-	-	-	-	1	-	-	-	-	1	1	-	-	-	1	1	5	-	-	-	-	-	-	-	-	-	1	-	1	-	-	-	-	-	1	-	-	-	1	-	2	
	P	*ACLY*	47	XM_005277027	72.7517	-	-	-	-	-	1	-	-	-	-	1	1	-	-	-	1	1	5	-	-	-	-	-	-	-	-	-	1	-	1	-	-	-	-	-	1	-	-	-	1	-	2	
	P	*ACLY*	47	XM_005257396	72.7517	-	-	-	-	-	1	-	-	-	-	1	1	-	-	-	1	1	5	-	-	-	-	-	-	-	-	-	-	-	-	-	-	-	-	-	1	-	-	-	1	-	2	
	P	*ACLY*	47	XM_005277026	72.7517	-	-	-	-	-	1	-	-	-	-	1	1	-	-	-	1	1	5	-	-	-	-	-	-	-	-	-	-	-	-	-	-	-	-	-	1	-	-	-	1	-	2	
	P	*ACLY*	47	XM_005257394	72.7517	-	-	-	-	-	1	-	-	-	-	1	1	-	-	-	1	1	5	-	-	-	-	-	-	-	-	-	-	-	-	-	-	-	-	-	1	-	-	-	1	-	2	
	P	*ACLY*	47	XM_005277024	72.7517	-	-	-	-	-	1	-	-	-	-	1	1	-	-	-	1	1	5	-	-	-	-	-	-	-	-	-	-	-	-	-	-	-	-	-	1	-	-	-	1	-	2	
	P	*ACLY*	47	XM_005257393	72.7517	-	-	-	-	-	1	-	-	-	-	1	1	-	-	-	1	1	5	-	-	-	-	-	-	-	-	-	-	-	-	-	-	-	-	-	1	-	-	-	1	-	2	

Citrate cycle (TCA cycle)	P	ACLY	47	XM_005277023	72.7517	-	-	-	-	-	1	-	-	-	-	1	1	-	-	-	1	1	5	-	-	-	-	-	-	-	-	-	-	-	-	-	-	-	-	-	1	-	-	-	1	-	2
	P	ACLY	47	XM_005257395	72.7517	-	-	-	-	-	1	-	-	-	-	1	1	-	-	-	1	1	5	-	-	-	-	-	-	-	-	-	-	-	-	-	-	-	-	-	1	-	-	-	1	-	2
	P	ACLY	47	XM_005277025	72.7517	-	-	-	-	-	1	-	-	-	-	1	1	-	-	-	1	1	5	-	-	-	-	-	-	-	-	-	-	-	-	-	-	-	-	-	1	-	-	-	1	-	2
	P	ACLY	47	NM_198830	72.7517	-	-	-	-	-	1	-	-	-	-	1	1	-	-	-	1	1	5	-	-	-	-	-	-	-	-	-	1	-	1	-	-	-	-	-	1	-	-	-	1	-	2
	P	IDH1	3417	XM_005246521	-	-	-	-	-	-	-	1	-	-	-	-	-	-	-	-	1	-	2	-	-	-	-	-	-	-	-	1	1	-	2	-	-	-	-	-	-	-	-	-	1	-	1
	P	IDH1	3417	XM_005246524	-	-	-	-	-	-	-	-	-	-	-	-	-	-	-	-	1	-	1	-	-	-	-	-	-	-	-	-	-	-	-	-	-	-	-	-	-	-	-	-	1	-	1
	P	IDH1	3417	NM_005896	-	-	-	-	-	-	-	-	-	-	-	-	-	-	-	-	1	-	1	-	-	-	-	-	-	-	-	-	1	-	1	-	-	-	-	-	-	-	-	-	1	-	1
	P	IDH3B	3420	NM_006899	-	-	-	-	-	-	-	-	-	-	-	-	-	-	-	-	-	-	-	-	-	-	-	-	-	-	-	1	-	-	1	-	-	-	-	-	-	-	-	1	1	-	2
	P	IDH3B	3420	NM_174855	-	-	-	-	-	-	-	-	-	-	-	-	-	-	-	-	-	-	-	-	-	-	-	-	-	-	-	-	-	-	-	-	-	-	-	-	-	-	-	-	1	-	1
	P	IDH3B	3420	XM_005260716	-	-	-	-	-	-	-	-	-	-	-	-	-	-	-	-	-	-	-	-	-	-	-	-	-	-	-	-	-	-	-	-	-	-	-	-	-	-	-	-	1	-	1
	P	IDH3B	3420	NM_001258384	-	-	-	-	-	-	-	-	-	-	-	-	-	-	-	-	1	-	1	-	-	-	-	-	-	-	-	-	-	-	-	-	-	-	-	-	-	-	-	-	1	-	1
	P	IDH3G	3421	NM_174869	-	-	-	-	-	-	-	-	-	-	-	-	-	-	-	-	1	-	1	-	-	-	-	-	-	-	-	-	-	-	-	-	-	-	-	-	1	-	-	1	1	-	3
	P	IDH3G	3421	NM_004135	-	-	-	-	-	-	-	-	-	-	-	-	-	-	-	-	-	-	-	-	-	-	-	-	-	-	-	-	-	-	-	-	-	-	-	-	1	-	-	-	1	-	2
	P	OGDH	4967	XM_005249761	-	-	-	-	-	-	1	-	-	-	-	-	-	-	-	-	-	-	1	-	-	-	-	-	-	-	-	1	1	-	2	-	-	-	-	-	-	-	-	1	1	-	2
	P	OGDH	4967	NM_001003941	-	-	-	-	-	-	-	-	-	-	-	-	-	-	-	-	-	-	-	-	-	-	-	-	-	-	-	-	1	-	1	-	-	-	-	-	-	-	-	-	1	-	1
	P	OGDH	4967	XM_005249763	-	-	-	-	-	-	1	-	-	-	-	-	-	-	-	-	-	-	1	-	-	-	-	-	-	-	-	-	1	-	1	-	-	-	-	-	-	-	-	-	1	-	1
	P	OGDH	4967	XM_005249760	-	-	-	-	-	-	1	-	-	-	-	-	-	-	-	-	-	-	1	-	-	-	-	-	-	-	-	-	1	-	1	-	-	-	-	-	-	-	-	-	1	-	1
	P	OGDH	4967	XM_005249759	-	-	-	-	-	-	1	-	-	-	-	-	-	-	-	-	-	-	1	-	-	-	-	-	-	-	-	-	1	-	1	-	-	-	-	-	-	-	-	-	1	-	1
	P	OGDH	4967	XM_005249762	-	-	-	-	-	-	1	-	-	-	-	-	-	-	-	-	-	-	1	-	-	-	-	-	-	-	-	-	1	-	1	-	-	-	-	-	-	-	-	-	1	-	1
	P	OGDH	4967	NM_001165036	-	-	-	-	-	-	1	-	-	-	-	-	-	-	-	-	-	-	1	-	-	-	-	-	-	-	-	-	1	-	1	-	-	-	-	-	-	-	-	-	1	-	1
	P	OGDH	4967	NM_002541	-	-	-	-	-	-	1	-	-	-	-	-	-	-	-	-	-	-	1	-	-	-	-	-	-	-	-	-	1	-	1	-	-	-	-	-	-	-	-	-	1	-	1
	P	OGDHL	55753	NM_018245	-	-	-	-	-	-	-	1	-	-	-	-	1	-	-	1	1	-	4	-	-	-	-	-	-	-	-	-	-	-	-	-	-	-	-	-	1	-	-	1	1	1	4
	P	OGDHL	55753	NM_001143997	-	-	-	-	-	-	-	-	-	-	-	-	1	-	-	-	1	-	2	-	-	-	-	-	-	-	-	-	-	-	-	-	-	-	-	-	1	-	-	-	1	1	3
	P	OGDHL	55753	NM_001143996	-	-	-	-	-	-	-	-	-	-	-	-	1	-	-	-	1	-	2	-	-	-	-	-	-	-	-	-	-	-	-	-	-	-	-	-	1	-	-	-	1	1	3
	P	DLST	1743	NM_001933	-	-	-	-	-	-	-	-	-	-	-	-	-	-	-	1	-	-	1	-	-	-	-	-	-	-	-	-	-	-	-	-	-	-	-	-	1	-	-	-	1	-	2
	P	DLST	1743	NM_001244883	-	-	-	-	-	-	-	-	-	-	-	-	-	-	-	-	-	-	-	-	-	-	-	-	-	-	-	-	-	-	-	-	-	-	-	-	-	-	-	-	1	-	1
	P	DLD	1738	NM_000108	-	-	-	-	-	-	-	1	-	-	-	-	-	-	-	-	1	-	2	-	-	-	-	-	-	-	-	-	-	-	-	-	-	-	-	-	1	-	-	-	1	1	3
	P	DLD	1738	XM_005250184	-	-	-	-	-	-	-	-	-	-	-	-	-	-	-	-	-	-	-	-	-	-	-	-	-	-	-	-	-	-	-	-	-	-	-	-	1	-	-	-	1	1	3
	P	DLD	1738	XM_005250183	-	-	-	-	-	-	-	-	-	-	-	-	-	-	-	-	-	-	-	-	-	-	-	-	-	-	-	-	-	-	-	-	-	-	-	-	1	-	-	-	1	1	3
	P	SUCLA2	8803	XM_005266579	-	-	-	-	-	-	-	-	-	-	-	-	-	-	-	1	-	-	1	-	-	-	-	-	-	-	-	-	-	-	-	-	-	-	-	-	-	0	0	1	1	0	2
	P	SUCLA2	8803	NM_003850	-	-	-	-	-	-	-	-	-	-	-	-	-	-	-	-	-	-	-	-	-	-	-	-	-	-	-	-	-	-	-	-	-	-	-	-	-	0	0	0	1	0	1
	P	SUCLA2	8803	XM_005266580	-	-	-	-	-	-	-	-	-	-	-	-	-	-	-	-	-	-	-	-	-	-	-	-	-	-	-	-	-	-	-	-	-	-	-	-	-	0	0	0	1	0	1
	P	SUCLA2	8803	XM_005266581	-	-	-	-	-	-	-	-	-	-	-	-	-	-	-	-	-	-	-	-	-	-	-	-	-	-	-	-	-	-	-	-	-	-	-	-	-	0	0	0	1	0	1
	P	SUCLG1	8802	NM_003849	-	-	-	-	-	-	-	-	-	-	-	-	-	-	1	-	1	-	2	-	-	-	-	-	-	-	-	-	1	-	1	1	8	507	500	0.0158	1	0	1	1	1	1	5
	P	SUCLG2	8801	NM_003848	-	-	-	-	-	-	-	-	-	-	-	-	-	-	1	1	1	-	3	-	-	-	-	-	-	-	-	-	1	-	1	-	-	-	-	-	-	-	-	-	-	-	-
	P	SUCLG2	8801	NM_001177599	-	-	-	-	-	-	-	-	-	-	-	-	-	-	-	-	-	-	-	-	-	-	-	-	-	-	-	-	1	-	1	-	-	-	-	-	-	-	-	-	-	-	-
	P	SUCLG2	8801	XM_005264773	-	-	-	-	-	-	-	-	-	-	-	-	-	-	-	-	-	-	-	-	-	-	-	-	-	-	-	-	1	-	1	-	-	-	-	-	-	-	-	-	-	-	-
	P	MDH1	4190	NM_005917	-	-	-	-	-	-	-	-	-	-	-	-	-	-	-	1	1	-	2	-	-	-	-	-	-	-	-	-	-	-	-	-	-	-	-	-	-	-	-	1	1	-	2
	P	MDH1	4190	XM_005264320	-	-	-	-	-	-	-	-	-	-	-	-	-	-	-	-	1	-	1	-	-	-	-	-	-	-	-	-	1	-	1	-	-	-	-	-	-	-	-	-	1	-	1
	P	MDH1	4190	NM_001199112	-	-	-	-	-	-	-	-	-	-	-	-	-	-	-	-	1	-	1	-	-	-	-	-	-	-	-	-	1	-	1	-	-	-	-	-	-	-	-	-	1	-	1
	P	MDH1	4190	NM_001199111	-	-	-	-	-	-	-	-	-	-	-	-	-	-	-	-	1	-	1	-	-	-	-	-	-	-	-	-	-	-	-	-	-	-	-	-	-	-	-	-	1	-	1
	P	MDH1B	130752	NM_001039845	83.2125	-	-	-	-	-	-	-	1	-	-	1	-	-	1	1	1	1	6	-	-	-	-	-	-	-	-	-	-	-	-	-	-	-	-	-	1	1	-	-	1	1	4
	P	MDH1B	130752	XM_005246316	83.2125	-	-	-	-	-	-	-	1	-	-	1	-	-	-	-	1	1	4	-	-	-	-	-	-	-	-	-	-	-	-	-	-	-	-	-	1	-	-	-	1	1	3
	P	MDH1B	130752	XM_005246315	83.2125	-	-	-	-	-	-	-	1	-	-	1	-	-	-	-	1	1	4	-	-	-	-	-	-	-	-	-	-	-	-	-	-	-	-	-	1	-	-	-	1	1	3
	P	MDH2	4191	NM_005918	56.234	-	-	-	-	-	1	-	-	-	-	-	-	-	-	-	1	-	2	-	-	-	-	-	-	-	-	-	-	-	-	-	-	-	-	-	1	-	-	-	1	1	3
	P	MDH2	4191	XM_005250350	56.234	-	-	-	-	-	1	-	-	-	-	-	-	-	-	-	1	-	2	-	-	-	-	-	-	-	-	-	-	-	-	-	-	-	-	-	-	-	-	-	1	-	1
	P	MDH2	4191	XM_005277582	56.234	-	-	-	-	-	1	-	-	-	-	-	-	-	-	-	1	-	2	-	-	-	-	-	-	-	-	-	-	-	-	-	-	-	-	-	-	-	-	-	1	-	1
	P	PCK1	5105	NM_002591	-	-	-	-	-	-	-	-	-	-	-	-	-	-	-	1	-	-	1	-	-	-	-	-	-	-	-	-	1	-	1	-	-	-	-	-	1	-	-	-	1	1	3
	P	PCK1	5105	XM_005260416	-	-	-	-	-	-	-	-	-	-	-	-	-	-	-	-	-	-	-	-	-	-	-	-	-	-	-	-	1	-	1	-	-	-	-	-	1	-	-	-	1	1	3
	P	PCK1	5105	XM_005260415	-	-	-	-	-	-	-	-	-	-	-	-	-	-	-	-	-	-	-	-	-	-	-	-	-	-	-	-	1	-	1	-	-	-	-	-	1	-	-	-	1	1	3
	P	PCK2	5106	XM_005267728	-	-	-	-	-	-	-	1	-	-	-	-	-	-	-	-	-	-	1	-	-	-	-	-	-	-	-	-	1	-	1	-	-	-	-	-	1	-	1	1	1	1	5
	P	PCK2	5106	NM_001018073	-	-	-	-	-	-	-	-	-	-	-	-	-	-	-	-	1	-	1	-	-	-	-	-	-	-	-	-	1	-	1	-	-	-	-	-	1	-	1	-	1	1	4
	P	PCK2	5106	XM_005267729	-	-	-	-	-	-	-	-	-	-	-	-	-	-	-	-	-	-	-	-	-	-	-	-	-	-	-	-	1	-	1	-	-	-	-	-	1	-	1	-	1	1	4
	P	PCK2	5106	NM_004563	-	-	-	-	-	-	-	-	-	-	-	-	-	-	-	-	-	-	-	-	-	-	-	-	-	-	-	-	1	-	1	-	-	-	-	-	-	-	-	-	1	-	1
	P	PCK2	5106	XM_005267726	-	-	-	-	-	-	-	-	-	-	-	-	-	-	-	-	-	-	-	-	-	-	-	-	-	-	-	-	1	-	1	-	-	-	-	-	1	-	1	-	1	1	4
	P	PCK2	5106	XM_005267727	-	-	-	-	-	-	-	-	-	-	-	-	-	-	-	-	-	-	-	-	-	-	-	-	-	-	-	-	1	-	1	-	-	-	-	-	1	-	1	-	1	1	4
	P	PDHA1	5160	NM_001173454	-	-	-	-	-	-	-	1	-	-	-	-	-	-	-	1	-	-	2	-	-	-	-	-	1	-	-	1	1	-	3	-	-	-	-	-	-	-	-	1	1	-	2
	P	PDHA1	5160	NM_001173456	-	-	-	-	-	-	-	-	-	-	-	-	-	-	-	-	-	-	-	-	-	-	-	-	1	-	-	-	1	-	2	-	-	-	-	-	-	-	-	-	1	-	1
	P	PDHA1	5160	NM_000284	-	-	-	-	-	-	-	-	-	-	-	-	-	-	-	-	-	-	-	-	-	-	-	-	1	-	-	-	1	-	2	-	-	-	-	-	-	-	-	-	1	-	1
	P	PDHA1	5160	NM_001173455	-	-	-	-	-	-	-	-	-	-	-	-	-	-	-	-	-	-	-	-	-	-	-	-	1	-	-	-	1	-	2	-	-	-	-	-	-	-	-	-	1	-	1
	P	PDHA2	5161	NM_005390	-	-	-	-	-	-	-	-	-	-	-	-	1	-	-	1	-	-	2	-	-	-	-	-	-	-	-	-	-	-	-	-	-	-	-	-	1	-	-	1	1	-	3
	P	PDHB	5162	NM_000925	-	-	-	-	-	-	-	-	-	-	-	-	-	-	-	-	-	-	-	-	-	-	-	-	-	-	-	-	-	-	-	-	-	-	-	-	1	-	-	1	1	-	3
	P	PDHB	5162	NM_001173468	-	-	-	-	-	-	-	-	-	-	-	-	-	-	-	-	-	-	-	-	-	-	-	-	-	-	-	-	-	-	-	-	-	-	-	-	1	-	-	-	1	-	2
	P	PDHX	8050	NM_003477	87.867	1	9	2666	2658	0.0037	1	1	1	-	1	1	1	1	1	1	1	1	11	-	-	-	-	-	-	-	-	-	-	-	-	-	-	-	-	-	1	1	1	1	1	1	6
	P	PDHX	8050	NM_001166158	87.867	1	9	1985	1977	0.0037	1	-	1	-	1	1	1	1	-	-	1	1	8	-	-	-	-	-	-	-	-	-	-	-	-	-	-	-	-	-	-	-	-	-	1	-	1
	P	PDHX	8050	NM_001135024	87.867	1	9	2337	2329	0.0037	1	-	1	-	1	1	1	1	-	-	1	1	8	-	-	-	-	-	-	-	-	-	-	-	-	-	-	-	-	-	1	-	1	-	1	1	4
Pentose phosphate pathway	P	GPI	2821	NM_001184722	75.933	-	-	-	-	-	-	1	-	-	-	1	-	-	-	1	1	-	4	-	-	-	-	-	-	-	-	-	-	-	-	-	-	-	-	-	-	-	-	1	1	-	2
	P	GPI	2821	NM_000175	75.933	-	-	-	-	-	-	-	-	-	-	1	-	-	1	-	1	-	3	-	-	-	-	-	-	-	-	-	-	-	-	-	-	-	-	-	-	-	-	-	1	-	1
	P	GPI	2821	XM_005258764	75.933	-	-	-	-	-	-	-	-	-	-	1	-	-	-	-	1	-	2	-	-	-	-	-	-	-	-	-	-	-	-	-	-	-	-	-	-	-	-	-	1	-	1
	P	GPI	2821	XM_005258763	75.933	-	-	-	-	-	-	-	-	-	-	1	-	-	-	-	1	-	2	-	-	-	-	-	-	-	-	-	-	-	-	-	-	-	-	-	-	-	-	-	1	-	1
	P	G6PD	2539	NM_000402	-	-	-	-	-	-	-	-	-	-	-	-	-	-	-	-	1	-	1	-	-	-	-	-	-	-	-	-	-	-	-	-	-	-	-	-	-	-	-	-	-	-	-
	P	G6PD	2539	XM_005274658	-	-	-	-	-	-	-	-	-	-	-	-	-	-	-	-	1	-	1	-	-	-	-	-	-	-	-	-	-	-	-	-	-	-	-	-	-	-	-	-	-	-	-
	P	G6PD	2539	XM_005277834	-	-	-	-	-	-	-	-	-	-	-	-	-	-	-	-	1	-	1	-	-	-	-	-	-	-	-	-	-	-	-	-	-	-	-	-	-	-	-	-	-	-	-
	P	G6PD	2539	NM_001042351	-	-	-	-	-	-	-	-	-	-	-	-	-	-	-	-	1	-	1	-	-	-	-	-	-	-	-	-	-	-	-	-	-	-	-	-	-	-	-	-	-	-	-
	P	G6PD	2539	XM_005274657	-	-	-	-	-	-	-	-	-	-	-	-	-	-	-	-	1	-	1	-	-	-	-	-	-	-	-	-	-	-	-	-	-	-	-	-	-	-	-	-	-	-	-
	P	G6PD	2539	XM_005277833	-	-	-	-	-	-	-	-	-	-	-	-	-	-	-	-	1	-	1	-	-	-	-	-	-	-	-	-	-	-	-	-	-	-	-	-	-	-	-	-	-	-	-
	P	PGD	5226	NM_002631	36.1415	-	-	-	-	-	-	-	-	-	-	1	-	-	1	-	1	-	3	-	-	-	-	-	-	-	-	-	-	-	-	-	-	-	-	-	-	-	-	-	1	-	1
	P	PGD	5226	XM_005263471	36.1415	-	-	-	-	-	-	-	-	-	-	1	-	-	-	-	1	-	2	-	-	-	-	-	-	-	-	-	-	-	-	-	-	-	-	-	-	-	-	1	1	-	2
	P	PGLS	25796	NM_012088	-	-	-	-	-	-	-	-	-	-	-	-	-	-	-	-	1	-	1	-	-	-	-	-	-	-	-	-	-	-	-	-	-	-	-	-	-	-	-	1	1	-	2
	P	PGLS	25796	XM_005259849	-	-	-	-	-	-	-	-	-	-	-	-	-	-	-	-	-	-	-	-	-	-	-	-	-	-	-	-	-	-	-	-	-	-	-	-	-	-	-	-	1	-	1
	P	TALDO1	6888	NM_006755	-	-	-	-	-	-	-	-	-	-	-	-	-	-	-	-	-	-	-	-	-	-	-	-	-	-	-	-	-	-	-	-	-	-	-	-	-	-	-	-	1	-	1
	P	TALDO1	6888	XM_005253089	-	-	-	-	-	-	-	-	-	-	-	-	-	-	-	-	-	-	-	-	-	-	-	-	-	-	-	-	1	-	1	-	-	-	-	-	-	-	-	-	1	-	1
	P	TKTL1	8277	NM_012253	-	-	-	-	-	-	-	-	-	-	-	-	-	-	-	-	1	-	1	-	-	-	-	-	-	-	-	1	-	-	1	-	-	-	-	-	1	-	-	1	1	-	3
	P	TKTL1	8277	XM_005274756	-	-	-	-	-	-	-	-	-	-	-	-	-	-	-	-	1	-	1	-	-	-	-	-	-	-	-	-	-	-	-	-	-	-	-	-	1	-	-	-	1	-	2
	P	TKTL1	8277	XM_005277915	-	-	-	-	-	-	-	-	-	-	-	-	-	-	-	-	1	-	1	-	-	-	-	-	-	-	-	-	-	-	-	-	-	-	-	-	1	-	-	-	1	-	2
	P	TKTL1	8277	NM_001145934	-	-	-	-	-	-	-	-	-	-	-	-	-	-	-	-	1	-	1	-	-	-	-	-	-	-	-	-	1	-	1	-	-	-	-	-	1	-	-	-	1	-	2
	P	TKTL1	8277	NM_001145933	-	-	-	-	-	-	-	-	-	-	-	-	-	-	-	-	1	-	1	-	-	-	-	-	-	-	-	-	-	-	-	-	-	-	-	-	1	-	-	-	1	-	2
	P	TKTL2	84076	NM_032136	64.4937	1	7	2637	2631	0.0474	1	1	-	-	-	1	-	-	1	1	1	1	7	-	-	-	-	-	-	-	-	1	1	-	2	-	-	-	-	-	1	1	-	1	1	1	5
	P	TKTL2	84076	XM_005263279	64.4937	1	7	2429	2423	0.0454	1	-	-	-	-	1	-	-	-	-	1	1	4	-	-	-	-	-	-	-	-	-	1	-	1	-	-	-	-	-	1	-	-	-	1	1	3
	P	RPIA	22934	NM_144563	69.6616	-	-	-	-	-	1	1	-	-	-	1	-	-	1	-	1	1	6	-	-	-	-	-	-	-	-	-	-	-	-	-	-	-	-	-	-	-	-	1	1	-	2
	P	DERA	51071	NM_015954	69.7246	1	7	1514	1508	0.0390	1	1	-	-	-	1	-	-	1	1	1	1	7	-	-	-	-	-	-	-	-	-	-	-	-	-	-	-	-	-	-	-	-	-	1	-	1
	P	RBKS	64080	NM_022128	-	-	-	-	-	-	-	-	-	-	-	-	-	-	-	-	-	-	-	-	-	-	-	-	-	-	-	-	-	-	-	-	-	-	-	-	-	-	-	-	1	-	1
	P	PRPS1	5631	NM_002764	51.84	-	-	-	-	-	1	-	1	-	-	1	-	-	1	-	1	1	6	-	-	-	-	-	-	-	-	-	1	-	1	-	-	-	-	-	-	-	-	1	1	-	2
	P	PRPS1	5631	NM_001204402	51.84	-	-	-	-	-	1	-	1	-	-	1	-	-	-	-	1	1	5	-	-	-	-	-	-	-	-	-	1	-	1	-	-	-	-	-	-	-	-	-	1	-	1
	P	PRPS1	5631	XM_005262171	51.84	-	-	-	-	-	1	-	1	-	-	1	-	-	-	-	1	1	5	-	-	-	-	-	-	-	-	-	1	-	1	-	-	-	-	-	-	-	-	-	1	-	1
	P	PRPS2	5634	NM_001039091	-	-	-	-	-	-	-	-	-	-	-	-	-	-	-	1	1	-	2	-	-	-	-	-	-	-	-	-	-	-	-	-	-	-	-	-	-	-	-	-	1	-	1
	P	PRPS2	5634	NM_002765	-	-	-	-	-	-	-	-	-	-	-	-	-	-	-	-	1	-	1	-	-	-	-	-	-	-	-	-	-	-	-	-	-	-	-	-	-	-	-	-	1	-	1
	P	SHPK	23729	NM_013276	87.8176	-	-	-	-	-	1	-	-	-	-	1	-	-	1	1	1	1	6	-	-	-	-	-	-	-	-	-	1	-	1					-	1	1	-	1	1	1	5
	P	SHPK	23729	XM_005256582	87.8176	-	-	-	-	-	-	-	-	-	-	1	-	-	-	-	-	-	1					-	-	-	-	-	1	-	1	1	7	386	380	0.0442	1	-	-	-	1	1	3
Glycogen synthase	P	GSK3A	2931	NM_019884	-	-	-	-	-	-	-	-	-	-	-	-	-	-	-	-	1	-	1	-	-	-	-	-	-	-	-	1	-	-	1	-	-	-	-	-	1	-	-	1	1	-	3
	V	GSK3B	2932	NM_002093	-	1	10	3212	3203	0.0046	1	1	1	-	-	1	-	1	-	1	1	1	8	-	-	-	-	-	-	-	-	-	1	-	1	-	-	-	-	-	-	-	-	-	1	-	1
	V	GSK3B	2932	NM_001146156	-	1	10	3173	3164	0.0046	1	-	1	-	-	1	-	1	-	-	1	1	6	-	-	-	-	-	-	-	-	-	1	-	1	-	-	-	-	-	-	-	-	-	1	-	1
	P	EIF4B	1975	XM_005268709	-	-	-	-	-	-	-	-	-	-	-	-	-	-	-	-	1	-	1	-	-	-	-	-	-	-	-	-	-	-	-	-	-	-	-	-	-	-	-	-	-	-	-
	P	EIF4B	1975	XM_005268710	-	-	-	-	-	-	-	-	-	-	-	-	-	-	-	-	1	-	1	-	-	-	-	-	-	-	-	-	-	-	-	-	-	-	-	-	-	-	-	-	-	-	-
	P	EIF4B	1975	NM_001417	-	-	-	-	-	-	-	-	-	-	-	-	-	-	-	-	1	-	1	-	-	-	-	-	-	-	-	-	-	-	-	-	-	-	-	-	-	-	-	-	-	-	-
	P	EIF4E	1977	NM_001130679	-	-	-	-	-	-	-	1	-	-	-	-	-	-	-	-	1	-	2	-	-	-	-	-	-	-	-	-	1	-	1	-	-	-	-	-	-	-	-	-	1	-	1
	P	EIF4E	1977	NM_001130678	-	-	-	-	-	-	-	-	-	-	-	-	-	-	-	-	1	-	1	-	-	-	-	-	-	-	-	-	1	-	1	-	-	-	-	-	-	-	-	-	1	-	1
	P	EIF4E	1977	NM_001968	-	-	-	-	-	-	-	-	-	-	-	-	-	-	-	-	-	-	-	-	-	-	-	-	-	-	-	-	-	-	-	-	-	-	-	-	-	-	-	-	1	-	1
	P	EIF4E1B	253314	NM_001099408	11.7322	-	-	-	-	-	-	-	-	-	-	1	-	-	1	-	1	-	3	-	-	-	-	-	-	-	-	-	-	-	-	-	-	-	-	-	1	-	-	-	1	-	2
	P	EIF4E2	9470	XM_005246976	-	-	-	-	-	-	-	-	-	-	-	1	-	-	-	-	1	-	2	-	-	-	-	-	-	-	-	-	-	-	-	-	-	-	-	-	-	-	-	1	1	-	2
	P	EIF4E2	9470	NM_004846	-	-	-	-	-	-	-	-	-	-	-	1	-	-	-	-	-	-	1	-	-	-	-	-	-	-	-	-	-	-	-	-	-	-	-	-	-	-	-	-	1	-	1
	P	EIF4E2	9470	XM_005246975	-	-	-	-	-	-	-	-	-	-	-	1	-	-	-	-	-	-	1	-	-	-	-	-	-	-	-	-	-	-	-	-	-	-	-	-	-	-	-	-	1	-	1
	P	EIF4EBP1	1978	NM_004095	-	-	-	-	-	-	-	-	-	-	-	-	-	-	-	-	-	-	-	-	-	-	-	-	-	-	-	-	-	-	-	-	-	-	-	-	-	-	-	1	1	-	2
	P	LAMTOR1	55004	NM_017907	-	-	-	-	-	-	-	-	-	-	-	-	-	-	-	-	1	-	1	-	-	-	-	-	-	-	-	-	-	-	-	-	-	-	-	-	-	-	-	-	1	-	1
	P	LAMTOR3	8649	NM_021970	43.3561	-	-	-	-	-	1	1	-	-	-	1	-	-	1	1	1	1	7	-	-	-	-	-	-	-	-	-	-	-	-	-	-	-	-	-	-	-	-	1	1	-	2
	P	LAMTOR3	8649	NM_001243736	43.3561	-	-	-	-	-	1	-	-	-	-	1	-	-	-	-	1	1	4	-	-	-	-	-	-	-	-	-	-	-	-	-	-	-	-	-	-	-	-	-	1	-	1
	P	LAMTOR4	389541	NM_001008395	-	-	-	-	-	-	-	-	-	-	-	1	-	-	1	1	1	-	4	-	-	-	-	-	-	-	-	-	-	-	-	-	-	-	-	-	-	-	-	-	1	-	1
	P	LAMTOR5	10542	NM_006402	-	-	-	-	-	-	-	-	-	-	-	-	-	-	-	-	-	-	-	-	-	-	-	-	-	-	-	-	-	-	-	-	-	-	-	-	-	-	-	-	1	-	1
	P	MLST8	64223	XM_005255480	-	-	-	-	-	-	-	-	-	-	-	1	-	-	-	-	1	-	2	-	-	-	-	-	-	-	-	-	-	-	-	-	-	-	-	-	1	-	-	1	1	-	3
	P	MLST8	64223	XM_005255483	-	-	-	-	-	-	-	-	-	-	-	1	-	-	-	-	1	-	2	-	-	-	-	-	1	-	-	-	-	-	1	-	-	-	-	-	-	-	-	-	1	-	1
	P	MLST8	64223	XM_005255482	-	-	-	-	-	-	-	-	-	-	-	1	-	-	-	-	1	-	2	-	-	-	-	-	1	-	-	-	-	-	1	-	-	-	-	-	-	-	-	-	1	-	1
	P	MLST8	64223	XM_005255477	-	-	-	-	-	-	-	-	-	-	-	1	-	-	-	-	1	-	2	-	-	-	-	-	-	-	-	-	-	-	-	-	-	-	-	-	1	-	-	-	1	-	2

doi: 10.18632/oncotarget.14769

PTEN/AKT/AMPK/mTOR Pathway

P	MLST8	64223	XM_005255476	-	-	-	-	-	-	-	-	-	-	-	1	-	-	-	-	1	-	2	-	-	-	-	-	-	-	-	-	-	-	-	-	-	-	-	-	1	-	-	-	1	-	2
P	MLST8	64223	NM_001199174	-	-	-	-	-	-	-	-	-	-	-	1	-	-	-	-	1	-	2	-	-	-	-	-	-	-	-	-	-	-	-	-	-	-	-	-	1	-	-	-	1	-	2
P	MLST8	64223	XM_005255475	-	-	-	-	-	-	-	-	-	-	-	1	-	-	-	-	1	-	2	-	-	-	-	-	-	-	-	-	-	-	-	-	-	-	-	-	1	-	-	-	1	-	2
P	MLST8	64223	XM_005255479	-	-	-	-	-	-	-	-	-	-	-	1	-	-	-	-	1	-	2	-	-	-	-	-	-	-	-	-	-	-	-	-	-	-	-	-	1	-	-	-	1	-	2
P	MLST8	64223	XM_005255481	-	-	-	-	-	-	-	-	-	-	-	1	-	-	-	-	1	-	2	-	-	-	-	-	1	-	-	-	-	-	1	-	-	-	-	-	-	-	-	-	1	-	1
P	MLST8	64223	XM_005255478	-	-	-	-	-	-	-	-	-	-	-	1	-	-	-	-	1	-	2	-	-	-	-	-	-	-	-	-	-	-	-	-	-	-	-	-	1	-	-	-	1	-	2
P	MLST8	64223	NM_001199173	-	-	-	-	-	-	-	-	-	-	-	1	-	-	-	-	1	-	2	-	-	-	-	-	-	-	-	-	-	-	-	-	-	-	-	-	1	-	-	-	1	-	2
P	MLST8	64223	NM_001199175	-	-	-	-	-	-	-	-	-	-	-	1	-	-	-	-	1	-	2	-	-	-	-	-	-	-	-	-	-	-	-	-	-	-	-	-	1	-	-	-	1	-	2
P	MLST8	64223	NM_022372	-	-	-	-	-	-	-	-	-	-	-	1	-	-	-	-	1	-	2	-	-	-	-	-	-	-	-	-	-	-	-	-	-	-	-	-	1	-	-	-	1	-	2
P	MTOR	2475	XM_005263438	-	-	-	-	-	-	-	-	-	-	-	-	-	-	-	-	1	-	1	-	-	-	-	-	-	-	-	-	-	-	-	-	-	-	-	-	1	-	1	1	1	1	5
P	MTOR	2475	XM_005263439	-	-	-	-	-	-	-	-	-	-	-	-	-	-	-	-	1	-	1	-	-	-	-	-	-	-	-	-	1	-	1	-	-	-	-	-	1	-	1	-	1	1	4
P	MTOR	2475	NM_004958	-	-	-	-	-	-	-	-	-	-	-	-	-	-	-	-	1	-	1	-	-	-	-	-	-	-	-	-	-	-	-	-	-	-	-	-	1	-	1	-	1	1	4
P	MTOR	2475	XM_005263440	-	-	-	-	-	-	-	-	-	-	-	-	-	-	-	-	-	-	-	-	-	-	-	-	-	-	-	-	-	-	-	-	-	-	-	-	1	-	-	-	1	1	3
P	MTOR	2475	XM_005263441	-	-	-	-	-	-	-	-	-	-	-	-	-	-	-	-	-	-	-	-	-	-	-	-	-	-	-	-	-	-	-	-	-	-	-	-	1	-	-	-	1	-	2
P	RHEB	6009	NM_005614	9.49118	-	-	-	-	-	-	-	-	-	-	1	-	-	1	-	1	-	3	-	-	-	-	-	-	-	-	-	1	-	1	-	-	-	-	-	-	-	-	1	1	-	2
P	RHEB	6009	XM_005250034	9.49118	-	-	-	-	-	-	-	-	-	-	1	-	-	-	-	1	-	2	-	-	-	-	-	-	-	-	-	1	-	1	-	-	-	-	-	-	-	-	-	1	-	1
P	RHOA	387	NM_001664	-	-	-	-	-	-	-	-	-	-	-	-	-	-	-	1	1	-	2	-	-	-	-	-	-	-	-	-	1	-	1	-	-	-	-	-	-	-	-	1	1	-	2
P	RHOA	387	XM_005265116	-	-	-	-	-	-	-	-	-	-	-	-	-	-	-	-	1	-	1	-	-	-	-	-	-	-	-	-	1	-	1	-	-	-	-	-	-	-	-	-	1	-	1
P	RICTOR	253260	XM_005248277	82.0888	-	-	-	-	-	-	1	-	-	-	1	-	-	-	1	1	1	5	-	-	-	-	-	-	-	-	-	1	-	1	-	-	-	-	-	1	1	1	1	1	1	6
P	RICTOR	253260	NM_152756	82.0888	-	-	-	-	-	-	-	-	-	-	1	-	1	1	-	1	1	5	-	-	-	-	-	-	-	-	-	1	-	1	-	-	-	-	-	1	-	1	-	1	1	4
P	RICTOR	253260	XM_005248279	82.0888	-	-	-	-	-	1	-	-	-	-	1	-	-	-	-	1	1	4	-	-	-	-	-	-	-	-	-	-	-	-	-	-	-	-	-	1	-	-	-	1	-	2
P	RICTOR	253260	XM_005248278	82.0888	-	-	-	-	-	1	-	-	-	-	1	-	-	-	-	1	1	4	-	-	-	-	-	-	-	-	-	-	-	-	-	-	-	-	-	1	-	-	-	1	-	2
P	RNF152	220441	XM_005266653	95.2273	-	-	-	-	-	1	1	1	-	-	-	-	-	-	-	1	1	5	-	-	-	-	-	1	-	-	-	1	-	2	-	-	-	-	-	1	1	1	-	1	1	5
P	RNF152	220441	XM_005266654	95.2273	-	-	-	-	-	1	-	1	-	-	-	-	-	-	-	1	1	4	-	-	-	-	-	-	-	-	-	1	-	1	-	-	-	-	-	1	-	1	-	1	1	4
P	RNF152	220441	XM_005266652	95.2273	-	-	-	-	-	1	-	1	-	-	-	-	-	-	-	1	1	4	-	-	-	-	-	-	-	-	-	1	-	1	-	-	-	-	-	1	-	1	-	1	1	4
P	RNF152	220441	XM_005266651	95.2273	-	-	-	-	-	1	-	1	-	-	-	-	-	-	-	1	1	4	-	-	-	-	-	-	-	-	-	1	-	1	-	-	-	-	-	1	-	1	-	1	1	4
P	RNF152	220441	XM_005266650	95.2273	-	-	-	-	-	1	-	1	-	-	-	-	-	-	-	1	1	4	-	-	-	-	-	-	-	-	-	1	-	1	-	-	-	-	-	1	-	1	-	1	1	4
P	RNF152	220441	NM_173557	95.2273	-	-	-	-	-	-	-	-	-	-	-	-	-	-	-	1	-	1	-	-	-	-	-	-	-	-	-	1	-	1	-	-	-	-	-	1	-	1	-	1	1	4
P	RPS6	6194	XM_005251542	-	-	-	-	-	-	-	1	-	-	-	-	-	-	-	-	-	-	1	-	-	-	-	-	1	-	-	-	1	-	2	-	-	-	-	-	-	-	-	1	1	-	2
P	RPS6	6194	XM_005251543	-	-	-	-	-	-	-	-	-	-	-	-	-	-	-	-	-	-	-	-	-	-	-	-	-	-	-	-	-	-	-	-	-	-	-	-	1	-	-	-	1	-	2
P	RPS6	6194	NM_001010	-	-	-	-	-	-	-	-	-	-	-	-	-	-	-	-	-	-	-	-	-	-	-	-	-	-	-	-	-	-	-	-	-	-	-	-	1	-	-	-	1	-	2
P	RPS6KA1	6195	NM_002953	-	-	-	-	-	-	-	1	-	-	-	-	-	-	-	-	-	-	1	-	-	-	-	-	-	-	-	1	-	-	1	-	-	-	-	-	-	-	-	1	1	-	2
P	RPS6KA1	6195	XM_005245967	-	-	-	-	-	-	-	-	-	-	-	-	-	-	-	-	-	-	-	-	-	-	-	-	-	-	-	-	1	-	1	-	-	-	-	-	-	-	-	-	1	-	1
P	RPS6KA1	6195	XM_005245966	-	-	-	-	-	-	-	-	-	-	-	-	-	-	-	-	-	-	-	-	-	-	-	-	-	-	-	-	-	-	-	-	-	-	-	-	-	-	-	-	1	-	1
P	RPS6KA1	6195	NM_001006665	-	-	-	-	-	-	-	-	-	-	-	-	-	-	-	-	-	-	-	-	-	-	-	-	-	-	-	-	-	-	-	-	-	-	-	-	-	-	-	-	1	-	1
P	RPS6KA2	6196	XM_005267092	81.1615	-	-	-	-	-	1	-	1	-	-	1	-	-	-	1	1	1	6	-	-	-	-	-	-	-	-	-	-	-	-	-	-	-	-	-	-	-	-	1	1	-	2
P	RPS6KA2	6196	NM_001006932	81.1615	-	-	-	-	-	1	-	1	-	-	1	-	-	-	-	1	1	5	-	-	-	-	-	-	-	-	-	-	-	-	-	-	-	-	-	-	-	-	-	1	-	1
P	RPS6KA2	6196	NM_021135	81.1615	-	-	-	-	-	1	-	1	-	-	1	-	-	-	-	1	1	5	-	-	-	-	-	-	-	-	-	1	-	1	-	-	-	-	-	-	-	-	-	1	-	1
P	RPS6KA3	6197	NM_004586	-	-	-	-	-	-	1	-	-	-	-	-	-	-	1	-	1	-	3	-	-	-	-	-	-	-	-	-	-	-	-	-	-	-	-	-	-	-	-	1	1	-	2
P	RPS6KA3	6197	XM_005274574	-	-	-	-	-	-	1	-	-	-	-	-	-	-	-	-	1	-	2	-	-	-	-	-	1	-	-	1	-	-	2	-	-	-	-	-	-	-	-	-	1	-	1
P	RPS6KA3	6197	XM_005274577	-	-	-	-	-	-	1	-	-	-	-	-	-	-	-	-	1	-	2	-	-	-	-	-	-	-	-	-	-	-	-	-	-	-	-	-	-	-	-	-	1	-	1
P	RPS6KA3	6197	XM_005274576	-	-	-	-	-	-	1	-	-	-	-	-	-	-	-	-	1	-	2	-	-	-	-	-	-	-	-	-	-	-	-	-	-	-	-	-	-	-	-	-	1	-	1
P	RPS6KA3	6197	XM_005274575	-	-	-	-	-	-	1	-	-	-	-	-	-	-	-	-	1	-	2	-	-	-	-	-	-	-	-	-	-	-	-	-	-	-	-	-	-	-	-	-	1	-	1
P	RPS6KA3	6197	XM_005274573	-	-	-	-	-	-	1	-	-	-	-	-	-	-	-	-	1	-	2	-	-	-	-	-	-	-	-	-	-	-	-	-	-	-	-	-	-	-	-	-	1	-	1
P	RPS6KA6	27330	NM_014496	-	-	-	-	-	-	1	-	-	-	-	-	-	-	-	-	1	-	2	-	-	-	-	-	-	-	-	-	-	-	-	-	-	-	-	-	-	-	-	-	1	-	1
P	RPS6KA6	27330	XM_005262123	-	-	-	-	-	-	1	-	-	-	-	-	-	-	-	-	1	-	2	-	-	-	-	-	-	-	-	-	-	-	-	-	-	-	-	-	-	-	-	-	1	-	1
P	RPS6KB1	6198	NM_001272044	92.7466	-	-	-	-	-	-	-	-	-	-	-	-	-	-	-	-	-	-	-	-	-	-	-	-	-	-	-	1	-	1	-	-	-	-	-	-	-	-	-	1	-	1
P	RPS6KB1	6198	NM_001272043	92.7466	-	-	-	-	-	1	-	1	-	-	1	-	-	-	-	1	1	5	-	-	-	-	-	-	-	-	-	1	-	1	-	-	-	-	-	-	-	-	-	1	-	1
P	RPS6KB1	6198	XM_005257565	92.7466	-	-	-	-	-	1	-	1	-	-	1	-	-	-	-	1	1	5	-	-	-	-	-	-	-	-	-	1	-	1	-	-	-	-	-	-	-	-	-	1	-	1
P	RPS6KB1	6198	NM_001272042	92.7466	-	-	-	-	-	1	-	1	-	-	1	-	-	-	-	1	1	5	-	-	-	-	-	-	-	-	-	1	-	1	-	-	-	-	-	-	-	-	-	1	-	1
P	RPS6KB1	6198	NM_001272060	92.7466	-	-	-	-	-	1	-	1	-	-	1	-	-	-	-	1	1	5	-	-	-	-	-	-	-	-	-	1	-	1	-	-	-	-	-	-	-	-	-	1	-	1
P	RPS6KB1	6198	NM_003161	92.7466	-	-	-	-	-	-	-	-	-	-	-	-	-	-	-	-	-	-	-	-	-	-	-	-	-	-	-	1	-	1	-	-	-	-	-	-	-	-	-	1	-	1
P	RPS6KB2	6199	NM_003952	16.3256	-	-	-	-	-	-	1	-	-	-	1	1	-	1	1	1	-	6	-	-	-	-	-	-	-	-	-	1	-	1	-	-	-	-	-	-	-	-	1	1	-	2
P	RPS6KB2	6199	XM_005274165	16.3256	-	-	-	-	-	-	-	-	-	-	1	1	-	-	-	1	-	3	-	-	-	-	-	-	-	-	-	1	-	1	-	-	-	-	-	-	-	-	-	1	-	1
P	RPS6KB2	6199	XM_005274164	16.3256	-	-	-	-	-	-	-	-	-	-	1	1	-	-	-	1	-	3	-	-	-	-	-	-	-	-	-	-	-	-	-	-	-	-	-	-	-	-	-	1	-	1
P	RPTOR	57521	NM_020761	-	-	-	-	-	-	-	-	-	-	-	1	-	-	-	1	1	-	3	-	-	-	-	-	-	-	-	-	-	-	-	-	-	-	-	-	1	1	1	1	1	1	6
P	RPTOR	57521	XM_005257540	-	-	-	-	-	-	-	-	-	-	-	1	-	-	-	-	1	-	2	-	-	-	-	-	-	-	-	-	-	-	-	-	-	-	-	-	-	-	-	-	1	-	1
P	RPTOR	57521	XM_005257541	-	-	-	-	-	-	-	-	-	-	-	1	-	-	-	-	1	-	2	-	-	-	-	-	-	-	-	-	1	-	1	-	-	-	-	-	-	-	-	-	1	-	1
P	RPTOR	57521	XM_005257539	-	-	-	-	-	-	-	-	-	-	-	1	-	-	-	-	1	-	2	-	-	-	-	-	-	-	-	-	-	-	-	-	-	-	-	-	-	-	-	-	1	-	1
P	RPTOR	57521	NM_001163034	-	-	-	-	-	-	-	-	-	-	-	1	-	-	-	-	1	-	2	-	-	-	-	-	-	-	-	-	-	-	-	-	-	-	-	-	1	-	1	-	1	1	4
P	RRAGA	10670	NM_006570	-	-	-	-	-	-	-	-	-	-	-	-	-	-	-	-	-	-	-	-	-	-	-	-	1	-	-	-	-	-	1	-	-	-	-	-	-	-	-	1	1	-	2
P	RRAGB	10325	XM_005261973	-	1	9	2064	2056	0.0038	1	-	1	-	-	-	-	-	-	-	1	1	4	-	-	-	-	-	-	-	-	-	-	-	-	-	-	-	-	-	-	-	-	-	1	-	1
P	RRAGB	10325	NM_016656	-	-	-	-	-	-	-	-	-	-	-	-	-	-	-	-	1	-	1	-	-	-	-	-	-	-	-	-	-	-	-	-	-	-	-	-	-	-	-	-	1	-	1
P	RRAGB	10325	XM_005261974	-	-	-	-	-	-	-	-	-	-	-	-	-	-	-	-	1	-	1	-	-	-	-	-	-	-	-	-	-	-	-	-	-	-	-	-	-	-	-	-	1	-	1
P	RRAGB	10325	NM_006064	-	-	-	-	-	-	-	-	-	-	-	-	-	-	-	-	-	-	-	-	-	-	-	-	-	-	-	-	-	-	-	-	-	-	-	-	-	-	-	-	1	-	1
P	RRAGB	10325	XM_005261975	-	-	-	-	-	-	-	-	-	-	-	-	-	-	-	-	-	-	-	-	-	-	-	-	-	-	-	-	-	-	-	-	-	-	-	-	-	-	-	-	1	-	1
P	RRAGC	64121	NM_022157	-	-	-	-	-	-	1	-	-	-	-	-	-	1	-	-	1	-	3	-	-	-	-	-	-	-	-	-	1	-	1	-	-	-	-	-	-	-	-	-	1	-	1
P	RRAGC	64121	NM_001271851	-	-	-	-	-	-	1	-	-	-	-	-	-	1	-	-	1	-	3	-	-	-	-	-	-	-	-	-	1	-	1	-	-	-	-	-	-	-	-	-	1	-	1
P	RRAGD	58528	NM_021244	79.8089	-	-	-	-	-	1	1	1	-	-	1	-	-	-	1	1	1	7	-	-	-	-	-	-	-	-	-	-	-	-	-	-	-	-	-	-	-	-	-	1	-	1
P	RRAGD	58528	XM_005248755	79.8089	1	8	4137	4130	0.0476	1	-	1	-	-	1	-	-	-	-	1	1	5	-	-	-	-	-	-	-	-	-	-	-	-	-	-	-	-	-	-	-	-	1	1	-	3
P	SEC13	6396	NM_001136232	-	-	-	-	-	-	-	-	-	-	-	-	-	-	-	-	-	-	-	-	-	-	-	-	1	-	-	-	1	-	2	-	-	-	-	-	-	-	-	1	1	1	6
P	SEC13	6396	XM_005265378	-	-	-	-	-	-	-	-	-	-	-	-	-	-	-	-	-	-	-	-	-	-	-	-	-	-	-	-	1	-	1	-	-	-	-	-	1	-	-	-	1	-	2
P	SEC13	6396	XM_005265379	-	-	-	-	-	-	-	-	-	-	-	-	-	-	-	-	-	-	-	-	-	-	-	-	-	-	-	-	1	-	1	-	-	-	-	-	1	-	-	-	1	-	1
P	SEC13	6396	NM_001278946	-	-	-	-	-	-	-	-	-	-	-	-	-	-	-	-	-	-	-	-	-	-	-	-	-	-	-	-	-	-	-	-	-	-	-	-	1	-	-	-	1	-	2
P	SEC13	6396	XM_005265377	-	-	-	-	-	-	-	-	-	-	-	-	-	-	-	-	-	-	-	-	-	-	-	-	-	-	-	-	-	-	-	-	-	-	-	-	1	-	-	-	1	-	2
P	SEC13	6396	NM_183352	-	-	-	-	-	-	-	-	-	-	-	-	-	-	-	-	-	-	-	-	-	-	-	-	-	-	-	-	-	-	-	-	-	-	-	-	1	-	-	-	1	-	2
P	SEC13	6396	NM_030673	-	-	-	-	-	-	-	-	-	-	-	-	-	-	-	-	-	-	-	-	-	-	-	-	-	-	-	-	-	-	-	-	-	-	-	-	1	-	-	-	1	-	2
P	SEC13	6396	NM_001136026	-	-	-	-	-	-	-	-	-	-	-	-	-	-	-	-	-	-	-	-	-	-	-	-	-	-	-	-	-	-	-	-	-	-	-	-	1	-	-	-	1	-	2
P	SEH1L	81929	NM_031216	81.4037	1	8	3272	3265	0.0344	1	1	1	-	1	1	-	-	1	1	1	1	9	-	-	-	-	-	1	-	-	-	-	-	1	-	-	-	-	-	1	-	-	-	1	-	2
P	SEH1L	81929	NM_001013437	81.4037	1	8	1650	1643	0.0074	1	-	1	-	1	1	-	-	-	-	1	1	6	-	-	-	-	-	1	-	-	-	-	-	1	-	-	-	-	-	1	-	-	-	1	-	1
P	SEH1L	81929	XM_005258152	81.4037	1	8	1498	1491	0.0073	1	-	1	-	-	1	-	-	-	-	1	1	5	-	-	-	-	-	1	-	-	-	-	-	1	-	-	-	-	-	-	-	-	1	1	-	2
P	SEH1L	81929	XM_005258153	81.4037	-	-	-	-	-	-	-	-	-	-	1	-	-	-	-	1	-	2	-	-	-	-	-	1	-	-	-	-	-	1	-	-	-	-	-	1	-	-	-	1	-	2
P	SEH1L	6400	NM_005065	81.4037	-	-	-	-	-	1	1	-	-	-	1	-	-	1	1	1	1	7	-	-	-	-	-	-	-	-	-	-	-	-	-	-	-	-	-	-	-	-	-	1	-	1
P	SEH1L	6400	XM_005267988	81.4037	-	-	-	-	-	1	-	-	-	-	1	-	-	-	-	1	1	4	-	-	-	-	-	-	-	-	-	-	-	-	-	-	-	-	-	-	-	-	-	1	-	1
P	SEH1L	6400	XM_005267989	81.4037	-	-	-	-	-	1	-	-	-	-	1	-	-	-	-	1	1	4	-	-	-	-	-	-	-	-	-	-	-	-	-	-	-	-	-	-	-	-	1	1	-	2
P	SEH1L	6400	NM_001244984	81.4037	-	-	-	-	-	1	-	-	-	-	1	-	-	-	-	1	-	3	-	-	-	-	-	-	-	-	-	-	-	-	-	-	-	-	-	-	-	-	-	1	-	1
P	SEH1L	6400	XM_005267990	81.4037	-	-	-	-	-	-	-	-	-	-	1	-	-	-	-	-	-	1	-	-	-	-	-	-	-	-	-	-	-	-	-	-	-	-	-	-	-	-	-	-	-	-
P	SESN2	83667	NM_031459	70.7366	1	8	3149	3142	0.0257	1	1	1	-	1	1	-	1	1	1	1	1	10	-	-	-	-	-	-	-	-	-	-	-	-	-	-	-	-	-	-	-	-	1	1	-	2
P	SGK1	6446	NM_001143676	49.7505	-	-	-	-	-	-	-	-	-	-	1	-	-	-	1	1	-	3	-	-	-	-	-	1	1	-	-	1	-	3	-	-	-	-	-	-	-	-	-	1	-	1
P	SGK1	6446	NM_005627	49.7505	-	-	-	-	-	-	-	-	-	-	1	-	-	1	-	1	-	3	-	-	-	-	-	-	-	-	-	-	-	-	-	-	-	-	-	-	-	-	-	1	-	1
P	SGK1	6446	XM_005267096	49.7505	-	-	-	-	-	-	-	-	-	-	1	-	-	-	-	1	-	2	-	-	-	-	-	-	-	-	-	-	-	-	-	-	-	-	-	-	-	-	-	1	-	1
P	SGK1	6446	NM_001143677	49.7505	-	-	-	-	-	-	-	-	-	-	1	-	-	-	-	1	-	2	-	-	-	-	-	-	-	-	-	-	-	-	-	-	-	-	-	-	-	-	-	1	-	1
P	SGK1	6446	NM_001143678	49.7505	-	-	-	-	-	-	-	-	-	-	1	-	-	-	-	1	-	2	-	-	-	-	-	-	-	-	-	-	-	-	-	-	-	-	-	-	-	-	-	1	-	1
P	SKP2	6502	NM_005983	-	-	-	-	-	-	-	-	-	-	-	-	-	-	-	-	1	-	1	-	-	-	-	-	-	-	-	-	-	-	-	-	-	-	-	-	-	-	-	-	-	-	-
P	SKP2	6502	NM_001243120	-	-	-	-	-	-	-	-	-	-	-	-	-	-	-	-	1	-	1	-	-	-	-	-	-	-	-	-	1	-	1	-	-	-	-	-	-	-	-	-	1	-	1
P	SKP2	6502	NM_032637	-	-	-	-	-	-	-	-	-	-	-	-	-	-	-	-	-	-	-	-	-	-	-	-	-	-	-	-	-	-	-	-	-	-	-	-	-	-	-	-	1	-	1
P	SLC38A9	153129	NM_001258286	-	-	-	-	-	-	-	1	-	-	-	-	-	-	-	-	1	-	2	-	-	-	-	-	1	1	-	1	1	-	4	-	-	-	-	-	-	-	-	-	1	-	1
P	SLC38A9	153129	NM_173514	-	-	-	-	-	-	-	-	-	-	-	-	-	-	-	-	1	-	1	-	-	-	-	-	-	-	-	-	-	-	-	-	-	-	-	-	-	-	-	-	1	-	1
P	SLC38A9	153129	NM_001258287	-	-	-	-	-	-	-	-	-	-	-	-	-	-	-	-	1	-	1	-	-	-	-	-	1	1	-	-	1	-	3	-	-	-	-	-	-	-	-	1	1	-	2
P	SLC3A2	6520	NM_001012662	-	-	-	-	-	-	-	-	-	-	-	-	-	-	-	-	-	-	-	-	-	-	-	-	-	-	-	-	-	-	-	1	8	1790	1783	0.0285	1	-	1	-	1	1	4
P	SLC3A2	6520	NM_001013251	-	-	-	-	-	-	-	-	-	-	-	-	-	-	-	-	-	-	-	-	-	-	-	-	-	-	-	-	-	-	-	1	8	1378	1371	0.0240	1	-	1	-	1	1	4
P	SLC3A2	6520	NM_001012664	-	-	-	-	-	-	-	-	-	-	-	-	-	-	-	-	-	-	-	-	-	-	-	-	-	-	-	-	-	-	-	1	8	1601	1594	0.0257	1	-	1	-	1	1	4
P	SLC3A2	6520	NM_002394	-	-	-	-	-	-	-	-	-	-	-	-	-	-	-	-	-	-	-	-	-	-	-	-	-	-	-	-	-	-	-	1	8	1787	1780	0.0285	1	-	1	-	1	1	5
P	SLC7A5	8140	NM_003486	57.4833	-	-	-	-	-	1	1	1	-	-	1	-	-	1	1	1	1	8	-	-	-	-	-	-	-	-	-	-	-	-	-	-	-	-	-	-	-	-	-	-	-	-
P	PPM1E	22843	NM_014906	48.6559	-	-	-	-	-	1	1	1	-	-	-	-	1	1	-	1	1	7	-	-	-	-	-	-	-	-	-	-	-	-	-	-	-	-	-	-	-	-	-	1	-	1
P	PPM1E	22843	XM_005257169	48.6559	-	-	-	-	-	1	-	1	-	-	-	-	-	-	-	1	1	4	-	-	-	-	-	-	-	-	-	-	-	-	-	-	-	-	-	-	-	-	-	1	-	1
P	PPM1F	9647	NM_014634	-	-	-	-	-	-	-	-	-	-	-	-	-	-	-	1	1	-	2	-	-	-	-	-	-	-	-	-	-	-	-	-	-	-	-	-	1	-	-	1	1	1	4
P	PPM1F	9647	XM_005261861	-	-	-	-	-	-	-	-	-	-	-	-	-	-	-	-	1	-	1	-	-	-	-	-	-	-	-	-	-	-	-	-	-	-	-	-	1	-	-	-	1	1	3
P	STK11	6794	XM_005259618	38.6141	-	-	-	-	-	1	-	-	-	-	1	-	-	-	1	1	-	4	-	-	-	-	-	-	-	-	-	1	-	1	-	-	-	-	-	-	-	-	-	1	-	1
P	STK11	6794	NM_000455	38.6141	-	-	-	-	-	1	-	-	-	-	1	-	-	1	-	1	-	4	-	-	-	-	-	-	-	-	-	1	-	1	-	-	-	-	-	-	-	-	-	1	-	1
P	STK11	6794	XM_005259617	38.6141	-	-	-	-	-	1	-	-	-	-	1	-	-	-	-	1	-	3	-	-	-	-	-	-	-	-	-	1	-	1	-	-	-	-	-	-	-	-	-	1	-	1
P	TELO2	9894	NM_016111	-	-	-	-	-	-	-	-	-	-	-	-	-	-	-	1	1	-	2	-	-	-	-	-	-	-	-	-	-	-	-	-	-	-	-	-	1	-	-	1	1	-	3
P	TSC1	7248	NM_000368	94.1139	-	-	-	-	-	1	-	1	-	1	1	-	1	1	-	1	1	8	-	-	-	-	-	-	1	-	-	1	-	2	-	-	-	-	-	1	-	-	1	1	1	5
P	TSC1	7248	XM_005272211	94.1139	-	-	-	-	-	1	1	1	-	-	1	-	-	-	1	1	1	7	-	-	-	-	-	-	1	-	1	1	-	3	-	-	-	-	-	1	-	-	-	1	-	2
P	TSC1	7248	NM_001162427	94.1139	-	-	-	-	-	1	-	1	-	1	1	-	1	-	-	1	1	7	-	-	-	-	-	-	1	-	-	1	-	2	-	-	-	-	-	1	-	-	-	1	-	2
P	TSC1	7248	NM_001162426	94.1139	-	-	-	-	-	1	-	1	-	1	1	-	1	-	-	1	1	7	-	-	-	-	-	-	1	-	-	1	-	2	-	-	-	-	-	1	-	-	-	1	-	2
P	TSC1	7248	XM_005272212	94.1139	-	-	-	-	-	1	-	1	-	-	1	-	-	-	-	1	1	5	-	-	-	-	-	-	-	-	-	-	-	-	-	-	-	-	-	1	-	-	-	1	-	2
P	TSC2	7249	XM_005255527	-	-	-	-	-	-	-	1	-	-	-	-	-	-	-	1	-	-	2	-	-	-	-	-	-	-	-	-	-	-	-	-	-	-	-	-	1	-	1	-	1	1	4
P	TSC2	7249	XM_005255534	-	-	-	-	-	-	-	-	-	-	-	-	-	-	-	-	-	-	-	-	-	-	-	-	-	-	-	-	-	-	-	-	-	-	-	-	1	-	1	-	1	1	4
P	TSC2	7249	NM_001077183	-	-	-	-	-	-	-	-	-	-	-	-	-	-	-	-	-	-	-	-	-	-	-	-	-	-	-	-	-	-	-	-	-	-	-	-	1	-	1	-	1	1	4
P	TSC2	7249	NM_001114382	-	-	-	-	-	-	-	-	-	-	-	-	-	-	-	-	-	-	-	-	-	-	-	-	-	-	-	-	-	-	-	-	-	-	-	-	1	-	1	-	1	1	4
P	TSC2	7249	NM_000548	-	-	-	-	-	-	-	-	-	-	-	-	-	-	-	-	-	-	-	-	-	-	-	-	-	-	-	-	-	-	-	-	-	-	-	-	1	-	1	-	1	1	4

	P	***TSC2***	7249	XM_005255533	-	-	-	-	-	-	-	-	-	-	-	-	-	-	-	-	-	-	-	-	-	-	-	-	-	-	-	-	-	-	-	-	-	-	-	1	-	1	-	1	1	4	
	P	***TSC2***	7249	XM_005255532	-	-	-	-	-	-	-	-	-	-	-	-	-	-	-	-	-	-	-	-	-	-	-	-	-	-	-	-	-	-	-	-	-	-	-	1	-	1	-	1	1	4	
	P	***TSC2***	7249	XM_005255531	-	-	-	-	-	-	-	-	-	-	-	-	-	-	-	-	-	-	-	-	-	-	-	-	-	-	-	-	-	-	-	-	-	-	-	1	-	1	-	1	1	4	
	P	***TSC2***	7249	XM_005255530	-	-	-	-	-	-	-	-	-	-	-	-	-	-	-	-	-	-	-	-	-	-	-	-	-	-	-	-	-	-	-	-	-	-	-	1	-	1	-	1	1	4	
	P	***TSC2***	7249	XM_005255529	-	-	-	-	-	-	-	-	-	-	-	-	-	-	-	-	-	-	-	-	-	-	-	-	-	-	-	-	-	-	-	-	-	-	-	1	-	1	-	1	1	4	
	P	***TSC2***	7249	XM_005255528	-	-	-	-	-	-	-	-	-	-	-	-	-	-	-	-	-	-	-	-	-	-	-	-	-	-	-	-	-	-	-	-	-	-	-	1	-	1	-	1	-	2	
	P	***PRKAA1***	5562	NM_206907	-	-	-	-	-	-	-	-	-	-	-	1	-	-	-	1	1	-	3	-	-	-	-	-	-	-	-	-	-	-	-	-	-	-	-	-	1	-	-	1	1	1	4	
	P	***PRKAA1***	5562	XM_005248320	-	-	-	-	-	-	-	-	-	-	-	1	-	-	-	-	1	-	2	-	-	-	-	-	-	-	-	-	-	-	-	1	7	173	167	0.0390	1	-	-	-	1	1	3	
	P	***PRKAA1***	5562	NM_006251	-	-	-	-	-	-	-	-	-	-	-	1	-	-	-	-	1	-	2	-	-	-	-	-	-	-	-	-	-	-	-	-	-	-	-	-	1	-	-	-	1	1	3	
	V	***PRKAA2***	5563	NM_006252	69.5627	-	-	-	-	-	1	1	1	-	-	1	-	-	1	-	1	1	7	-	-	-	-	-	-	-	-	-	1	-	1	-	-	-	-	-	-	1	-	1	1	1	4	***doi: 10.3892/or.2016.5199***
	P	***PPARGC1A***	10891	XM_005248133	50.7709	-	-	-	-	-	1	1	-	-	-	1	-	-	-	1	1	-	5	-	-	-	-	-	-	-	-	1	-	-	1	-	-	-	-	-	1	-	-	1	1	-	3	
	P	***PPARGC1A***	10891	NM_013261	50.7709	-	-	-	-	-	1	-	-	-	-	1	-	-	1	-	1	-	4	-	-	-	-	-	-	-	-	-	1	-	1	-	-	-	-	-	1	-	-	-	1	-	2	
	P	***PPARGC1A***	10891	XM_005248134	50.7709	-	-	-	-	-	1	-	-	-	-	1	-	-	-	-	1	-	3	-	-	-	-	-	-	-	-	-	-	-	-	-	-	-	-	-	1	-	-	-	1	-	2	
	P	***PPARGC1A***	10891	XM_005248132	50.7709	-	-	-	-	-	1	-	-	-	-	1	-	-	-	-	1	-	3	-	-	-	-	-	-	-	-	-	-	-	-	-	-	-	-	-	1	-	-	-	1	-	2	
	P	***PPARGC1A***	10891	XM_005248130	50.7709	-	-	-	-	-	1	-	-	-	-	1	-	-	-	-	1	-	3	-	-	-	-	-	-	-	-	-	-	-	-	-	-	-	-	-	1	-	-	-	1	-	2	
	P	***PPARGC1A***	10891	XM_005248131	50.7709	-	-	-	-	-	1	-	-	-	-	1	-	-	-	-	1	-	3	-	-	-	-	-	-	-	-	-	-	-	-	-	-	-	-	-	1	-	-	-	1	-	2	
	P	***PTEN***	5728	NM_000314	-	-	-	-	-	-	-	1	-	-	-	-	-	-	-	-	1	-	2	-	-	-	-	-	-	-	-	-	1	-	1	-	-	-	-	-	-	-	-	-	1	-	1	
	V	***PINK1***	65018	NM_032409	53.7877	1	7	1908	1902	0.0500	1	1	-	-	-	1	-	1	1	-	1	1	6	-	-	-	-	-	-	-	-	-	1	-	1	-	-	-	-	-	-	-	-	-	1	-	1	***doi.org/10.1186/s13024-016-0121-4***
	V	***FBXW7***	55294	NM_033632	98.972	1	8	3672	3665	0.0247	1	1	1	-	1	1	1	1	-	1	1	1	10	-	-	-	-	-	-	-	-	-	-	-	-	-	-	-	-	-	1	-	-	1	1	-	3	
	V	***FBXW7***	55294	NM_001013415	98.972	1	8	3333	3326	0.0245	1	-	1	-	1	1	1	1	-	-	1	1	8	-	-	-	-	-	-	-	-	-	-	-	-	-	-	-	-	-	1	-	1	-	1	1	4	
	V	***FBXW7***	55294	NM_018315	98.972	-	-	-	-	-	1	-	1	-	1	1	1	1	-	-	1	1	8	-	-	-	-	-	-	-	-	-	-	-	-	-	-	-	-	-	1	-	-	-	1	-	2	
	V	***FBXW7***	55294	XM_005263108	98.972	1	8	3054	3047	0.0245	1	-	1	-	-	1	1	-	-	-	1	1	6	-	-	-	-	-	-	-	-	-	-	-	-	-	-	-	-	-	1	-	-	-	1	-	2	
	V	***FBXW7***	55294	NM_001257069	98.972	-	-	-	-	-	-	-	-	-	-	1	1	-	-	-	-	-	2	-	-	-	-	-	-	-	-	-	-	-	-	-	-	-	-	-	-	-	-	-	1	-	1	
**chemotherapy response and survival**	P	***ERCC1***	2067	NM_001983	-	-	-	-	-	-	1	1	-	-	-	1	-	-	-	1	1	-	5	-	-	-	-	-	-	-	-	-	-	-	-	-	-	-	-	-	-	-	-	1	1	-	2	
	P	***ERCC1***	2067	XM_005258638	-	-	-	-	-	-	1	-	-	-	-	1	-	-	-	-	1	-	3	-	-	-	-	-	-	-	-	-	-	-	-	-	-	-	-	-	-	-	-	-	1	-	1	
	P	***ERCC1***	2067	NM_001166049	-	-	-	-	-	-	1	-	-	-	-	1	-	-	-	-	1	-	3	-	-	-	-	-	-	-	-	-	-	-	-	-	-	-	-	-	-	-	-	-	1	-	1	
	P	***ERCC1***	2067	XM_005258637	-	-	-	-	-	-	-	-	-	-	-	1	-	-	-	-	-	-	1	-	-	-	-	-	-	-	-	-	-	-	-	-	-	-	-	-	1	-	-	-	1	-	2	
	P	***ERCC1***	2067	XM_005258634	-	-	-	-	-	-	-	-	-	-	-	1	-	-	-	-	-	-	1	-	-	-	-	-	-	-	-	-	-	-	-	-	-	-	-	-	1	-	-	-	1	-	2	
	P	***ERCC1***	2067	NM_202001	-	-	-	-	-	-	-	-	-	-	-	1	-	-	-	-	-	-	1	-	-	-	-	-	-	-	-	-	1	-	1	-	-	-	-	-	1	-	-	-	1	-	2	
	P	***ERCC1***	2067	XM_005258636	-	-	-	-	-	-	-	-	-	-	-	1	-	-	-	-	-	-	1	-	-	-	-	-	-	-	-	-	-	-	-	-	-	-	-	-	1	-	-	-	1	-	2	
	P	***ERCC1***	2067	XM_005258635	-	-	-	-	-	-	-	-	-	-	-	1	-	-	-	-	-	-	1	-	-	-	-	-	-	-	-	-	-	-	-	-	-	-	-	-	1	-	-	-	1	-	2	
	P	***ERCC4***	2072	NM_005236	66.6166	-	-	-	-	-	-	1	-	-	-	1	-	-	-	1	1	-	4	-	-	-	-	-	-	-	-	-	-	-	-	-	-	-	-	-	-	-	-	-	1	-	1	
	V	***DPYD***	1806	NM_000110	70.2679	-	-	-	-	-	1	1	-	-	-	1	1	1	1	1	1	1	9	-	-	-	-	-	-	-	-	-	-	-	-	-	-	-	-	-	1	1	1	1	1	1	6	***doi: 10.1158/1535-7163.***
	V	***DPYD***	1806	NM_001160301	70.2679	-	-	-	-	-	1	-	-	-	-	1	1	-	-	-	1	1	5	-	-	-	-	-	-	-	-	-	-	-	-	-	-	-	-	-	1	-	-	-	1	-	2	
	V	***DPYD***	1806	XM_005270562	70.2679	-	-	-	-	-	1	-	-	-	-	1	1	-	-	-	1	1	5	-	-	-	-	-	-	-	-	-	-	-	-	-	-	-	-	-	1	-	1	-	1	1	4	
	V	***DPYD***	1806	XM_005270561	70.2679	-	-	-	-	-	1	-	-	-	-	1	1	-	-	-	1	1	5	-	-	-	-	-	-	-	-	-	-	-	-	-	-	-	-	-	1	-	1	-	1	1	4	
	V	***DPYD***	1806	XM_005270564	70.2679	-	-	-	-	-	-	-	-	-	-	1	1	-	-	-	-	-	2	-	-	-	-	-	-	-	-	-	-	-	-	-	-	-	-	-	1	-	-	-	1	-	2	
	V	***DPYD***	1806	XM_005270563	70.2679	-	-	-	-	-	-	-	-	-	-	1	1	-	-	-	-	-	2	-	-	-	-	-	-	-	-	-	-	-	-	-	-	-	-	-	1	-	-	-	1	-	2	
**Other**	V	***SPRY2***	10253	NM_005842	85.0835	1	8	1718	1711	0.0121	1	-	1	-	1	1	-	1	1	-	1	1	8	-	-	-	-	-	-	-	-	-		-		-	-	-	-	-	1	-	-	1	1	-	3	***doi.org/10.1016/j.cdtm.2016.11.012***
	V	***SPRY2***	10253	XM_005266217	85.0835	1	8	2273	2266	0.0119	1	1	1	-	-	1	-	-	-	-	1	1	6	-	-	-	-	-	-	-	-	-	1	-	1	-	-	-	-	-	1	-	-	-	1	-	2	
